# Edge Transport in Haldane-Like Models with Quasi-Periodic Disorder

**DOI:** 10.1007/s00220-026-05560-1

**Published:** 2026-07-09

**Authors:** Fabrizio Caragiulo, Vieri Mastropietro, Marcello Porta

**Affiliations:** 1 SISSA, Via Bonomea 265, 34136 Trieste, Italy; 2 University of Rome Sapienza, P.le Aldo Moro 2, 00185 Roma, Italy

## Abstract

We consider two-dimensional topological insulators on the cylinder, in the presence of weak quasi-periodic disorder. In the absence of disorder, we assume the presence of a single edge state on the boundary of the system, as for the Haldane model. We prove that, at large distances, the boundary correlations agree with the correlations of a renormalized, translation-invariant, massless relativistic model in 1+1 dimensions, multiplied by non-universal oscillatory factors, incommensurate with the lattice spacing. Furthermore, we compute the edge conductance and the edge susceptibility, starting from Kubo formula. We obtain explicit expressions for these response functions, completely determined by the renormalized Fermi velocity of the edge modes. In particular, we prove the quantization of the edge conductance, and the non-universality of the susceptibility. The proof relies on multiscale analysis and rigorous renormalization group methods for quasi-periodic systems, and on lattice Ward identities.

## Introduction

One of the hallmarks of topological phases in condensed matter physics is the presence of stable currents at the boundary of the material. First predicted to arise for quantum Hall systems [31], these conducting states are called *edge modes*, and their very existence and stability is deeply related to the value of suitable bulk topological invariants labelling the phase of the system. More precisely, the *bulk-edge duality* for quantum Hall systems [16, 17, 32, 56] equates the signed number of such currents, where the sign takes into account the direction of propagation, to the integer value of the bulk Hall conductivity, in suitable units. In the last years, this remarkable duality has been extended to a large class of noninteracting topological materials; see e.g. [54] for a review.

Concerning the rigorous analysis of the edge modes, spectral theoretic methods have been used to study the stability of the associated continuous spectrum against perturbations; see e.g. [9, 11, 14, 21, 34, 43]. It is an interesting open question to obtain a more precise characterization of the edge modes for disordered systems, and to compute boundary observables. The goal of this paper is to obtain a quantitative understanding of the edge modes for in presence of weak disorder, by computing edge correlation functions, edge scaling exponents, and real-time transport coefficients.

At a formal level, these objects can be investigated adopting an effective quantum field theory (QFT) viewpoint [20, 61]. In the last years there has been progress in the rigorous justification of the chiral Luttinger liquid as an effective one-dimensional theory for the boundary of weakly interacting two-dimensional quantum Hall systems. The works [3, 48] put this reduction on rigorous grounds via rigorous renormalization group (RG) methods, for systems that are translationally invariant in the direction of the edge. It is an important open problem to develop rigorous RG techniques that allow to study interacting and disordered systems, and to understand the scaling limit of the disordered correlation functions.

In this work, we develop a RG method for a weakly disordered, non-interacting two-dimensional quantum systems on a cylinder with a single edge-mode per cylinder edge, thus tackling the problem of breaking the translation invariance. It is important to stress that the model we consider, even after the one-dimensional reduction, does not belong to the class of Schrödinger operators considered in the very large literature about quasi-periodic systems, from the seminal works [15, 18, 50] to more recent breakthroughs such as [5–7]. We will assume that the non-disordered Hamiltonian is finite-range, translationally invariant along the periodic direction of the cylinder, and that every edge supports one edge mode. A model fitting the picture is the Haldane model [30], a paradigmatic example of topological insulator. We will then perturb the model adding a weak quasi-periodic potential, whose frequency is incommensurate with the lattice spacing. Mathematically, we will assume that the frequency is a Diophantine number; these numbers are badly approximated by rational numbers, in a quantitative sense discussed below, and form a set of full measure in the reals.

**Results.** We obtain a precise characterization of the edge modes in presence of weak disorder by combining RG with a KAM-like analysis for the control of the small divisors introduced by the quasi-periodic potential. Our method allows us to compute boundary correlation functions and to extract their scaling limit; this turns out to agree with one dimensional relativistic fermions, with renormalized parameters, but also modulated by quasi-periodic amplitudes. While considering a non-interacting case is a simplification with respect to the many applications of fermionic RG of the last years, the presence of the quasi-periodic disorder breaks translation invariance and potentially introduces infinitely many new infrared singularities in the multiscale analysis. Thanks to the Diophantine assumption on the modulation frequency of the potential, we can ultimately control all these singularities, and prove that the net effect of the disorder is to renormalize the Fermi velocity, to shift the Fermi points, and to dress the correlation functions by oscillatory factors. The knowledge of the two-point function allows one to fully characterize all edge correlation functions, via the fermionic Wick rule. This is our first result. Notice that, in contrast to the widely studied quasi-periodic models in one dimension, where infinitely many gaps open as soon as the disorder is turned on, here the system is always gapless for all values of the chemical potential within the bulk spectral gap, in the sense that the two-point correlation function never decays exponentially, but always algebrically. The physical reason for this difference is that at least two counter-propagating edge-modes are needed to possibly backscatter and open a gap.

Our second result is an explicit computation of the Kubo edge conductance and of the Kubo edge susceptibility, defined as real-time linear response of the current and of the density operators to an external, slowly-varying perturbation supported in proximity of the boundary. To the best of our knowledge, this is the first time that the real-time linear response is rigorously evaluated for the edge modes of non-translation invariant systems. The values of the transport coefficients turn out to be completely determined by the renormalized Fermi velocity, a quantity defined by the scaling limit of the two-point function. In particular, the Kubo edge conductance turns out to be exactly quantized. As a corollary, for the class of systems considered in the paper, our result allows us to lift the bulk-edge duality for quasi-periodic systems at the level of equality of real-time Kubo formulas. Instead, the Kubo edge susceptibility is non-universal; nevertheless, it has a simple expression is terms of the effective Fermi velocity of the edge modes.

These results are achieved via the combination of RG methods with lattice conservation laws, for non-translation invariant systems. An important part of the proof is to show that, on large scales, the transport coefficients can be approximated by those of a renormalized, translation-invariant model. A major difficulty in proving this is introduced by the oscillatory factors that arise in the scaling of all edge correlation functions, which apparently break translation-invariance on large scales as well. To deal with this issue, we use lattice conservation laws to establish nontrivial relations between the amplitudes of the modes of the oscillatory functions appearing in the scaling limit of the two-point function and the Fermi velocity. These relations imply remarkable cancellations, which ultimately allow us to explicitly compute the edge transport coefficients.

From the physics viewpoint, our analysis is motivated by the recent progress in condensed matter physics, where lattice distortions, Moiré superlattices and magnetic impurities are often described in terms of quasi-periodic potentials; see for instance [12, 22, 36, 37, 40, 44, 51–53, 57, 58, 60], as well as the experimental works [33, 35, 59]. With respect to random systems, it is worth mentioning that quasiperiodic systems present different challenges, due to the fact that many tools used in the random case (such as the replica trick in the physics literature) are no longer applicable.

**Comparison with the literature.** The bulk-edge correspondence has been studied in many settings, using non-commutative geometry and *K*-theoretic ideas [2, 41, 54, 56], and using functional analytic methods [16, 17, 28]. These works establish the equality between the bulk Hall conductivity and a suitable edge index. The validity of the bulk-edge correspondence at the level of equality of bulk and edge transport coefficients has been recently investigated in [13], for non-interacting fermions, and in [42] for interacting fermions under a suitable assumption of local indistinguishability of the Gibbs state, known to hold at high enough temperatures. In the works [13, 42], the bulk Hall conductivity agrees with Kubo formula, while the edge conductance is defined as the variation of the edge current with respect to a change of the chemical potential in proximity of the boundary. In our work, the edge transport coefficient is defined via Kubo formula, as for the bulk transport. The edge Kubo formula describes the variation of the edge current after the *dynamical* introduction of a perturbation localized in proximity of the boundary, via an adiabatic switch on, in the linear response approximation. To the best of our knowledge, in the context of disordered systems, the edge Kubo formula is computed in the present paper for the first time. Concerning interacting, non-disordered systems, similar results have been obtained by two of us in [3, 48].

From the methodological viewpoint, the use of RG methods for quasi-periodic systems has been pioneered in [8], where the Schwinger functions of one-dimensional systems in the presence of weak quasi-periodic disorder have been studied at special values of the chemical potential, at which a gap opens. The analysis of the present paper is partially inspired by recent related work for the quasi-periodic critical Ising model [23]; however, the extension to the present case is nontrivial. The reference [23] studied the correlation functions for bilinear fermionic observables (such as the energy-energy correlations), while here we construct the two-point function, which gives access to all correlation functions via the fermionic Wick’s rule. Moreover, here we apply the methods to the evaluation of real-time transport coefficients, for the first time. Finally, similar methods have been applied in the past to study the localization and delocalization of one-dimensional interacting fermions with quasi-periodic disorder, for values of the density that rule out resonances between different Fermi points [45–47].

**Perspectives.** We believe that the methods of this paper open several interesting directions, such as the analysis of boundary currents in other models with quasi-periodic disorder, including non-interacting systems with multiple edge modes or interacting systems. The extension to several edge modes is far from being trivial, as the analysis of the almost-Mathieu model suggests: for suitable values of the chemical potential, counterpropagating chiral fermions are expected to annihilate, a phenomenon associated with mass generation from the QFT viewpoint. This of course cannot happen for all configurations of edge modes, since it would imply a violation of the bulk-edge duality. It would be interesting to have a RG perspective of the stability and on the disappearance of edge modes due to scattering induced by the disorder. Also, let us mention that the transport properties of interacting and disordered edge modes are not completely understood. For instance, we mention here the puzzle of the two-terminal conductance of the Hall bar: for interacting and translation-invariant systems, bosonization methods predict a non-universal result for materials with counterpropagating edge modes, in contrast with experiments. In order to recover the correct result, disorder should be taken into account; it has been suggested that disorder should drive the system to a different RG fixed point [38, 39], for which the quantization of the two-terminal conductance holds true. The rigorous formulation of this argument seems to be beyond present day techniques. Finally, it would be very interesting to extend the analysis of this paper to the case of weak random disorder, for instance using the supersymmetric (SUSY) representation of disordered systems; see [4] for a rigorous renormalization group analysis of a SUSY hierarchical model of weakly disordered three-dimensional semimetals, and [19] for the development of SUSY cluster expansion methods.

**Organization of the paper.** In Sect. 2 we introduce the class of models we consider, we define the Gibbs state and we state the assumptions on the non-disordered edge spectrum. We then introduce the response functions, and the Euclidean two-point function.

In Sect. 3 we state our main results, Theorem 3.1 about the two-point function, and Theorem 3.3 about the response functions, and we give a sketch of the proof of Theorem 3.3. The rest of the paper is devoted to the proofs; the article is essentially self-contained, and this contributes to its length.

In Sect. 4 we introduce the RG analysis, which is conveniently defined in the Grassmann representation of the model, and we prove Theorem 3.1. The main technical results of this section are Proposition 4.2, which constructs the effective Grassmann potential of the model at all scales, and Proposition 4.13, which extends the construction to the case of an external Grassmann field, needed in order to define the correlations.

In Sect. 5 we use the result about the two-point function to compute the edge response function, after the rigorous Wick rotation, and to prove Theorem 3.3.

**Fig. 1:**
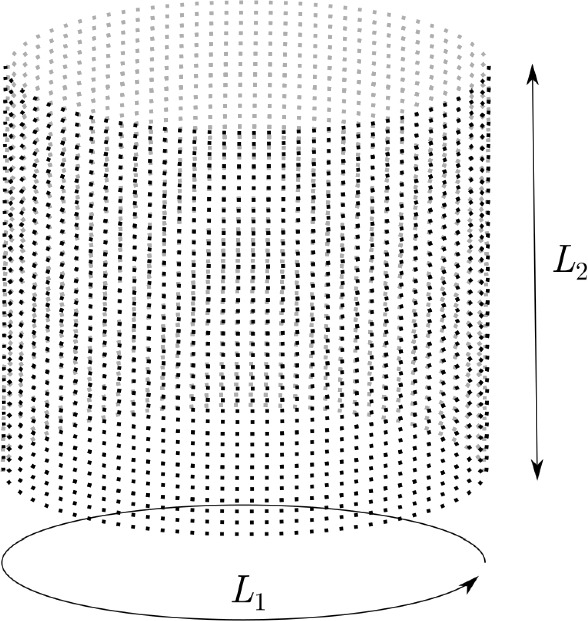
The lattice $$\Lambda _L$$

**Fig. 2:**
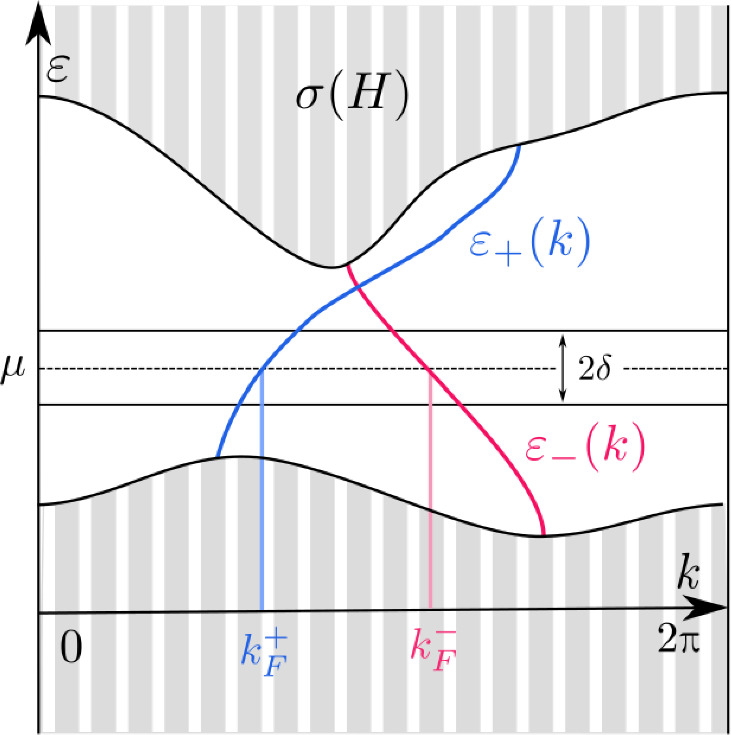
The colored lines corresponds to the edge modes, bulk spectrum sits inside the striped gray area

**Fig. 3:**
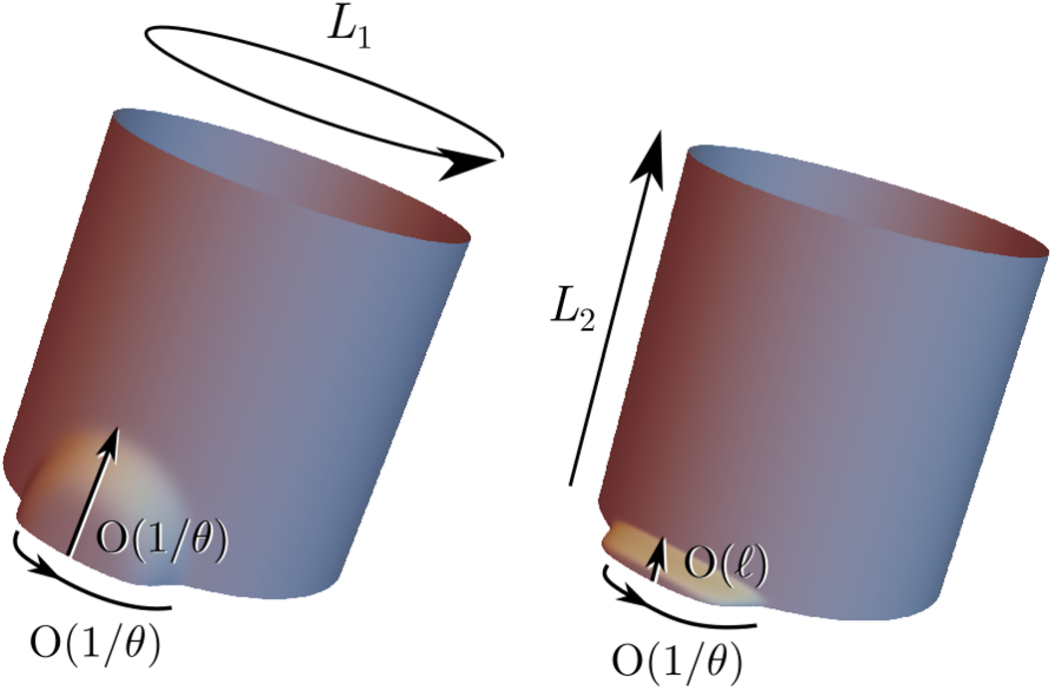
On the left is depicted the shape of $\mu (\theta \vec x)$ on the cylinder, on the right the shape of $\phi _{\ell }(\theta x_{1},x_{2})$

**Fig. 4:**
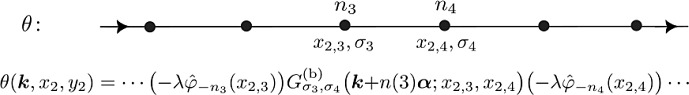
Example of chain graph

**Fig. 5:**
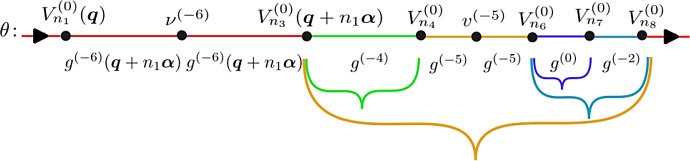
An example of a chain graph with 8 nodes and its clusters structure. For simplicity we omitted the ω labels and all arguments excepts a few ones. Each clusters is represented by a curly bracket

In Appendix A we prove an estimate for the propagator associated with the energies away from the Fermi level; in Appendix B we prove a recursion relation that allows us to determine the two-point function from the effective potential; in Appendix C we reproduce for completeness the computation of the relativistic bubble diagram, which plays a key role in the computation of the transport coefficients; while Appendices D, E collect technical details of auxiliary results in our proofs (Figs. 1, 2, 3, 4 and 5).

## The Model

### The Hamiltonian and the Gibbs State

Let $L_{1}, L_{2}\in \mathbb {N}$, $L = (L_{1}, L_{2})$, and consider the lattice:

2.1 $$\begin{aligned} \Lambda _{L} = \Big \{ \vec x \in \mathbb {Z}^{2} \Big |\; 1 \le x_{i} \le L_{i},\quad i =1,2 \Big \}. \end{aligned}$$

We shall equip the lattice with periodic boundary conditions in the direction 1, and Dirichlet boundary conditions in the direction 2. That is, we will consider functions $f(\vec{x})$ satisfying *cylindric boundary conditions:*

2.2 $$\begin{aligned} f(x_{1} + n L_{1}, x_{2}) = f(x_{1}, x_{2}),\quad f(x_{1}, 1) = f(x_{1}, L_{2}) = 0,\quad \text {for all } \vec x\in \Lambda _{L}. \end{aligned}$$

We will use the following distance between points on $\Lambda _{L}$:

2.3 $$\begin{aligned} \Vert \vec x - \vec y \Vert _{L}:= \min _{n\in \mathbb {Z}} \Vert \vec x - \vec y + n L e_{1} \Vert ,\qquad \text {for all } \vec x, \vec y \in \Lambda _{L}, \end{aligned}$$

with $\Vert \cdot \Vert$ the usual Euclidean norm on $\mathbb {R}^{2}$ and $e_{1} = (1,0)$, $e_{2} = (0,1)$.

We are interested in describing a fermionic lattice gas on $\Lambda _{L}$, in a grand-canonical setting. The fermionic Fock space is $\mathcal {F}_{L} = \bigoplus _{n\ge 0} \big (\ell ^{2}(\Lambda _{L}; \mathbb {C}^{S})\big )^{\wedge n}$, where the number $S \in \mathbb {N}$ denotes the total number of internal degrees of freedom, e.g. the spin label or the sublattice label. The Hamiltonian of the model is a self-adjoint operator on $\mathcal {F}_{L}$, of the following form:

2.4 $$\begin{aligned} \begin{aligned} \mathcal {H}&:= {\sum _{\vec x,\vec y\in \Lambda _{L}}\sum _{\sigma ,\zeta =1}^S {a}^*_{\vec x,\sigma } H_{\sigma ,\zeta }(\vec x,\vec y){a}_{\vec y,\zeta }} + \lambda \sum _{\vec x \in \Lambda _{L}} \sum _{\sigma = 1}^{S} \varphi (\vec x){a}^*_{\vec x,\sigma } {a}_{\vec x,\sigma } \;, \end{aligned} \end{aligned}$$

where $a^{*}, a$ are the usual fermionic creation and annihilation operators, *H* is a self-adjoint operator on $\ell ^{2}(\Lambda _{L}; \mathbb {C}^{S})$ (compatible with the cylindric boundary conditions), and $\lambda \varphi (\cdot )$ introduces a perturbation. The model describes an electron gas on a lattice in the presence of impurities, described by $\varphi (\cdot )$, and we will be interested in the equilibrium and in the transport properties of the model, in the linear response approximation. Let us specify the type of Hamiltonians *H* and perturbations φ that we shall consider.

#### Assumption 1

*(On the free Hamiltonian).* The Hamiltonian *H* is the periodization of a self-adjoint operator $H^{\infty }$ on $\ell ^{2}\big (\mathbb {Z} \times ([1,L_2] \cap \mathbb {Z}); \mathbb {C}^{S}\big )$. That is, for all $\vec x, \vec y\in \Lambda _{L}$:

2.5 $$\begin{aligned} H(\vec x, \vec y) = \sum _{n\in \mathbb {Z}} H^{\infty }\big (( x_{1} + n L_{1}, x_{2} ), (y_{1}, y_{2})\big ). \end{aligned}$$

We assume that $H^{\infty }$ is finite-range and translation invariant:

2.6 $$\begin{aligned} H^{\infty }(\vec x, \vec y) = 0 \quad \text {if } \Vert \vec x - \vec y\Vert > R, \qquad H^{\infty }(\vec x, \vec y) \equiv H^{\infty }(x_{1} - y_{1}; x_{2}, y_{2}). \end{aligned}$$

We will assume that $R = \sqrt{2}$. This is not a loss of generality, up to increasing the number of internal degrees of freedom *S*. Thus, *H* is also translation invariant, and finite-range: $H(\vec x, \vec y) = 0$, if $\Vert \vec x - \vec y \Vert _{L} > \sqrt{2}$.

Later, we will make further assumptions on the spectrum of *H*. We now define the type of perturbations we will consider.

#### Assumption 2

*(On the perturbation).* The function $\varphi (\cdot )$ is real-valued, and it has the form:

2.7 $$\begin{aligned} \varphi (x_{1},x_{2}) = \sum _{n\in \mathbb {Z}} e^{i n\alpha x_{1}} {\hat{\varphi }}_{n}(x_{2}),\qquad \text {with } | \hat{\varphi }_{n}(x_{2}) | \le Ce^{-c|n|} \text { uniformly in } x_{2}. \end{aligned}$$

Also, ${\hat{\varphi }}_{n}(x_{2})$ satisfies the Dirichlet boundary conditions. Furthermore, the number $\alpha /2\pi = m / L_{1}$ is the best rational approximant (in the sense of convergents of the continued fraction expansion, see e.g. [8, Appendix 1]), with denominator $L_{1}$, of a real number $\alpha _{\infty }/2\pi$ which is Diophantine. That is: there exists positive constants ${\tilde{c}}, \tau$ such that:

2.8 $$\begin{aligned} | n\alpha _{\infty } |_{\mathbb{T}} \ge \frac{\tilde{c}}{|n|^{\tau }},\qquad \text {for all } n\ne 0. \end{aligned}$$

#### Remark 2.1

(i) The function $\varphi (\vec x)$ describes a quasi-periodic perturbation. Notice that we are not making any assumption about the $x_{2}$ dependence of the perturbation.
(ii) By construction, $\varphi (\vec x)$ is compatible with the periodic boundary conditions. The thermodynamic limit is taken over sequences $\{L_{1}\}$ such that $\text {mcd}(m, L_{1}) = 1$.
(iii) The approximant α satisfies the following version of the bound (2.8). There exists c>0 such that for every n≠0, $|n| \le L_{1} / 2$:
  2.9 $$\begin{aligned} | n \alpha |_{\mathbb {T}} \ge \frac{c}{|n|^{\tau }}. \end{aligned}$$
  Furthermore, by the properties of the continued fractions expansion which is used to construct α (see e.g. [10]), we also have $| \alpha - \alpha _{\infty } | \le L_{1}^{-2}$.

Next, let us introduce the Gibbs state associated with the Hamiltonian (2.4). Given an operator $\mathcal {O}$ on $\mathcal {F}_{L}$, we define:

2.10 $$\begin{aligned} \langle \mathcal {O} \rangle _{\beta , \mu , L}:= \frac{{{\,\textrm{Tr}\,}}\big ( \mathcal {O} \, e^{-\beta (\mathcal {H} - \mu \mathcal {N})} \big ) }{\mathsf Z_{\beta , \mu , L}},\qquad \mathsf Z_{\beta ,\mu ,L}:= {{\,\textrm{Tr}\,}}e^{-\beta (\mathcal {H} - \mu \mathcal {N})}, \end{aligned}$$

with β>0 the inverse temperature and $\mu \in \mathbb {R}$ the chemical potential. The operator $\mathcal {N}$ is the number operator, $\mathcal {N} = \sum _{\sigma , \vec x} a^{*}_{\vec x, \sigma } a_{\vec x, \sigma }$. The Heisenberg evolution generated by $\mathcal {H}$ is denoted by $\tau _{t}(\mathcal {O}) = e^{i\mathcal {H} t} \mathcal {O} \,e^{-i\mathcal {H}t}$, which leaves the Gibbs state trivially invariant, $\langle \tau _{t}(\mathcal {O}) \rangle _{\beta , \mu , L} = \langle \mathcal {O} \rangle _{\beta , \mu , L}$.

Next, we will make an assumption on the form of the spectrum of the unperturbed Hamiltonian *H* in proximity of the chemical potential μ. Let $\mathscr {D}_{L}:= \frac{2\pi }{L_{1}} \big (\mathbb {Z} / L_{1}\mathbb {Z}\big )$ be the independent momenta associated with the periodic boundary conditions in $x_{1}$. As $L_{1}\rightarrow \infty$, these points fill densely the circle of length 2π, $\mathbb {T}$. Recall the definition of partial Fourier transform for a function $f(x_{1}, x_{2})$ on $\Lambda _{L}$, for $k_{1} \in \mathscr {D}_{L}$:

2.11 $$\begin{aligned} {\hat{f}}(k_{1}, x_{2}) = \sum _{x_{1} = 1}^{L_{1}} e^{-i k_{1} x_{1}} f(x_{1}, x_{2}),\qquad f(x_{1}, x_{2}) = \frac{1}{L_{1}} \sum _{k_{1} \in \mathscr {D}_{L}} e^{ik_{1} x_{1}} {\hat{f}}(k_{1}, x_{2}). \nonumber \\ \end{aligned}$$

Let us denote the partial Bloch decomposition ${\hat{H}}(k_{1})$ of *H* as the operator such that:

2.12 $$\begin{aligned} \widehat{(H f)}(k_{1}, x_{2}) = \big ({\hat{H}}(k_{1}) {\hat{f}}(k_{1})\big )(x_{2}). \end{aligned}$$

Observe that ${\hat{H}}(k_{1})$ is a self-adjoint operator acting on functions on $[1, L_{2}]\cap {\mathbb {Z}}$. Explicitly,

2.13 $$\begin{aligned} {\hat{H}}_{\sigma ,\zeta }(k_{1}; x_{2}, y_{2}) = \sum _{z_{1}=1}^{L_{1}} e^{-i k_{1} z_{1}} H_{\sigma ,\zeta }\big ((x_{1}+z_{1}, x_{2}), (x_{1}, y_{2})\big ). \end{aligned}$$

#### Remark 2.2

(i) Being *H* the periodization of $H^{\infty }$, see Eq. (2.5), it follows that:
  2.14 $$\begin{aligned} {\hat{H}}(k_{1}) = {\hat{H}}^{\infty }(k_{1})\qquad \text {for all } k_{1} \in \mathscr {D}_{L}. \end{aligned}$$
(ii) Thanks to the finite range property of $H^{\infty }$, ${\hat{H}}^{\infty }(k_{1})$ is analytic in $k_{1}$.

We will also adopt the following conventions for the Fourier transforms of the creation and annihilation operators:

2.15 $$\begin{aligned} {\hat{a}}_{k_{1}, x_{2}, \sigma } = \sum _{x_{1} = 1}^{L_{1}} e^{-ik_{1} x_{1}} a_{\vec x,\sigma },\qquad {\hat{a}}^{*}_{k_{1}, x_{2}, \sigma } = \sum _{x_{1} = 1}^{L_{1}} e^{ik_{1} x_{1}} a^{*}_{\vec x,\sigma }, \end{aligned}$$

which can be inverted as:

2.16 $$\begin{aligned} a_{\vec x,\sigma } = \frac{1}{L_{1}} \sum _{k_{1} \in \mathscr {D}_{L}} e^{i k_{1} x_{1}} {\hat{a}}_{k_{1}, x_{2}, \sigma },\qquad a^{*}_{\vec x,\sigma } = \frac{1}{L_{1}} \sum _{k_{1} \in \mathscr {D}_{L}} e^{-i k_{1} x_{1}} {\hat{a}}^{*}_{k_{1}, x_{2}, \sigma }. \end{aligned}$$

Observe that, with these definitions, the second-quantized unperturbed Hamiltonian can be written as:

2.17 $$\begin{aligned} \sum _{\vec x,\vec y}\sum _{\sigma ,\zeta } {a}^*_{\vec x,\sigma } H_{\sigma ,\zeta }(\vec x,\vec y){a}_{\vec y,\zeta } = \frac{1}{L_{1}} \sum _{k_{1}} \sum _{x_{2}, y_{2}} \sum _{\sigma ,\zeta } {\hat{a}}^{*}_{k_{1}, x_{2}, \sigma } {\hat{H}}_{\sigma ,\zeta }(k_{1}; x_{2}, y_{2}) {\hat{a}}_{k_{1}, y_{2}, \zeta }.\nonumber \\ \end{aligned}$$

The next assumption specifies properties of the spectrum of *H* in a neighbourhood of the chemical potential μ. In particular, we will require that the model supports edge states, on the boundary of the cylinder $\Lambda _{L}$.

#### Assumption 3

*(On the spectrum of* *H* *in proximity of* μ.*)*. Consider the eigenvalue equation for ${\hat{H}}^{\infty }(k_{1})$:

2.18 $$\begin{aligned} {\hat{H}}^{\infty }(k_{1}) \xi (k_{1}) = \varepsilon (k_{1}) \xi (k_{1})\qquad \text {for } k_{1} \in \mathbb {T}, \end{aligned}$$

with $\xi (k_{1}) \in \ell ^{2}\big ( [1, L_{2}]\cap {\mathbb {Z}}; \mathbb {C}^{S}\big )$ satisfying the Dirichlet boundary conditions. We suppose that, for our choice of the chemical potential μ, there exists δ>0, independent of $L_{2}$, such that the following is true. Let $(\xi (k_{1}), \varepsilon (k_{1}))$ be a solution of (2.18) with $\varepsilon (k_{1}) \in (\mu -\delta , \mu +\delta )$.

(i) The eigenvalue $\varepsilon (k_{1})$ is smooth in $k_{1}$. We shall suppose that there are two and only two such functions in the energy range $(\mu - \delta , \mu + \delta )$, that we call $\varepsilon _{\omega }(k_{1})$ with $\omega = \pm$.
(ii) We call Fermi points $k_{F}^{\omega }$ the solutions of $\varepsilon _{\omega }(k_{F}^{\omega }) = \mu$. Let $v_{\omega }$ be the Fermi velocity associated with $k_{F}^{\omega }$, $v_{\omega } = \partial _{k_{1}} \varepsilon _{\omega }(k_{F}^{\omega })$. We shall suppose that $v_{\omega } \ne 0$. Up to choosing a smaller δ, we assume that $\partial _{k_{1}}\varepsilon _{\omega }(k_{1}) \ne 0$ as long as $\varepsilon _{\omega }(k_{1})\in (\mu - \delta , \mu + \delta )$.
(iii) Let $\xi ^{\omega }(k_{1})$ be the eigenfunction associated with $\varepsilon _{\omega }(k_{1})$. Then:
  2.19 $$\begin{aligned} | \partial _{k_{1}}^{r} \xi _{\sigma }^{+}(k_{1};x_{2}) | \le C_{r}e^{-{\tilde{c}}x_{2}}\quad \text {and} \quad | \partial _{k_{1}}^{r} \xi _{\sigma }^{-}(k_{1}; x_{2}) | \le C_{r}e^{-{\tilde{c}}(L_{2} - x_{2})}. \end{aligned}$$
(iv) We shall assume that $H^{\infty }$ is the restriction to the strip of width $L_{2}$ of a finite-range Hamiltonian $\widetilde{H}^{\infty }$ on $\mathbb {Z}^{2}$, satisfying (2.6).

#### Remark 2.3

The last assumption easily implies that, for any $k_{1} \in \mathbb {T}$, for any $x_{2}, y_{2} \in \mathbb {N}$ and for any $z \in \mathbb {C}$ such that $\inf _{L_{2}}\text {dist}(z, \sigma ({\hat{H}}^{\infty }(k_{1})))>0$:

2.20 $$\begin{aligned} \lim _{L_{2} \rightarrow \infty } {\hat{H}}^{\infty }(k_{1}; x_{2}, y_{2})\qquad \text {and}\quad \lim _{L_{2}\rightarrow \infty } \frac{1}{z - {\hat{H}}^{\infty }(k_{1})}(x_{2};y_{2}) \qquad \text {exist.} \end{aligned}$$

In particular, this information will be used to infer the existence of the $L_{2}\rightarrow \infty$ limit of the Gibbs state of the model.

Thus, we are assuming the existence of two *chiral edge modes,* localized on the two sides of the cylinder. These edge modes correspond to (generalized) eigenfunctions for the original Hamiltonian *H*, of the form $e^{i k_{1} x_{1}} \xi ^{\omega }(k_{1}; x_{2})$. An example of model that fulfills the existence of such edge modes is the *Haldane model*, a celebrated example of topological insulator [30, 49]. Being delocalized in the $x_{1}$ direction and localized in proximity of one of the two boundaries, the edge modes support metallic currents, that is they allow for a nontrivial charge transport on the boundary. Of course, these edge modes are no longer eigenstates of the model after the quasi-periodic perturbation is turned on. One of the goals of the present paper is to probe edge currents in the presence of weak quasi-periodic disorder, after exposing the system to a suitable class of time-dependent perturbations.

More generally, our method will allow us to characterize all equilibrium correlation functions of the disordered system. We will be interested in the time-ordered, Euclidean correlation functions, defined as follows. Let:

2.21 $$\begin{aligned} \gamma _{t}(\mathcal {O}):= e^{t (\mathcal {H} - \mu \mathcal {N})} \mathcal {O} \,e^{-t (\mathcal {H} - \mu \mathcal {N})}. \end{aligned}$$

Observe that if $\mathcal {O}$ commutes with the number operator $\mathcal {N}$, $\gamma _{t}(\mathcal {O}) = \tau _{-it}(\mathcal {O})$, that is $\gamma _{t}(\mathcal {O})$ describes the imaginary time evolution of $\mathcal {O}$. The fermionic time-ordering $\textbf{T}$ acts on fermionic monomials, as follows. Let $t_{i}\in [0,\beta )$, for i=1,…,n. Then, for $t_{i} \ne t_{j}$, if i≠j:

2.22 $$\begin{aligned} \textbf{T}\big (\gamma _{t_{1}}(a^{\sharp _{1}}_{x_{1},\sigma _{1}}) \cdots \gamma _{t_{n}}(a^{\sharp _{n}}_{x_{n},\sigma _{n}}) \big )= \text {sgn}(\pi ) \gamma _{t_{\pi (1)}}(a^{\sharp _{\pi (1)}}_{x_{\pi (1)},\sigma _{\pi (1)}}) \cdots \gamma _{t_{\pi (n)}}(a^{\sharp _{\pi (n)}}_{x_{\pi (n)},\sigma _{\pi (n)}}), \nonumber \\ \end{aligned}$$

where $a^{\sharp }$ can be either *a* or $a^{*}$, and the permutation π is such that $t_{\pi (1)}> t_{\pi (2)}> \cdots > t_{\pi (n)}$. The definition (2.22) is extended to coinciding times by normal ordering, that is by placing creation operators to the left of annihilation operators. Finally, the definition (2.22) is extended by linearity to the whole (finite dimensional) algebra of operators on $\mathcal {F}_{L}$. We shall be interested in the expectation value of time-ordered monomials, the simplest example being the two-point correlation function. Let us introduce the notations:

2.23 $$\begin{aligned} {\boldsymbol{x}}:= (x_{0}, x_{1}),\qquad \vec {\boldsymbol{x}}:= (x_{0}, \vec {x}). \end{aligned}$$

Let $0\le x_{0}, y_{0} < \beta$. We define the Euclidean two-point correlation function as:

2.24 $$\begin{aligned} S^{\beta , L}_{2; \sigma , \zeta }(\vec {\boldsymbol{x}}, \vec {\boldsymbol{y}}):= \langle \textbf{T}\gamma _{x_{0}}(a_{\vec x, \sigma }) \gamma _{y_{0}}(a^{*}_{\vec y,\zeta } ) \rangle _{\beta , \mu , L}. \end{aligned}$$

The two-point function is then extended to all values of $x_{0}, y_{0}$ in $\mathbb {R}$, by antiperiodicity:

2.25 $$\begin{aligned} S^{\beta , L}_{2; \sigma , \zeta }\big ((x_{0} + n\beta , \vec x), (y_{0} + m\beta , \vec y)\big ):= (-1)^{n+m} \langle \textbf{T}\gamma _{x_{0}}(a_{\vec x, \sigma }) \gamma _{y_{0}}(a^{*}_{\vec y,\zeta } ) \rangle _{\beta , \mu , L}.\nonumber \\ \end{aligned}$$

Being the Hamiltonian (2.4) quadratic in the fermionic operators, all Euclidean correlation functions can be computed starting from the two-point function (2.24), via the fermionic Wick rule. For λ=0, the two-point function is space-time translation invariant, and it can be expressed in terms of the resolvent of ${\hat{H}}(k_{1})$. Let:

2.26 $$\begin{aligned} \mathscr {D}_{L,\beta }:= \Big \{ {\boldsymbol{k}} = (k_{0}, k_{1})\, \Big |\, k_{0} \in \frac{2\pi }{\beta } \Big (\mathbb {Z} + \frac{1}{2}\Big ),\; k_{1} \in \mathscr {D}_{L} \Big \}. \end{aligned}$$

Let $${\boldsymbol{k}} \in \mathscr {D}_{L,\beta }$$. Define:

2.27 $$\begin{aligned} G_{\sigma ,\zeta }({\boldsymbol{k}}, x_{2}, y_{2}):= \int _{0}^{\beta } dx_{0}\, e^{-ik_{0}x_{0}} \sum _{x_{1}} e^{-ik_{1} x_{1}} S^{\beta , L}_{2; \sigma , \zeta }(\vec {\boldsymbol{x}}, \vec {\boldsymbol{y}})\Big |_{\lambda = 0}. \end{aligned}$$

Then, it is well-known that:

2.28 $$\begin{aligned} G_{\sigma ,\zeta }({\boldsymbol{k}}, x_{2}, y_{2}) = \Big (\frac{1}{ik_{0} + {\hat{H}}(k_{1}) - \mu }\Big )_{\sigma ,\zeta }(x_{2}, y_{2}). \end{aligned}$$

The numbers $\{k_{0}\}$ are called fermionic Matsubara frequencies, and are the frequencies associated with the anti-periodicity in time of the two-point function. Thus, the function $G({\boldsymbol{k}})$ allows one to reconstruct the two-point function by taking the inverse Fourier transform (away from discontinuity points). Observe that item *(iv)* in Assumption 3 guarantees the existence of the infinite volume limit of the two-point function for λ=0.

For later use, we shall introduce the following distance, needed to quantify the decay properties of the two-point function:

2.29 $$\begin{aligned} \Vert \vec {\boldsymbol{x}} - \vec {\boldsymbol{y}} \Vert _{\beta , L}:= \min _{n\in \mathbb {Z}} | x_{0} - y_{0} + n\beta | + \Vert \vec x - \vec y \Vert _{L}, \end{aligned}$$

with $\Vert \vec x - \vec y \Vert _{L}$ as in (2.3). We shall also set:

2.30 $$\begin{aligned} \Vert {\boldsymbol{x}} - {\boldsymbol{y}} \Vert _{\beta , L}:= \min _{n\in \mathbb {Z}} | x_{0} - y_{0} + n\beta | + \min _{m\in \mathbb {Z}} | x_{1} - y_{1} + mL|. \end{aligned}$$

Finally, we will use the short-hand notation $\Vert \vec {\boldsymbol{x}} - \vec {\boldsymbol{y}} \Vert \equiv \Vert \vec {\boldsymbol{x}} - \vec {\boldsymbol{y}} \Vert _{\beta , L}$, $\Vert {\boldsymbol{x}} - {\boldsymbol{y}} \Vert \equiv \Vert {\boldsymbol{x}} - {\boldsymbol{y}} \Vert _{\beta , L}$.

#### Remark 2.4

We anticipate that the reason why we introduce the imaginary-time evolution of the fermionic operators is that we will be able to rewrite real-time transport coefficients in terms of imaginary-time correlation functions. In contrast to real-time correlation functions, the imaginary-time correlation functions admit much better decay estimates. This will open the way to a rigorous computation of the transport coefficients in presence of disorder, discussed in Sect. 5 of this work.

### Transport

Let us consider the time-dependent Hamiltonian, for t≤0 and $\eta , \theta > 0$:

2.31 $$\begin{aligned} \mathcal {H}(\eta t) = \mathcal {H} - e^{\eta t} \mathcal {P},\qquad \mathcal {P} = \sum _{{\vec x} \in \Lambda _{L}} \mu (\theta \vec x) n_{{\vec x}}, \end{aligned}$$

where $n_{{\vec x}} = \sum _{\sigma }a^{*}_{{\vec x,\sigma }} a_{{\vec x,\sigma }}$ is the density operator, and $\mu (\theta \vec x)$ is of the form:

2.32 $$\begin{aligned} \mu (\theta \vec x) = \sum _{n\in \mathbb {Z}} \mu _{\infty }\big (\theta (x_{1} + nL_{1}), \theta x_{2}\big ). \end{aligned}$$

We will suppose that $\mu _{\infty }(\vec x)$ is a smooth function $\mathbb {R}^{2}$, that decays faster than any power:

2.33 $$\begin{aligned} | \partial _{x_{0}}^{n_{0}} \partial _{x_{1}}^{n_{1}}\mu _{\infty }(\vec x) | \le \frac{C_{r, n_{0},n_{1}}}{1+|\vec x|^{r}}\qquad \text {for all } n_{0}, n_{1}, r\in \mathbb {N}. \end{aligned}$$

In particular, this implies decay of $\mu (\theta \vec x)$:

2.34 $$\begin{aligned} |\mu (\theta \vec x)| \le \frac{C_{r}}{1 + \theta ^{r} | x_{1} |^{r}_{L_{1}}} \frac{1}{1 + \theta ^{r}|x_{2}|^{r}}\qquad \text {with } | x_{1} |_{L_{1}} = \min _{n\in \mathbb {Z}} | x_{1} + n L_{1} |, \text { for all } r\in \mathbb {N}. \nonumber \\ \end{aligned}$$

The dynamics of the equilibrium state of the system generated by (2.31) is:

2.35 $$\begin{aligned} i\partial _{t} \rho (t) = [ \mathcal {H}(\eta t), \rho (t) ],\qquad \rho (-\infty ) = \rho _{\beta , \mu , L}, \end{aligned}$$

where $\rho _{\beta , \mu , L}$ is the Gibbs state of the Hamiltonian $\mathcal {H}$. We shall be interested in the linear response of the density and of the current operators, defined as follows. Consider the lattice continuity equation:

2.36 $$\begin{aligned} \partial _{t} \tau _{t}(n_{\vec x}) + \sum _{i=1,2} \text {d}_{x_{i}} \tau _{t}( j_{i, \vec x} ) = 0, \end{aligned}$$

with $\tau _{t}$ the Heisenberg evolution generated by $\mathcal {H}$, and $\text {d}_{x_{i}}$ the discrete derivative, $\text {d}_{x_i} f(\vec y) = f(\vec y) - f(\vec y-\vec e_i)$. The operator $j_{i,\vec x}$ is called the current density operator, and it can be explicitly determined. We compute:

2.37 $$\begin{aligned} \partial _t \tau _t ( n_{\vec x}) =i \tau _{t}\big (\big [\mathcal {H}, n_{\vec x}\big ]\big ) = \sum _{{\vec y\in \Lambda _{L} }} \tau _{t}(j_{\vec y, \vec x}), \end{aligned}$$

where the bond current $j_{\vec y, \vec x}$ is:

2.38 $$\begin{aligned} j_{\vec y, \vec x} = i\sum _{\sigma ,\zeta } \big ( H_{\zeta ,\sigma }(\vec y,\vec x) a^*_{\vec y,\zeta } a_{\vec x,\sigma }-H_{\sigma ,\zeta }(\vec x,\vec y) a^*_{\vec x,\sigma } a_{\vec y,\zeta }\big ). \end{aligned}$$

Observe that the bond current is antisymmetric, $j_{\vec y, \vec x} = - j_{\vec x, \vec y}$. In terms of these operators, we can express the current density $j_{i,\vec x}$ as, for j≠i:

2.39 $$\begin{aligned} j_{i,\vec x}= j_{\vec x,\vec x+\vec e_i}+\frac{1}{2}\big ( j_{\vec x,\vec x+\vec e_i+\vec e_j}+ j_{\vec x,\vec x+\vec e_i-\vec e_j}+ j_{\vec x+\vec e_j,\vec x+\vec e_i}+ j_{\vec x-\vec e_j,\vec x+\vec e_i}\big ). \end{aligned}$$

It is convenient to collect current and densities into a single 3-current; to this end, we define $j_{0,\vec x}:= n_{\vec x}$, and we shall collect density and currents into a single vector with components $j_{\nu ,\vec x}$, ν=0,1,2. Also, we shall set:

2.40 $$\begin{aligned} j_{\nu ,x_1}:= \sum _{x_2 = 1}^{L_{2}} j_{\nu ,\vec x}. \end{aligned}$$

In the following, we shall consider smeared version of the current densities, against suitable test functions localized in proximity of the $x_{2} = 0$ boundary of the cylinder.

Let ℓ>1, let $\phi _{\infty }(\vec x)$ be a smooth, compactly supported function on $\mathbb {R}^{2}$, and let $\phi _{\infty ,\ell }(\vec x) = \phi _{\infty }(x_{1}, x_{2}/\ell )$. We denote by $\phi _{\ell }(\theta x_{1}, x_{2})$ the $x_{1}$-periodization of $\phi _{\infty ,\ell } (\theta x_{1}, x_{2})$, as in (2.32), and we set $\phi _{\theta ,\ell }(\vec x) = \theta \phi _{\ell } (\theta x_{1}, x_{2})$. We define:

2.41 $$\begin{aligned} j_{\nu }(\phi _{\theta ,\ell }):= \theta \sum _{\vec x\in \Lambda _{L}} \phi _{\ell }(\theta x_{1}, x_{2})\,j_{\nu ,\vec x}. \end{aligned}$$

Observe that the normalization of the test function implies that the operator $j_{\nu }(\phi _{\theta ,\ell })$ is bounded uniformly in θ (but not in ℓ). The parameter ℓ will be chosen independently of θ and η, and it defines the width of the strip where we will measure the edge current. We will be interested in the order of limits: $L_{2}, L_{1} \rightarrow \infty$; then $\beta \rightarrow \infty$; then $\eta ,\theta \rightarrow 0$ (different relative orders of $\eta \rightarrow 0$ and $\theta \rightarrow 0$ will give different results); and finally $\ell \rightarrow \infty$.

We shall be interested in the linear response of $j_{\nu }(\phi _{\theta ,\ell })$:

2.42 $$\begin{aligned} {{\,\textrm{Tr}\,}}\big (j_{\nu }(\phi _{\theta ,\ell }) \rho (0) \big )- {{\,\textrm{Tr}\,}}\big (j_{\nu }(\phi _{\theta ,\ell }) \rho _{\beta , \mu , L} \big )= \chi ^{\beta ,L}_{\nu ;\ell }(\eta , \theta ) + \text {h.o.t.}, \end{aligned}$$

where “$\text {h.o.t.}$” denotes higher order terms, and where $\chi ^{\beta ,L}_{\nu ;\ell }(\eta , \theta )$ is given by Kubo formula:

2.43 $$\begin{aligned} \chi ^{\beta ,L}_{\nu ;\ell }(\eta , \theta ) = i\int _{-\infty }^{0} d t\, e^{\eta t} {{\,\textrm{Tr}\,}}\big [ j_{\nu }(\phi _{\theta ,\ell }), \tau _{t}( \mathcal {P} ) \big ] \rho _{\beta , \mu , L}. \end{aligned}$$

Eq. (2.42) is nothing but the Duhamel expansion of the non-autonomous dynamics generated by (2.31), at first order in the perturbation. The higher order terms collect the higher order commutators produced in this expansion. In this work, we will not be concerned with proving the validity of linear response (2.42) in the zero temperature and infinite volume limit. Instead, we will start from Kubo formula (2.43), and study rigorously this transport coefficient. We shall be interested in computing the zero temperature and infinite volume limit of this quantity,

2.44 $$\begin{aligned} \chi _{\nu ;\ell }(\eta , \theta ) = \lim _{\beta \rightarrow \infty } \lim _{L_{2}\rightarrow \infty } \lim _{L_{1}\rightarrow \infty } \chi ^{\beta ,L}_{\nu ;\ell }(\eta , \theta ). \end{aligned}$$

Observe that already proving that the time-integral in (2.43) is finite uniformly in η is non-trivial, in view of the lack of spectral gap at the Fermi level. Furthermore, the slow decay of correlations, also implied by the lack of spectral gap, gives rise to a non-trivial infrared problem in the zero temperature and infinite volume limit. Our main result will not only prove that these limits exist and are finite, but will also provide an explicit expression for $\chi _{\nu ;\ell }(\eta , \theta )$, for small quasi-periodic perturbation.

## Results

### Boundary Correlations and Transport Coefficients

Let us denote by $\chi (\cdot )$ a smooth cutoff function: $\chi (x) \in C^{\infty }(\mathbb {R})$ such that $\chi (x) = \chi (-x)$ and, for δ>0:

3.1 $$\begin{aligned} \chi (x) = 1\quad \text {for } |x| < \delta /2, \qquad \chi (x) = 0\quad \text {for } |x|>\delta . \end{aligned}$$

The parameter δ is chosen as in Assumption 3. Also, let us define:

3.2 $$\begin{aligned} |x_{2}|_{+}:= x_{2},\qquad |x_{2}|_{-}:= L_{2} - x_{2}. \end{aligned}$$

In what follows, we shall denote by ${\bar{c}}$ the complex conjugate of the number $c\in \mathbb {C}$. We are now ready to state our first result.

#### Theorem 3.1

(Two-point function). Suppose that H,μ satisfy Assumption 3. For any κ>0 there exists $\lambda _{0}, \beta _0 >0$ such that for $|\lambda | < \lambda _{0}$ the following holds. For $\beta _0\le \beta \le \kappa L_{1}$, $\beta \le \kappa L_{2}$ the two-point function can be written as:

3.3 $$\begin{aligned} S^{\beta ,L}_{2; \sigma , \zeta }(\vec {\boldsymbol{x}}; \vec {\boldsymbol{y}}) = \sum _{\omega = \pm } e^{i k_{F,L}^{\omega }(\lambda )(x_{1} - y_{1})} Z_{\omega ,\sigma }(\vec x) \check{g}_{\omega ;\textrm{s}}({\boldsymbol{x}} - {\boldsymbol{y}}) \overline{Z_{\omega ,\zeta }(\vec y)} + R_{\sigma ,\zeta }(\vec {\boldsymbol{x}}; \vec {\boldsymbol{y}}), \nonumber \\ \end{aligned}$$

where:

3.4 $$\begin{aligned} \check{g}_{\omega ;\textrm{s}}({\boldsymbol{x}} - {\boldsymbol{y}}) = \frac{1}{\beta L_{1}} \sum _{{\boldsymbol{q}} \in \mathscr {D}_{L,\beta }} e^{i {\boldsymbol{q}}\cdot ( {\boldsymbol{x}} - {\boldsymbol{y}})} \frac{\chi ({\boldsymbol{q}})}{i v_{0,\omega }(\lambda ) q_{0} + v_{1,\omega }(\lambda ) q_{1}} \end{aligned}$$

with $k^{\omega }_{F,L}(\lambda )$ an approximant in $\mathscr {D}_{L}$ of a smooth function $k^{\omega }_{F}(\lambda )$ of λ such that $k^{\omega }_{F}(0) = k_{F}^{\omega }$. The parameters $v_{0}(\lambda ), v_{1}(\lambda )$ are smooth in λ and satisfy:

3.5 $$\begin{aligned} v_{0,\omega }(0) = 1,\qquad v_{1,\omega }(0) = v_{\omega }; \end{aligned}$$

the functions $Z_{\omega ,\sigma }(\vec x)$ are smooth in λ and have the form:

3.6 $$\begin{aligned} Z_{\omega ,\sigma }(\vec x) = \sum _{n \in \mathbb {N}} Z_{n,\omega ,\sigma }(x_{2}) e^{-i n \alpha x_{1}}. \end{aligned}$$

The Fourier coefficients satisfy:

3.7 $$\begin{aligned} |Z_{n,\omega ,\sigma }(x_{2})| \le C |\lambda |^{1-\delta _{n,0}} e^{-c |n|} e^{-c |x_{2}|_{\omega }},\qquad Z_{0,\omega ,\sigma }(\vec x)\Big |_{\lambda = 0} = \xi ^{\omega }_{\sigma }(k^{+}_{F}; x_{2}). \end{aligned}$$

Also, the velocities $v_{0,\omega }(\lambda )$, $v_{1,\omega }(\lambda )$ satisfy the identities:

3.8 $$\begin{aligned} \begin{aligned} v_{0,\omega }(\lambda )&= \sum _{n,\sigma ,x_{2}} Z_{n,\omega ,\sigma }(x_{2}) \overline{Z_{n,\omega ,\sigma }(x_{2})} \\ v_{1,\omega }(\lambda )&= \sum _{\begin{array}{c} n,\sigma ,\zeta \\ x_{2}, y_{2} \end{array}} \overline{Z_{n,\omega ,\sigma }(x_{2})} \Big ( \partial _{k_{1}} {\hat{H}}_{\sigma ,\zeta }\big (k_{F,L}^{\omega }(\lambda ) - n \alpha ; x_{2}, y_{2}\big ) \Big ) {Z_{n,\omega ,\zeta }(y_{2})}. \end{aligned} \end{aligned}$$

Finally, the error term in (3.3) satisfies:

3.9 $$\begin{aligned} \begin{aligned} R_{\sigma , \zeta }(\vec {\boldsymbol{x}}; \vec {\boldsymbol{y}})&= \sum _{n\in \mathbb {Z}} R_{n;\sigma , \zeta }({\boldsymbol{x}} - {\boldsymbol{y}}; x_{2}, y_{2}) e^{-in \alpha y_{1}} \\ | R_{n;\sigma , \zeta }({\boldsymbol{x}} - {\boldsymbol{y}}; x_{2}, y_{2}) |&\le C |\lambda |^{\delta _{n\ne 0}}e^{-c|n|} \frac{e^{-c| x_{2} -y_{2} |}}{1 + \Vert {\boldsymbol{x}} - {\boldsymbol{y}} \Vert ^{1 + \xi }}\quad \text {for some } \xi >0. \end{aligned} \end{aligned}$$

#### Remark 3.2

(i) By Wick’s rule, Theorem 3.1 allows us to fully characterize the large scale behavior of all the edge correlation functions of the system. A similar analysis has been performed in [23] for the quasi-periodic Ising model, which however only considered the energy-energy correlation function (it is related to a density-density correlation function in the fermionic representation).
(ii) The expressions (3.3), (3.4) show that the boundary correlations agree with those of a relativistic effective model, up to the presence of oscillatory and non-universal amplitudes $Z_{\omega }(\vec x)$, that encode the breaking of translation-invariance at the macroscopic scale. This is the “oscillatory” nature of the emergent QFT describing the disordered edge modes, mentioned in the introduction.
(iii) All λ-dependent quantities in the above theorem are still (weakly) dependent on $\beta , L_{1}, L_{2}$. The method of the proof also allows us to show that they converge to a limit as $\beta , L_{1}, L_{2} \rightarrow \infty$, where the $L_{1}\rightarrow \infty$ limit is always understood as taken over sequences specified in Remark 2.1 item (ii).

Our next result allows us to compute the zero temperature and infinite volume edge transport coefficients, defined as (recall Eq. (2.44)):

3.10 $$\begin{aligned} G_{0}:= \lim _{\ell \rightarrow \infty }\lim _{\theta \rightarrow 0} \lim _{\eta \rightarrow 0^{+}} \frac{\chi _{0;\ell }(\eta , \theta )}{\langle \mu ,\phi \rangle _{\text {edge}}},\qquad G_{1}:= \lim _{\ell \rightarrow \infty }\lim _{\theta \rightarrow 0} \lim _{\eta \rightarrow 0^{+}} \frac{\chi _{1;\ell }(\eta ,\theta )}{\langle \mu ,\phi \rangle _{\text {edge}}}, \end{aligned}$$

where:

3.11 $$\begin{aligned} \langle \mu ,\phi \rangle _{\text {edge}}:= \int _{\mathbb {R}} dx\, \overline{\mu _{\infty }(x,0)} \phi _{\infty }(x,0). \end{aligned}$$

The quantity $G_{0}$ is the edge susceptibility, while the quantity $G_{1}$ is the edge conductance. The normalization in (3.10) is natural, in view of the arbitrariness of the shape of the perturbation and of the test function in the observable.

#### Theorem 3.3

(Transport coefficients). Under the same assumptions of Theorem 3.1 the following is true. Let $v^{\infty }_{i,+}(\lambda ) = \lim _{\beta \rightarrow \infty } \lim _{L_{2}, L_{1}\rightarrow \infty } v_{i,+}(\lambda )$ with $v_{i,+}(\lambda )$ as in Theorem 3.1. Let $\mathfrak {v}(\lambda ):= v^{\infty }_{1,+}(\lambda ) / v^{\infty }_{0,+}(\lambda )$. Then, the edge transport coefficients are given by:

3.12 $$\begin{aligned} G_{0} = \frac{1}{2\pi |\mathfrak {v}(\lambda )|},\qquad G_{1} = \frac{\text {sgn}(\mathfrak {v}(\lambda ))}{2\pi }. \end{aligned}$$

#### Remark 3.4

(i) Theorem 3.3 proves, in particular, the quantization of the edge conductance for the model in presence of weak disorder, starting from Kubo formula. The integer is the same as for the bulk Hall conductivity, in agreement with the bulk-edge duality.
(ii) The order of limits in the definitions (3.10) is important. As the proof will show, taking the η, θ limits in the reverse order would give a vanishing result.
(iii) The proof Theorem 3.3 relies in a crucial way on the construction of the two-point function, obtained in Theorem 3.1. In particular, a key role in the computation the of the limiting transport coefficients in (3.10) is played by the asymptotic behavior of the two-point function, Eq. (3.3), and by the relations (3.8). These last identities are a consequence of lattice conservation laws, and imply key cancellations in the computation of the transport coefficients, from which the expressions (3.12) follow.

### Elements of the Proofs

Theorem 3.1 is based on an extension of the multiscale analysis for quasi-periodic systems, see e.g. [8, 23, 45–47], here applied to edge modes of topological insulators for the first time. Our main new contribution is the determination of the asymptotic behavior of the two-point function. The previous works obtained estimates for the two-point function in momentum space, actually only for the average of the product of fermionic operators with equal momenta (“translation-invariant contribution”); in the present paper, instead, we control the average of fermionic bilinears involving fermionic operators with momenta shifted by any multiple of the Diophantine frequency, which in particular allows one to determine the asymptotic behavior of the two-point function in configuration space.

A similar result has been obtained in [23], for the energy-energy correlations of the quasi-periodic Ising model, where the energy operator corresponds to a quadratic fermionic observable. Here, the construction of the fermionic two-point function allows us to determine all boundary correlation functions. The key ingredient is the multiscale decomposition of the two-point function, where all contributions are related by a suitable recursion relation. Theorem 3.1 follows from a careful analysis of this recursion relation, and from the asymptotic behavior of its solution at small scales.

Let us give an outline of the proof of Theorem 3.3. The starting point is the rewriting of the response function (2.43) in imaginary times (Wick’s rotation):

3.13 $$\begin{aligned} \chi ^{\beta ,L}_{\nu ;\ell }(\eta , \theta )= &   \theta \sum _{\vec x,\vec y } \mu (\theta \vec x) \phi _{\ell }(\theta y_{1}, y_{2}) \int _{-\beta /2}^{\beta /2} d s\, e^{-i\eta _{\beta } s} \nonumber \\  &   \times \langle \textbf{T}\gamma _{s}(n_{\vec x}); j_{\nu ,\vec y} \rangle _{\beta ,\mu ,L} + O(1 / (\beta \eta ^{3})). \end{aligned}$$

This rewriting has been used in several places, see e.g. [3, 25, 27, 29, 48]. The advantage is that we got rid of real-time correlation functions, for which no effective decay estimates can be proved, in favor of imaginary-time correlations, which can be studied in a more efficient way. Being the model quasi-free, the right-hand side can actually be expressed in terms of the Euclidean two-point function (2.24). Still, the presence of disorder makes a direct computation essentially impossible.

The main idea, which is an extension to quasi-periodic systems of the method used in [3, 48], is to isolate the “scaling limit” contribution in the integrand in (3.13):

3.14 $$\begin{aligned} \chi ^{\beta ,L}_{\nu ;\ell }(\eta , \theta ) = \chi ^{\text {sing}}_{\nu ;\ell }(\eta , \theta ) + \chi ^{\text {reg}}_{\nu ;\ell }(\eta , \theta ), \end{aligned}$$

where: $\chi ^{\text {sing}}_{\nu ;\ell }$, the singular part, is obtained by replacing the two-point function by its leading oscillatory contribution in (3.3); while $\chi ^{\text {reg}}_{\nu ;\ell }(\eta , \theta )$, the regular part, is expressed in terms of quantities that have improved decay estimates. Crucially, lattice conservation laws allow us to express the regular part in terms of the singular part, via *Ward identities* for Euclidean correlations. As discussed in Sect. 5.2, the regularity estimate

3.15 $$\begin{aligned} \big |\chi ^{\text {reg}}_{\nu ;\ell }(\eta ,\theta ) - \chi ^{\text {reg}}_{\nu ;\ell }(\eta ,\theta ')\big |\le C_{\ell } (|\theta |^{\alpha } + |\theta '|^{\alpha }) \end{aligned}$$

combined with the lattice continuity equation (2.36) imply:

3.16 $$\begin{aligned} \chi ^{\text {reg}}_{\nu ;\ell }(\eta , \theta ) = - \chi ^{\text {sing}}_{\nu ;\ell }(\eta , \eta ^{2}) + O_{\ell }(|\theta |^{\alpha } + |\eta |^{\alpha }). \end{aligned}$$

The regularity estimate (3.15) is a consequence of Lemma 5.5, and we will not discuss it here. Let us now focus on the singular part. For simplicity, we will outline the computation of the susceptibility, ν=0; the analysis for the conductance, ν=1, proceeds along the same lines. Let us summarize the analysis of Sect. 5.4.1. Thanks to the asymptotics (3.3), the response function can be rewritten as, in Fourier space:

3.17 $$\begin{aligned} \begin{aligned} \chi ^{\text {sing}}_{0;\ell }(\eta , \theta )&= - \frac{\theta }{L_{1}} \sum _{p} \sum _{n,m} \sum _{x_{2},y_{2}} (Z_{+}\star \overline{Z_{+}})(m;y_{2}) (Z_{+}\star \overline{Z_{+}})(n;x_{2})\\&\qquad \cdot {\hat{\mu }}_{\theta }(-p-n\alpha ,x_2) {\hat{\phi }}_{\theta ,\ell }( p-m\alpha ,y_{2}) \mathcal {B}_{+}^{\infty }(\eta , p) + \textrm{o}(1). \end{aligned} \end{aligned}$$

Here and below, the $\textrm{o}(1)$ error terms vanish in the order of limits: first $L_{1,2}\rightarrow \infty$ (irrespective of the order); then $\beta \rightarrow \infty$; then $\eta ,\theta \rightarrow 0$ (irrespective of the order); then $\ell \rightarrow \infty$. The function $\mathcal {B}_{+}^{\infty }(\eta , p)$ is the so-called anomalous bubble diagram, computed for a free relativistic 1+1 dimensional model with propagator (3.4) with ω=+,

3.18 $$\begin{aligned} \mathcal {B}_{+}^{\infty }(\eta , p) = \frac{1}{4\pi \big |v_{1,+}(\lambda ) v_{0,+}(\lambda ) \big |}\frac{iv_{0,+}(\lambda )\eta -v_{1,+}(\lambda ) p}{i v_{0,+}(\lambda )\eta +v_{1,+}(\lambda ) p} + r_{+}(\eta , p), \end{aligned}$$

where $v_{\mu ,+}(\lambda )$ are as in (3.4) and $r_{+}(\eta , p)$ vanishes continuously as $(\eta , p) \rightarrow (0,0)$; while

3.19 $$\begin{aligned} (Z_{+}\star \overline{Z_{+}})(n;x_{2}) = \sum _{\sigma } \sum _{m\in {\mathbb {Z}}}Z_{m,+,\sigma }(x_2) \overline{Z_{m-n,+,\sigma }(x_2)}, \end{aligned}$$

with $Z_{m,+,\sigma }(x_2)$ the amplitudes of the oscillatory functions appearing in the leading term (3.3), see also (3.6). Our goal at this point is to use (3.14), (3.16), (3.17) to show that, approximating the sum by an integral and performing a change of variable in *p*:

3.20 $$\begin{aligned} \begin{aligned} \chi ^{\beta ,L}_{0;\ell }(\eta , \theta )&= - \int \frac{dp}{(2\pi )} \sum _{x_{2},y_{2}} (Z_{+}\star \overline{Z_{+}})(0;y_{2}) (Z_{+}\star \overline{Z_{+}})(0;x_{2})\\&\qquad \cdot {\hat{\mu }}_{\infty }(-p,x_2) {\hat{\phi }}_{\infty ,\ell }( p,y_{2}) \Big ( \mathcal {B}_{+}^{\infty }(\eta , \theta p) - \mathcal {B}_{+}^{\infty }(\eta , \eta ^{2} p)\Big ) + \textrm{o}(1). \end{aligned} \end{aligned}$$

This resembles a response function computed in a translation-invariant setting, *but* with renormalized parameters, due to homogeneization effects on a macroscopic scale. For instance, the test functions are “dressed” by $(Z_{+}\star \overline{Z_{+}})(0;x_{2})$, $(Z_{+}\star \overline{Z_{+}})(0;y_{2})$, which are disorder-dependent quantities. Let us postpone for a moment the analysis of the leading term in (3.20), and let us briefly discuss why (3.20) holds.

The subtraction of $- \mathcal {B}_{+}^{\infty }(\eta , \eta ^{2} p)$ takes into account the presence of the regular part, thanks to (3.16), up to subleading terms in η and θ. Then, we observe that the smoothness of the test functions in configuration space morally impose, in (3.17), that $|p + n \alpha |_{\mathbb {T}} \lesssim \theta$ and that $| p - m \alpha |_{\mathbb {T}} \lesssim \theta$. If n≠-m, this implies that $| (n + m)\alpha |_{\mathbb {T}} \lesssim \theta$; and by the Diophantine condition (2.9), since m+n≠0, this means that $|n + m| \gtrsim \theta ^{-\frac{1}{\tau }}$. Thus, the corresponding contribution to the transport coefficient is overwhelmingly small in θ, thanks to the exponential decay of the amplitudes $Z_{k,+,\sigma }(z_2)$ appearing in the expression for the two-point function and hence in (3.20), recall (3.6), (3.7).

Consider now the contribution of the terms n=-m≠0 in (3.17). After a change of variables, we are left with:

3.21 $$\begin{aligned}  &   - \frac{\theta }{L_{1}} \sum _{p} \sum _{0\ne n} \sum _{x_{2},y_{2}} (Z_{+}\star \overline{Z_{+}})(-n;y_{2}) (Z_{+}\star \overline{Z_{+}})(n;x_{2}) \nonumber \\  &   \quad \cdot {\hat{\mu }}_{\theta } (-p,x_2) \hat{\phi }_{\theta ,\ell }( p,y_{2}) \mathcal {B}_{+}^{\infty }(\eta , p - n\alpha ). \end{aligned}$$

Here we rely again on the Diophantine condition. The properties of the test functions imply that $|p|_{\mathbb {T}} \lesssim \theta$; we shall distinguish two regimes, $|n\alpha |_{\mathbb {T}} \ge \sqrt{\theta }$ and $|n\alpha |_{\mathbb {T}} < \sqrt{\theta }$. In the first regime, and for *p* in the range of the test functions, the function $\mathcal {B}_{+}^{\infty }(\eta , p - n\alpha )$ is actually *continuous* at (η,p)=(0,0). This ultimately implies that this regime contributes to (3.17) with a term that satisfies the bound (3.15). Thus, the corresponding contribution can be reabsorbed in a redefinition of $\chi ^{\text {reg}}_{0;\ell }(\eta , \theta )$. Concerning the second regime, since n≠0 we can use again the Diophantine condition, to prove that the corresponding contribution is overwhelmingly small in θ.

Therefore, we are left with (3.20). Up to subleading terms,

3.22 $$\begin{aligned} \begin{aligned} \chi ^{\beta ,L}_{0;\ell }(\eta , \theta )&= - \int \frac{d p}{2\pi } \sum _{x_{2},y_{2}} (Z_{+}\star \overline{Z_{+}})(0;y_{2}) (Z_{+}\star \overline{Z_{+}})(0;x_{2}) \\&\qquad \cdot {\hat{\mu }}_{\infty }(- p, 0) {\hat{\phi }}_{\infty }( p,0) \big ( \mathcal {B}_{+}^{\infty }(\eta , \theta p) - \mathcal {B}_{+}^{\infty }(\eta , 0)\big ) + \textrm{o}(1). \end{aligned} \end{aligned}$$

Calling:

3.23 $$\begin{aligned} \zeta _{0,+}:= \sum _{y_{2} = 0}^{L_{2}} (Z_{+}\star \overline{Z_{+}})(0;y_{2}), \end{aligned}$$

we then have, from the explicit expression of the anomalous bubble diagram (3.18):

3.24 $$\begin{aligned} \chi ^{\beta ,L}_{0;\ell }(\eta , \theta ) = \frac{\zeta _{0,+}^{2}}{v_{0,+}^{2}} \int \frac{dp}{(2\pi )^2} \hat{\mu }_{\infty }(- p, 0) {\hat{\phi }}_{\infty }( p,0)\frac{\text {sgn}(v_{1,+})\,\theta p}{i \eta + \mathfrak {v}(\lambda ) \theta p} + \textrm{o}(1), \end{aligned}$$

with $\mathfrak {v}(\lambda ) = v_{1}(\lambda ) / v_{0}(\lambda )$. Hence, for $\eta \ll \theta$:

3.25 $$\begin{aligned} \chi ^{\beta ,L}_{0;\ell }(\eta , \theta ) = \frac{\zeta _{0,+}^{2}}{v_{0,+}^{2}} \frac{\langle \mu , \phi \rangle _{\text {edge}}}{2\pi |\mathfrak {v}(\lambda )|} + \textrm{o}(1), \end{aligned}$$

recall the notation (3.11) for the edge scalar product. This implies our main result for the susceptibility *if* we can prove that $\zeta _{0,+}^{2} / v_{0,+}^{2} = 1$. To prove this, we rely once more on the lattice continuity equation, and in particular on the Ward identity for the so-called vertex function, discussed in Sect. 5.3. The edge conductance is computed following a similar strategy. This concludes the sketch of the proof of Theorem 3.3.

## Renormalization Group Analysis

In this section we will introduce the renormalization group analysis that allows us to prove our main results, Theorems 3.1, 3.3. We will start from the analysis of the partition function, which will allows us to introduce the notion of effective potential, and its RG flow. The section starts with the Grassmann representation of the model, and with the discussion of the integration of the ultraviolet degrees of freedom and of the massive modes. We then show in what sense the model can be reduced to an effective one-dimensional system, describing disordered edge modes. Our main technical result is Proposition 4.2, which constructs the effective potential of the model. The proof of this result relies on a suitable diagrammatic representation of the multiscale integration, in terms of decorated chain graphs, which is discussed in Sect. 4.2.4. Finally, the convergence of the iterative scheme is proved as a consequence of the control of the flow of the running coupling constants, discussed in Sect. 4.2.5.

Then, we will discuss how to adapt the construction to the case of the correlation functions, which will be computed introducing an external field in the partition function, and taking derivatives. The main result here is Proposition 4.13, contained in Sect. 4.3, which allows us to extend the construction of the effective potential of Proposition 4.2 in presence of an external Grassmann field. The proof of Theorem 3.1 is then given in Sect. 4.3.2.

### Integration of the Massive Modes

**Grassmann representation.** A convenient starting point for the renormalization group analysis is the representation of the partition function $\textsf{Z}_{\beta ,\mu ,L} \equiv \textsf{Z}$ of the disordered system, recall Eq. (2.10), in terms of a Gaussian Grassmann integral. We have, denoting by $\textsf{Z}^{0}_{\beta ,\mu ,L} \equiv \textsf{Z}^{0}$ the partition function of the non-disordered system:

4.1 $$\begin{aligned} \textsf{Z} /\textsf{Z}^{0}=\lim _{N\rightarrow +\infty } e^{-\beta \lambda \frac{S}{2} \sum _{\vec x\in \Lambda _{L}} \varphi (\vec x)} \int \mathbb {P}_{N}(d\psi ) \,\exp \big ({\mathcal V_{N}( \psi )}\big ), \end{aligned}$$

where:

(i) $\mathcal V_{N}$ is a polynomial in the Grassmann variables $\psi ^{\pm }_{{\boldsymbol{k}}, x_{2}, \sigma }$,
  4.2 $$\begin{aligned} \mathcal {V}_{N}( \psi ):= - \frac{\lambda }{\beta L_{1}}\sum _{n}\, \sum _{\boldsymbol{k}, x_2, \sigma }{\hat{\varphi }}_{n}(x_2) \psi _{\boldsymbol{k}+n{\boldsymbol{\alpha }},x_2,\sigma }^+ \psi _{\boldsymbol{k},x_2,\sigma }^- \end{aligned}$$
  defined on indices $\boldsymbol{k}=(k_0, k_1)$, with $k_{0}$ a fermionic Matsubara frequency and $k_{1}$ a spatial momentum, belonging to:
  4.3 $$\begin{aligned} \mathscr {D}_{N, L,\beta } = \Big \{ (k_{0}, k_{1}) \in \mathscr {D}_{L,\beta }\, \Big |\, k_{0} = \frac{2\pi }{\beta } \Big (n_{0} + \frac{1}{2}\Big ),\; |n_{0}| \le N \Big \}. \end{aligned}$$
  That is *N* plays the role of ultraviolet cutoff, for the Matsubara frequencies.
(ii) $\boldsymbol{\alpha }:= (0,\alpha )$ with $\alpha /2\pi = m / L_{1}$ is the best rational approximant (with denominator $L_{1}$) of the Diophantine number $\alpha _{\infty }/2\pi$, recall Assumption 2.
(iii) $\int \mathbb {P}_{N}(d\psi )$ is the Grassmann Gaussian integration with covariance:
  4.4 $$\begin{aligned} \begin{aligned} \int \hspace{-1.111pt}{\mathbb {P}}_{N}( d \psi )\psi _{\boldsymbol{k},x_2;\sigma }^-\psi ^{+}_{\boldsymbol{h},y_2;\zeta }&= \beta L_{1}\delta _{k_0,h_0}\delta _{k_1, h_1} \Big (\frac{1}{ik_{0} + {\hat{H}}(k_{1}) - \mu }\Big )_{\sigma , \zeta }(x_{2}, y_{2}) \\&\equiv \beta L_{1}\delta _{k_0,h_0}\delta _{k_1, h_1} G_{\sigma ,\zeta }(\boldsymbol{k},x_2, y_2). \end{aligned} \end{aligned}$$

The Grassmann representation of fermionic Gibbs states is well known, and widely used in rigorous studies of condensed matter systems; see e.g. [25, 55] for an introduction.^1^ The goal of this section will be to introduce an algorithm that allows us to compute (4.1) as a convergent series in λ. The difficulty in doing this is that, as Eq. (4.4) shows, the Grassmann field is singular: in the limit $\beta , L_{1} \rightarrow \infty$, the momentum-space covariance is unbounded, since by assumption μ belongs to the spectrum of the unperturbed Hamiltonian. In order to deal with this infrared problem, we shall perform a multiscale analysis, which is made possible by the addition principle of Grassmann Gaussian integrations: we split the Grassmann field into a sum of independent Grassmann fields, whose covariances are supported at a given distance from the singularity. The fields will then be integrated iteratively, and this will give rise to a flow of effective potentials. Instead, the ultraviolet limit $N\rightarrow \infty$ can be studied in a much simpler way; the only subtlety will be discussed at the end of Sect. 4.1 (finiteness of the tadpole graph).

**Symmetries.** Before starting the analysis, we spell out an important symmetry property of the theory. Let us rewrite:

4.5 $$\begin{aligned} \int \mathbb {P}_{N}(d\psi ) \,\exp \big ({\mathcal V_{N}( \psi )}\big ) = C_{N} \int D\psi \, e^{S_{N}(\psi )}, \end{aligned}$$

where $C_{N}$ is a normalization constant, Dψ is the Grassmann integration, and $S_{N}(\psi )$ takes into account the Grassmann covariance and the perturbation,

4.6 $$\begin{aligned} S_{N}(\psi ) = -\frac{1}{\beta L_{1}} \sum _{{\boldsymbol{k}}} \big ( \psi ^{+}_{{\boldsymbol{k}}}, (ik_{0} + {\hat{H}}(k_{1}) - \mu ) \psi ^{+}_{{\boldsymbol{k}}} \big ) - \mathcal {V}_{N}( \psi ). \end{aligned}$$

Then, a direct check shows that the action $S_{N}(\psi )$ is left invariant by the following transformation:

4.7 $$\begin{aligned} \psi ^{+}_{(k_{0}, k_{1}), x_{2}, \sigma } \rightarrow -\psi ^{-}_{(-k_{0}, k_{1}), x_{2}, \sigma },\qquad \psi ^{-}_{(k_{0}, k_{1}), x_{2}, \sigma } \rightarrow \psi ^{+}_{(-k_{0}, k_{1}), x_{2}, \sigma },\qquad c\rightarrow {\bar{c}}, \end{aligned}$$

where *c* is a generic constant in the action $S_{N}(\psi )$ (recall that $\overline{{\hat{\varphi }}_{n}(x_{2})} = {\hat{\varphi }}_{-n}(x_{2})$ and that ${\hat{H}}(k_{1})$ is self-adjoint). This fact will have consequences on the structure of the effective potential on smaller scales.

**Integration of the bulk modes.** To begin, we isolate the potentially divergent contribution to the covariance *G*. To this end, recall the definition of the smooth cutoff function, Eq. (3.1). Let $\chi ({\hat{H}}(k_{1}) - \mu )$ be the smoothed spectral projection, defined via functional calculus, which only depends on eigenstates of ${\hat{H}}(k_{1})$ with energies $\varepsilon (k_{1})$ in the interval $|\mu - \varepsilon (k_{1})| \le \delta$. Let us choose δ as in Assumption 3. Then, all the eigenstates of ${\hat{H}}(k_{1})$ in this energy range correspond to edge modes. We define:

4.8 $$\begin{aligned} G^{(\mathrm e)}({\boldsymbol{k}}, x_{2}, y_{2}):= \chi (k_{0}) \Big (\chi \big ({\hat{H}}(k_{1}) - \mu \big ) G(\boldsymbol{k})\Big )(x_2, y_2), \end{aligned}$$

which we can rewrite more explicitly as:

4.9 $$\begin{aligned} G^{(\mathrm e)}_{\sigma ,\zeta }({\boldsymbol{k}}, x_{2}, y_{2}) = \sum _{\omega =\pm } \chi (k_0)\chi \big (\varepsilon _\omega (k_1)-\mu \big )\frac{{\hat{\xi }}^{\omega }_{\sigma }(k_1;x_2) \overline{{\hat{\xi }}^{\omega }_{\zeta }(k_1; y_2)}}{i k_0+\varepsilon _{\omega }(k_1)-\mu }. \end{aligned}$$

The label $(\textrm{e})$ stands for “edge”: the covariance $G^{(\mathrm e)}$ is spatially supported in proximity of the two boundaries of the cylinder, by the exponential decay of ${\hat{\xi }}^{\omega }_{\sigma }(k_1;x_2)$, recall Eq. (2.19). Next, let us introduce:

4.10 $$\begin{aligned} G^{(\mathrm b)}_{\sigma ,\zeta }({\boldsymbol{k}}, x_{2}, y_{2}):= G_{\sigma ,\zeta }(\boldsymbol{k},x_2, y_2) - G^{(\mathrm e)}_{\sigma ,\zeta }({\boldsymbol{k}}, x_{2}, y_{2}). \end{aligned}$$

where $(\textrm{b})$ stands for “bulk”. By construction, this covariance is bounded uniformly in ${\boldsymbol{k}}$. Let us define the discrete derivatives:

4.11 $$\begin{aligned}  &   \text {d}_{k_{0}} f(k_{0}) = \frac{\beta }{2\pi } \big ( f(k_{0}) - f(k_{0} - 2\pi /\beta ) \big ),\nonumber \\  &   \text {d}_{k_{1}} f(k_{1}) = \frac{L_{1}}{2\pi } \big ( f(k_{1}) - f(k_{1} - 2\pi /L_{1}) \big ). \end{aligned}$$

Then, we have:

4.12 $$\begin{aligned} \Big | \text {d}_{k_{0}}^{n_{0}} \text {d}_{k_{1}}^{n_{1}} G^{(\mathrm b)}_{\sigma ,\zeta }({\boldsymbol{k}}, x_{2}, y_{2}) \Big | \le \frac{C^{\delta }_{n_{0}, n_{1}} e^{-c|x_{2} - y_{2}|}}{1 + |k_{0}|}\;, \end{aligned}$$

uniformly in ${\boldsymbol{k}}$. The bound (4.12) can be proved using the estimate for the edge modes (2.19), and a Combes-Thomas type argument; see Appendix A for details.

We now represent the Grassmann field $\psi ^{\pm }$ as:

4.13 $$\begin{aligned} \psi ^{\pm }_{{\boldsymbol{k}}, x_{2}, \sigma } = \psi ^{(\textrm{e})\pm }_{{\boldsymbol{k}}, x_{2}, \sigma } + \psi ^{(\textrm{b})\pm }_{{\boldsymbol{k}}, x_{2}, \sigma }, \end{aligned}$$

where $\psi ^{(\textrm{e})\pm }$, $\psi ^{(\textrm{b})\pm }$ have covariances given respectively by $G^{(\mathrm e)}$ and $G^{(\mathrm b)}$. Then, we write, using the addition principle of the Gaussian Grassmann integration, see e.g. [24]:

4.14 $$\begin{aligned} \begin{aligned} \frac{\textsf{Z}}{\textsf{Z}^{0}}&= \lim _{N\rightarrow +\infty } e^{-\beta \lambda \frac{S}{2} \sum _{\vec x\in \Lambda _{L}} \varphi (\vec x)}\int \mathbb {P}_{\textrm{e}}( d \psi ^{(\mathrm e)}) \int \mathbb {P}_{\textrm{b}}( d \psi ^{(\mathrm b)})\,e^{{\mathcal V_{N}( \psi ^{(\mathrm e)} + \psi ^{(\mathrm b)})}}\\&= z_{\textrm{b}} \int \mathbb {P}_{\textrm{e}}( d \psi ^{(\mathrm e)}) e^{{\mathcal V^{(\textrm{e})}( \psi ^{(\mathrm e)})}}, \end{aligned} \end{aligned}$$

where: $z_{\textrm{b}}$ is a suitable constant, to be discussed; $\mathbb {P}_{\textrm{e}}$, $\mathbb {P}_{\textrm{b}}$ are the Grassmann Gaussian integrations with covariances given by $G^{(\mathrm e)}$ and $G^{(\mathrm b)}$; and $\mathcal {V}^{(\textrm{e})}$ is the *edge effective potential.* The effective potential is defined as a sum of truncated expectations (or cumulants) of the $\psi ^{(\textrm{b})}$ field:

4.15 $$\begin{aligned} \log (z_{\textrm{b} }) + \mathcal {V}^{(\textrm{e})}(\psi ^{(\mathrm e)}) = \lim _{N\rightarrow \infty } \sum _{m\ge 1} \frac{1}{m!} \mathbb {E}^{\text {T}}_{\textrm{b}} \big (\mathcal {V}_{N}(\psi ^{(\mathrm e)} + \cdot ); m\big )\;, \end{aligned}$$

where $\mathbb {E}_{\textrm{b}}^{\text {T}}$ denotes the truncated expectation:

4.16 $$\begin{aligned} \mathbb {E}^{\text {T}}_{\textrm{b}} \big (\mathcal {V}_{N}(\psi ^{(\mathrm e)} + \cdot ); m\big ):= \frac{\partial ^{m}}{\partial t^{m}} \bigg (\log \int \mathbb {P}_{\textrm{b}}( d \psi ^{(\mathrm b)})\,\exp \big (t{\mathcal V_{N}( \psi ^{(\mathrm e)} + \psi ^{(\mathrm b)})}\big ) \bigg )\bigg |_{t=0}. \nonumber \\ \end{aligned}$$

It is well-known that the truncated expectations of Gaussian fields can be conveniently expressed in terms of tree graphs. In this case, due to the fact that the initial potential $\mathcal {V}_{N}$ is quadratic, we are left with particularly simple graphs, called chains. We have, see e.g. [24] for a review:

4.17 $$\begin{aligned} \mathcal {V}^{(\textrm{e})}(\psi ^{(\mathrm e)}) = \frac{1}{\beta L_{1}} \sum _{\begin{array}{c} n, {\boldsymbol{k}}, \\ x_{2}, y_{2}, \sigma , \zeta \end{array}} V_{n;\sigma ,\zeta }^{(\text {e})}(\boldsymbol{k},x_2,y_2)\psi _{\boldsymbol{k},x_2,\sigma }^{(\text {e})+} \psi _{\boldsymbol{k}+n\boldsymbol{\alpha },y_2,\zeta }^{(\text {e})-} , \end{aligned}$$

where $V_{n;\sigma ,\zeta }^{(\text {e})}(\boldsymbol{k},x_2,y_2)=-\lambda \hat{\varphi }_{-n}\delta _{x_2,y_2}\delta _{\sigma ,\zeta }+\mathrm O(\lambda ^2)$ is given by:

4.18 $$\begin{aligned} \begin{aligned} V_{n;\sigma ,\zeta }^{(\text {e})}(\boldsymbol{k},x_2,y_2) = \sum _{s\ge 1} \bigg (\frac{1}{s!} \sum _{\theta \in {\mathscr {T}}^{(\textrm{b})}_{s;n}} \theta (\boldsymbol{k},x_2,y_2)\bigg ), \end{aligned} \end{aligned}$$

where the sum is over chain graphs θ with values $\theta (\boldsymbol{k},x_2,y_2)$, with the following properties:

(i) the chains θ are labelled, and have nodes v=1,…,s with s≥1;
(ii) to each node we associate an integer $n_{v}$ and a position label $x_{2,v}$. Each node represents a perturbation $-\lambda {\hat{\varphi }}_{-n_{v}}(x_{2,v})$. The integers satisfy the condition $\sum _{v = 1}^{s} n_{v} = n$;
(iii) Each each node has two oriented lines attached, an incoming one and an outgoing one; the incoming one corresponds to a field $\psi ^{(\text {b})-}$, the outgoing one corresponds to a field $\psi ^{(\text {b})+}$. The node, together with the two lines, is understood as a graphical representation for one of the monomials entering in the definition of $\mathcal {V}_{N}$. The lines are decorated by the labels of the fields that they represent. The chain is formed by joining lines with opposite orientation, and with the same momentum labels. The outgoing line of the last node and the incoming line of the first node are not paired;
(iv) The contraction of an incoming line labelled by ${\boldsymbol{k}}, \sigma _{v}, x_{2,v}$ with an outgoing line labelled by ${\boldsymbol{p}}, \sigma _{v+1}, x_{2,v+1}$ corresponds to a propagator $\delta _{{\boldsymbol{k}}, {\boldsymbol{p}}}G^{(\text {b})}_{\sigma _v,\sigma _{v+1}}\big (\boldsymbol{k}, x_{2,v}, x_{2,v+1}\big )$;
(v) the value of the chain is:
  4.19 $$\begin{aligned} \theta (\boldsymbol{k},x_2,y_2):= \Big (\prod _{v=1}^s -\lambda {\hat{\varphi }}_{-n_v}(x_{2,v})\Big ) \prod _{v=1}^{s-1}G^{(\text {b})}_{\sigma _v,\sigma _{v+1}}\big (\boldsymbol{k} + n(v)\boldsymbol{\alpha }, x_{2,v}, x_{2,v+1}\big ), \nonumber \\ \end{aligned}$$
  where $$n(v) = \sum _{v'\le v} n_{v'}$$.

#### Remark 4.1

(i) That is, the value of the graph is obtained taking the products of the potentials associated with the nodes, times the propagators arising from the contractions of the lines. The momentum flowing in each propagator is obtained from the Kirchhoff law, with the rule that each node is a “source” of momentum $-n_{v} {\boldsymbol{\alpha }}$, which is transported along each propagator following the orientation of the lines. The momentum ${\boldsymbol{k}}$ is the outgoing momentum of the graph; the incoming momentum is then ${\boldsymbol{k}}$ plus the net momentum shift due to the nodes, which is set to be $n{\boldsymbol{\alpha }}$.
(ii) The sum over θ takes into account the sum over all possible contractions between nodes, and the sum over all labels. If *s* is the number of vertices, the number of possible connected graphs is *s*!, which is compensated by the factor 1/*s*! in (4.18).

Thus, in view of the bound (4.12) and of the decay of the Fourier modes of the potential (2.7), we obtain that the effective potential is analytic in λ for |λ| small enough uniformly in β,L; moreover we get the following bound, for suitable constants $C_{n_{0},n_{1}}, c>0$:

4.20 $$\begin{aligned} \Big | \text {d}_{k_{0}}^{n_{0}} \text {d}_{k_{1}}^{n_{1}} V_{n;\sigma ,\zeta }^{(\text {e})}(\boldsymbol{k},x_2,y_2) \Big |\le C_{n_{0},n_{1}} |\lambda | e^{- c |n|} e^{-c|x_2-y_2|}. \end{aligned}$$

As a consequence of the symmetry property (4.7), we have:

4.21 $$\begin{aligned} \overline{V_{n;\sigma ,\zeta }^{(\mathrm e)}((k_{0}, k_{1}),x_2,y_2)} = V_{-n;\zeta ,\sigma }^{(\mathrm e)}\big ((-k_{0}, k_{1} + n \alpha ),y_2,x_2\big ). \end{aligned}$$

Finally, the constant $z_{\textrm{b}}$ in (4.14) takes into account the exponential of the sum over “vacuum diagrams”, that is with no external lines. It is:

4.22 $$\begin{aligned} \log (z_{\textrm{b}}) = -\beta \lambda \frac{S}{2} \sum _{\vec x\in \Lambda _{L}} \varphi (\vec x) + \sum _{{\boldsymbol{k}}} \sum _{n}\sum _{s\ge 1} \bigg (\frac{1}{s!} \sum _{\theta \in \widetilde{{\mathscr {T}}}^{(\textrm{b})}_{s;n}} \theta (\boldsymbol{k})\bigg ), \end{aligned}$$

where the graphs in $\widetilde{{\mathscr {T}}}^{(\textrm{b})}_{s;n}$ are obtained as before, with the only difference that all lines are now paired. The sum over $k_{0}$, which is unbounded as $N\rightarrow \infty$, is performed using the estimate (4.12). This allows us to control all graphs with at least two nodes, hence two propagators. In the case of a graph with a single node, the so-called tadpole graph, an explicit computation shows that the $k_{0}$ sum is bounded uniformly in *N* (this is related to the fact that the propagator is aymptotically odd in $k_{0}$). Thus, $\log (z_{\textrm{b}})$ is analytic in λ for |λ| small enough, and furthermore:

4.23 $$\begin{aligned} |\log (z_{\textrm{b}}) | \le C |\lambda | \beta L_{1} L_{2}. \end{aligned}$$

### The Flow of the Effective Potentials

#### One-dimensional Reduction

We are now left with the integration of the massless Gaussian field, with covariance given by $G^{(\mathrm e)}$, Eq. (4.9). As we will see, this can be reduced to the analysis of a massless, one-dimensional field; a similar dimensional reduction argument has been used in [3, 48], for non-disordered, interacting systems. Let us introduce the Grassmann field $\psi ^{(\le 1)}_{\omega , {\boldsymbol{k}}}$, with measure $\mathbb {P}_{(\le 1)}$ and covariance:

4.24 $$\begin{aligned} \int \mathbb {P}_{(\le 1)}(d\psi ^{(\le 1)})\, \psi ^{(\le 1)-}_{\omega ,{\boldsymbol{k}}} \psi ^{(\le 1)+}_{\omega ', {\boldsymbol{h}}}:= &   \beta L_{1}\delta _{\omega ,\omega '} g^{(\le 1)}_{\omega }({\boldsymbol{k}}), \nonumber \\ g^{(\le 1)}_{\omega }({\boldsymbol{k}}):= &   \frac{\chi (k_{0}) \chi (\varepsilon _\omega (k_1)-\mu )}{ik_{0} + \varepsilon _{\omega }(k_{1}) - \mu }. \end{aligned}$$

It is understood that the fields are only defined for momenta ${\boldsymbol{k}}$ in the support of the propagators. The one-dimensional reduction of the effective model obtained after integrating out $\psi ^{(\textrm{b})}$ is based on the observation that the field $\psi ^{(\mathrm e)}_{{\boldsymbol{k}}, x_{2}, \sigma }$ can be represented as:

4.25 $$\begin{aligned} \psi ^{(\mathrm e)+}_{{\boldsymbol{k}}, x_{2}, \sigma } = \sum _{\omega = \pm } \psi ^{(\le 1)+}_{\omega , {\boldsymbol{k}}} \overline{\xi ^{\omega }_{\sigma }(k_{1}; x_{2})},\qquad \psi ^{(\mathrm e)-}_{{\boldsymbol{k}}, x_{2}, \sigma } = \sum _{\omega = \pm } \psi ^{(\le 1)-}_{\omega , {\boldsymbol{k}}} \xi ^{\omega }_{\sigma }(k_{1}; x_{2}). \end{aligned}$$

Using this representation of the field, we have [3, 48]:

4.26 $$\begin{aligned} \frac{\textsf{Z}}{\textsf{Z}^{0}} = z_{\textrm{b}} \int \mathbb {P}_{\mathrm e}( d \psi ^{(\mathrm e)})\exp \big ({\mathcal V^{(\textrm{e})}( \psi ^{(\mathrm e)})}\big ) = z_{\textrm{b}} \int \mathbb {P}_{(\le 1)}(d\psi ^{(\le 1)}) \exp \big ( \mathcal {V}^{(1)}(\psi ^{(\le 1)}) \big ),\nonumber \\ \end{aligned}$$

where

4.27 $$\begin{aligned} \mathcal {V}^{(1)}(\psi ^{(\le 1)}) = \frac{1}{\beta L_{1}} \sum _{n, {\boldsymbol{k}}} \sum _{\omega ,\omega '} V_{n;\omega ,\omega '}^{(1)}(\boldsymbol{k})\psi _{\boldsymbol{k},\omega }^{(\le 1)+} \psi _{\boldsymbol{k}+ n {\boldsymbol{\alpha }},\omega '}^{(\le 1)-} ; \end{aligned}$$

for ${\boldsymbol{k}}$ and ${\boldsymbol{k}} + n {\boldsymbol{\alpha }}$ in the support for the fields, the effective potential is:

4.28 $$\begin{aligned} V_{n;\omega ,\omega '}^{(1)}(\boldsymbol{k}) = \sum _{x_{2}, y_{2}} \sum _{\sigma ,\zeta } V_{n;\sigma ,\zeta }^{(\text {e})}(\boldsymbol{k},x_2,y_2) \overline{\xi ^{\omega }_{\sigma }(k_{1}; x_{2})} \xi ^{\omega '}_{\zeta }(k_{1}+ n\alpha ; y_{2}), \end{aligned}$$

while it is set to zero for ${\boldsymbol{k}}$ or ${\boldsymbol{k}} + n {\boldsymbol{\alpha }}$ not in the support of the fields.

Thus, in Eq. (4.26) we are reducing the computation of the partition function of the original 2*d* model to the analysis of the partition function of an effective one-dimensional model; the effective potential of this one-dimensional model corresponds to the “projection” of the original kernels, on the two edges. The effective potential is analytic for |λ| small enough; furthermore, from the bound (4.20) and the assumptions on the edge modes (2.19), we have:

4.29 $$\begin{aligned} \Big | \text {d}_{k_{0}}^{n_{0}} \text {d}_{k_{1}}^{n_{1}} V_{n;\omega ,\omega '}^{(1)}(\boldsymbol{k}) \Big | \le C_{n_{0},n_{1}} |\lambda | e^{-c|n|} e^{-{\tilde{c}}\delta _{\omega ,-\omega '}L_{2}}. \end{aligned}$$

In particular, we see that the scattering between the different edges is exponentially suppressed, for $L_{2}$ large. To conclude the paragraph, it is useful to write the first order contribution to the effective potential. It is:

4.30 $$\begin{aligned} V_{n;\omega ,\omega '}^{(1)}(\boldsymbol{k})= -\lambda \sum _{x_{2},\sigma } \overline{\xi ^{\omega }_{\sigma }(k_{1}; x_{2})} \xi ^{\omega '}_{\sigma }(k_{1} + n\alpha ; y_{2}) \hat{\varphi }_{-n}(x_{2})+\mathrm O(\lambda ^2). \end{aligned}$$

Thus, we see that, already at first order in λ, the two edge modes are coupled by the disorder. Also, we conclude by observing that the analogue of (4.21) holds true, as a consequence of the symmetry property (4.7):

4.31 $$\begin{aligned} \overline{V_{n;\omega ,\omega '}^{(1)}((k_{0}, k_{1}))} = V_{-n;\omega ',\omega }^{(1)}((-k_{0}, k_{1} + n\alpha )). \end{aligned}$$

#### Integration of the First Scale

**Shift of the Fermi points.** We now set up the multiscale integration of the one-dimensional field. In order to do this, it is convenient to allow for a small shift of the Fermi points. Let $\chi ^{(\le 1)}_{\omega }({\boldsymbol{k}}):= \chi (k_{0}) \chi (\varepsilon _\omega (k_1)-\mu )$. Let $\nu _{\omega }({\boldsymbol{k}}) = \nu _{\omega } \chi ^{(\le 1)}_{\omega }({\boldsymbol{k}})$ with $\nu _{\omega }$ real-valued, to be determined. This quantity plays the role of counterterm in our RG analysis; for the moment, its precise numerical value will not be important, and we will only assume that it satisfies $|\nu _{\omega }| \le C|\lambda |$. The value of $\nu _{\omega }$ will be chosen in a second moment via fixed point argument (see Proposition 4.9), in a way that allows us to prove the smallness of the relevant terms generated by the RG analysis. We rewrite the partition function as:

4.32 $$\begin{aligned} \textsf{Z} /\textsf{Z}^{0} = {\tilde{z}}_{\textrm{b}} \int \widetilde{\mathbb {P}}_{(\le 1)}(d\psi ^{(\le 1)}) \exp \big ( \widetilde{\mathcal {V}}^{(1)}(\psi ^{(\le 1)}) \big ) \end{aligned}$$

where: the new Grassmann Gaussian integration has covariance given by

4.33 $$\begin{aligned} {\tilde{g}}^{(\le 1)}_{\omega }({\boldsymbol{k}}) = \frac{\chi ^{(\le 1)}_{\omega }({\boldsymbol{k}})}{ik_{0} + \varepsilon _{\omega }(k_{1}) - \mu + \nu _{\omega }({\boldsymbol{k}})}; \end{aligned}$$

the constant ${\tilde{z}}_{\textrm{b}}$ takes into account the change in normalization of the Grassmann integration^2^; the new effective potential is:

4.35 $$\begin{aligned} \widetilde{\mathcal {V}}^{(1)}(\psi ^{(\le 1)})= &   \mathcal {V}^{(1)}(\psi ^{(\le 1)}) + \frac{1}{\beta L_{1}} \sum _{{\boldsymbol{k}}, \omega } \nu _{\omega } \psi _{\boldsymbol{k},\omega }^{(\le 1)+} \psi _{\boldsymbol{k},\omega }^{(\le 1)-} \nonumber \\  &   \equiv \frac{1}{\beta L_{1}} \sum _{\begin{array}{c} n, {\boldsymbol{k}}, \\ \omega , \omega ' \end{array}} \widetilde{V}_{n;\omega ,\omega '}^{(1)}(\boldsymbol{k}) \psi _{\boldsymbol{k},\omega }^{(\le 1)+}\psi _{\boldsymbol{k}+ n {\boldsymbol{\alpha }},\omega '}^{(\le 1)-} , \end{aligned}$$

with

4.36 $$\begin{aligned} \widetilde{V}_{n;\omega ,\omega '}^{(1)}(\boldsymbol{k}) = V_{n;\omega ,\omega '}^{(1)}(\boldsymbol{k}) + \delta _{n,0} \delta _{\omega ,\omega '} \nu _{\omega }. \end{aligned}$$

This expression is obtained adding and subtracting the term $\nu _{\omega }(\textbf{k})$ at the denominator of the Grassmann covariance and moving one of the two $\nu _{\omega }(\textbf{k})$’s in the effective potential. This new term will be used later on to control the flow of the relevant terms in the renormalization group sense. Thus, we now have that the denominator of the covariance is vanishing in correspondence with a slightly different point, to be denoted ${\boldsymbol{k}}_{F}^{\omega }(\lambda ) = \big (0, k_{F}^{\omega }(\lambda )\big )$, where $k^{\omega }_{F}(\lambda )$ solves:

4.37 $$\begin{aligned} \varepsilon _{\omega }\big (k^{\omega }_{F}(\lambda )\big ) - \mu + \nu _{\omega } = 0. \end{aligned}$$

Since $|\nu _{\omega }|\le C|\lambda |$, we also have that $| k^{\omega }_{F}(\lambda ) - k_{F}^{\omega } | \le K|\lambda |$. The new Fermi point is thus parametrized by $\nu _{\omega }$, and the final value of the renormalized Fermi points will be fixed after choosing $\nu _{\omega }$ as in Proposition (4.9). Observe that Eq. (4.37) might not have a solution in the discrete Brillouin zone $\mathscr {D}_{L}$. With a slight abuse of notation, we shall use the notation $k^{\omega }_{F}(\lambda )$ to denote the best lattice approximation of the solution of (4.37), for which the identity (4.37) holds with 0 in the right-hand side replaced by a quantity bounded by *C*/*L*. This offset will not play any role in what follows.

Next, we center the momenta around the two new Fermi points. We rewrite any ${\boldsymbol{k}}$ in the support of ${\tilde{g}}^{(\le 1)}_{\omega }({\boldsymbol{k}})$ as ${\boldsymbol{k}} = {\boldsymbol{q}} + {\boldsymbol{k}}_{F}^{\omega }(\lambda )$, with $\Vert {\boldsymbol{q}} \Vert \le C\delta$. With a slight abuse of notation, we shall set:

4.38 $$\begin{aligned} \psi ^{(\le 1)\pm }_{{\boldsymbol{q}} + {\boldsymbol{k}_{F}^{\omega }}(\lambda ), \omega } \equiv \psi ^{(\le 1)\pm }_{{\boldsymbol{q}}, \omega }. \end{aligned}$$

Whevener it does not create ambiguities, in what follows we may drop the λ-dependence of the shifted Fermi points.

**Field integration.** Let us denote by $\tilde{v}_{\omega }$ the Fermi velocity of the shifted Fermi point, $\tilde{v}_{\omega } = \partial _{k_{1}} \varepsilon _{\omega }\big (k_{F}^{\omega }(\lambda )\big )$. By the assumptions on the energy bands of the edge modes, $|v_{\omega } - {\tilde{v}}_{\omega }| \le C|\lambda |$. We define the new cutoff function, for γ>2:

4.39 $$\begin{aligned} \chi ^{(\le 0)}_{\omega }({\boldsymbol{q}}):= \chi \Big (\gamma \sqrt{ q_{0}^{2} + {\tilde{v}}_{\omega }^{2} q_{1}^{2} }\Big ). \end{aligned}$$

For γ large enough, the support of this cutoff function is strictly contained in the support of the cutoff function appearing in the definition of the covariance (4.24). Let us define the new propagator:

4.40 $$\begin{aligned} g^{(\le 0)}_{\omega }({\boldsymbol{q}}):= \frac{\chi ^{(\le 0)}_{\omega }({\boldsymbol{q}})}{iq_{0} + \varepsilon _{\omega }(q_{1}) - \mu + \nu _{\omega }}, \end{aligned}$$

where we used that, choosing γ large enough, $\nu _{\omega }\big ({\boldsymbol{q}} \big ) = \nu _{\omega }$ for ${\boldsymbol{q} }$ in the support of $\chi ^{(\le 0)}_{\omega }({\boldsymbol{q}})$. We rewrite the propagator ${\tilde{g}}^{(\le 1)}_{\omega }$ as:

4.41 $$\begin{aligned} {\tilde{g}}^{(\le 1)}_{\omega }\big ({\boldsymbol{q}} + {\boldsymbol{k}}_{F}^{\omega }\big ) = g^{(\le 0)}_{\omega }({\boldsymbol{q}}) + g^{(1)}_{\omega }({\boldsymbol{q}}) \end{aligned}$$

where $g^{(1)}_{\omega }({\boldsymbol{q}})$ is supported for momenta $(\delta /\gamma ^2) \lesssim \Vert {\boldsymbol{q}} \Vert \lesssim \delta$, and it is bounded together with all its derivatives:

4.42 $$\begin{aligned} \big | \text {d}_{k_{0}}^{n_{0}} \text {d}_{k_{1}}^{n_{1}} g^{(1)}_{\omega }({\boldsymbol{q}}) \big | \le C_{n_{0}, n_{1}}, \end{aligned}$$

for a constant $C_{n_{0}, n_{1}}$ which depends on γ.

We then represent the propagator $\psi ^{(\le 1)}$ as $\psi ^{(\le 1)} = \psi ^{(1)} + \psi ^{(\le 0)}$, where the field $\psi _{\omega }^{(1)}$ has covariance $g^{(1)}_{\omega }$ while the field $\psi _{\omega }^{(\le 0)}$ has covariance $g^{(\le 0)}_{\omega }$. By the addition principle,

4.43 $$\begin{aligned} \frac{\textsf{Z}}{\textsf{Z}^{0}}  &   = {\tilde{z}}_{\textrm{b}} \int \mathbb {P}_{(\le 0)}(d\psi ^{(\le 0)}) \int \mathbb {P}_{(1)}(d\psi ^{(1)}) e^{\widetilde{\mathcal {V}}^{(1)}(\psi ^{(\le 0)} + \psi ^{(1)})}\nonumber \\  &   \equiv {\tilde{z}}_{\textrm{b}} z_{1} \int \mathbb {P}_{(\le 0)}(d\psi ^{(\le 0)}) e^{\mathcal {V}^{(0)}(\psi ^{(\le 0)})}, \end{aligned}$$

where the single-scale integration is performed via a cumulant expansion, similarly to (4.15). The new effective potential has the form:

4.44 $$\begin{aligned} \mathcal {V}^{(0)}(\psi ^{(\le 0)}) = \frac{1}{\beta L_{1}} \sum _{n, {\boldsymbol{q}}} \sum _{\omega ,\omega '}V_{n;\omega ,\omega '}^{(0)}({\boldsymbol{q}}) \psi _{{\boldsymbol{q}},\omega }^{(\le 0)+} \psi _{{\boldsymbol{q}} + n {\boldsymbol{\alpha }} + s_{\omega \omega '},\omega '}^{(\le 0)-}, \end{aligned}$$

with $s_{\omega \omega '} = {\boldsymbol{k}}_{F}^{\omega } - {\boldsymbol{k}}_{F}^{\omega '}$, for new functions $V_{n;\omega ,\omega '}^{(0)}({\boldsymbol{q}})$. Informally, these new functions are easily understood diagrammatically, by contracting the old $\widetilde{V}_{n;\omega ,\omega '}^{(1)}(\cdot )$ through chains of single-scale propagators on scale 1. Similarly, the constant $z_{1}$ is obtained by collecting the vacuum diagrams constructed using the contractions of the single-scale propagators on scale 1. In the next section we will give a precise definition of the general structure of these objects, in a more general context, hence we refrain from giving more details here. What we can say at this stage is that the new constant $z_{1}$ and the new functions $V_{n;\omega ,\omega '}^{(0)}({\boldsymbol{q}})$ are analytic in λ for |λ| small enough, and satisfy similar properties as their counterparts $z_{\textrm{b}}$ and $V_{n;\omega ,\omega '}^{(1)}(\boldsymbol{k})$. In particular:

4.45 $$\begin{aligned} \Big | \text {d}_{q_{0}}^{n_{0}} \text {d}_{q_{1}}^{n_{1}} V_{n;\omega ,\omega '}^{(0)}(\boldsymbol{q}) \Big | \le C_{n_{0},n_{1}} |\lambda | e^{-c|n|} e^{-{\tilde{c}}\delta _{\omega ,-\omega '}L_{2}}. \end{aligned}$$

Also, as a consequence of the symmetry (4.7), we have:

4.46 $$\begin{aligned} \overline{V_{n;\omega ,\omega '}^{(0)}((q_{0}, q_{1}))} = V_{-n;\omega ',\omega }^{(0)}\big ((-q_{0}, q_{1} + n\alpha - k_{F}^{\omega } + k_{F}^{\omega '})\big ). \end{aligned}$$

The important remark at this point is that analyticity holds *for a smaller range of* λ, which so far depends on γ: this is due to the fact that the estimate (4.42) is sensitive to the smallest value that ${\boldsymbol{q}}$ can attain, which is now of order $1/\gamma ^3$ instead of order $1/\gamma ^2$. In order to iterate this integration to smaller scales, and to ensure a positive radius of convergence in λ, we will have to introduce a suitable localization and renormalization procedure.

#### Iterative Integration

In this section we shall discuss the integration of the smaller scales. To do so, we will have to introduce a suitable *localization* and *renormalization* procedure. This is the content of the next proposition.

##### Proposition 4.2

(Multiscale construction of the effective potential). Under the same assumption of Theorem 3.1, the following holds. Let $h_{\beta } \in \mathbb {Z}_{-}$ such that $\delta \gamma ^{h_{\beta } - 1} \le \frac{\pi }{\beta }\le \delta \gamma ^{h_{\beta }}$. Let $h \in \mathbb {Z}_{-}$, such that $h_{\beta } \le h$. The partition function can be rewritten as, for $|\lambda | < \lambda _{0}$:

4.47 $$\begin{aligned} \textsf{Z} /\textsf{Z}^{0} = z_{(> h)} \int \mathbb {P}_{(\le h)} (d\psi ^{(\le h)}) \exp \big ( \mathcal {V}^{(h)}(\psi ^{(\le h)}) \big ), \end{aligned}$$

with the following meaning of the various objects in (4.47).

(i) The field $\psi ^{(\le h)\pm }_{{\boldsymbol{q}}, \omega }$ has covariance given by:

4.48 $$\begin{aligned} \begin{aligned} \int \mathbb {P}_{(\le h)} (d\psi ^{(\le h)}) \psi ^{-}_{{\boldsymbol{q}}, \omega } \psi ^{+}_{{\boldsymbol{h}}, \omega '}&= \beta L_{1} \delta _{{\boldsymbol{q}}, {\boldsymbol{h}}} \delta _{\omega ,\omega '} g^{(\le h)}_{\omega }({\boldsymbol{q}}), \\ g^{(\le h)}_{\omega }({\boldsymbol{q}})&= \frac{\chi ^{(\le h)}_{\omega }({\boldsymbol{q}})}{i v_{0,\omega ,h+1} q_{0} + v_{1,\omega ,h+1} q_{1} + r_{\omega }(q_{1})}, \end{aligned} \end{aligned}$$

where: $\chi ^{(\le h)}_{\omega }({\boldsymbol{q}}) = \chi \Big (\gamma ^{-h-1} \sqrt{v_{0,\omega ,h+1}^{2}q_{0}^{2} + v_{1,\omega ,h+1}^{2}q_{1}^{2} }\Big )$ with $v_{0,\omega , h+1}$, $v_{1,\omega , h+1}$ real valued and such that:

4.49 $$\begin{aligned} | v_{0,\omega ,h+1} - 1 |\le C|\lambda |,\qquad | v_{1,\omega ,h+1} - v_{\omega }| \le C|\lambda |. \end{aligned}$$

The term $r_{\omega }(q_{1})$ is real-valued and it satisfies:

4.50 $$\begin{aligned} \big | \text {d}_{q_{1}}^{n_{1}} r_{\omega }(q_{1}) \big | \le C_{ n_{1}} | q_{1} |^{\max {(2 - n_{1},0)}}. \end{aligned}$$

(ii) The effective potential has the form:

4.51 $$\begin{aligned} \mathcal {V}^{(h)}(\psi ^{(\le h)}) = \frac{1}{\beta L_{1}} \sum _{n, {\boldsymbol{q}}} \sum _{\omega ,\omega '} V_{n;\omega ,\omega '}^{(h)}({\boldsymbol{q}}) \psi _{{\boldsymbol{q}},\omega }^{(\le h)+} \psi _{{\boldsymbol{q}} + n {\boldsymbol{\alpha }} + s_{\omega \omega '},\omega '}^{(\le h)-}\;, \end{aligned}$$

where $$V_{n;\omega ,\omega '}^{(h)}({\boldsymbol{q}})$$ satisfies:

4.52 $$\begin{aligned} \Big | \text {d}_{q_{0}}^{n_{0}} \text {d}_{q_{1}}^{n_{1}} V^{(h)}_{n;\omega ,\omega '}({\boldsymbol{q}}) \Big | \le K_{n_{0},n_{1}} \gamma ^{h(1 - n_{0} - n_{1})} |\lambda | e^{-\frac{c}{4}|n|} e^{-\frac{{\tilde{c}}}{4}\delta _{\omega ,-\omega '}L_{2}}, \end{aligned}$$

with $c, {\tilde{c}} >0$ as in (4.45). Furthermore,

4.53 $$\begin{aligned} \overline{V_{n;\omega ,\omega '}^{(h)}((q_{0}, q_{1}))} = V_{-n;\omega ',\omega }^{(h)}\big ((-q_{0}, q_{1} + n\alpha - k_{F}^{\omega } + k_{F}^{\omega '})\big ). \end{aligned}$$

(iii) The overall normalization constant satisfies:

4.54 $$\begin{aligned} \big | \log (z_{(> h)}) \big | \le C \beta L_{1} L_{2}. \end{aligned}$$

All constants appearing in the above estimates are uniform in the scale label β, and do not depend on and on $L_1, L_2$.

##### Remark 4.3

(i) The proof of the proposition actually yields an explicit construction of the objects appearing in Proposition 4.2 via a convergent expansion.
(ii) The above result shows that, in a quantitative sense, the low-energy renormalized covariance of the Grassmann field theory agrees with the one of massless, relativistic fermions in 1+1 dimensions (the term $r_{\omega }(q_{1})$ takes into account the nonlinearity of the bands), exposed to a suitable effective potential $\mathcal {V}^{(h)}$, which breaks translation-invariance, and which gets weaker on smaller scales.

The rest of Sect. 4.2 will be devoted to the proof of Proposition 4.2. We will proceed in an inductive fashion. Observe that the claims of the Proposition are true on scale 0, by the discussion of Sects. 4.2.1, 4.2.2.

**Localization and renormalization.** In order to integrate the scale *h*, we will renormalize the Gaussian integration, by reabsorbing into the covariance the dangerous contributions to the effective potential. Let ${\boldsymbol{0}}^{+}_{\beta }:= (\pi /\beta , 0)$, ${\boldsymbol{0}}^{-}_{\beta }:= (-\pi /\beta , 0)$ and let, for an arbitrary function *F*:

4.55 $$\begin{aligned} F({\boldsymbol{0}}_{\beta }):= \frac{1}{2} \sum _{\alpha = \pm } F({\boldsymbol{0}}^{\alpha }_{\beta }). \end{aligned}$$

We are now ready to introduce the notion of localization.

##### Definition 4.4

*(Localization operator)*. Let $V^{(h)}_{n;\omega ,\omega '}({\boldsymbol{q}})$ be a coefficient of the effective potential (4.51). We define:

4.56 $$\begin{aligned} \mathfrak L V^{(h)}_{n;\omega ,\omega '}({\boldsymbol{q}}):= \delta _{n,0} \delta _{\omega ,\omega '}\big (V^{(h)}_{n;\omega ,\omega '}({\boldsymbol{0}}_{\beta }) + q_{0} \text {d}_{q_{0}}\! V^{(h)}_{n;\omega ,\omega '}({\boldsymbol{0}}_{\beta }) + q_{1} \text {d}_{q_{1}}\! V^{(h)}_{n;\omega ,\omega '}({\boldsymbol{0}}_{\beta })\big ). \nonumber \\ \end{aligned}$$

The action of $\mathfrak {L}$ is extended to $\mathcal {V}^{(h)}$ by linearity. Furthermore, we define:

4.57 $$\begin{aligned} \mathfrak RV^{(h)}_{n;\omega ,\omega '}({\boldsymbol{q}}):= V^{(h)}_{n;\omega ,\omega '}({\boldsymbol{q}}) - \mathfrak L V^{(h)}_{n;\omega ,\omega '}({\boldsymbol{q}}). \end{aligned}$$

We shall also set:

4.58 $$\begin{aligned} \mathfrak L_{0} V^{(h)}_{n;\omega ,\omega }({\boldsymbol{q}}):= &   \delta _{n,0} V^{(h)}_{0;\omega ,\omega }({\boldsymbol{0}}_{\beta }),\quad \nonumber \\ \mathfrak L_{1} V^{(h)}_{n;\omega ,\omega }({\boldsymbol{q}}):= &   \delta _{n,0}\big ( q_{0} \text {d}_{q_{0}}\! V^{(h)}_{n;\omega ,\omega }({\boldsymbol{0}}_{\beta }) + q_{1} \text {d}_{q_{1}}\! V^{(h)}_{n;\omega ,\omega }({\boldsymbol{0}}_{\beta })\big ), \end{aligned}$$

and hence $$\mathfrak L = \mathfrak L_{0} + \mathfrak L_{1}$$.

##### Remark 4.5

(i) The operator L is called the localization operator, and its role is to extract from the effective potential $\mathcal {V}^{(h)}$ the relevant and marginal terms in the renormalization group sense; these terms require a separate discussion. Ultimately, they will be controlled by a suitable choice of the parameters $\nu _{\omega }$, entering the definition of shifted chemical potentials.
(ii) The operator $\mathfrak L_{0}$ isolates the *relevant terms* in the renormalization group sense, while the operator $\mathfrak L_{1}$ isolates the *marginal terms* in the renormalization group sense. The naive dimensional estimate for the former diverges exponentially in |*h*|, while the naive dimensional estimate for the latter diverge linearly in |*h*|. Observe that we are not defining a localization operator on the “off-diagonal terms”, that is the terms involving different $\omega ,\omega '$ labels. Being associated with different edge modes, these contributions turn out to be exponentially small in $L_{2}$, recall the bound (4.52). In particular, we will be able to define a range of temperatures and of cylinder widths for which the edge-edge scatterings are subleading.
(iii) The reader might wonder why in the definition of localization we are only selecting the n=0 terms. As we will see, thanks to the Diophantine properties of α, Eq. (2.9), the contributions to the effective potential associated with n≠0 and $\omega =\omega '$ will be suppressed by an extra, scale dependent, small factor.
(iv) It is important to observe that the operator R acts as a projection: $\mathfrak R^{2} V^{(h)}_{n}({\boldsymbol{q}}) = \mathfrak R V^{(h)}_{n}({\boldsymbol{q}})$. If n≠0, this is trivial, since ${\mathfrak R}$ acts as the identity in this case. If n=0, it follows from an explicit check, we omit the details.
(v) Observe that, by (4.55), we are defining the localization in a “symmetrized” way. This definition combined with (4.53) implies:
  4.59 $$\begin{aligned} \begin{aligned} \overline{{\mathfrak L} V^{(h)}_{0;\omega ,\omega }((q_{0}, q_{1}))}&= {\mathfrak L} V^{(h)}_{0;\omega ,\omega }((- q_{0}, q_{1})), \\ \overline{{\mathfrak R} V^{(h)}_{n;\omega ,\omega '}((q_{0}, q_{1}))}&= {\mathfrak R} V^{(h)}_{-n;\omega ',\omega }\big ((- q_{0}, q_{1} + n\alpha - k_{F}^{\omega } + k_{F}^{\omega '})\big ). \end{aligned} \end{aligned}$$
  These properties will be important to ensure the reality properties of the velocities appearing in the propagator on smaller scales.

**The beta function.** Let us now use the localization operator to rewrite the argument of the Grassmann integral in a way which is more convenient for setting up an iterative integration. We have:

4.60 $$\begin{aligned} \begin{aligned} \int \mathbb {P}_{(\le h)} (d\psi ^{(\le h)}) \,e^{\mathcal {V}^{(h)}(\psi ^{(\le h)})}&= \int \mathbb {P}_{(\le h)} (d\psi ^{(\le h)})\, e^{(\mathfrak L_{0} + \mathfrak L_{1} + \mathfrak R) \mathcal {V}^{(h)}(\psi ^{(\le h)})} \\&= {\tilde{z}}_{h} \int \widetilde{\mathbb {P}}_{(\le h)} (d\psi ^{(\le h)})\, e^{(\mathfrak L_{0} + \mathfrak R) \mathcal {V}^{(h)}(\psi ^{(\le h)})}, \end{aligned} \end{aligned}$$

where $\widetilde{\mathbb {P}}_{(\le h)}$ is a new Grassmann Gaussian integration, whose covariance is *renormalized* by including $\mathfrak L_{1} \mathcal {V}^{(h)}$ into its definition. The constant ${\tilde{z}}_{h}$ takes into account the change of normalization of the integral, and it is bounded as $|{\tilde{z}}_{h}| \le \text {exp}(\beta L_{1} C|\lambda | \gamma ^{h})$. Let us discuss the structure of the new covariance. To this end, we define:

4.61 $$\begin{aligned} -\text {d}_{q_{0}}\! V^{(h)}_{0;\omega ,\omega }({\boldsymbol{0}}_{\beta }) =: i \beta ^{v}_{0,h+1,\omega },\qquad -\text {d}_{q_{1}}\! V^{(h)}_{0;\omega ,\omega }({\boldsymbol{0}}_{\beta }) =: \beta ^{v}_{1,h+1,\omega }. \end{aligned}$$

Observe that, thanks to (4.53),

4.62 $$\begin{aligned} \overline{\beta ^{v}_{\nu ,\omega ,h+1}} = \beta ^{v}_{\nu ,\omega ,h+1} \qquad \nu = 0,1, \end{aligned}$$

that is $\beta ^{v}_{\nu ,\omega ,h+1}$ is real. The propagator of the new Grassmann integration in (4.60) is:

4.63 $$\begin{aligned} \widetilde{g}^{(\le h)}_{\omega }({\boldsymbol{q}}):= \frac{\chi ^{(\le h)}_{\omega }({\boldsymbol{q}})}{i v_{0,\omega ,h}({\boldsymbol{q}}) q_{0} + v_{1,\omega ,h}({\boldsymbol{q}}) q_{1} + r_{\omega }(q_{1})} \end{aligned}$$

where:

4.64 $$\begin{aligned} v_{\nu ,\omega ,h}({\boldsymbol{q}}):= v_{\nu ,\omega ,h+1} + \beta ^{v}_{\nu ,\omega ,h+1} \chi _{\omega }^{(\le h)}({\boldsymbol{q}}). \end{aligned}$$

By (4.62), the function $v_{\nu ,\omega ,h}({\boldsymbol{q}})$ is real-valued. For later purposes, we shall also define:

4.65 $$\begin{aligned} v_{\nu ,\omega ,h}:= v_{\nu ,\omega ,h}({\boldsymbol{0}}_{\beta }); \end{aligned}$$

by the properties of the function $\chi _{\omega }^{(\le h)}(\cdot )$, we have $v_{\nu ,\omega ,h}({\boldsymbol{q}}) = v_{\nu ,\omega ,h}$ for

4.66 $$\begin{aligned} \sqrt{v_{0,\omega ,h+1}^{2}q_{0}^{2} + v_{1,\omega ,h+1}^{2}q_{1}^{2} } \le \delta \gamma ^{h-1}. \end{aligned}$$

Thus, the new propagator has a form very similar to the previous one, up to a renormalization of order λ of the parameters. Next, let us look at the effective potential in the last step of (4.60). We set, recalling (4.58):

4.67 $$\begin{aligned} \mathfrak L_{0} V^{(h)}_{0;\omega ,\omega }({\boldsymbol{q}}) =: \gamma ^{h} \nu _{\omega ,h},\qquad \beta ^{\nu }_{\omega ,h+1}:= \gamma ^{-1} \nu _{\omega ,h} - \nu _{\omega ,h+1}. \end{aligned}$$

As a consequence of (4.53), the running coupling constant $\nu _{\omega ,h}$ is real. Thus, the *flow of the running coupling constants* is:

4.68 $$\begin{aligned} \nu _{\omega ,h} = \gamma \nu _{\omega ,h+1} + \gamma \beta ^{\nu }_{\omega ,h+1},\qquad v_{\nu ,\omega ,h} = v_{\nu ,\omega ,h+1} + \beta ^{v}_{\nu ,\omega ,h+1}. \end{aligned}$$

The initial datum of this discrete dynamical system is read off from (4.39), (4.43). Thanks to (4.45), it satisfies the estimates:

4.69 $$\begin{aligned} v_{0,\omega ,0} = 1,\qquad |v_{1,\omega ,0} - v_{\omega }| \le C|\lambda |,\qquad |\nu _{\omega ,0} - \nu _{\omega }|\le C|\lambda |. \end{aligned}$$

The sequence $( \beta ^{v}_{\nu ,\omega ,h}, \beta ^{\nu }_{\omega ,h} )_{h = h_{\beta } + 1}^{0}$ defines the *beta function* of the model. It is a nontrivial task to prove bounds for the beta function that are summable in the scale label, and that allow to prove that the solution of the recursion relation (4.68) is bounded uniformly in *h*. The first equation in (4.68) describes the flow of the *relevant* running coupling constants, while the second equation in (4.68) describes the flow of the marginal running coupling constants. One of our key technical goals will be to prove that, for a suitable choice of $\nu _{\omega } = O(\lambda )$, and for $L_{2} \ge \kappa \beta$:

4.70 $$\begin{aligned} |v_{\nu ,\omega ,h} - v_{\nu ,\omega ,0}| \le C|\lambda |^{2},\qquad |\nu _{\omega ,h}| \le C|\lambda | \gamma ^{\xi h}\;\qquad \text {for } \xi > 0. \end{aligned}$$

##### Remark 4.6

Thus, we can allow for a cylinder with finite width $L_{2}$, provided that the width grows at least linearly in the inverse temperature β. In this range of parameters, the two edge modes living on the two boundaries of $\Lambda _{L}$ are only weakly correlated.

**Single-scale integration.** We now integrate the scale *h*. This is done by writing the propagator (4.63) as:

4.71 $$\begin{aligned} \widetilde{g}^{(\le h)}_{\omega }({\boldsymbol{q}}) = g^{(\le h-1)}_{\omega }({\boldsymbol{q}}) + g^{(h)}_{\omega }({\boldsymbol{q}}), \end{aligned}$$

where:

4.72 $$\begin{aligned} g^{(\le h-1)}_{\omega }({\boldsymbol{q}}):= &   \frac{\chi ^{(\le h-1)}_{\omega }({\boldsymbol{q}})}{i v_{0,\omega ,h} q_{0} + v_{1,\omega ,h}q_{1} + r_{\omega }(q_{1})}, \nonumber \\ g^{(h)}_{\omega }({\boldsymbol{q}}):= &   \frac{f^{(h)}_{\omega }({\boldsymbol{q}})}{i v_{0,\omega ,h}({\boldsymbol{q}}) q_{0} + v_{1,\omega ,h}({\boldsymbol{q}}) q_{1} + r_{\omega }(q_{1})}, \end{aligned}$$

with $\chi ^{(\le h-1)}_{\omega }(\cdot )$ defined as after (4.48) with *h* replaced by h-1, and $f^{(h)}_{\omega }({\boldsymbol{q}}) = \chi ^{(\le h)}_{\omega }({\boldsymbol{q}}) - \chi ^{(\le h-1)}_{\omega }({\boldsymbol{q}})$. Observe that, at the denominator of $g^{(\le h-1)}_{\omega }({\boldsymbol{q}})$, the functions $v_{\nu ,\omega ,h}({\boldsymbol{q}})$ have been replaced by the real constants $v_{\nu ,\omega ,h}$, recall Eq. (4.65); this is due to the fact that the condition (4.66) is satisfied in the support of $\chi ^{(\le h-1)}_{\omega }(\cdot )$, up to possibly choosing a larger value of γ once and for all.

In constrast to $g^{(\le h-1)}$, the single-scale propagator $g^{(h)}$ is bounded; it satisfies the estimate:

4.73 $$\begin{aligned} \Big | \text {d}_{q_{0}}^{n_{0}} \text {d}_{q_{1}}^{n_{1}} g^{(h)}_{\omega }({\boldsymbol{q}}) \Big | \le C_{n_{0}, n_{1}} \gamma ^{-h (1 + n_{0} + n_{1})}. \end{aligned}$$

We are now ready to integrate the scale *h*. We represent the Grassmann field as $\psi ^{(\le h)} = \psi ^{(\le h-1)} + \psi ^{(h)}$, where $\psi ^{(\le h-1)}$, $\psi ^{(h)}$ are independent fields with propagators given by (4.72). We then rewrite the partition function (4.47) as:

4.74 $$\begin{aligned} \begin{aligned} \frac{\textsf{Z}}{\textsf{Z}^{0}}&= z_{(> h)} {\tilde{z}}_{h} \int \mathbb {P}_{(\le h-1)} (d\psi ^{(\le h-1)}) \int \mathbb {P}_{(h)}(d\psi ^{(h)}) e^{(\mathfrak L_{0} + \mathfrak R) \mathcal {V}^{(h)}(\psi ^{(\le h-1)} + \psi ^{(h)})} \\&= z_{(> h)} {\tilde{z}}_{h} z_{h} \int \mathbb {P}_{(\le h-1)} (d\psi ^{(\le h-1)}) e^{\mathcal {V}^{(h-1)}(\psi ^{(\le h-1)})}, \end{aligned} \end{aligned}$$

for a new effective potential $\mathcal {V}^{(h-1)}(\psi ^{(\le h-1)})$ which is obtained via a cumulant expansion associated with the Gaussian integration over the $\psi ^{(h)}$ field, and where $z_{h}$ satisfies $|z_{h}| \le \text {exp}(\beta L_{1} C|\lambda | \gamma ^{h})$, as it will follow from the forthcoming analysis. The expression obtained in (4.74) has the form of (4.47) with *h* replaced by h-1, after defining $z_{(>h-1)}:= z_{(> h)} {\tilde{z}}_{h} z_{h}$. Notice that the identity (4.53) follows from (4.7).

In order to prove the inductive assumption stated at the beginning of this section, and to actually derive an explicit representation for the effective potential at all scales, we will introduce a graphical representation for the effective potential at a given scale in terms of a hierarchy of *clusters*. This will be the goal of the next section.

#### Chain Expansion

The goal of this section will be to set up a graphical representation for the effective potential at all scales, which will be useful to prove estimates for the kernels of the potential, and in particular to prove Eq. (4.52). This representation has been introduced in [8] and it has been used in many subsequent works. It is similar to the one used recently in [23]. The main difference here is the presence of two Fermi points instead of one, corresponding to the two edge modes of the system. However, thanks to the localization properties of the edge modes, the fields corresponding to the two Fermi points will be essentially independent, provided $L_{2}$ is chosen large enough. Since the structure of the expansion will be used in the rest of the paper, we prefer to rederive it, rather than referring to previous papers and stating the differences.

Being the Grassmann theory quadratic in the fermions, the graphical representation will involve chain graphs, as in Sect. 4.1. We will express the kernel of the effective potential on scale h-1 as:

4.75 $$\begin{aligned} V^{(h-1)}_{n;\omega ,\omega '}({\boldsymbol{q}}) = \sum _{s \ge 1} \bigg (\frac{1}{s!} \sum _{\theta \in {\mathscr {T}}_{n;s, \omega ,\omega '}^{(h)}}\theta ({\boldsymbol{q}})\bigg ), \end{aligned}$$

where ${\mathscr {T}}_{n;s, \omega ,\omega '}^{(h)}$ is the set of labelled chains on scale *h*, to be defined after Eq. (4.83) below, and $\theta ({\boldsymbol{q}})$ is the value associated with each chain with external quasi-momentum ${\boldsymbol{q}}$. Let us explain how the graphs in ${\mathscr {T}}_{n;s, \omega ,\omega '}^{(h)}$ are defined. After the integration of the scale *h*, the effective potential is expressed as a sum of chain graphs similarly to what we discussed in (4.18). The definition of the values of the chains proceeds as after (4.18), with the following differences. To every node *v*, v=1,…,s, we attach a frequency label $n_{v}$, and two labels, $\omega _{v}$ and $\omega '_{v}$, associated respectively with the outgoing and the incoming line to the node; we impose the constraint $\omega '_{s} = \omega '$ and $\omega _{1} = \omega$. The edge outgoing from a node *v* is associated with a single scale propagator, $g^{(h)}_{\omega _{v}}\big ({\boldsymbol{q}} + {\boldsymbol{n}}(v)\big )$, where:

4.76 $$\begin{aligned} {\boldsymbol{n}}(v):= \sum _{v'\le v} \Big (n_{v'} {\boldsymbol{\alpha }} - {\boldsymbol{k}}_{F}^{\omega _{v}} + {\boldsymbol{k}}_{F}^{\omega '_{v}}\Big ) \end{aligned}$$

has the interpretation of quasi-momentum outgoing a vertex *v*. The sum over all the quasi-momentum shifts satisfies ${\boldsymbol{n}}(v) = n {\boldsymbol{\alpha }} - \delta _{n\ne 0}({\boldsymbol{k}}_{F}^{\omega }(\lambda ) - {\boldsymbol{k}}_{F}^{\omega '}(\lambda ))$. To every node of the chain graph we associate:

4.77 $$\begin{aligned} N^{(h)}_{n_{v}; \omega _{v}, \omega '_{v}}\big ({\boldsymbol{q}} + {\boldsymbol{n}}'(v)\big ):= (\mathfrak L_{0} + \mathfrak R) V^{(h)}_{n_{v};\omega _{v},\omega '_{v}}\big ({\boldsymbol{q}} + {\boldsymbol{n}}'(v)\big ) \end{aligned}$$

where ${\boldsymbol{n}}'(v) = {\boldsymbol{n}}(v-1)$: the momentum ${\boldsymbol{q}} + {\boldsymbol{n}}'(v)$ has the interpretation of incoming quasi-momentum in the node *v*. For later convenience, we shall also set ${\boldsymbol{n}}(v) = {\boldsymbol{n}}'(v) + \delta {\boldsymbol{n}}(v)$.

Thus, we can represent $V^{(h-1)}_{n;\omega ,\omega '}$ as the sum of chain graphs with the following values (compare with (4.19)):

4.78 $$\begin{aligned} \theta ({\boldsymbol{q}}) = \Big (\prod _{v=1}^s N^{(h)}_{n_{v}; \omega _{v}, \omega '_{v}}\big ({\boldsymbol{q}} + {\boldsymbol{n}}'(v)\big ) \Big ) \Big (\prod _{v=1}^{s-1}g^{(h)}_{\omega _{v}}\big ({\boldsymbol{q}} + {\boldsymbol{n}}(v)\big )\Big ). \end{aligned}$$

We can obtain a more explicit expression combining the definition (4.77) with the identity (4.78). This allows us to express $V^{(h-1)}_{n;\omega ,\omega '}({\boldsymbol{q}})$ in terms of new chain graphs, whose edges are associated with propagators on all scales from *h* to 0; this is done as follows. We write

4.79 $$\begin{aligned} N^{(h)}_{n_{v}; \omega _{v}, \omega '_{v}}\big ({\boldsymbol{q}} + {\boldsymbol{n}}'(v)\big ) = \mathfrak L_{0} V^{(h)}_{n_{v};\omega _{v},\omega '_{v}}\big ({\boldsymbol{q}} + {\boldsymbol{n}}'(v)\big ) + \mathfrak R V^{(h)}_{n_{v};\omega _{v},\omega '_{v}}\big ({\boldsymbol{q}} + {\boldsymbol{n}}'(v)\big ); \end{aligned}$$

for the first term in the right-hand side, we recall the definitions of Eqs. (4.58), (4.67):

4.80 $$\begin{aligned} \mathfrak L_{0} V^{(h)}_{n_{v};\omega _{v},\omega '_{v}}\big ({\boldsymbol{q}} + {\boldsymbol{n}}'(v)\big ) = \delta _{n_{v},0} \delta _{\omega _{v},\omega '_{v}}\gamma ^{h} \nu _{\omega _{v},h}. \end{aligned}$$

The values of the running coupling constants are determined recursively, by solving (4.68). Now, if $\mathfrak R V^{(h)}_{n_{v};\omega _{v},\omega '_{v}}(\cdot )$ was not present in (4.79), the expression (4.78) combined with (4.80) would give rise to chains whose nodes are labelled by running coupling constants and propagators on scale *h*. We shall call (4.80) the *nodes on scale h*, and we shall denote by $\mathscr {N}_{h}(\theta )$ the set of nodes on scale *h* of a chain θ in ${\mathscr {T}}_{n;s, \omega ,\omega '}^{(h)}$. The value of each such node will be denoted by $V_{v}$, and it corresponds to the right-hand side of (4.80).

In order to take into account the presence of $\mathfrak R V^{(h)}_{n_{v};\omega _{v},\omega '_{v}}(\cdot )$ in (4.79), we proceed as follows. Let $\widetilde{\mathscr {N}}_{h}(\theta )$ be the nodes that are associated with $\mathfrak R V^{(h)}$. We express $V^{(h)}$ at the argument of R in terms of chains involving propagators on scale $g^{(h+1)}$, nodes on scale h+1, and kernels $\mathfrak R V^{(h+1)}$. Thus, we can express the value of every chain $\theta \equiv \theta _{h}$ in ${\mathscr {T}}_{n;s, \omega ,\omega '}^{(h)}$ as a linear combination of:

4.81 $$\begin{aligned} \Big ( \prod _{v \in \mathscr {N}_{h}(\theta _{h})} V_{v}\Big ) \Big (\prod _{v=1}^{s-1}g^{(h)}_{\omega _{v}}\big ({\boldsymbol{q}} + {\boldsymbol{n}}(v)\big )\Big ) \bigg ( \prod _{v\in \widetilde{\mathscr {N}}_{h}(\theta _{h})} \mathfrak R\Big ( \theta _{h+1}\big ({\boldsymbol{q}} + {\boldsymbol{n}}'(v)\big )\Big ) \bigg ), \end{aligned}$$

where $\theta _{h+1}$ at the argument of the last product belongs to ${\mathscr {T}}_{n_{v};w_{v}, \omega _{v},\omega _{v}'}^{(h+1)}$. Recall the action of R, see Definition 4.4:

4.82 $$\begin{aligned} \mathfrak R\big ( \theta ({\boldsymbol{q}})\big )  &   = \theta ({\boldsymbol{q}}),\qquad \text {if } \theta \in {\mathscr {T}}_{n_{v};s_{v}, \omega _{v},\omega _{v}'}^{(h+1)} \text { with } n_{v}\ne 0 \text { and } \omega _{v} = \omega '_{v},\nonumber \\  &   \qquad \qquad \qquad \text { or with } \omega _{v} \ne \omega '_{v}, \end{aligned}$$

and:

4.83 $$\begin{aligned} \mathfrak R\big ( \theta ({\boldsymbol{q}})\big ) = \Big ( \theta ({\boldsymbol{q}}) - \theta ({\boldsymbol{0}}_{\beta }) - q_{0} \text {d}_{q_{0}} \theta ({\boldsymbol{0}}_{\beta }) - q_{1} \text {d}_{q_{1}} \theta ({\boldsymbol{0}}_{\beta }) \Big ) \qquad \text {otherwise.} \end{aligned}$$

In the final estimate for the renormalized graph, the accumulation of derivatives is avoided by the fact that the operator R annihilates first order polynomials in the momentum variable. Iterating the above argument, we end up with chain graphs involving nodes and propagators from scales *h* to 0. Let us introduce the necessary concepts in order to describe the resulting expansion.

(i) The chain is formed by a sequence of nodes connected by edges. Each node *v* is labelled by a scale label $h_{v} \ge h$, by a frequency label $n_{v}$, by quasi-particle labels $(\omega _{v}, \omega '_{v})$. The nodes are decorated by an incoming and an outgoing line; the incoming line is decorated by the quasi-particle label $\omega '_{v}$, the outgoing line is decorated by the quasi-particle label $\omega _{v}$. The chain is formed joining lines attached to nodes, with opposite orientations and with coinciding quasi-particle labels.
(ii) The nodes with scale $h_{v} < 0$ correspond to $n_{v} = 0$. They correspond to the running coupling constants (4.80). The nodes with $h_{v} = 0$ might have $n_{v} = 0$ or $n_{v} \ne 0$. If they are labelled by $n_{v} \ne 0$, they correspond to ${\mathfrak R}V^{(0)}_{n_{v}; \omega _{v}, \omega '_{v}}(\cdot )$. If they are labelled by $n_{v} = 0$, they correspond to either the running coupling constant (4.80), or to ${\mathfrak R} V^{(0)}_{n_{v}; \omega _{v}, \omega '_{v}}(\cdot )$.
(iii) The nodes associated with $n_{v} = 0$ and $\omega _{v} = \omega '_{v}$ are called *resonant*, while all the other nodes are called *non-resonant*. We denote by $\mathscr {N}(\theta )$ the set of nodes, by $\mathscr {N}_{\text {r}}(\theta )$ the set of resonant nodes, and by $\mathscr {N}_{\text {nr}}(\theta )$ the set of non-resonant nodes.
(iv) Two consecutive nodes v,v+1 are connected by an edge *e*, corresponding to a propagator $g^{(h_{e})}_{\omega _{v}}$ where $\omega _e \equiv \omega _v$. We denote by $\mathscr {E}(\theta )$ the set of all edges of θ. The scale *h* is comprised between the scale *h* and the minimum of $h_{v}$, $h_{v+1}$. The incoming momentum of the whole chain is ${\boldsymbol{q}}$, and the outgoing momentum of the chain is ${\boldsymbol{q}} + {\boldsymbol{n}}(|\mathscr {N}(\theta )|)$ with ${\boldsymbol{n}}(v)$ as in (4.76). Each propagator supports a momentum which is determined by the incoming momentum plus the momentum shift determined by the frequency labels of all vertices preceding the edge (Kirchhoff rule): if the edge *e* connects *v* to v+1, then the corresponding propagator supports the momentum ${\boldsymbol{q}}_{e} = {\boldsymbol{q}} + {\boldsymbol{n}}(v)$. Given a scale arrangement on the edges, we define the external scale $h_{v}^\textrm{ext}$ of a node *v* as the maximum of the scales of the edges attached to *v*.
(v) A *cluster* *T* is a connected segment contained in the chain θ, so that the incoming line and the outgoing line of *T* have scale labels strictly smaller than the scales contained in the cluster. We use the convention that clusters must contain at least one edge: that is, nodes are not clusters. We denote by $\mathscr {C}(\theta )$ the set of clusters strictly contained in θ and by $\mathscr {C}_{0}(\theta )$ the set $\mathscr {C}(\theta ) \cup \theta$. We denote by $h^{\text {ext}}_{T}$ is the maximal scale of the external lines of *T* (the *external scale* of *T*), and by $h_{T}$ the smallest scale contained in *T* (the *scale* of *T*); as a consequence of the above definitions, $h^{\text {ext}}_{T} < h_{T}$.
(vi) A cluster is *resonant* if the incoming and the outgoing momenta of *T* are equal, and if the quasi-particle labels of the incoming and of the outgoing lines are equal. Otherwise, the cluster is called *non-resonant.* We denote by $$\mathscr {C}_{\text {r}}(\theta )$$ the set of resonant clusters strictly contained in θ, and by $$\mathscr {C}_{\text {nr}}(\theta )$$ the set of non-resonant clusters strictly contained in θ. In particular, $$\mathscr {C}(\theta ) = \mathscr {C}_{\text {r}}(\theta )\cup \mathscr {C}_{\text {nr}}(\theta )$$. A cluster $T^{\prime }\subset T$ is *maximal* if it is not contained into any other subcluster of *T*. Similarly, a node v∈T is called maximal if it is not contained into any other subcluster of *T*. We call $$s^{\text {c}}_{T}$$ the number of maximal clusters contained in *T*, $$s^{\text {v}}_{T}$$ the number of maximal nodes contained in *T*, and we set $$s_{T} = s^{\text {c}}_{T} + s^{\text {v}}_{T}$$. We denote by $$s_{T}^{\text {nr}}$$ the number of maximal non-resonant clusters or nodes contained in *T*, and by $$s^{\text {r}}_{T}$$ the number of maximal resonant clusters or nodes contained in *T*.

The next proposition is the main technical result of the section.

##### Proposition 4.7

(Bound for chain graphs). Let $\theta \in {\mathscr {T}}_{n;s, \omega ,\omega '}^{(h)}$. Then, assuming the validity of (4.70) the following estimate holds true:

4.84 $$\begin{aligned} \begin{aligned} | \text {d}_{0}^{n_{0}} \text {d}_{1}^{n_{1}} \theta ({\boldsymbol{q}}) |&\le C_{n_{0},n_{1}}\gamma ^{h(1 - n_{0} - n_{1})} e^{- \varepsilon (\theta )2^{h} C \gamma ^{-h / \tau }} K^{|\mathscr {N}(\theta )|} |\lambda |^{|\mathscr {N}_{\text {nr}}(\theta )|} \Big ( \prod _{v\in \mathscr {N}(\theta )} e^{-\frac{c}{2}|n_{v}|} \Big ) \\&\quad \cdot \Big ( \prod _{v\in \mathscr {N}_{\text {r}}(\theta )} |\nu _{\omega _{v},h_{v}}| \Big )\Big (\prod _{v\in \mathscr {N}_{\text {nr}}(\theta )} e^{- \frac{{\tilde{c}}}{4} \delta _{\omega _{v},-\omega _{v}} L_{2}} \Big ) \Big (\prod _{T \in \mathscr {C}(\theta ) \cup \mathscr {N}_{\text {nr}}(\theta )} \gamma ^{ (h^{\text {ext}}_{T} - h_{T})}\Big ) \end{aligned}\nonumber \\ \end{aligned}$$

where $\varepsilon (\theta ) = 1$ if the cluster is non-resonant and $\varepsilon (\theta ) = 0$ otherwise.

The bound (4.84) is summable over the scale labels, and over the frequency labels. In fact:

4.85 $$\begin{aligned} \sum _{\{n_{v}\}: \sum _{v} n_{v} = n}\Big ( \prod _{v\in \mathscr {N}(\theta )} e^{-\frac{c}{2}|n_{v}|}\Big )\le &   C^{|\mathscr {N}(\theta )|} e^{-\frac{c}{4} |n|} \quad \text {and}\quad \nonumber \\ \sum _{\{h_{T}\}: h^{\text {ext}}_{\theta } = h}\Big (\prod _{T \in \mathscr {C}(\theta )} \gamma ^{ (h^{\text {ext}}_{T} - h_{T})}\Big )\le &   1. \end{aligned}$$

Concerning the combinatorics for a given cluster *T*, the number of chain graphs obtained connecting $s_{T}$ maximal clusters or nodes is $s_{T}!$. On the other hand, following the iterative construction of the chain graphs discussed starting from (4.75), we see that the value of the resulting graphs is accompanied by a prefactor $\prod _{T \in \mathscr {C}_{0}(\theta )} (1/s_{T}!)$. Thus, the overall combinatorial factor is 1. In conclusion, from (4.75) we get, up to a redefinition of the overall constant and for |λ| small enough:

4.86 $$\begin{aligned} | \text {d}_{0}^{n_{0}} \text {d}_{1}^{n_{1}} V^{(h-1)}_{n;\omega ,\omega '}({\boldsymbol{q}})| \le C_{n_{0},n_{1}}|\lambda | \gamma ^{(h-1)(1 - n_{0} - n_{1})} e^{-\frac{c}{4} |n|} e^{- \frac{{\tilde{c}}}{4} \delta _{\omega ,-\omega '} L_{2}}, \end{aligned}$$

which is the claimed bound (4.52) on scale h-1.

##### Proof of Proposition 4.7

To begin, it is useful to first introduce the *unrenormalized value* $\theta _{\text {nr}}$, as follows:

4.87 $$\begin{aligned} \theta _{\text {nr}}({\boldsymbol{q}}) = \Big (\prod _{v \in \mathscr {N}_{\text {r}}(\theta )} V_{v}\Big ) \Big (\prod _{v\in \mathscr {N}_{\text {nr}}(\theta )} {\mathfrak R}V^{(0)}_{n_{v}; \omega _{v},\omega '_{v}}\big ({\boldsymbol{q}} + {\boldsymbol{n}}'(v)\big ) \Big ) \Big (\prod _{e\in \mathscr {E}(\theta )} g^{(h_{e})}_{\omega _{e}}\big ({\boldsymbol{q}} + \tilde{\boldsymbol{n}}(e)\big )\Big ), \nonumber \\ \end{aligned}$$

where if e=(v,v+1), $\tilde{\boldsymbol{n}}(e) = {\boldsymbol{n}}(v)$. This expression is generated iterating (4.81) neglecting the ${\mathfrak R}$ operators acting on $\theta _{h+1}$. Let us first derive an estimate for the unrenormalized value, and then discuss the actual estimate for $\theta ({\boldsymbol{q}})$, taking into account the action of ${\mathfrak R}$.

Assume that the estimates for the running coupling constants (4.70) hold true on scales greater or equal than *h*, and recall the bound (4.81) for the single-scale propagator and the bound (4.45) for the kernels on scale 0. Then, we have:

4.88 $$\begin{aligned} | \theta _{\text {nr}}({\boldsymbol{q}}) | \le \Big (\prod _{v \in \mathscr {N}_{\text {r}}(\theta )} \gamma ^{h_{v}} |\nu _{\omega _{v},h_{v}}| \Big ) \Big (\prod _{v\in \mathscr {N}_{\text {nr}}(\theta )} C|\lambda | e^{-c|n_{v}|} e^{-\frac{{\tilde{c}}}{2} \delta _{\omega _{v},-\omega _{v}} L_{2}} \Big ) \Big (\prod _{e\in \mathscr {E}(\theta )} C\gamma ^{-h_{e}}\Big ). \nonumber \\ \end{aligned}$$

The sum over all chains implies a sum over all scale labels. Due to the last factor in Eq. (4.88), it is clear that the bound (4.88) cannot be used to prove the bound (4.52) for the effective potential on scale *h*.

In order to improve the situation, we have to take into account the action of the ${\mathfrak R}$ operator in (4.81), on a general $\theta _{h+1}({\boldsymbol{q}})$. The action is nontrivial only if $\theta _{h+1}({\boldsymbol{q}})$ is associated with a resonant cluster. To begin, observe that the estimate (4.88) can be rewritten as:

4.89 $$\begin{aligned} | \theta _{\text {nr}}({\boldsymbol{q}}) |\le &   \Big (\prod _{v \in \mathscr {N}_{\text {r}}(\theta )} \gamma ^{h_{v}} |\nu _{\omega _{v},h_{v}}| \Big ) \Big (\prod _{v\in \mathscr {N}_{\text {nr}}(\theta )} C|\lambda | e^{-c|n_{v}|} e^{-\frac{{\tilde{c}}}{2} \delta _{\omega _{v},-\omega _{v}} L_{2}} \Big ) \nonumber \\  &   \cdot \Big (\prod _{T\in \mathscr {C}_{0}(\theta )} \gamma ^{-h_{T} (s_{T} - 1)}\Big ). \end{aligned}$$

Since the operator ${\mathfrak R}$ extracts the second-order Taylor remainder from the value of the subgraph associated with a cluster *T*, it introduces a factor $\gamma ^{2(h^{\text {ext}}_{T} - h_{T})}$ in the estimates, see e.g. [8, 24]: the factor $\gamma ^{2 h^{\text {ext}}_{T}}$ is the estimate for the external momenta to the cluster, while the factor $\gamma ^{-2 h_{T}}$ takes into account the action of the second derivative. Thus, the action of ${\mathfrak R}$ on all resonant clusters introduces the following factor in the bound for the chain:

4.90 $$\begin{aligned} \prod _{T \in \mathscr {C}_{\text {r}}(\theta )} \gamma ^{2(h^{\text {ext}}_{T} - h_{T})}. \end{aligned}$$

The bound for the renormalized chain is:

4.91 $$\begin{aligned} \begin{aligned} | \theta ({\boldsymbol{q}}) |&\le C^{|\mathscr {N}(\theta )|} \Big (\prod _{v \in \mathscr {N}_{\text {r}}(\theta )} \gamma ^{h_{v}} |\nu _{\omega _{v},h_{v}}| \Big ) \Big (\prod _{v\in \mathscr {N}_{\text {nr}}(\theta )} C|\lambda | e^{-c|n_{v}|} e^{-\frac{{\tilde{c}}}{2} \delta _{\omega _{v},-\omega _{v}} L_{2}} \Big ) \\&\qquad \cdot \Big (\prod _{T\in \mathscr {C}_{0}(\theta )} \gamma ^{-h_{T} (s_{T} - 1)}\Big ) \Big (\prod _{T\in \mathscr {C}_{\text {r}}(\theta )} \gamma ^{2 (h^{\text {ext}}_{T} - h_{T})}\Big ), \end{aligned} \end{aligned}$$

where the factor $C^{|\mathscr {N}(\theta )|}$ also bounds the number of ways to distribute the derivatives coming from the ${\mathfrak R}$ operators. To better understand the right-hand side of (4.91), let us write:

4.92 $$\begin{aligned} \prod _{T\in \mathscr {C}_{0}(\theta )} \gamma ^{-h_{T} (s_{T} - 1)} = \Big (\prod _{T\in \mathscr {C}_{0}(\theta )} \gamma ^{-h_{T} (s^{\text {r}}_{T} - 1)}\Big ) \Big (\prod _{T\in \mathscr {C}_{0}(\theta )} \gamma ^{-h_{T} s^{\text {nr}}_{T}}\Big ), \end{aligned}$$

where we recall that $s_{T}^{\text {r}}$ is the number of maximal resonant clusters or vertices contained in *T*, and $s_{T}^{\text {nr}}$ is the number of non-resonant maximal clusters or vertices contained in *T*. We will control the first factor in the right-hand side of (4.92) using part of the last factor in (4.91). Calling $s^{\text {c,r}}_{T}$ the number of maximal resonant clusters contained in *T*:

4.93 $$\begin{aligned} \begin{aligned} \Big (\prod _{T\in \mathscr {C}_{0}(\theta )} \gamma ^{-h_{T} (s^{\text {r}}_{T} - 1)}\Big ) \Big (\prod _{T\in \mathscr {C}_{\text {r}}(\theta )} \gamma ^{(h^{\text {ext}}_{T} - h_{T})}\Big )&\le \gamma ^{h} \Big (\prod _{T\in \mathscr {C}_{0}(\theta )} \gamma ^{-h_{T} s^{\text {r}}_{T}}\Big ) \Big (\prod _{T\in \mathscr {C}_{\text {r}}(\theta )} \gamma ^{h^{\text {ext}}_{T}}\Big ) \\&= \gamma ^{h} \Big (\prod _{T\in \mathscr {C}_{0}(\theta )} \gamma ^{-h_{T} s^{\text {r}}_{T}}\Big ) \Big (\prod _{T\in \mathscr {C}_{0}(\theta )} \gamma ^{h_{T} s^{\text {c,r}}_{T}}\Big ) \\&= \gamma ^{h} \prod _{v\in \mathscr {N}_{\text {r}}(\theta )} \gamma ^{-h_{v}}. \end{aligned} \nonumber \\ \end{aligned}$$

Thus, plugging this bound in (4.91) we obtain:

4.94 $$\begin{aligned} \begin{aligned} | \theta ({\boldsymbol{q}}) |&\le \gamma ^{h} K^{|\mathscr {N}(\theta )|} |\lambda |^{|\mathscr {N}_{\text {nr}}(\theta )|} \Big (\prod _{v \in \mathscr {N}_{\text {r}}(\theta )} |\nu _{\omega _{v},h_{v}}|\Big ) \\&\quad \cdot \Big (\prod _{v\in \mathscr {N}_{\text {nr}}(\theta )} e^{-c|n_{v}|} e^{-\frac{{\tilde{c}}}{2} \delta _{\omega _{v},-\omega _{v}} L_{2}} \Big ) \Big (\prod _{T\in \mathscr {C}_{0}(\theta )} \gamma ^{-h_{T} s^{\text {nr}}_{T}}\Big ) \Big (\prod _{T\in \mathscr {C}_{\text {r}}(\theta )} \gamma ^{ (h^{\text {ext}}_{T} - h_{T})}\Big ). \end{aligned} \nonumber \\ \end{aligned}$$

Hence, thanks to the renormalization procedure, the sum over the scale labels of the resonant clusters can be controlled by the last factor in (4.94).

##### Remark 4.8

The above estimate makes it clear why the localization operation is defined up to order 1 in the Taylor expansion in the momenta, recall Definition 4.4: it is essential that the prefactor of $(h^{\text {ext}}_{T} - h_{T})$ in $\gamma ^{2 (h^{\text {ext}}_{T} - h_{T})}$ in the bound (4.91) is bigger than 1, in order to control the sum over the scale labels of the resonant clusters, as we did above.

We are left with discussing the dependence on the scale labels associated with non-resonant clusters. Here, a key role will be played by the approximate Diophantine property (2.9); it will allow us to use part of the second factor in the second line of (4.94) to overcompensate the bad third factor in (4.94). Let us define $\mathscr {N}_{\text {nr}}^{\text {d}}(\theta )$ as the set of diagonal non-resonant nodes, that is with $\omega _{v} = \omega '_{v}$ and $n_{v}\ne 0$, and $\mathscr {N}_{\text {nr}}^{\text {od}}(\theta )$ as the set of off-diagonal non-resonant nodes, that is with $\omega _{v} \ne \omega '_{v}$. We start by writing:

4.95 $$\begin{aligned} \Big (\prod _{v\in \mathscr {N}_{\text {nr}}^{\text {od}}(\theta )}e^{-\frac{{\tilde{c}}}{4} L_{2}}\Big ) \Big (\prod _{v\in \mathscr {N}^{\text {d}}_{\text {nr}}(\theta )} e^{-\frac{c}{2}|n_{v}|}\Big ) = \prod _{v\in \mathscr {N}_{\text {nr}}(\theta )} e^{-a_{v}} \end{aligned}$$

with

4.96 $$\begin{aligned} a_{v}:= (c/2) |n_{v}| \quad \text {if } v\in \mathscr {N}_{\text {nr}}^{\text {d}}(\theta ) \quad \text {and}\quad a_{v} = ({\tilde{c}} /4) L_{2}\quad \text {if } v\in \mathscr {N}_{\text {nr}}^{\text {od}}(\theta ). \end{aligned}$$

Then,

4.97 $$\begin{aligned} \begin{aligned} \prod _{v\in \mathscr {N}_{\text {nr}}(\theta )} e^{-a_{v}}&= \prod _{v\in \mathscr {N}_{\text {nr}}(\theta )} \prod _{k = -\infty }^{0} e^{-2^{k} a_{v}} \\&\le \prod _{k = -\infty }^{0} \prod _{\begin{array}{c} v\in \mathscr {N}_{\text {nr}}(\theta ) \\ \text {s.t.}\,v\in T\, \text {with}\, h^{\text {ext}}_{T} = k \end{array}} e^{-2^{h^{\text {ext}}_{T}} \!\! a_{v} } \le \prod _{T \in \theta \cup \mathscr {C}_{\text {nr}}(\theta ) \cup \mathscr {N}_{\text {nr}}(\theta )} e^{-2^{h^{\text {ext}}_{T}}\!\! a_{T}} \end{aligned} \end{aligned}$$

where we set $a_{T}:= \sum _{v\in T} a_{v}$. In the last inequality of (4.97), the product runs over clusters and endpoints, and with a slight abuse of notation we use the symbol *T* to denote the elements of both sets. The presence of the non-resonant endpoints in the last product is due to the contributions of the clusters *T* with external scale zero, which are by definition endpoints. Given *T* in the last product in (4.97), suppose that *T* contains at least one non-resonant off-diagonal node. Then:

4.98 $$\begin{aligned} e^{-2^{h^{\text {ext}}_{T}}a_{T}} \le e^{-2^{h^{\text {ext}}_{T}} \frac{{\tilde{c}}}{4} L_{2}} \le e^{-2^{h^{\text {ext}}_{T}} {\tilde{C}} \gamma ^{-h^{\text {ext}}_{T}}} \end{aligned}$$

where we used that $\kappa L_{2} \ge \beta \ge C\gamma ^{-h^{\text {ext}}_{T}}$. Alternatively, suppose that *T* does not contain any non-resonant off-diagonal node. Observe that the difference between the incoming and the outgoing momenta of the cluster/node *T* is bounded by $C \gamma ^{h^{\text {ext}}_{T}}$. Since no off-diagonal node is present, we derive the following constraint for the sum over the momentum shifts associated with the diagonal non-resonant nodes (recall that α is the frequency of the quasi-periodic potential, Eq. (2.7)):

4.99 $$\begin{aligned} \bigg | \sum _{v\in T \cap \mathscr {N}^{\text {d}}_{\text {nr}}(\theta )} n_{v} \alpha \bigg |_{\mathbb {T}} \le C\gamma ^{h^{\text {ext}}_{T}}. \end{aligned}$$

Let $n_{T}^{\text {d}}:= \sum _{v\in T \cap \mathscr {N}_{\text {nr}}^{\text {d}}} n_{v}$. Suppose that the cluster *T* is non-resonant, that is $n_{T}^{\text {d}} \ne 0$. Suppose that $| n^{\text {d}}_{T} | \le L_{1} / 2$. Then, by the Diophantine condition (2.9) we have:

4.100 $$\begin{aligned} \frac{c}{| n^{\text {d}}_{T} |^{\tau }} \le \bigg | \sum _{v\in T \cap \mathscr {N}^{\text {d}}_{\text {nr}}(\theta )} n_{v} \alpha \bigg |_{\mathbb {T}} \le C \gamma ^{h^{\text {ext}}_{T}}, \end{aligned}$$

or equivalently $|n^{\text {d}}_{T}| \ge K\gamma ^{-h^{\text {ext}}_{T} / \tau }$. Therefore, we get:

4.101 $$\begin{aligned} e^{-2^{h^{\text {ext}}_{T}}a_{T}} \le e^{-2^{h^{\text {ext}}_{T}} K \gamma ^{-h^{\text {ext}}_{T} / \tau }}. \end{aligned}$$

Finally, suppose that $| n^{\text {d}}_{T} | > L_{1} / 2$. In this case, we simply use that:

4.102 $$\begin{aligned} \begin{aligned} e^{-2^{h^{\text {ext}}_{T}} a_{T}}&\le e^{-2^{h^{\text {ext}}_{T}}\frac{c}{8} L_{1}} \le e^{-2^{h^{\text {ext}}_{T}}C \gamma ^{-h_{\beta }}} \le e^{-2^{h^{\text {ext}}_{T}}C \gamma ^{-h^{\text {ext}}_{T}}} \end{aligned} \end{aligned}$$

where in the second inequality we used that $\kappa L_{1} \ge \beta$, and in the last we used that $h^{\text {ext}}_{T} \ge h_{\beta }$. Putting together (4.98), (4.101), (4.102), we have that, in all cases:

4.103 $$\begin{aligned} e^{-2^{h^{\text {ext}}_{T}} a_{T}} \le e^{-2^{h^{\text {ext}}_{T}} C \gamma ^{-h^{\text {ext}}_{T} / \tau }}. \end{aligned}$$

Using this estimate in (4.97), we find, for $\mathscr {C}_{0;\text {nr}}(\theta ) := \mathscr {C}_{\text {nr}}(\theta ) \cup \theta$ if θ is non-resonant, and $\mathscr {C}_{0;\text {nr}}(\theta ) := \mathscr {C}_{\text {nr}}(\theta )$ otherwise:

4.104 $$\begin{aligned} \prod _{v\in \mathscr {N}_{\text {nr}}(\theta )} e^{-a_{v}}\le &   \prod _{T \in \mathscr {C}_{0;\text {nr}}(\theta ) \cup \mathscr {N}_{\text {nr}}(\theta )} e^{-2^{h^{\text {ext}}_{T}} C \gamma ^{-h^{\text {ext}}_{T} / \tau }}\nonumber \\\le &   e^{- \varepsilon (\theta )2^{h}\widetilde{C} \gamma ^{-h / \tau }} \prod _{T\in \mathscr {C}_{0}(\theta )} e^{- s^{\text {nr}}_{T} 2^{h_{T}}\widetilde{C} \gamma ^{-h_{T} / \tau }}. \end{aligned}$$

where $\varepsilon (\theta ) = 0$ if θ is resonant and $\varepsilon (\theta ) = 1$ otherwise. We will now use this bound in (4.94). We have:

4.105 $$\begin{aligned} \begin{aligned} | \theta ({\boldsymbol{q}}) |&\le \gamma ^{h} e^{- \varepsilon (\theta )2^{h}\widetilde{C} \gamma ^{-h / \tau }} K^{|\mathscr {N}(\theta )|} |\lambda |^{|\mathscr {N}_{\text {nr}}(\theta )|} \Big (\prod _{v \in \mathscr {N}_{\text {r}}(\theta )} |\nu _{\omega _{v},h_{v}}| \Big ) \\&\quad \cdot \Big (\prod _{v\in \mathscr {N}_{\text {nr}}(\theta )} e^{-\frac{c}{2} |n_{v}|}e^{-\frac{{\tilde{c}}}{4} \delta _{\omega _{v},-\omega _{v}} L_{2}} \Big ) \\&\quad \cdot \Big (\prod _{T\in \mathscr {C}_{0}(\theta )} \gamma ^{-h_{T} s^{\text {nr}}_{T}} e^{- s^{\text {nr}}_{T} 2^{h_{T}}\widetilde{C} \gamma ^{-h_{T} / \tau }}\Big ) \Big (\prod _{T\in \mathscr {C}_{\text {r}}(\theta )} \gamma ^{ (h^{\text {ext}}_{T} - h_{T})}\Big ). \end{aligned} \end{aligned}$$

Next, the factor in the third product can be estimated as $C \gamma ^{h_{T} s_{T}^{\text {nr}}}$, for a constant *C* that only depends on γ. Therefore, since the number of clusters in $\mathscr {C}(\theta )$ is bounded by $|\mathscr {N}(\theta )|$, we have:

4.106 $$\begin{aligned} \begin{aligned} | \theta ({\boldsymbol{q}}) |&\le \gamma ^{h} e^{- \varepsilon (\theta )2^{h}\widetilde{C} \gamma ^{-h / \tau }} \widetilde{K}^{|\mathscr {N}(\theta )|} |\lambda |^{|\mathscr {N}_{\text {nr}}(\theta )|} \Big (\prod _{v \in \mathscr {N}_{\text {r}}(\theta )} |\nu _{\omega _{v},h_{v}}|\Big ) \\&\quad \cdot \Big (\prod _{v\in \mathscr {N}_{\text {nr}}(\theta )} e^{-\frac{c}{2} |n_{v}|}e^{-\frac{{\tilde{c}}}{4} \delta _{\omega _{v},-\omega _{v}} L_{2}} \Big ) \cdot \Big (\prod _{T \in \mathscr {C}(\theta )} \gamma ^{ (h^{\text {ext}}_{T} - h_{T})}\Big ), \end{aligned} \end{aligned}$$

where we used that:

4.107 $$\begin{aligned} \prod _{T\in \mathscr {C}_{0}(\theta )} \gamma ^{h_{T} s^{\text {nr}}_{T}} = \prod _{T\in \mathscr {C}_{\text {nr}}(\theta ) \cup \mathscr {N}_{\text {nr}}(\theta )} \gamma ^{h^{\text {ext}}_{T}} \le \prod _{T\in \mathscr {C}_{\text {nr}}(\theta ) \cup \mathscr {N}_{\text {nr}}(\theta )} \gamma ^{h^{\text {ext}}_{T} - h_{T}}, \end{aligned}$$

recall that the scale of the non-resonant nodes is zero. This concludes the proof of (4.84), for $n_{0} = n_{1} = 0$. The bound for the derivatives of $\theta ({\boldsymbol{q}})$ can be proved in the same way, using the estimates (4.73) for the derivatives of the propagator. We omit the details. □

#### The Flow of the Running Coupling Constants

To conclude the proof of Proposition 4.2, in this section we will control the flow of the running coupling constants. In particular, our goal will be to show the bounds (4.70) on all scales greater or equal than *h*. This is the content of the next proposition.

##### Proposition 4.9

(The flow of the beta function). There exists a choice of $\nu _{\omega } = O(\lambda )$ in $\mathbb {R}$ such that the estimates (4.70) hold true on all scales.

##### Proof

We will show how to prove the bounds for the marginal terms, assuming the validity of the bound for $\nu _{h,\omega }$, and the validity of the same estimates on scales strictly greater than *h*. The proof of the bound for $\nu _{h,\omega }$ can be done via a standard fixed point argument which has been carried out in many previous papers, see e.g. Section 6.4.2 of [26] for a recent application (in a more difficult context). Recall the flow equation (4.64)–(4.68); the beta function can be represented in terms of chain graphs, as follows:

4.108 $$\begin{aligned} \beta ^{v}_{\nu ,\omega ,h+1} = i^{\delta _{\nu },0}\sum _{s \ge 1} \frac{1}{s!} \sum _{\theta \in {\mathscr {T}}_{n;s, \omega ,\omega }^{(h+1)}} \text {d}_{\nu } \theta ({\boldsymbol{0}}_{\beta }). \end{aligned}$$

The argument of the sum could be estimated with (4.84). This however would produce a bound that is not summable in *h*, and it would not allow to check the desired estimates on scale *h*. To obtain a better estimate, we observe that if all nodes of the chain θ contributing to the beta function are resonant, i.e. associated with $n_{v} = 0$, then the quasi-momentum flowing on every propagator on the chain is either ${\boldsymbol{0}}_{\beta }^{+}$ or ${\boldsymbol{0}}_{\beta }^{-}$. Since these quasi-momenta do not belong to the support of the propagators on scales h+1 with $h\ge h_{\beta }$, the corresponding chain graph is vanishing.

Thus, all the chain graphs contributing to the beta function must have at least one non-resonant node. In fact, since the outgoing and the incoming momentum of the whole chain have to be the same, there must be at least two non-resonant nodes. Also, the chain graphs contributing to $\beta ^{v}_{\nu ,\omega ,h+1}$ must have at least one propagator on scale h+1; let us denote by $(v_{*}, v_{*}+1)$ the two nodes connected by $g^{(h+1)}_{\omega _{v_{*}}}$. By the Kirchhoff rule, the momentum flowing on this propagator is ${\boldsymbol{n}}(v_{*})$, and ${\boldsymbol{n}}(v_{*}) \ne 0$ otherwise the propagator is vanishing, by the support of the single-scale cutoff in the definition of $g^{(h+1)}$.

We write:

4.109 $$\begin{aligned} \begin{aligned} \sum _{\theta \in {\mathscr {T}}_{n;s, \omega ,\omega }^{(h+1)}} \text {d}_{\nu } \theta ({\boldsymbol{0}}_{\beta }) = \sum _{j=2}^{s-1}\sum _{\begin{array}{c} \theta \in {\mathscr {T}}_{n;s, \omega ,\omega }^{(h+1)} \\ v_{*} = j \end{array}} \text {d}_{\nu }\theta ({\boldsymbol{0}}_{\beta }), \end{aligned} \end{aligned}$$

and we further split:

4.110 $$\begin{aligned} \sum _{\begin{array}{c} \theta \in {\mathscr {T}}_{n;s, \omega ,\omega }^{(h+1)} \\ v_{*} = j \end{array}} \text {d}_{\nu }\theta ({\boldsymbol{0}}_{\beta }) = \text {A} + \text {B}, \end{aligned}$$

where $\text {A}$ takes into account chains with no off-diagonal non-resonant vertex before $v_{*}$, and $\text {B}$ takes into account the contributions where we have at least one off-diagonal non-resonant vertex before $v_{*}$. Let us estimate $\text {B}$. Using the bound (4.84), we easily get:

4.111 $$\begin{aligned} | \text {B} | \le C s! |\lambda |^{s} e^{-\frac{{\tilde{c}}}{4} L_{2}}. \end{aligned}$$

Observe that s≥2, since there must be at least two off-diagonal vertices. Consider now the term ${\text{ A }}$. Here, there are no off-diagonal non-resonant vertices before $v_{*}$. Since all non-resonant vertices before $v_{*}$ are diagonal, by the support of the propagator on scale h+1, $\Big | \sum _{v\le v_{*}} n_{v} \alpha \Big | \le C\gamma ^{h}$. Suppose that $\big |\sum _{v\le v_{*}} n_{v}\big | \le L_{1} / 2$. Since $\sum _{v\le v_{*}} n_{v} \ne 0$ (otherwise the propagator $g^{(h+1)}$ connecting $v_{*}$ to $v_{*} + 1$ is vanishing), by the Diophantine condition (2.9) we have:

4.112 $$\begin{aligned} \Big | \sum _{v\le v_{*}} n_{v} \Big | \ge K\gamma ^{-h / \tau }. \end{aligned}$$

This will introduce an extra small factor in the estimate for the beta function. To see this, we estimate the first product in Eq. (4.84) as:

4.113 $$\begin{aligned} \Big ( \prod _{v\in \mathscr {N}(\theta )} e^{-\frac{c}{2}|n_{v}|} \Big )\le &   \Big ( \prod _{\begin{array}{c} v\in \mathscr {N}(\theta ) \\ v\le v_{*} \end{array}} e^{-\frac{c}{4}|n_{v}|} \Big ) \Big ( \prod _{v\in \mathscr {N}(\theta )} e^{-\frac{c}{4}|n_{v}|} \Big ) \nonumber \\\le &   e^{-\frac{c}{4} K \gamma ^{-h/\tau }} \Big ( \prod _{v\in \mathscr {N}(\theta )} e^{-\frac{c}{4}|n_{v}|} \Big ), \end{aligned}$$

where the second bound follows from (4.112). If $\Big |\sum _{v\le v_{*}} n_{v}\Big | > L_{1} / 2$ we proceed as after (4.107), and also in this case we end up with the estimate (4.113). We then get:

4.114 $$\begin{aligned} |\text {A}| \le Cs! |\lambda |^{s}e^{-\frac{c}{4} K \gamma ^{-h/\tau }} \end{aligned}$$

Also here s≥2, since there must be at least two non-resonant vertices. We are now ready to prove the estimate for the beta function. Putting together (4.110), (4.111), (4.114) we obtain, recalling that $L_{2} \ge C\beta \ge C\gamma ^{-h}$, and using that s≥2:

4.115 $$\begin{aligned} | \beta ^{v}_{\nu , \omega ,h} | \le K C_{m}|\lambda |^{2} \gamma ^{m h},\qquad \forall m\in \mathbb {N}. \end{aligned}$$

This concludes the proof of Proposition 4.9. □

The control of the flow of the running coupling constants completes the construction of the effective potential on all scales, and concludes the proof of Proposition 4.2. To conclude the section, we observe that the effective potential admits improved estimates.

##### Proposition 4.10

(Improved bound for the effective potential). There exists 0<ξ<1 such that:

4.116 $$\begin{aligned} | \text {d}_{0}^{n_{0}} \text {d}_{1}^{n_{1}} V^{(h)}_{n;\omega ,\omega '}({\boldsymbol{q}})| \le C_{n_{0},n_{1}}|\lambda | \gamma ^{\xi h}\gamma ^{h(1 - n_{0} - n_{1})} e^{-\frac{c}{4} |n|} e^{- \frac{{\tilde{c}}}{4} \delta _{\omega ,-\omega '} L_{2}}. \end{aligned}$$

##### Remark 4.11

That is, the bound (4.116) improves the estimate (4.52) by a gain factor $\gamma ^{\xi h}$.

##### Proof

The proof is based on a simple adaptation of the chain expansion discussed above; let us sketch it. Consider the graphs with only resonant nodes (that is, $n_{v} = 0$ and $\omega _{v} = \omega _{v}'$), and recall the bound $|\nu _{h,\omega }| \le C|\lambda | \gamma ^{\xi h}$. The factor $\gamma ^{\xi h}$ in the estimate for the running coupling constants allows us to obtain the desired dimensional gain in the estimate for the fully resonant graphs. Consider now the graphs with at least one non-resonant node (there has to be at least one non-resonant node if n≠0). Non-resonant nodes are on scale 0: thus, the cluster stucture of the graphs contributing to the effective potential reaches scale 0. For this reason, we can rewrite the last factor in (4.84) as, for 0<ξ<1:

4.117 $$\begin{aligned} \begin{aligned} \prod _{T\in \mathscr {C}(\theta ) \cup \mathscr {N}_{\text {nr}}(\theta )} \gamma ^{ (h^{\text {ext}}_{T} - h_{T})}&= \Big (\prod _{T\in \mathscr {C}(\theta ) \cup \mathscr {N}_{\text {nr}}(\theta )} \gamma ^{ \xi (h^{\text {ext}}_{T} - h_{T})}\Big ) \Big ( \prod _{T\in \mathscr {C}(\theta ) \cup \mathscr {N}_{\text {nr}}(\theta )} \gamma ^{ (1-\xi )(h^{\text {ext}}_{T} - h_{T})} \Big ) \\&\le \gamma ^{\xi h} \Big ( \prod _{T\in \mathscr {C}(\theta ) \cup \mathscr {N}_{\text {nr}}(\theta )} \gamma ^{ (1-\xi )(h^{\text {ext}}_{T} - h_{T})} \Big ), \end{aligned} \end{aligned}$$

where we used that the external scale of the deepest cluster is *h* and the internal scale of the highest cluster is 0, and we estimated the first factor as a telescopic product. This allows us to extract a factor $\gamma ^{\xi h}$ also for the graphs containing at least one non-resonant node; the remaining product allows us to control the sum over the scale labels, since 1-ξ>0. Finally, if $\omega \ne \omega '$ the graph must have at least one off-diagonal node; the exponential decay in $L_{2}$ in (4.116) follows from (4.84). This concludes the proof. □

Our next task will be to compute the two-point correlation function of the model; this will be discussed in the next section, via an adaptation of the multi-scale analysis used to construct the effective potential.

### The Two-point Function

In this section we shall discuss how to adapt the previous multiscale analysis, to compute the two point correlation function (2.24). Being the state quasi-free, the computation of the two-point correlation function allows us to determine all correlation functions of the model via Wick’s rule.

#### The Generating Functional of Correlations

Let us define the configuration space Grassmann field as:

4.118 $$\begin{aligned} \psi ^{\pm }_{\vec {\boldsymbol{x}},\sigma }:= \frac{1}{L\beta } \sum _{{\boldsymbol{k}} \in \mathscr {D}_{N, L,\beta }} e^{\mp i{\boldsymbol{x}}\cdot {\boldsymbol{k}}} \psi ^{\pm }_{{\boldsymbol{k}},x_2,\sigma }, \end{aligned}$$

with $\mathscr {D}_{N, L,\beta }$ as in Eq. (4.3). Let us introduce the generating functional of correlations,

4.119 $$\begin{aligned} e^{\mathcal {W}_{N}(\phi )}:= &   \int \mathbb {P}_{N}(d\psi ) \,e^{{\mathcal V_{N}( \psi ) + (\psi , \phi )}},\quad \nonumber \\ (\psi , \phi ):= &   \sum _{\sigma = 1}^{M} \sum _{\vec { x} \in \Lambda _{L}} \int _{0}^{\beta } dx_0\, (\phi ^+_{\vec {\boldsymbol{x}},\sigma } \psi _{\vec {\boldsymbol{x}},\sigma }^- + \psi ^+_{\vec {\boldsymbol{x}},\sigma }\phi ^-_{\vec {\boldsymbol{x}},\sigma }), \end{aligned}$$

where $\phi ^{\pm }$ is an external Grassmann field, which can also be written as in (4.118) in terms of suitable momentum space fields $\phi ^{\pm }_{{\boldsymbol{k}}, x_{2}, \sigma }$. Then, the two-point correlation function (at non-coinciding space-time points) can be represented as:

4.120 $$\begin{aligned} \big \langle \textbf{T}\, \gamma _{x_0}(a_{{\vec x},\sigma }); \gamma _{y_0}(a_{\vec y,\zeta }^{*})\big \rangle _{\beta ,\mu ,L} = \lim _{N\rightarrow \infty } \frac{\partial ^{2}}{\partial \phi ^{+}_{\vec {\boldsymbol{x}}, \sigma } \partial \phi ^{-}_{\vec {\boldsymbol{y}}, \zeta }} \mathcal {W}_{N}(\phi )\Big |_{\phi = 0}. \end{aligned}$$

Our goal will be to compute the generating functional of correlations, adapting the multiscale analysis developed for the partition function. Proceeding as in Sect. 4.1, we end up with:

4.121 $$\begin{aligned} \exp \big (\mathcal {W}_{N}(\phi )\big ) = z_{>0}(\phi ) \int \mathbb {P}_{(\le 0)}(d\psi ^{(\le 0)}) \exp \big (\mathcal {V}^{(0)}(\psi ^{(\le 0)}) + \mathcal {W}^{(0)}(\psi ^{(\le 0)}, \phi )\big ), \nonumber \\ \end{aligned}$$

where:

(i) the overall prefactor is:
  4.122 $$\begin{aligned} \begin{aligned} z_{>0}(\phi )&= {\tilde{z}}_{\textrm{b}} \exp \big ( \phi ^{+}, W^{(>0)} \phi ^{-} \big ) \\ \big ( \phi ^{+}, W^{(>0)} \phi ^{-} \big )&= \int _{0}^{\beta } dx_{0} dy_{0}\, \sum _{\vec x, \vec y \in \Lambda _{L}} \sum _{\sigma , \zeta } \phi ^{+}_{\vec {\boldsymbol{x}}, \sigma } W^{(>0)}_{\sigma ,\zeta }(\vec {\boldsymbol{x}}, \vec {\boldsymbol{y}}) \phi ^{-}_{\vec {\boldsymbol{y}}, \zeta } \end{aligned} \end{aligned}$$
  and the kernels satisfy $\big | W^{(>0)}_{\sigma ,\zeta }(\vec {\boldsymbol{x}}, \vec {\boldsymbol{y}}) \big | \le C e^{-c\Vert \vec {\boldsymbol{x}} - \vec {\boldsymbol{y}} \Vert }$.
(ii) The effective potential $\mathcal {W}^{(\le 0)}(\psi ^{(\le 0)}, \phi )$ has the form:
  4.123 $$\begin{aligned} \begin{aligned}&\mathcal W^{(0)}( \psi ^{(\le 0)},\phi ) \\&= \frac{1}{\beta L_{1}} \sum _{\begin{array}{c} n, {\boldsymbol{k}} \\ x_{2},\omega ,\sigma \end{array}} \Big ( \phi ^+_{{\boldsymbol{k}}, x_{2}, \sigma } W^{(0)}_{\phi \psi ;n,\omega ,\sigma } \big (\boldsymbol{q}, x_{2}\big ) \psi ^{(\le 0)-}_{ \boldsymbol{q} + n{\boldsymbol{\alpha }}, \omega } + \psi ^{(\le 0)+}_{\boldsymbol{q}, \omega } W^{(0)}_{\psi \phi ;n,\omega ,\sigma } \big (\boldsymbol{q}, x_{2}\big ) \phi ^{-}_{ {\boldsymbol{k}} + n{\boldsymbol{\alpha }}, x_{2},\sigma }\Big ) \end{aligned} \nonumber \\ \end{aligned}$$
  where we recall $\boldsymbol{q}\equiv \boldsymbol{q}(\boldsymbol{k})=\boldsymbol{k}-\boldsymbol{k}_F^\omega$, as in (4.38). The two kernels are related by:
  4.124 $$\begin{aligned} \overline{W^{(0)}_{\phi \psi ;n,\omega ,\sigma } ( (q_{0}, q_{1}), x_{2})} = W^{(0)}_{\psi \phi ;-n,\omega ,\sigma } \big ( (-q_{0}, q_{1} + n\alpha ), x_{2}\big ), \end{aligned}$$
  and they satisfy the estimate:
  4.125 $$\begin{aligned} \Big | \text {d}_{q_{0}}^{n_{0}} \text {d}_{q_{1}}^{n_{1}} W^{(0)}_{\phi \psi ;n,\omega ,\sigma } ( {\boldsymbol{q}}, x_{2}) \Big | \le C_{n_{0}, n_{1}} |\lambda |^{\delta _{n\ne 0}} e^{-c|n|} e^{-{\tilde{c}} |x_{2}|_{\omega }}, \end{aligned}$$
  with $|x_{2}|_{\omega }$ defined in (3.2).

Next, we set-up a multiscale analysis, as we did for the effective potential. Suppose that the generating functional of correlations can be written as:

4.126 $$\begin{aligned} \exp \big (\mathcal {W}_{N}(\phi )\big )= &   z_{(>h)}(\phi ) \int \mathbb {P}_{(\le h)}(d\psi ^{(\le h)}) \exp \big (\mathcal {V}^{(h)}(\psi ^{(\le h)})\nonumber \\  &   + \mathcal {W}^{(h)}(\psi ^{(\le h)}, \phi )\big ), \end{aligned}$$

for suitable $z_{(>h)}(\cdot )$, $\mathcal {W}^{(h)}(\cdot )$, similarly to (4.122), (4.123). In particular, we assume that:

4.127 $$\begin{aligned} z_{(>h)}(\phi ) = z_{(>h)} \exp (\phi ^{+}, W^{(>h)} \phi ^{-}), \end{aligned}$$

with $z_{(>h)}$ as in Sect. 4.2.3, and:

4.128 $$\begin{aligned} (\phi ^{+}, W^{(>h)} \phi ^{-})= &   \sum _{j=h+1}^{0}\frac{1}{\beta L_{1}} \sum _{n, \boldsymbol{k}} \phi ^{+}_{\boldsymbol{k}, x_{2},\sigma } W_{\phi \phi ;n,\sigma ,\zeta }^{(j)}({\boldsymbol{k}}; x_{2}, y_{2}) \phi ^{-}_{\boldsymbol{k} + n{\boldsymbol{\alpha }}, y_{2},\zeta }\nonumber \\  &   + (\phi ^{+}, W^{(>0)} \phi ^{-}),\nonumber \\ \end{aligned}$$

for suitable kernels $W^{(k)}$ to be determined inductively. To set up the integration of the single-scale, we proceed as in Sect. 4.2.3. We have:

4.129 $$\begin{aligned} \exp \big (\mathcal {W}_{N}(\phi )\big ) = z_{(>h)}(\phi ) {\tilde{z}}_{h} \int {\widetilde{P}}_{(\le h)}(d\psi ^{(\le h)}) \exp \big (\widetilde{ \mathcal {V}}^{(h)}(\psi ^{(\le h)}) + \mathcal {W}^{(h)}(\psi ^{(\le h)}, \phi )\big ),\nonumber \\ \end{aligned}$$

where, recalling that $s_{\omega \omega '} = {\boldsymbol{k}}_{F}^{\omega } - {\boldsymbol{k}}_{F}^{\omega '}$:

4.130 $$\begin{aligned} \widetilde{\mathcal {V}}^{(h)}(\psi ^{(\le h)}) = (\mathfrak L_{0} + \mathfrak R) \mathcal {V}^{(h)}(\psi ^{(\le h)}) = \frac{1}{\beta L_{1}} \sum _{n, {\boldsymbol{q}}}\sum _{\omega ,\omega '} \widetilde{V}_{n;\omega ,\omega '}^{(h)}({\boldsymbol{q}}) \psi _{{\boldsymbol{q}},\omega }^{(\le h)+} \psi _{{\boldsymbol{q}} + n {\boldsymbol{\alpha }} + s_{\omega \omega '},\omega '}^{(\le h)-}.\nonumber \\ \end{aligned}$$

Let us discuss the integration of the scale *h*. We have:

4.131 $$\begin{aligned} z_{(>h-1)}(\phi ) = z_{(>h)}(\phi ) {\tilde{z}}_{h} z_{h} \exp {(\phi ^{+}, W^{(h)} \phi ^{-})} \end{aligned}$$

where

4.132 $$\begin{aligned} (\phi ^{+}, W^{(h)} \phi ^{-}) = \frac{1}{\beta L_{1}} \sum _{n, {\boldsymbol{k}}} \phi ^{+}_{\boldsymbol{k}, x_{2},\sigma } W_{\phi \phi ;n,\sigma ,\zeta }^{(h)}({\boldsymbol{k}}; x_{2}, y_{2}) \phi ^{-}_{\boldsymbol{k} + n{\boldsymbol{\alpha }}, y_{2},\zeta }; \end{aligned}$$

the kernels satisfy the following recursion relation, recalling the notation ${\boldsymbol{q}} = {\boldsymbol{k}} - {\boldsymbol{k}}_{F}^{\omega }$:

4.133 $$\begin{aligned} \begin{aligned}&W_{\phi \phi ;n,\sigma ,\zeta }^{(h)} ({\boldsymbol{k}}; x_{2}, y_{2}) \\&\quad = \sum _{m,\omega } W^{(h)}_{\phi \psi ;m,\omega ,\sigma } ( {\boldsymbol{q}}, x_{2}) g^{(h)}_{\omega }({\boldsymbol{q}} + m{\boldsymbol{\alpha }}) W^{(h)}_{\psi \phi ;-m + n,\omega ,\zeta } ( {\boldsymbol{q}} + m{\boldsymbol{\alpha }}, y_{2}) \\&\qquad + \sum _{m,m'} \sum _{\omega ,\omega '}W^{(h)}_{\phi \psi ;m,\omega ,\sigma } ( {\boldsymbol{q}}, x_{2}) g^{(h)}_{\omega }({\boldsymbol{q}} + m{\boldsymbol{\alpha }}) V^{(h-1)}_{m';\omega ,\omega '}({\boldsymbol{q}} + m{\boldsymbol{\alpha }} ) \\&\qquad \cdot g^{(h)}_{\omega '}\big ({\boldsymbol{q}} + (m + m'){\boldsymbol{\alpha }} + s_{\omega \omega '}\big ) W^{(h)}_{\psi \phi ;-m - m' + n,\omega ',\zeta } \big ( {\boldsymbol{q}} + (m + m'){\boldsymbol{\alpha }} + s_{\omega \omega '}, y_{2}\big ), \end{aligned} \nonumber \\ \end{aligned}$$

and:

4.134 $$\begin{aligned}  &   W^{(h-1)}_{\phi \psi ;n,\omega ,\sigma } ( {\boldsymbol{q}}, x_{2}) \nonumber \\  &   \quad = W^{(h)}_{\phi \psi ;n,\omega ,\sigma } ( {\boldsymbol{q}}, x_{2}) + \sum _{m,\omega '} W^{(h)}_{\phi \psi ;m,\omega ',\sigma } ( {\boldsymbol{q}} + s_{\omega \omega '}, x_{2}) g^{(h)}_{\omega '}({\boldsymbol{q}} + m{\boldsymbol{\alpha }} + s_{\omega \omega '})\nonumber \\  &   \qquad \cdot V^{(h-1)}_{n-m;\omega ',\omega }({\boldsymbol{q}} + m {\boldsymbol{\alpha }} + s_{\omega \omega '}), \end{aligned}$$

with $V^{(h-1)}$ the effective potential on scale h-1. These identities are quite evident from the point of view of the chain expansion. See Appendix B for the proof of (4.133), (4.134), based on Grassmann integration by parts.

##### Remark 4.12

The kernels $W^{(h)}_{\phi \psi ;m,\omega }({\boldsymbol{q}})$, respectively $W^{(h)}_{\psi \phi ;-m+n,\omega }({\boldsymbol{q}} + m{\boldsymbol{\alpha }})$, are multiplied by fields $\psi ^{(\le h)-}_{{\boldsymbol{q}} + m{\boldsymbol{\alpha }}.\omega }$, respectively $\psi ^{(\le h)+}_{{\boldsymbol{q}} + m{\boldsymbol{\alpha }},\omega }$. These are defined as zero for ${\boldsymbol{q}} + m{\boldsymbol{\alpha }}$ outside of the support of the corresponding propagator. Nevertheless, the chain expansion for the kernels and the recursion relation (4.134) make sense for all ${\boldsymbol{q}}$ and for all *n*. In what follows, we will consider the $W^{(h)}_{\phi \psi ;n,\omega }({\boldsymbol{q}})$ as defined for all ${\boldsymbol{q}}$ and for all *n*.

The next proposition allows us to bound the solution of the recursion relation (4.134).

##### Proposition 4.13

(Flow of the $\phi \psi$-kernels). For |λ| small enough, the following is true:

4.135 $$\begin{aligned} \Big | W^{(h-1)}_{\phi \psi ;n,\omega ,\sigma } ( {\boldsymbol{q}}, x_{2}) \Big | \le C |\lambda |^{\delta _{n\ne 0}} e^{-\frac{c}{8}|n|} e^{- \frac{{\tilde{c}}}{32}|x_{2}|_{\omega }}. \end{aligned}$$

Also, for k≥h:

4.136 $$\begin{aligned} \big | W^{(h-1)}_{\phi \psi ;n,\omega ,\sigma } ( {\boldsymbol{q}}, x_{2}) - W^{(k)}_{\phi \psi ;n,\omega ,\sigma } ( {\boldsymbol{q}}, x_{2}) \big | \le K |\lambda |^{\delta _{n\ne 0}} \gamma ^{\xi k} e^{-\frac{c}{8}|n|} e^{- \frac{{\tilde{c}}}{32}|x_{2}|_{\omega }}. \end{aligned}$$

Furthermore, for $$n_{0} + n_{1} =1$$:

4.137 $$\begin{aligned} \big | \text {d}_{q_{0}}^{n_{0}} \text {d}_{q_{1}}^{n_{1}}W^{(h-1)}_{\phi \psi ;n,\omega ,\sigma } ( {\boldsymbol{q}}, x_{2}) \big | \le C \gamma ^{(\xi - 1)h } |\lambda |^{\delta _{n\ne 0}} e^{-\frac{c}{8}|n|} e^{- \frac{\tilde{c}}{32}|x_{2}|_{\omega }}. \end{aligned}$$

##### Remark 4.14

These bounds also allow to control the kernels $W^{(h-1)}_{\psi \phi }$, using that:

4.138 $$\begin{aligned} W^{(h-1)}_{\psi \phi ;n,\omega ,\sigma } ( (q_{0}, q_{1}), x_{2}) = \overline{W^{(h-1)}_{\phi \psi ;-n,\omega ,\sigma } \big ( (-q_{0}, q_{1} + n\alpha ), x_{2}\big )}. \end{aligned}$$

This identity is proved as (4.53), see Sect. 4.2.4.

##### Proof

Let us start by proving (4.135). To begin, recall the bound (4.116), for $L_{2} \ge C\beta$:

4.139 $$\begin{aligned} \big | V^{(h-1)}_{n-m;\omega ',\omega }({\boldsymbol{q}} + m {\boldsymbol{\alpha }})\big | \le C \gamma ^{h(1+\xi )} e^{-\frac{c}{4} |n-m|} |\lambda | e^{-\frac{{\tilde{c}}}{4} L_{2} \delta _{\omega ,-\omega '} }. \end{aligned}$$

We will proceed by induction. Let h<0, and assume that:

4.140 $$\begin{aligned} \big | W^{(h)}_{\phi \psi ;n,\omega ,\sigma } ( {\boldsymbol{q}}, x_{2}) \big | \le \sum ^{0}_{j > h} \gamma ^{\xi j} C|\lambda |^{\delta _{n\ne 0}} e^{-\frac{c}{8}|n|} e^{- \frac{{\tilde{c}}}{32}|x_{2}|_{\omega }}. \end{aligned}$$

Clearly, Eq. (4.140) also implies:

4.141 $$\begin{aligned} \big | W^{(h)}_{\phi \psi ;n,\omega ,\sigma } ( {\boldsymbol{q}}, x_{2}) \big | \le K|\lambda |^{\delta _{n\ne 0}} e^{-\frac{c}{8}|n|} e^{- \frac{\tilde{c}}{32}|x_{2}|_{\omega }}, \end{aligned}$$

which is (4.135). The assumption (4.140) is true for h=0, by (4.125). Our plan will be to check (4.140), using the recursion relation (4.134). From (4.134), we have:

4.142 $$\begin{aligned} \begin{aligned}&\big | W^{(h-1)}_{\phi \psi ;n,\omega ,\sigma } ( {\boldsymbol{q}}, x_{2}) \big | \le \big | W^{(h)}_{\phi \psi ;n,\omega ,\sigma } ( {\boldsymbol{q}}, x_{2}) \big | + \sum _{m,\omega '} \big | W^{(h)}_{\phi \psi ;m,\omega ',\sigma } ( {\boldsymbol{q}} + s_{\omega \omega '}, x_{2})\big | \\&\qquad \cdot \big | g^{(h)}_{\omega '}({\boldsymbol{q}} + m{\boldsymbol{\alpha }} + s_{\omega \omega '}) \big | \big |V^{(h-1)}_{n-m;\omega ',\omega }({\boldsymbol{q}} + m {\boldsymbol{\alpha }} + s_{\omega \omega '})\big |. \end{aligned} \end{aligned}$$

Consider the second term in the right-hand side of (4.142). We have, using the bounds (4.139), (4.141):

4.143 $$\begin{aligned} \begin{aligned}&\sum _{m,\omega '}\big | W^{(h)}_{\phi \psi ;n,\omega ',\sigma } ( {\boldsymbol{q}} + s_{\omega \omega '}, x_{2})\big | \big | g^{(h)}_{\omega '}({\boldsymbol{q}} + n{\boldsymbol{\alpha }} + s_{\omega \omega '}) \big | \big | V^{(h-1)}_{n-m;\omega ',\omega }({\boldsymbol{q}} + n {\boldsymbol{\alpha }} + s_{\omega \omega '})\big | \\&\quad \le \sum _{m,\omega '} K |\lambda |^{1+\delta _{n\ne 0}} e^{-\frac{c}{8}|m|} e^{- \frac{{\tilde{c}}}{32}|x_{2}|_{\omega '}} \gamma ^{\xi h} e^{-\frac{c}{4} |n-m|} e^{-\frac{{\tilde{c}}}{32} L_{2} \delta _{\omega ,-\omega '} } \\&\quad \le C |\lambda |^{\delta _{n\ne 0}} e^{-\frac{c}{8}|n|} e^{- \frac{{\tilde{c}}}{32}|x_{2}|_{\omega }} \gamma ^{\xi h} \end{aligned} \end{aligned}$$

for |λ| small enough, with *C* as in (4.139). We used that $|x_{2}|_{\omega '} - |x_{2}|_{\omega } + 2L_{2} \ge L_{2}$, recall (3.2). Plugging the bounds (4.140), (4.143) in (4.142) we get:

4.144 $$\begin{aligned} \big | W^{(h-1)}_{\phi \psi ;n,\omega ,\sigma } ( {\boldsymbol{q}}, x_{2}) \big | \le \sum ^{0}_{j > h} \gamma ^{\xi j} C|\lambda |^{\delta _{n\ne 0}} e^{-\frac{c}{8}|n|} e^{- \frac{{\tilde{c}}}{32}|x_{2}|_{\omega }} + \gamma ^{\xi h} C |\lambda |^{\delta _{n\ne 0}} e^{-\frac{c}{8}|n|} e^{- \frac{{\tilde{c}}}{32}|x_{2}|_{\omega }}, \nonumber \\ \end{aligned}$$

which concludes the check of (4.140) with *h* replaced by h-1. Let us now prove (4.136). From the recursion relation (4.134), we have:

4.145 $$\begin{aligned} \begin{aligned}&\big | W^{(h-1)}_{\phi \psi ;n,\omega ,\sigma } ( {\boldsymbol{q}}, x_{2}) - W^{(h)}_{\phi \psi ;n,\omega ,\sigma } ( {\boldsymbol{q}}, x_{2})\big | \\&\quad \le \sum _{m,\omega '} \big | W^{(h)}_{\phi \psi ;m,\omega ',\sigma } ( {\boldsymbol{q}} + s_{\omega \omega '}, x_{2})\big | \big | g^{(h)}_{\omega '}({\boldsymbol{q}} + m{\boldsymbol{\alpha }} + s_{\omega \omega '})\big | \big | V^{(h-1)}_{n-m;\omega ',\omega }({\boldsymbol{q}} + m {\boldsymbol{\alpha }} + s_{\omega \omega '})\big |. \end{aligned} \nonumber \\ \end{aligned}$$

By (4.143), (4.139) we get:

4.146 $$\begin{aligned} \big | W^{(h-1)}_{\phi \psi ;n,\omega ,\sigma } ( {\boldsymbol{q}}, x_{2}) - W^{(h)}_{\phi \psi ;n,\omega ,\sigma } ( {\boldsymbol{q}}, x_{2})\big | \le C \gamma ^{\xi h} |\lambda |^{1+\delta _{n\ne 0}} e^{-\frac{c}{8}|n|} e^{- \frac{{\tilde{c}}}{32}|x_{2}|_{\omega }}. \nonumber \\ \end{aligned}$$

Therefore, by a telescopic sum argument:

4.147 $$\begin{aligned} \begin{aligned}&\big | W^{(h-1)}_{\phi \psi ;n,\omega ,\sigma } ( {\boldsymbol{q}}, x_{2}) - W^{(k)}_{\phi \psi ;n,\omega ,\sigma } ( {\boldsymbol{q}}, x_{2}) \big |\\&\quad \le \sum _{j=h}^{k} \big |W^{(j-1)}_{\phi \psi ;n,\omega ,\sigma } ( {\boldsymbol{q}}, x_{2}) - W^{(j)}_{\phi \psi ;n,\omega ,\sigma } ( {\boldsymbol{q}}, x_{2}) \big | \\&\quad \le \sum _{j=h}^{k} C \gamma ^{\xi j} |\lambda |^{1+\delta _{n\ne 0}} e^{-\frac{c}{8}|n|} e^{- \frac{{\tilde{c}}}{32}|x_{2}|_{\omega }} \\&\quad \le K |\lambda |^{1+\delta _{n\ne 0}} \gamma ^{\xi k} e^{-\frac{c}{8}|n|} e^{- \frac{{\tilde{c}}}{32}|x_{2}|_{\omega }} \end{aligned} \end{aligned}$$

where the second inequality follows from (4.146). This proves (4.136). To conclude, let us now prove (4.137). Assume that it holds on scales k≥h:

4.148 $$\begin{aligned} \big | \text {d}_{q_{0}}^{n_{0}} \text {d}_{q_{1}}^{n_{1}}W^{(k)}_{\phi \psi ;n,\omega ,\sigma } ( {\boldsymbol{q}}, x_{2}) \big | \le C \gamma ^{(\xi - 1)(k+1)} |\lambda |^{\delta _{n\ne 0}} e^{-\frac{c}{8}|n|} e^{- \frac{\tilde{c}}{32}|x_{2}|_{\omega }}. \end{aligned}$$

As for (4.141), the bound (4.148) is true on scale 0. Let us check it on smaller scales. By the recursion relation:

4.149 $$\begin{aligned} \begin{aligned} \text {d}_{q_{0}}^{n_{0}}&\text {d}_{q_{1}}^{n_{1}} W^{(h-1)}_{\phi \psi ;n,\omega ,\sigma } ( {\boldsymbol{q}}, x_{2}) = \text {d}_{q_{0}}^{n_{0}} \text {d}_{q_{1}}^{n_{1}} W^{(h)}_{\phi \psi ;n,\omega ,\sigma } ( {\boldsymbol{q}}, x_{2})\\&\quad + \sum _{m,\omega '} \text {d}_{q_{0}}^{n_{0}} \text {d}_{q_{1}}^{n_{1}} \Big (W^{(h)}_{\phi \psi ;m,\omega ',\sigma } ( {\boldsymbol{q}} + s_{\omega \omega '}, x_{2}) g^{(h)}_{\omega '}({\boldsymbol{q}} + m{\boldsymbol{\alpha }} + s_{\omega \omega '}) \\&\qquad \cdot V^{(h-1)}_{n-m;\omega ',\omega }({\boldsymbol{q}} + m {\boldsymbol{\alpha }} + s_{\omega \omega '})\Big ). \end{aligned} \end{aligned}$$

Using the improved estimate (4.116) on the derivatives of the effective potential together with (4.137) on scale *h*, we get:

4.150 $$\begin{aligned} \begin{aligned} \Big | \text {d}_{q_{0}}^{n_{0}}&\text {d}_{q_{1}}^{n_{1}} W^{(h-1)}_{\phi \psi ;n,\omega ,\sigma } ( {\boldsymbol{q}}, x_{2}) \Big | \le C \gamma ^{(\xi - 1)(h+1) } |\lambda |^{\delta _{n\ne 0}} e^{-\frac{c}{8}|n|} e^{- \frac{{\tilde{c}}}{32}|x_{2}|_{\omega }} \\&+ \sum _{m,\omega '} K |\lambda |^{1+\delta _{n\ne 0}} e^{-\frac{c}{8}|m|} e^{- \frac{{\tilde{c}}}{32}|x_{2}|_{\omega '}} \gamma ^{(\xi - 1) h} e^{-\frac{c}{4} |n-m|} e^{-\frac{{\tilde{c}}}{32} L_{2} \delta _{\omega ,-\omega '} }; \end{aligned} \end{aligned}$$

choosing γ such that $\gamma ^{\xi - 1} \le 1/2$ and |λ| small enough, we get that both terms in the right-hand side of (4.150) are bounded by:

4.151 $$\begin{aligned} \frac{C}{2} \gamma ^{(\xi - 1)h} |\lambda |^{\delta _{n\ne 0}} e^{-\frac{c}{8}|n|} e^{- \frac{{\tilde{c}}}{32}|x_{2}|_{\omega }} \end{aligned}$$

with *C* as in (4.148). This concludes the proof of (4.137). □

##### Remark 4.15

The bound (4.137) allows us to prove that, for $\Vert {\boldsymbol{q}} + n{\boldsymbol{\alpha }}\Vert \le \gamma ^{h}$:

4.152 $$\begin{aligned} \begin{aligned}&\big | W^{(h-1)}_{\phi \psi ;n,\omega ,\sigma } ( {\boldsymbol{q}}, x_{2}) - W^{(h-1)}_{\phi \psi ;n,\omega ,\sigma } ( -n{\boldsymbol{\alpha }}, x_{2}) \big |\\&\quad \le \int _{0}^{1} dt\, \Big |\frac{d}{dt} W^{(h-1)}_{\phi \psi ;n,\omega ,\sigma } \big ( -n{\boldsymbol{\alpha }} + t ({\boldsymbol{q}} + n{\boldsymbol{\alpha }}), x_{2}\big )\Big | \\&\quad \le C \gamma ^{\xi h} |\lambda |^{\delta _{n\ne 0}} e^{-\frac{c}{8}|n|} e^{- \frac{{\tilde{c}}}{32}|x_{2}|_{\omega }}. \end{aligned} \end{aligned}$$

This will be particularly useful in the analysis of the two-point function.

#### Proof of Theorem 3.1

In this section we will show how to use Proposition 4.13 to obtain the asymptotic behavior of the two-point function, Eq. (3.3). We start by writing:

4.153 $$\begin{aligned} \langle \textbf{T}\, \gamma _{x_0}(a_{{\vec x},\sigma }); \gamma _{y_0}(a_{\vec y,\zeta }^{*})\big \rangle _{\beta ,\mu ,L} = \frac{1}{\beta L_{1}} \sum _{n} \sum _{{\boldsymbol{k}}} e^{i{\boldsymbol{k}}\cdot ({\boldsymbol{x}} - {\boldsymbol{y}})} e^{-i n \alpha y_{1}} {\hat{S}}_{2;n,\sigma ,\zeta }({\boldsymbol{k}}; x_{2}, y_{2}), \nonumber \\ \end{aligned}$$

where, using the Grassmann representation of the two-point function:

4.154 $$\begin{aligned} {\hat{S}}_{2;n,\sigma ,\zeta }({\boldsymbol{k}}; x_{2}, y_{2}) = \beta L_{1} \lim _{N\rightarrow \infty } \frac{\partial ^{2}}{\partial {\hat{\phi }}^{+}_{{\boldsymbol{k}}, x_{2}, \sigma } \partial {\hat{\phi }}^{-}_{{\boldsymbol{k}} + n{\boldsymbol{\alpha }}, y_{2}, \zeta }} \mathcal {W}_{N}(\phi )\Big |_{\phi = 0}. \end{aligned}$$

By (4.126)-(4.128), we have:

4.155 $$\begin{aligned} {\hat{S}}_{2;n,\sigma ,\zeta }({\boldsymbol{k}}; x_{2}, y_{2}) = \sum _{h = h_{\beta }}^{0} W^{(h)}_{\phi \phi ; n, \sigma ,\zeta }({\boldsymbol{k}}; x_{2}, y_{2}) + {\hat{R}}_{2;n,\sigma ,\zeta }({\boldsymbol{k}}; x_{2}, y_{2}), \end{aligned}$$

where:

(i) the kernels $W^{(h)}_{\phi \phi ; n, \sigma ,\zeta }({\boldsymbol{k}}; x_{2}, y_{2})$ satisfy the recursion (4.133);
(ii) ${\hat{R}}_{2} = W^{(>0)}$, recall (4.122), and it satisfies:
  4.156 $$\begin{aligned} \Big | \text {d}_{k_{0}}^{n_{0}} \text {d}_{k_{1}}^{n_{1}} {\hat{R}}_{2;n,\sigma ,\zeta }({\boldsymbol{k}}; x_{2}, y_{2}) \Big | \le C_{n_{0},n_{1}} |\lambda |^{\delta _{n\ne 0}} e^{-c|n|}e^{- c|x_{2} - y_{2}|}. \end{aligned}$$

Let us now focus on the main term in (4.155), due to the integration of the single-scale fields. Recall Eq. (4.133); we rewrite it as:

4.157 $$\begin{aligned} W_{\phi \phi ;n,\sigma ,\zeta }^{(h)}({\boldsymbol{k}}; x_{2}, y_{2}) = \text {A}^{(h)}_{n,\sigma ,\zeta }({\boldsymbol{k}}; x_{2}, y_{2}) + \textrm{B}^{(h)}_{n,\sigma ,\zeta }({\boldsymbol{k}}; x_{2}, y_{2}) \end{aligned}$$

with:

4.158 $$\begin{aligned} \begin{aligned}&\text {A}^{(h)}_{n,\sigma ,\zeta }({\boldsymbol{k}}; x_{2}, y_{2}) \\&\quad := \sum _{m,\omega } W^{(h)}_{\phi \psi ;m,\omega ,\sigma } ( {\boldsymbol{q}}, x_{2}) g^{(h)}_{\omega }({\boldsymbol{q}} + m{\boldsymbol{\alpha }}) W^{(h)}_{\psi \phi ;-m + n,\omega ,\zeta } ( {\boldsymbol{q}} + m{\boldsymbol{\alpha }}, y_{2}) \\&\quad \equiv \sum _{\omega } \text {A}^{(h)}_{n,\sigma ,\zeta ,\omega }({\boldsymbol{q}}; x_{2}, y_{2}), \end{aligned} \end{aligned}$$

where we recall that, at the argument of the sum over ω, ${\boldsymbol{q}} \equiv {\boldsymbol{q}}({\boldsymbol{k}}) = {\boldsymbol{k}} - {\boldsymbol{k}_{F}^{\omega }}$ and:

4.159 $$\begin{aligned} \begin{aligned}&\textrm{B}^{(h)}_{n,\sigma ,\zeta }({\boldsymbol{k}}; x_{2}, y_{2})\\  &:= \sum _{\begin{array}{c} m,m' \\ \omega ,\omega ' \end{array}}W^{(h)}_{\phi \psi ;m,\omega ,\sigma } ( {\boldsymbol{q}}, x_{2}) g^{(h)}_{\omega }({\boldsymbol{q}} + m{\boldsymbol{\alpha }}) V^{(h-1)}_{m';\omega ,\omega '}({\boldsymbol{q}} + m{\boldsymbol{\alpha }} ) \\&\qquad \quad \,\,\, \cdot g^{(h)}_{\omega '}\big ({\boldsymbol{q}} + (m + m'){\boldsymbol{\alpha }} + s_{\omega \omega '}\big ) W^{(h)}_{\psi \phi ;-m - m' + n,\omega ',\zeta } \big ( {\boldsymbol{q}} + (m + m'){\boldsymbol{\alpha }} + s_{\omega \omega '}, y_{2}\big )\\&\equiv \sum _{\omega ,\omega '} \textrm{B}^{(h)}_{n,\sigma ,\zeta ,\omega ,\omega '}({\boldsymbol{q}}; x_{2}, y_{2}). \end{aligned} \end{aligned}$$

We define:

4.160 $$\begin{aligned} Z^{(h)}_{\phi \psi ;n,\omega ,\sigma }(x_{2}):= W^{(h)}_{\phi \psi ;n,\omega ,\sigma } ({\boldsymbol{0}}_{\beta } - n{\boldsymbol{\alpha }}, x_{2}),\qquad Z^{(h)}_{\psi \phi ;n,\omega ,\sigma }(x_{2}):= W^{(h)}_{\psi \phi ;n,\omega ,\sigma }({\boldsymbol{0}}_{\beta }, x_{2}). \nonumber \\ \end{aligned}$$

By the identity (4.138), these two quantities are related by:

4.161 $$\begin{aligned} Z^{(h)}_{\psi \phi ;n,\omega ,\sigma }(x_{2}) = \overline{Z^{(h)}_{\phi \psi ;-n,\omega ,\sigma }(x_{2})}. \end{aligned}$$

Furthermore, we define:

4.162 $$\begin{aligned} \begin{aligned} {\mathfrak R} W^{(h)}_{\phi \psi ;n,\omega ,\sigma } ( {\boldsymbol{q}}, x_{2})&:= W^{(h)}_{\phi \psi ;n,\omega ,\sigma } ( {\boldsymbol{q}}, x_{2}) - Z^{(h)}_{\phi \psi ;n,\omega ,\sigma }(x_{2}) \\ {\mathfrak R} W^{(h)}_{\psi \phi ;-m + n,\omega ,\zeta } ( {\boldsymbol{q}} + m{\boldsymbol{\alpha }}, y_{2})&:= W^{(h)}_{\psi \phi ;-m + n,\omega ,\zeta } ( {\boldsymbol{q}} + m{\boldsymbol{\alpha }}, y_{2})- Z^{(h)}_{\psi \phi ;-m+n,\omega ,\zeta }(y_{2}). \end{aligned} \end{aligned}$$

**Discussion of** $\text {A}^{(h)}$. We rewrite:

4.163 $$\begin{aligned} \text {A}^{(h)}_{n,\sigma ,\zeta ,\omega }({\boldsymbol{q}}; x_{2}, y_{2})= &   \sum _{m} Z^{(h)}_{\phi \psi ;m,\omega ,\sigma } ( x_{2}) g^{(h)}_{\omega }({\boldsymbol{q}} + m{\boldsymbol{\alpha }}) Z^{(h)}_{\psi \phi ;-m + n,\omega ,\zeta } (y_{2}) \nonumber \\  &   + \text {A}^{(h)}_{1; n,\sigma ,\zeta ,\omega }({\boldsymbol{q}}; x_{2}, y_{2}) \end{aligned}$$

where $\text {A}^{(h)}_{1}$ contains at least one between

4.164 $$\begin{aligned} {\mathfrak R} W^{(h)}_{\phi \psi ;m,\omega ,\sigma } ( {\boldsymbol{q}}, x_{2}),\qquad {\mathfrak R} W^{(h)}_{\psi \phi ;-m + n,\omega ,\zeta } ( {\boldsymbol{q}} + m{\boldsymbol{\alpha }}, y_{2}). \end{aligned}$$

For $\Vert {\boldsymbol{q}} + m\alpha \Vert \le \gamma ^{h+1}$, which is imposed by the support of the single-scale propagator in $\text {A}^{(h)}$, both kernels in (4.164) can be estimated as in Eq. (4.152). We have:

4.165 $$\begin{aligned} \big | {\mathfrak R} W^{(h)}_{\phi \psi ;m,\omega ,\sigma } ( {\boldsymbol{q}}, x_{2}) \big | \le C \gamma ^{\xi (h+1)} |\lambda |^{\delta _{m\ne 0}} e^{-\frac{c}{8}|m|} e^{- \frac{{\tilde{c}}}{32}|x_{2}|_{\omega }},\ \end{aligned}$$

and the same bound holds true for the second kernel in (4.164). Therefore, using that every ${\boldsymbol{q}}$ derivative introduces a factor $\gamma ^{-h}$ in the estimate for the kernel, we find:

4.166 $$\begin{aligned} | \text {d}_{{ q}_{0}}^{n_{0}}\text {d}_{{ q}_{1}}^{n_{1}} \text {A}^{(h)}_{1; n,\sigma ,\zeta ,\omega }({\boldsymbol{q}}; x_{2}, y_{2}) \big | \le C_{n_{0},n_{1}} \gamma ^{-h(1 + n_{0} + n_{1})} \gamma ^{\xi h} |\lambda |^{\delta _{n\ne 0}} e^{-\frac{c}{16}|n|} e^{- \frac{{\tilde{c}}}{32}|x_{2}|_{\omega }} e^{- \frac{\tilde{c}}{32}|y_{2}|_{\omega }}. \nonumber \\ \end{aligned}$$

Let us now consider the main term in (4.163). By using (4.136), we have:

4.167 $$\begin{aligned} \big |Z^{(h_{\beta })}_{\phi \psi ;n,\omega ,\sigma }(x_{2}) - Z^{(h)}_{\phi \psi ;n,\omega ,\sigma }(x_{2})\big |\le K |\lambda |^{\delta _{n\ne 0}} \gamma ^{\xi h} e^{-\frac{c}{8}|n|} e^{- \frac{{\tilde{c}}}{32}|x_{2}|_{\omega }}. \end{aligned}$$

Thus, we have:

4.168 $$\begin{aligned} \begin{aligned}&\sum _{m} Z^{(h)}_{\phi \psi ;m,\omega ,\sigma } ( x_{2}) g^{(h)}_{\omega }({\boldsymbol{q}} + m{\boldsymbol{\alpha }}) Z^{(h)}_{\psi \phi ;-m + n,\omega ,\zeta } (y_{2}) \\&\quad = \sum _{m} Z^{(h_{\beta })}_{\phi \psi ;m,\omega ,\sigma } ( x_{2}) g^{(h)}_{\omega }({\boldsymbol{q}} + m{\boldsymbol{\alpha }}) Z^{(h_{\beta })}_{\psi \phi ;-m + n,\omega ,\zeta } (y_{2})+ \text {A}^{(h)}_{2; n,\sigma ,\zeta ,\omega }({\boldsymbol{q}}; x_{2}, y_{2}), \end{aligned} \end{aligned}$$

where $\text {A}^{(h)}_{2}$ satisfies the bound (4.166). Let us now focus on the main term in (4.168). We introduce the short-hand notation:

4.169 $$\begin{aligned} Z_{m,\omega ,\sigma } ( x_{2}):= Z^{(h_{\beta })}_{\phi \psi ;m,\omega ,\sigma } ( x_{2}); \end{aligned}$$

by (4.161) we have $Z^{(h_{\beta })}_{\psi \phi ;-m+n,\omega ,\zeta } ( y_{2}) = \overline{Z_{-n+m,\omega ,\zeta }(y_{2})}$. Thus, we can rewrite (4.163) as:

4.170 $$\begin{aligned} \text {A}^{(h)}_{n,\sigma ,\zeta }({\boldsymbol{k}}; x_{2}, y_{2})= &   \sum _{\omega ,m} Z_{m,\omega ,\sigma }(x_{2}) g^{(h)}_{\omega }({\boldsymbol{q}} + m{\boldsymbol{\alpha }}) \overline{Z_{-n+m,\omega ,\zeta }(y_{2})} \nonumber \\  &   + \sum _{i=1,2} \text {A}^{(h)}_{i;n,\sigma ,\zeta }({\boldsymbol{k}}; x_{2}, y_{2}). \end{aligned}$$

Now, recall the expression of the single-scale propagator, Eq. (4.72). By the estimate (4.115) on the beta function, we know that the velocities $v_{\nu ,\omega ,h}({\boldsymbol{q}})$ approach their infrared limit $v_{\nu ,\omega }:= v_{\nu ,\omega ,h_{\beta }}$ with the following rate:

4.171 $$\begin{aligned} |v_{\nu ,\omega ,h}({\boldsymbol{q}}) - v_{\nu ,\omega }| \le C_{r} \gamma ^{r h} |\lambda |^{2}\qquad \text {for all } r\in \mathbb {N}. \end{aligned}$$

Let $g^{(h)}_{\omega ;\textrm{s}}({\boldsymbol{q}}):= f^{(h)}_{\omega ;\textrm{s}}({\boldsymbol{q}}) / (i v_{0,\omega } q_{0} + v_{1,\omega } q_{1})$ be the propagator after replacing the velocities with constant values, and let $f^{(h)}_{\omega ;\textrm{s}}({\boldsymbol{q}})$ be the new single-scale cutoff function after replacing the velocities, $f^{(h)}_{\omega ;\textrm{s}}({\boldsymbol{q}}):= \chi \Big (\!2^{-h} \sqrt{v_{0,\omega }^{2}q_{0}^{2} + v_{1,\omega }^{2}q_{1}^{2} }\!\Big ) - \chi \Big (\! 2^{-(h-1)} \sqrt{v_{0,\omega }^{2}q_{0}^{2} + v_{1,\omega }^{2}q_{1}^{2} }\!\Big )$. Hence,

4.172 $$\begin{aligned} \text {A}^{(h)}_{n,\sigma ,\zeta }({\boldsymbol{k}}; x_{2}, y_{2})= &   \sum _{\omega ,m} Z_{m,\omega ,\sigma }(x_{2}) g^{(h)}_{\omega ;\textrm{s}}({\boldsymbol{q}} + m{\boldsymbol{\alpha }}) \overline{Z_{-n+m,\omega ,\zeta }(y_{2})} \nonumber \\  &   + \sum _{i=1,2,3} \text {A}^{(h)}_{i;n,\sigma ,\zeta }({\boldsymbol{k}}; x_{2}, y_{2}), \end{aligned}$$

where the new error term $\text {A}^{(h)}_{3}$ takes into account the replacement of the velocities, and it is not difficult to see that it satisfies the bound (4.166). The main term in (4.172) will contribute to the scaling limit of the two-point function, while all the other terms will contribute with subleading corrections, that decay faster than the main term in configuration space.

**Discussion of** $\textrm{B}^{(h)}$. Consider now the term $\textrm{B}^{(h)}$, Eq. (4.159). This term is subleading with respect to the main term in (4.172): informally, with respect to (4.172), this term contains an extra single-scale propagator, and an extra potential term. Since the single scale propagator is bounded dimensionally as $\gamma ^{-h}$, while the potential term is bounded proportionally to $\gamma ^{h(1+\xi )}$, we have a net dimensional gain of $\gamma ^{\xi h}$ with respect to the leading term in (4.172). Ultimately, the term $\textrm{B}^{(h)}_{n,\sigma ,\zeta ,\omega ,\omega '}({\boldsymbol{q}}; x_{2}, y_{2})$ satisfies a bound similar to (4.166):

4.173 $$\begin{aligned}  &   \big | \text {d}_{{q}_{0}}^{n_{0}}\text {d}_{{ k}_{1}}^{n_{1}} \textrm{B}^{(h)}_{n,\sigma ,\zeta ,\omega }({\boldsymbol{q}}; x_{2}, y_{2}) \big | \nonumber \\  &   \quad \le C_{n_{0},n_{1}} \gamma ^{-h(1 + n_{0} + n_{1})} \gamma ^{\xi h} |\lambda |^{1+\delta _{n\ne 0}} e^{-\frac{c}{16}|n|} e^{- \frac{{\tilde{c}}}{32}|x_{2}|_{\omega }} e^{- \frac{\tilde{c}}{32}|y_{2}|_{\omega '}}. \end{aligned}$$

We omit the details.

**Bounds for the remainder term.** All in all, we have:

4.174 $$\begin{aligned}  &   \big \langle \textbf{T}\, \gamma _{x_0}(a_{{\vec x},\sigma }); \gamma _{y_0}(a_{\vec y,\zeta }^{*})\big \rangle _{\beta ,\mu ,L} \nonumber \\  &   \quad = \frac{1}{\beta L_{1}} \sum _{n,m,\omega } \sum _{{\boldsymbol{k}}} e^{i{\boldsymbol{k}}\cdot ({\boldsymbol{x}} - {\boldsymbol{y}})} e^{-i n \alpha y_{1}} Z_{m,\omega ,\sigma }(x_{2}) g_{\omega ;\textrm{s}}\big ({\boldsymbol{q}(\boldsymbol{k})} + m{\boldsymbol{\alpha }}\big ) \overline{Z_{-n+m,\omega ,\zeta }(y_{2})} \nonumber \\  &   \qquad + R_{\sigma ,\zeta }(\vec {\boldsymbol{x}}, \vec {\boldsymbol{y}}), \end{aligned}$$

where we set:

4.175 $$\begin{aligned} g_{\omega ,\textrm{s}}({\boldsymbol{q}}):= \sum _{h=h_{\beta }}^{0} g^{(h)}_{\omega ,\textrm{s}}({\boldsymbol{q}}) = \frac{\chi \Big (\sqrt{v_{0,\omega }^{2}q_{0}^{2} + v_{1,\omega }^{2}q_{1}^{2} }\Big )}{i v_{0,\omega } q_{0} + v_{1,\omega } q_{1}}. \end{aligned}$$

Eq. (4.175) is an effective relativistic propagator, with renormalized velocities, and an ultraviolet cutoff; recall the definition of the cutoff function, Eq. (3.1). The remainder term in (4.174) is $R_{\sigma ,\zeta }(\vec {\boldsymbol{x}}, \vec {\boldsymbol{y}}) = R_{1;\sigma ,\zeta }(\vec {\boldsymbol{x}}, \vec {\boldsymbol{y}}) + R_{2;\sigma ,\zeta }(\vec {\boldsymbol{x}}, \vec {\boldsymbol{y}})$ with:

4.176 $$\begin{aligned} R_{i;\sigma ,\zeta }(\vec {\boldsymbol{x}}, \vec {\boldsymbol{y}})  &   = \frac{1}{\beta L_{1}} \sum _{n,{\boldsymbol{k}}} e^{i{\boldsymbol{k}}\cdot ({\boldsymbol{x}} - {\boldsymbol{y}})} e^{-i n \alpha y_{1}} {\hat{R}}_{i;n,\sigma ,\zeta }({\boldsymbol{k}}; x_{2}, y_{2}) \nonumber \\  &   \equiv \sum _{n} e^{-i n \alpha y_{1}} R_{i;n,\sigma ,\zeta }({\boldsymbol{x} - {\boldsymbol{y}}}; x_{2}, y_{2}) \end{aligned}$$

where ${\hat{R}}_{2;n,\sigma ,\zeta }({\boldsymbol{k}}; x_{2}, y_{2})$ satisfies the estimate (4.156) while ${\hat{R}}_{1;n,\sigma ,\zeta }({\boldsymbol{k}}; x_{2}, y_{2})$ is given by:

4.177 $$\begin{aligned} {\hat{R}}_{1;n,\sigma ,\zeta }({\boldsymbol{k}}; x_{2}, y_{2}) = \sum _{h=h_{\beta }}^{0} \Big (\sum _{i=1,2,3} \text {A}^{(h)}_{i;n,\sigma ,\zeta }({\boldsymbol{k}}; x_{2}, y_{2}) + \textrm{B}^{(h)}_{n,\sigma ,\zeta }({\boldsymbol{k}}; x_{2}, y_{2})\Big ). \nonumber \\ \end{aligned}$$

To prove a decay estimate for $R_{i;n,\sigma ,\zeta }({\boldsymbol{x} - {\boldsymbol{y}}}; x_{2}, y_{2})$, we proceed as follows. Let:

4.178 $$\begin{aligned} (x_{0} - y_{0})_{L}:= \frac{\beta }{\pi } \sin \big (\pi (x_{0} - y_{0}) / \beta \big ) ,\qquad (x_{1} - y_{1})_{L}:= \frac{L_{1}}{\pi } \sin \big (\pi (x_{0} - y_{0}) / L_{1}\big ). \nonumber \\ \end{aligned}$$

Observe that, for $\beta , L_{1}\rightarrow \infty$, these objects converge pointwise to $(x_{0} - y_{0})$ and to $(x_{1} - y_{1})$. Then, observe that, for ν=0,1:

4.179 $$\begin{aligned} \begin{aligned}&\Big |(x_{\nu } - y_{\nu })_{L}^{m}R^{(h)}_{i;n,\sigma ,\zeta }({\boldsymbol{x} - {\boldsymbol{y}}}; x_{2}, y_{2})\Big | \\&\quad = \Big |\frac{1}{\beta L_{1}} \sum _{{\boldsymbol{k}}} \big (\text {d}_{k_\nu }^{m} e^{i{\boldsymbol{k}}\cdot ({\boldsymbol{x}} - {\boldsymbol{y}})}\big ) e^{-i n \alpha y_{1}} {\hat{R}}^{(h)}_{i;n,\sigma ,\zeta }({\boldsymbol{k}}; x_{2}, y_{2})\Big | \\&\quad = \Big |\frac{1}{\beta L_{1}} \sum _{{\boldsymbol{k}}} e^{i{\boldsymbol{k}}\cdot ({\boldsymbol{x}} - {\boldsymbol{y}})} e^{-i n \alpha y_{1}} \big (\text {d}_{\nu }^{m} {\hat{R}}^{(h)}_{i;n,\sigma ,\zeta }({\boldsymbol{k}}; x_{2}, y_{2})\big )\Big |, \end{aligned} \end{aligned}$$

where the last step follows from summation by parts, using that ${\hat{R}}^{(h)}_{i}$ vanishes as $k_{0}\rightarrow \infty$ and it is periodic in $k_{1}$ in the (discrete) Brillouin zone. Consider the error term $R_{1}$. From the estimate

4.180 $$\begin{aligned} \big |\text {d}_{\nu }^{m} {\hat{R}}^{(h)}_{1;n,\sigma ,\zeta }({\boldsymbol{k}}; x_{2}, y_{2})\big | \le C_{m} \gamma ^{-h(1 + m)} \gamma ^{\xi h} |\lambda |^{\delta _{n\ne 0}} e^{-\frac{c}{16}|n|} e^{- \frac{\tilde{c}}{32}|x_{2}|_{L}} e^{- \frac{{\tilde{c}}}{32}|y_{2}|_{L}}, \nonumber \\ \end{aligned}$$

we find, using that ***k*** is in the support of the propagator on scale *h*:

4.181 $$\begin{aligned} \big |\gamma ^{mh}(x_{\nu } - y_{\nu })_{L}^{m}R^{(h)}_{1;n,\sigma ,\zeta }({\boldsymbol{x} - {\boldsymbol{y}}}; x_{2}, y_{2})\big | \le K_{m} \gamma ^{h(1+\xi )} |\lambda |^{\delta _{n\ne 0}} e^{-\frac{c}{16}|n|} e^{- \frac{{\tilde{c}}}{32}|x_{2}|_{L}} e^{- \frac{{\tilde{c}}}{32}|y_{2}|_{L}}.\nonumber \\ \end{aligned}$$

From (4.181) we easily get:

4.182 $$\begin{aligned} \big |R^{(h)}_{1;n,\sigma ,\zeta }({\boldsymbol{x} - {\boldsymbol{y}}}; x_{2}, y_{2})\big | \le \frac{K_{m} \gamma ^{h(1+\xi )} |\lambda |^{\delta _{n\ne 0}} e^{-\frac{c}{16}|n|} e^{- \frac{\tilde{c}}{32}|x_{2}|_{L}} e^{- \frac{{\tilde{c}}}{32}|y_{2}|_{L}}}{1 + (\gamma ^{h} \Vert {\boldsymbol{x}} - {\boldsymbol{y}} \Vert )^{m}}.\nonumber \\ \end{aligned}$$

Thus, writing, for $0<\xi '<\xi$:

4.183 $$\begin{aligned} \gamma ^{h(1 + \xi )} = \gamma ^{h(\xi - \xi ')} \gamma ^{h(1 + \xi ')} = \gamma ^{h(\xi - \xi ')} \frac{1+\Vert {\boldsymbol{x}} - {\boldsymbol{y}}\Vert ^{1 + \xi '}}{1+\Vert {\boldsymbol{x}} - {\boldsymbol{y}}\Vert ^{1 + \xi '}} \gamma ^{h(1 + \xi ')}, \end{aligned}$$

and observing that $\frac{(1+\Vert {\boldsymbol{x}} - {\boldsymbol{y}}\Vert ^{1 + \xi '}) \gamma ^{h(1 + \xi ')}}{ 1 + (\gamma ^{h} \Vert {\boldsymbol{x}} - {\boldsymbol{y}} \Vert )^{m} } \le C$ uniformly in $\Vert {\boldsymbol{x}} - {\boldsymbol{y}} \Vert$, we obtain:

4.184 $$\begin{aligned} \begin{aligned} \big |R_{1;n,\sigma ,\zeta }({\boldsymbol{x} - {\boldsymbol{y}}}; x_{2}, y_{2})\big |&\le \sum _{h=h_{\beta }}^{0} \big |R^{(h)}_{1;n,\sigma ,\zeta }({\boldsymbol{x} - {\boldsymbol{y}}}; x_{2}, y_{2})\big | \\&\le \frac{K |\lambda |^{\delta _{n\ne 0}} e^{-\frac{c}{16}|n|} e^{- \frac{{\tilde{c}}}{32}|x_{2}|_{L}} e^{- \frac{{\tilde{c}}}{32}|y_{2}|_{L}}}{1 + \Vert {\boldsymbol{x}} - {\boldsymbol{y}}\Vert ^{1 + \xi '}} \sum _{h=h_{\beta }}^{0} \gamma ^{h(\xi - \xi ')} \\&\le \frac{C |\lambda |^{\delta _{n\ne 0}} e^{-\frac{c}{16}|n|} e^{- \frac{{\tilde{c}}}{32}|x_{2}|_{L}} e^{- \frac{{\tilde{c}}}{32}|y_{2}|_{L}}}{1 + \Vert {\boldsymbol{x}} - {\boldsymbol{y}}\Vert ^{1 + \xi '}}. \end{aligned} \nonumber \\ \end{aligned}$$

Similarly, using the bound (4.156) we obtain:

4.185 $$\begin{aligned} \big |R_{2;n,\sigma ,\zeta }(\vec {\boldsymbol{x}}, \vec {\boldsymbol{y}})\big | \le C |\lambda |^{\delta _{n\ne 0}} e^{-\frac{c}{16}|n|} e^{-c\Vert \vec {\boldsymbol{x}} - \vec {\boldsymbol{y}}\Vert }. \end{aligned}$$

Eqs. (4.176), (4.184), (4.185) prove (3.9).

**Asymptotics of the leading term.** Consider now the main term in (4.174). It is:

4.186 $$\begin{aligned} S_{2; \sigma , \zeta }^{\textrm{s}}(\vec {\boldsymbol{x}}, \vec {\boldsymbol{y}}):= \frac{1}{\beta L_{1}} \sum _{\begin{array}{c} n,m \\ \omega , {\boldsymbol{k}} \end{array}} e^{i{\boldsymbol{k}}\cdot ({\boldsymbol{x}} - {\boldsymbol{y}})} e^{-i n \alpha y_{1}} Z_{m,\omega ,\sigma }(x_{2}) g_{\omega ;\textrm{s}}\big (\boldsymbol{q}(\boldsymbol{k} )+ m{\boldsymbol{\alpha }}\big ) \overline{Z_{-n+m,\omega ,\zeta }(y_{2})}.\nonumber \\ \end{aligned}$$

Let:

4.187 $$\begin{aligned} Z_{\omega ,\sigma }(\vec x):= \sum _{m} e^{-im\alpha x_{1}} Z_{m,\omega ,\sigma }(x_{2}). \end{aligned}$$

Then, it is easy to see that:

4.188 $$\begin{aligned} S_{2; \sigma , \zeta }^{\textrm{s}}(\vec {\boldsymbol{x}}, \vec {\boldsymbol{y}}) = \sum _{\omega } e^{i k_{F}^{\omega }(\lambda )(x_{1} - y_{1})} Z_{\omega ,\sigma }(\vec x) \check{g}_{\omega ;\textrm{s}}({\boldsymbol{x}} - {\boldsymbol{y}}) \overline{Z_{\omega ,\zeta }(\vec y)}. \end{aligned}$$

This concludes the proof of (3.3). The only ingredient missing to conclude the proof of Theorem 3.1 are the relations (3.8), which will be proved in Sect. 5.3.1.

## Transport Coefficients

In this section we shall use the construction of the two-point function, Theorem 3.1, to compute the edge conductivity and the edge susceptibility of the model. We will start from the linear response ansatz, as given from Kubo formula, Eq. (2.43). The precise asymptotics of the two-point function derived in the previous section will allow us to compute these transport coefficients, and to prove Theorem 3.3.

In Sect. 5.1 we recall the representation of the real-time transport coefficients in terms of Euclidean correlation functions, and in Sect. 5.2 we discuss the implications of the lattice continuity equation on these Euclidean correlations, called Ward identities. In Sect. 5.3 we discuss implications of the lattice continuity equation for another class of Euclidean correlations, called vertex functions. The Ward identities of Sects. 5.2, 5.3, together with the asymptotic behavior of the two-point function of Theorem 3.1, will allow us to prove our second main result, Theorem 3.3. The proof of this theorem is contained in Sect. 5.4.

### Response Functions in Imaginary Times

A key role in the analysis is played by the lattice continuity equation (2.36), which implies Ward identities for the Euclidean correlation functions. Specifically, we will need the current-current Ward identity and the vertex Ward identity; they will be discussed in this section. Later, we will discuss how these Ward identities can be used to study transport in real time; this is possible thanks to a complex deformation argument, that allows us to relate the real-time transport coefficients to imaginary-time correlation functions. In fact, it turns out that the real-time response functions (2.43) can be rewritten as, see e.g. [3, 29, 48],

5.1 $$\begin{aligned} \chi _{\nu ;\ell }^{\beta ,L}(\eta ,\theta )= &   \theta \sum _{\vec x,\vec y } \mu (\theta \vec x) \phi _{\ell }(\theta y_{1}, y_{2}) \int _{-\beta /2}^{\beta /2} ds\, e^{-i\eta _{\beta } s} \langle \textbf{T}\gamma _{s}(n_{\vec x}); j_{\nu ,\vec y} \rangle _{\beta ,\mu ,L} \nonumber \\  &   + \varepsilon _{\nu }^{\beta ,L}(\eta ,\theta ) \end{aligned}$$

where: $\eta _{\beta }$ is the best approximation of η in $(2\pi /\beta )\mathbb {N}$ such that $\eta _{\beta } \ge \eta$, while $\varepsilon _{\nu ;\ell }^{\beta ,L}(\eta ,\theta ) = 0$ if $\eta \in \frac{2\pi }{\beta } \mathbb {N}$, and it satisfies the bound:

5.2 $$\begin{aligned} | \varepsilon _{\nu ;\ell }^{\beta ,L}(\eta ,\theta ) | \le \frac{C_{\ell }}{\beta \eta ^{3}}\; \end{aligned}$$

uniformly in *L* and θ. The rewriting (5.1) is particularly convenient: the Euclidean correlation function in the right-hand side can be studied via our renormalization group construction. Furthermore, as we shall see in the next section, the lattice continuity equation introduces nontrivial constraints on the structure of this correlation function, that will play an important role in the computation of the response function.

### Current-Current Ward Identity

To begin, let us rewrite the conservation law (2.36) in imaginary time. Using that $\tau _{t}(\mathcal {O}) = \gamma _{it}(\mathcal {O})$ if $[\mathcal {O}, \mathcal {N}] = 0$, we get the Euclidean continuity equation:

5.3 $$\begin{aligned} i\partial _{t} \gamma _{t}(n_{x}) + \sum _{k=1,2} \text {d}_{x_{k}} \gamma _{t}(j_{k,x}) = 0. \end{aligned}$$

We will be interested in the consequences of this identity at the level of the Euclidean correlation functions. Let $\eta \in \frac{2\pi }{\beta }\cdot \mathbb {N}$, and recall the definition (2.41) of the smeared current operator. Recall the notation $\phi _{\theta ,\ell }(\vec x) = \theta \phi _{\ell }(\theta x_{1}, x_{2})$ and let $\mu _{\theta }(\vec x) = \theta \mu (\theta x_{1},\theta x_{2})$. We define:

5.4 $$\begin{aligned} j_{\nu }(\eta , \phi _{\theta ,\ell }):= \int _{0}^{\beta } dt\,e^{-i\eta t} \gamma _{t}\big ( j_{\nu }(\phi _{\theta ,\ell }) \big ). \end{aligned}$$

By time-translation invariance and β-periodicity of the Gibbs state, we have:

5.5 $$\begin{aligned} \begin{aligned}&\langle {\textbf{T}} j_{0}(\eta , \mu _{\theta }); j_{\nu }(-\eta , \phi _{\theta ,\ell }) \rangle _{\beta , \mu , L} \\&\quad = \beta \int _{-\frac{\beta }{2}}^{\frac{\beta }{2}} d s\,e^{-is\eta } \big [ \Theta (s\ge 0) \big \langle \gamma _{s}\big ( j_{0}(\mu _{\theta }) \big ); j_{\nu }(\phi _{\theta ,\ell }) \big \rangle _{\beta ,\mu ,L}\\&\qquad + \Theta (s\le 0) \big \langle j_{\nu }(\phi _{\theta ,\ell }); \gamma _{s}\big ( j_{0}(\mu _{\theta }) \big ) \big \rangle _{\beta ,\mu ,L} \big ], \end{aligned} \end{aligned}$$

with $\Theta (\cdot )$ the indicator function.^3^ Hence, omitting from now on the $\beta , \mu , L$ labels in the Gibbs state:

5.6 $$\begin{aligned} \begin{aligned} \eta \langle \textbf{Tj}_{0}(\eta , \mu _{\theta }); j_{\nu }(-\eta , \phi _{\theta ,\ell }) \rangle&= \beta \int _{-\frac{\beta }{2}}^{\frac{\beta }{2}}\mathrm ds\, (i\partial _{s}\mathrm e^{-is\eta }) \Big [ \Theta (s\ge 0) \big \langle \gamma _{s}\big ( j_{0}(\mu _{\theta }) \big ); j_{\nu }(\phi _{\theta ,\ell }) \big \rangle \\&\quad + \Theta (s\le 0) \big \langle j_{\nu }(\phi _{\theta ,\ell }); \gamma _{s}\big ( j_{0}(\mu _{\theta }) \big ) \big \rangle \Big ]; \end{aligned} \end{aligned}$$

integrating by parts, using the KMS identity $\langle A \gamma _{s}(B) \rangle = \langle \gamma _{s+\beta }(B) A \rangle$ to show the cancellation of the boundary terms, and using the continuity equation (5.3):

5.7 $$\begin{aligned}  &   \eta \langle \textbf{Tj}_{0}(\eta , \mu _{\theta }); j_{\nu }(-\eta , \phi _{\theta ,\ell }) \rangle \nonumber \\  &   \quad = \sum _{k=1,2} \langle \textbf{T}(\text {d}_{x_{k}}j_{k})(\eta , \mu _{\theta }); j_{\nu }(-\eta , \phi _{\theta ,\ell }) \rangle -i\beta \langle [ j_{0}(\mu _{\theta }), j_{\nu }(\phi _{\theta ,\ell }) ] \rangle . \end{aligned}$$

Next, observe that:

5.8 $$\begin{aligned} \begin{aligned} \sum _{k=1,2} (\text {d}_{x_{k}}j_{k})(\eta , \mu _{\theta })&= \theta \int _{-\frac{\beta }{2}}^{\frac{\beta }{2}} d s\, e^{-i\eta s} \sum _{\vec x \in \Lambda _{L} } \sum _{k=1,2}\mu (\theta \vec x) \text {d}_{x_{k}}\gamma _{s}(j_{k,\vec x}) \\&= - \theta \int _{-\frac{\beta }{2}}^{\frac{\beta }{2}} d s\, e^{-i\eta s}\sum _{\vec x \in \Lambda _{L}} \sum _{k=1,2} \big (\text {d}_{x_{k}}\mu (\theta \vec x)\big ) \gamma _{s}(j_{k,\vec x}) \\&\equiv -\theta \int _{-\frac{\beta }{2}}^{\frac{\beta }{2}} d s\, e^{-i\eta s}\sum _{\vec x \in \Lambda _{L}} \sum _{k=1,2} \mu _{\theta ; k}(\theta \vec x) \gamma _{s}(j_{k,\vec x}) \end{aligned} \end{aligned}$$

where in the second equality we summed by parts and used the cylindric boundary conditions, while in the last equality we introduced the function:

5.9 $$\begin{aligned} \mu _{\theta ;k}(\theta \vec x):= {\mu (\theta \vec x) - \mu \big (\theta (\vec x - \vec {e}_{k})\big )}, \end{aligned}$$

which has similar qualitative properties as $\mu _{\theta }(\vec x)$. Thus, we obtained the following identity:

5.10 $$\begin{aligned}  &   \eta \langle \textbf{Tj}_{0}(\eta , \mu _{\theta }); j_{\nu }(-\eta , \phi _{\theta ,\ell }) \rangle =\nonumber \\  &   \quad - \theta \sum _{k=1,2} \langle \textbf{Tj}_{k}(\eta , \mu _{\theta ;k}); j_{\nu }(-\eta , \phi _{\theta ,\ell }) \rangle - i \beta \langle [ j_{0}(\mu _{\theta }), j_{\nu }( \phi _{\theta ,\ell }) ] \rangle . \end{aligned}$$

Eq. (5.10) is the Ward identity for the current-current correlation function.

#### Remark 5.1

The Ward identity (5.10) immediately implies that the response functions are vanishing if one first takes the limit $\theta \rightarrow 0$ and then $\eta \rightarrow 0$.

#### Implications of the Current-Current Ward Identity

The Ward identity (5.10) allows us to derive important constraints on the current-current correlation functions. Let:

5.11 $$\begin{aligned} K^{\beta ,L}_{\mu \nu }(\eta ,\theta ):= \frac{1}{\theta \beta }\langle \textbf{Tj}_{\mu }(\eta , \mu _{\theta ;\mu }); j_{\nu }(-\eta , \phi _{\theta ,\ell }) \rangle _{\beta , \mu , L}, \end{aligned}$$

with $\mu _{\theta ;0} = \mu _{\theta }$. Observe that, for $\eta \in \frac{2\pi }{\beta } \mathbb {N}$:

5.12 $$\begin{aligned} \chi _{\nu ;\ell }^{\beta ,L}(\eta ,\theta ) = K^{\beta ,L}_{0\nu }(\eta ,\theta ); \end{aligned}$$

for general η>0, the identity holds with the additive error $\varepsilon _{\nu ;\ell }^{\beta ,L}(\eta ,\theta )$, as in Eq. (5.1). Suppose that $K^{\beta ,L}_{\mu \nu }(\eta ,\theta )$ satisfies the estimate:

5.13 $$\begin{aligned} | K^{\beta ,L}_{\mu \nu }(\eta ,\theta ) | \le C_{\ell } |\log \theta |, \end{aligned}$$

uniformly in $\eta , \beta , L_{1}, L_{2}$. This bound will be proven later, as a consequence of the result on the two-point function, Theorem 3.1, and of the support properties of the test functions. Also, suppose that the following decomposition holds:

5.14 $$\begin{aligned} K^{\beta ,L}_{\mu \nu }(\eta ,\theta ) = K^{\text {sing}}_{\mu \nu }(\eta ,\theta ) + K^{\text {reg}}_{\mu \nu }(\eta ,\theta ), \end{aligned}$$

where, for α>0:

5.15 $$\begin{aligned} \big |K^{\text {reg}}_{\mu \nu }(\eta ,\theta ) - K^{\text {reg}}_{\mu \nu }(\eta ,\theta ')\big |\le C_{\ell } (|\theta |^{\alpha } + |\theta '|^{\alpha }). \end{aligned}$$

The constant $C_{\ell }$ might depend on ℓ, entering the definition of the test function ϕ, but it is independent from all the other parameters. We shall call $K^{\text {reg}}_{\mu \nu }$ the regular part of $K^{\beta ,L}_{\mu \nu }$, while $K^{\text {sing}}_{\mu \nu }$, which may not satisfy (5.15), is called the singular part. As we will see later, Theorem 3.1 will imply the decomposition (5.14), with a relatively explicit form for $K^{\text {sing}}_{\mu \nu }(\eta ,\theta )$.

Let ν=0. By (5.10), we get:

5.16 $$\begin{aligned} \eta K_{00}^{\text {sing}}(\eta , \theta ) + \eta K_{00}^{\text {reg}}(\eta , \theta ) = - \theta \sum _{k=1,2} K^{\beta ,L}_{k0}(\eta ,\theta ). \end{aligned}$$

This identity implies that:

5.17 $$\begin{aligned} K_{00}^{\text {reg}}(\eta , \theta ) = -K_{00}^{\text {sing}}(\eta ,\theta ) - \frac{\theta }{\eta } \sum _{k=1,2} K^{\beta ,L}_{k0}(\eta ,\theta ). \end{aligned}$$

By (5.15), we have:

5.18 $$\begin{aligned} K_{00}^{\text {reg}}(\eta , \theta ) = K_{00}^{\text {reg}}(\eta , \eta ^{2}) + \mathfrak {e}_{1}(\eta ,\theta ) = - K_{00}^{\text {sing}}(\eta , \eta ^{2}) + \mathfrak {e}_{2}(\eta ,\theta ), \end{aligned}$$

where:

5.19 $$\begin{aligned} | \mathfrak {e}_{i}(\eta ,\theta ) | \le C_{\ell }(|\theta |^{\alpha } + |\eta |^{\alpha }),\qquad i =1,2; \end{aligned}$$

the bound on the error terms follows from (5.13), (5.15) and from (5.17). Therefore, we obtained the following rewriting of the susceptibility, recall Eqs. (5.1), (5.12):

5.20 $$\begin{aligned} \begin{aligned} K^{\beta ,L}_{00}(\eta , \theta )= \big ( K^{\text {sing}}_{00}(\eta , \theta ) - K^{\text {sing}}_{00}(\eta , \eta ^{2})\big ) + E^{\beta ,L}_{00}(\eta ,\theta ), \end{aligned} \end{aligned}$$

where $E^{\beta ,L}_{00}(\eta ,\theta )$ is bounded as in (5.19). This rewriting is particularly useful, due to the relatively explicit form of $K^{\text {sing}}_{00}$ that we will obtain.

A similar rewriting can be obtained for $\chi _{1}(\eta ,\theta )$. Using (5.10), we have:

5.21 $$\begin{aligned} \eta K_{01}^{\text {sing}}(\eta , \theta ) + \eta K_{01}^{\text {reg}}(\eta , \theta ) = - \theta \sum _{j=1,2} K^{\beta ,L}_{j1}(\eta ,\theta ) + \Delta _{1}(\theta ), \end{aligned}$$

where $\Delta _{1}(\theta ) = -\frac{i}{\theta } \langle [ j_{0}(\mu _{\theta }), j_{\nu }( \phi _{\theta ,\ell }) ] \rangle _{\beta ,\mu ,L}$. We claim that:

5.22 $$\begin{aligned} |\Delta _{1}(\theta )| \le C |\theta | \ell , \end{aligned}$$

where ℓ is the width of the strip in proximity of the edge, entering the definition of $\phi _{\theta ,\ell }$. In fact:

5.23 $$\begin{aligned} {[} j_{0}(\mu _{\theta }), j_{\nu }( \phi _{\theta ,\ell }) ] = \theta ^{2} \sum _{\vec x,\vec y } \mu (\theta \vec x) \phi _{\ell }(\theta y_{1}, y_{2}) [ n_{\vec x}, j_{1;\vec y} ]. \end{aligned}$$

Using that $j_{1,x}$ commutes with the number operator, we can rewrite:

5.24 $$\begin{aligned} {[} j_{0}(\mu _{\theta }), j_{\nu }( \phi _{\theta ,\ell }) ] = \theta ^{2} \sum _{\begin{array}{c} \vec x,\vec y \\ \Vert \vec x-\vec y\Vert \le \sqrt{2} \end{array}} \big (\mu (\theta \vec x) - \mu (\theta y)\big ) \phi _{\ell }(\theta y_{1}, y_{2}) [ n_{\vec x}, j_{1;\vec y} ], \end{aligned}$$

where we used that the commutator between the density and the current operator is zero if $\Vert \vec x-\vec y\Vert >\sqrt{2}$. Also, from the regularity properties of $\mu (\cdot )$ we have $|\mu (\theta \vec x) - \mu (\theta \vec y)| \le C \theta \Vert \vec x-\vec y\Vert$. Thus, we have:

5.25 $$\begin{aligned} \big \Vert \big [ j_{0}(\mu _{\theta }), j_{\nu }( \phi _{\theta ,\ell }) \big ] \big \Vert \le K|\theta |^{3} \sum _{\vec y } \phi _{\ell }(\theta y_{1}, y_{2}) \le C\theta ^{2} \ell , \end{aligned}$$

by the support properties of φ. This implies (5.22). Therefore, we can repeat the strategy followed for $\chi _{0}(\eta ,\theta )$, and we obtain:

5.26 $$\begin{aligned} \begin{aligned} K^{\beta ,L}_{01}(\eta , \theta ) = \big ( K^{\text {sing}}_{01}(\eta , \theta ) - K^{\text {sing}}_{01}(\eta , \eta ^{2})\big ) + E^{\beta ,L}_{01}(\eta ,\theta ), \end{aligned} \end{aligned}$$

where $E^{\beta ,L}_{01}(\eta ,\theta )$ satisfies the bound (5.19). Equations (5.20), (5.26) are the main result of this section, and will allow to explicitly compute the response functions.

### Vertex Ward Identity

Let us introduce the vertex function:

5.27 $$\begin{aligned} S_{3;\mu ,\sigma ,\zeta }(\vec {\boldsymbol{w}},\vec {\boldsymbol{x}},\vec {\boldsymbol{y}}):= \big \langle \textbf{T}\, \gamma _{w_0}(j_{\mu , \vec w}); \gamma _{x_{0}}(a_{{\vec x},\sigma }) \gamma _{y_{0}}(a^{*}_{{\vec y},\zeta }) \big \rangle _{\beta , \mu , L}. \end{aligned}$$

We will derive an identity for the vertex function, starting from the lattice continuity equation (5.3) in imaginary times. We compute:

5.28 $$\begin{aligned} \begin{aligned}&i\partial _{w_0} \big \langle \textbf{T}\, \gamma _{w_0}(j_{0, \vec w}); \gamma _{x_{0}}(a_{{\vec x},\sigma }) \gamma _{y_{0}}(a^{*}_{{\vec y},\zeta }) \big \rangle _{\beta , \mu , L} \\&\quad = -\sum _{\nu =1,2} \text {d}_{w_{\nu }} \big \langle \textbf{T}\, \gamma _{w_0}(j_{\nu , \vec w}); \gamma _{x_{0}}(a_{{\vec x},\sigma }) \gamma _{y_{0}}(a^{*}_{{\vec y},\zeta }) \big \rangle _{\beta , \mu , L}\\&\qquad -i \big ( \delta ({\vec {\boldsymbol{x}}} - {\vec {\boldsymbol{w}}})- \delta ({\vec {\boldsymbol{y}}} - {\vec {\boldsymbol{w}}}) \big ) \langle \textbf{T}\gamma _{x_0}(a_{{\vec x},\sigma }) \gamma _{y_0}(a^{*}_{{\vec y},\zeta }) \rangle _{\beta , \mu , L}. \end{aligned} \end{aligned}$$

Eq. (5.28) is the vertex Ward identity. Summing over $w_{2}$ from 1 to $L_{2}$ and using the Dirichlet boundary conditions, omitting from now on the $\beta ,\mu ,L$ labels:

5.29 $$\begin{aligned} \begin{aligned}&i\partial _{w_0} \big \langle \textbf{T}\, \gamma _{w_0}(j_{0, w_{1}}); \gamma _{x_{0}}(a_{{\vec x},\sigma }) \gamma _{y_{0}}(a^{*}_{{\vec y},\zeta }) \big \rangle = -\text {d}_{w_{1}}\big \langle \textbf{T}\, \gamma _{w_0}(j_{1, w_{1}}); \gamma _{x_{0}}(a_{{\vec x},\sigma }) \gamma _{y_{0}}(a^{*}_{{\vec y},\zeta }) \big \rangle \\&\qquad \qquad \qquad \qquad \qquad - i\big (\delta ({{\boldsymbol{x}}} - {{\boldsymbol{w}}})- \delta ({\boldsymbol{y}} -{\boldsymbol{w}}) \big ) \big \langle \textbf{T}\gamma _{x_0}(a_{\vec x, \sigma }) \gamma _{y_0}(a^{*}_{\vec y,\zeta }) \big \rangle , \end{aligned} \nonumber \\ \end{aligned}$$

where we recall:

5.30 $$\begin{aligned} j_{\nu ,x_{1}}:= \sum _{x_{2} = 1}^{L_{2}} j_{\nu ,\vec x}. \end{aligned}$$

Next, let us introduce the partial Fourier transform of the vertex function:

5.31 $$\begin{aligned} \begin{aligned}&{\hat{S}}_{3; \mu ,\sigma ,\zeta }(\boldsymbol{p}, \boldsymbol{k}, \boldsymbol{h}; x_{2}, y_{2}):= \\&\frac{1}{\beta L_{1}} \int _{[0,\beta ]^{3}}\mathrm d w_0 d x_0 d y_0\Big (\sum _{w_{1}, x_{1}, y_{1}} e^{-i\boldsymbol{p} \cdot \boldsymbol{w} - i\boldsymbol{k} \cdot \boldsymbol{x} + i\boldsymbol{h} \cdot \boldsymbol{y} } \big \langle \textbf{T}\, \gamma _{w_0}(j_{\mu , w_{1}}); \gamma _{x_{0}}(a_{{\vec x},\sigma }) ; \gamma _{y_{0}}(a^{*}_{{\vec y},\zeta }) \big \rangle \Big )\;, \end{aligned} \nonumber \\ \end{aligned}$$

and the partial Fourier transform of the two-point function:

5.32 $$\begin{aligned} {\hat{S}}_{2;\sigma ,\zeta }(\boldsymbol{k}, \boldsymbol{h}; x_{2}, y_{2}):= \frac{1}{\beta L_{1}} \int _{[0,\beta ]^{2}}\mathrm dx_{0}\mathrm dy_{0} \sum _{x_{1}, y_{1}} e^{-i\boldsymbol{k} \cdot \boldsymbol{x} + i\boldsymbol{h} \cdot \boldsymbol{y} }\langle \textbf{T}\gamma _{x_{0}}(a_{{\vec x},\sigma }) \gamma _{y_{0}}(a^{*}_{{\vec y},\zeta }) \big \rangle .\nonumber \\ \end{aligned}$$

Then, the vertex Ward identity (5.28) can be rewritten as

5.33 $$\begin{aligned} \begin{aligned}&-p_{0} {\hat{S}}_{3; 0,\sigma ,\zeta }(\boldsymbol{p}, \boldsymbol{k}, \boldsymbol{h}; x_{2}, y_{2}) + (1 - e^{-ip_{1}}) {\hat{S}}_{3; 1,\sigma ,\zeta }(\boldsymbol{p}, \boldsymbol{k}, \boldsymbol{h}; x_{2}, y_{2}) \\&\qquad =i {\hat{S}}_{2;\sigma ,\zeta }(\boldsymbol{k}, \boldsymbol{h} -\boldsymbol{p}; x_{2}, y_{2})- i {\hat{S}}_{2;\sigma ,\zeta }(\boldsymbol{k}+ \boldsymbol{p}, \boldsymbol{h}; x_{2}, y_{2}). \end{aligned} \end{aligned}$$

In order to extract useful information from this identity, it will be convenient to rewrite the current operator $j_{1,z_{1}}$ as, recalling (2.39) and (5.30), and using the Dirichlet boundary conditions:

5.34 $$\begin{aligned} j_{1,z_{1}} = \sum _{z_{2} = 1}^{L_{2}} \big ( j_{\vec z,\vec z+\vec e_{1}} + j_{\vec z, \vec z+ \vec e_{1} - \vec e_{2}} + j_{\vec z, \vec z+\vec e_{1} + \vec e_{2}} \big ). \end{aligned}$$

Taking the Fourier transform:

5.35 $$\begin{aligned} {\hat{j}}_{1,p_{1}}= &   \sum _{z_{1} = 1}^{L_{1}} e^{-ip_1 z_{1}} j_{1,z_{1}} \nonumber \\= &   \frac{i}{L_{1}} \sum _{k_{1} } \Big ( e^{ik_{1}} \big ( {\hat{a}}^{*}_{k_1-p_{1}}, H(-\vec e_{1}) {\hat{a}}_{k_1} \big ) - e^{-i (k_{1} - p_{1})} \big ( {\hat{a}}^{*}_{k_{1} - p_{1}}, H(\vec e_{1}) {\hat{a}}_{k_{1}} \big )\Big ), \nonumber \\ \end{aligned}$$

where $H(\vec e_{1})$ is the operator with kernel $H_{\sigma \zeta }(\vec e_{1}; x_{2}, y_{2})$, and we used the notation $( f, A g ):= \sum _{\sigma ,\zeta } \sum _{x_{2}, y_{2}} f_{\sigma ,x_{2}} A_{\sigma \zeta }(x_{2},y_{2}) g_{\zeta ,y_{2}}$. Recall that the Hamiltonian is translation invariant in the $x_{1}$ direction, $H_{\sigma \zeta }(\vec e_{1}; x_{2}, y_{2}) \equiv H_{\sigma \zeta }\big ((x_{1} + 1, x_{2}); (x_{1}, y_{2})\big )$. Observe that, for $p_{1} = 0$:

5.36 $$\begin{aligned} {\hat{j}}_{1;0} = \frac{1}{L_{1}} \sum _{k_{1} } \Big ( {\hat{a}}^{*}_{k_{1}}, \big (\partial _{k_{1}} {\hat{H}}(k_{1})\big ) {\hat{a}}_{k_{1}} \Big ), \end{aligned}$$

where:

5.37 $$\begin{aligned} \partial _{k_{1}} {\hat{H}}(k_{1}; x_{2}, y_{2}) = \partial _{k_{1}} \sum _{|x_{1}|\le 1} e^{-ik_{1} x_{1}} H(x_{1}, 0; x_{2}, y_{2}). \end{aligned}$$

#### Implications of the Vertex Ward Identity

Here we shall use the vertex Ward identity (5.33) in order to prove the relations (3.8) between the renormalized parameters appearing in the leading contribution to the two-point function, Eqs. (3.3), (3.4). These relations will be important in the computations of the response functions. To do this, we will resolve the main contribution to the vertex function for small external momenta. We will then compare the resulting expression with the two-point function at low momenta, via the Ward identity (5.33).

Let us rewrite the operators ${\hat{j}}_{0,p_{2}}$, ${\hat{j}}_{1,p_{1}}$ as:

5.38 $$\begin{aligned} \begin{aligned} {\hat{j}}_{\mu ,p_{1}}&= \frac{1}{L_{1}} \sum _{k_{1}} \big ( {\hat{a}}^{*}_{k_{1} - p_{1}}, {\hat{J}}_{\mu }(k_{1},p_{1}) {\hat{a}}_{k_{1}} \big ), \\ {\hat{J}}_{0;\sigma ,\zeta }(k_{1},p_{1};x_{2}, y_{2})&= \delta _{\sigma ,\zeta }\delta _{x_{2},y_{2}},\\ {\hat{J}}_{1;\sigma ,\zeta }(k_{1},p_{1};x_{2},y_{2})&= i e^{ik_{1}} H_{\sigma ,\zeta }(-\vec e_{1}; x_{2},y_{2}) - i e^{-i(k_{1}-p_{1})} H_{\sigma ,\zeta }(\vec e_{1}; x_{2},y_{2}). \end{aligned} \end{aligned}$$

In what follows, we will choose external momenta $\boldsymbol{k}, \boldsymbol{p}$ such that, for some M>1:

5.39 $$\begin{aligned} \big |k_{1} - k_{F}^{+}(\lambda )\big |^{M} \le |k_{0}|\le |k_{1} - k_{F}^{+}(\lambda )|,\quad |p_{1}| \le \frac{1}{2}| k_{1} - k_{F}^{+}(\lambda ) |,\qquad |p_{0}| \le \frac{1}{2}|k_{0}|. \nonumber \\ \end{aligned}$$

We recall that $k_{F}^{+}$ is the Fermi momentum of the edge mode localized in proximity of the boundary at $x_{2} = 0$.

##### Lemma 5.2

(Implication of the vertex Ward identity). Under the same assumptions of Theorem 3.1, the following holds. Let:

5.40 $$\begin{aligned} \zeta _{\mu ,+}:= \sum _{n} \langle Z_{-n,+}, J_{\mu }(k_{F}^{+}(\lambda ) + n\alpha ,0) Z_{-n,+} \rangle , \end{aligned}$$

and let $v_{\mu ,+}$ be the parameters appearing in the asymptotics of the two-point function (3.4). Then:

5.41 $$\begin{aligned} \zeta _{0,+} = v_{0,+},\qquad \zeta _{1,+} = v_{1,+}. \end{aligned}$$

Lemma 5.2 proves the relations (3.8), and concludes the proof of Theorem 3.1 about the two-point function. These relations will play an important role in the computation of the transport coefficients, discussed in the next section. The analysis can be adapted to prove the relations $\zeta _{0,-} = v_{0,-}$, $\zeta _{1,-} = v_{1,-}$, which however will not be needed in the following.

The proof of Lemma 5.2 is based on the two following propositions. In what follows, we shall use the notation ${\boldsymbol{q}} = {\boldsymbol{k}} - {\boldsymbol{k}}_{F}^{+}$.

##### Proposition 5.3

(Asymptotics of the vertex function). Assume (5.39). Then, for $\Vert {\boldsymbol{k}} - {\boldsymbol{k}}_{F}^{+}(\lambda ) \Vert \ll 1$, $\Vert {\boldsymbol{p}} \Vert \ll 1$ and $L_{2}$ large enough:

5.42 $$\begin{aligned} \begin{aligned} {\hat{S}}_{3; \mu ,\sigma ,\zeta }(\boldsymbol{p}, \boldsymbol{k}, \boldsymbol{k} + {\boldsymbol{p}}; x_{2}, y_{2})&= Z_{0,+,\sigma }(x_{2}) \overline{Z_{0,+,\zeta }(y_{2})} g_{+;\textrm{s}}({\boldsymbol{q}}) g_{+;\textrm{s}}({\boldsymbol{q}} + {\boldsymbol{p}}) \zeta _{\mu ,+} \\&\quad + R^{\text {tot}}_{3; \mu ,\sigma ,\zeta }(\boldsymbol{p}, \boldsymbol{k}, \boldsymbol{k} + {\boldsymbol{p}}; x_{2}, y_{2}), \end{aligned} \end{aligned}$$

where $\zeta _{\mu ,+}$ is an in (5.40), and:

5.43 $$\begin{aligned}  &   \sum _{x_{2}, y_{2}} \big | Z_{0,+,\sigma }(x_{2})\big | \big | Z_{0,+,\zeta }(y_{2})\big | \Big |g^{-1}_{+;\textrm{s}}({\boldsymbol{q}})\Big | \Big |g^{-1}_{+;\textrm{s}}({\boldsymbol{q}} + {\boldsymbol{p}})\Big | \Big |R^{\text {tot}}_{3; \mu ,\sigma ,\zeta }(\boldsymbol{p}, \boldsymbol{k}, \boldsymbol{k} + {\boldsymbol{p}}; x_{2}, y_{2})\Big | \nonumber \\  &   \quad = \textrm{o}(1). \end{aligned}$$

##### Proposition 5.4

(Asymptotics of the two-point function). Assume (5.39), and let 0<ξ<1. Then, for $\Vert {\boldsymbol{k}} - {\boldsymbol{k}}_{F}^{+}(\lambda ) \Vert \ll 1$ and $L_{2}$ large enough:

5.44 $$\begin{aligned} {\hat{S}}_{2;\sigma ,\zeta }(\boldsymbol{k},{\boldsymbol{k}}; x_{2}, y_{2}) = Z_{0,+,\sigma }(x_{2}) \overline{Z_{0,+,\zeta }(y_{2})} g_{+;\textrm{s}}({\boldsymbol{q}}) + {\hat{R}}^{\text {tot}}_{\sigma ,\zeta }({\boldsymbol{k}}; x_{2}, y_{2}), \end{aligned}$$

with:

5.45 $$\begin{aligned}  &   \Big | \text {d}_{k_{0}}^{n_{0}} \text {d}_{k_{1}}^{n_{1}} {\hat{R}}^{\text {tot}}_{\sigma ,\zeta }({\boldsymbol{k}}; x_{2}, y_{2}) \Big | \nonumber \\  &   \quad \le C_{n_{0},n_{1}}\frac{e^{-{\tilde{c}}|x_{2} - y_{2}|}}{\Vert {\boldsymbol{k}} - {\boldsymbol{k}}_{F}^{+} \Vert ^{n_{0} + n_{1} + 1-\xi }} + \sum _{\begin{array}{c} \omega _{1},\omega _{2} \\ (\omega _{1},\omega _{2}) \ne (+,+) \end{array}} \!\!\! C_{n_{0},n_{1}}\frac{e^{-c(|x_{2}|_{\omega _{1}} + |y_{2}|_{\omega _{2}})}}{|k_{0}|^{n_{0} + n_{1} + 1}}. \end{aligned}$$

Before discussing the proofs of these propositions, let us see how they imply Lemma 5.2.

##### Proof of Lemma 5.2

Suppose that the assumptions of Propositions 5.3, 5.4 hold true. Consider the left-hand side of the Ward identity (5.33), for external momenta satisfying (5.39). We have, from Proposition 5.3,

5.46 $$\begin{aligned} \begin{aligned}&\sum _{\begin{array}{c} x_{2}, y_{2} \\ \sigma , \zeta \end{array}} \overline{Z_{0,+,\sigma }(x_{2})} Z_{0,+,\zeta }(y_{2}) \\&\quad \cdot \Big (-p_{0}{\hat{S}}_{3; 0,\sigma ,\zeta }(\boldsymbol{p}, \boldsymbol{k}, \boldsymbol{k} + {\boldsymbol{p}}; x_{2}, y_{2}) + (1 - e^{-ip_{1}}) {\hat{S}}_{3; 1,\sigma ,\zeta }(\boldsymbol{p}, \boldsymbol{k}, \boldsymbol{k} + {\boldsymbol{p}}; x_{2}, y_{2})\Big )\\&= \Vert Z_{0,+} \Vert ^{4} (-p_{0} \zeta _{0,+} + ip_{1}\zeta _{1,+}) g_{+;\textrm{s}}({\boldsymbol{q}}) g_{+;\textrm{s}}({\boldsymbol{q}} + {\boldsymbol{p}}) + \mathcal {E}_{1}({\boldsymbol{k}},{\boldsymbol{p}}) \end{aligned} \nonumber \\ \end{aligned}$$

where the error term satisfies:

5.47 $$\begin{aligned} \big | g^{-1}_{+;\textrm{s}}({\boldsymbol{q}})\big | \big |g^{-1}_{+;\textrm{s}}({\boldsymbol{q}} + {\boldsymbol{p}})\big | \big |\mathcal {E}_{1}({\boldsymbol{k}},{\boldsymbol{p}})\big | = o(\Vert \textbf{p}\Vert ). \end{aligned}$$

Next, consider the right-hand side of the Ward identity (5.33). We have, by Proposition 5.4

5.48 $$\begin{aligned} \begin{aligned}&\sum _{\begin{array}{c} x_{2}, y_{2} \\ \sigma , \zeta \end{array}} \overline{Z_{0,+,\sigma }(x_{2})} Z_{0,+,\zeta }(y_{2}) \Big (i {\hat{S}}_{2;\sigma ,\zeta }(\boldsymbol{k}, \boldsymbol{k}; x_{2}, y_{2})- i {\hat{S}}_{2;\sigma ,\zeta }(\boldsymbol{k}+ \boldsymbol{p}, \boldsymbol{k}+ \boldsymbol{p}; x_{2}, y_{2})\Big ) \\&\quad = \Vert Z_{0,+} \Vert ^{4} \big ( g_{+;\textrm{s}}({\boldsymbol{q}}) - g_{+;\textrm{s}}({\boldsymbol{q}} + {\boldsymbol{p}}) \big ) + \mathcal {E}_{2}({\boldsymbol{k}},{\boldsymbol{p}}), \end{aligned} \end{aligned}$$

where the error term satisfies:

5.49 $$\begin{aligned} \big | g^{-1}_{+;\textrm{s}}({\boldsymbol{q}})\big | \big |g^{-1}_{+;\textrm{s}}({\boldsymbol{q}} + {\boldsymbol{p}})\big | \big |\mathcal {E}_{2}({\boldsymbol{k}},{\boldsymbol{p}})\big | = o(\Vert \textbf{p}\Vert ). \end{aligned}$$

Here we used that the difference of the error terms in (5.44) can be estimated as $\Vert {\boldsymbol{p}}\Vert$ times the derivative of the error term, which is estimated in (5.45). Then, observing that, for momenta small enough so that the cutoff function in the definition of $g_{+;\textrm{s}}$ is 1,

5.50 $$\begin{aligned} g_{+;\textrm{s}}({\boldsymbol{q}}) - g_{+;\textrm{s}}({\boldsymbol{q}} + {\boldsymbol{p}}) = (i v_{0,+} p_{0} + v_{1,+} p_{1}) g_{+;\textrm{s}}({\boldsymbol{q}}) g_{+;\textrm{s}}({\boldsymbol{q}} + {\boldsymbol{p}}), \end{aligned}$$

and using that $\Vert Z_{0,+} \Vert ^{2} = 1 + O(\lambda )$, we have, equating (5.46), (5.48):

5.51 $$\begin{aligned}  &   (-p_{0} \zeta _{0,+} + ip_{1}\zeta _{1,+}) \nonumber \\  &   \quad = (-v_{0,+} p_{0} + i v_{1,+} p_{1}) + g_{+;\textrm{s}}({\boldsymbol{q}})^{-1} g_{+;\textrm{s}}({\boldsymbol{q}} + {\boldsymbol{p}})^{-1}\Big (\mathcal {E}_{2}({\boldsymbol{k}},{\boldsymbol{p}}) - \mathcal {E}_{1}({\boldsymbol{k}},{\boldsymbol{p}})\Big ). \nonumber \\ \end{aligned}$$

The relations (5.41) follows from (5.51) by choosing $|p_{1}| \ll |p_{0}|$, resp. $|p_{0}| \ll |p_{1}|$.

We are left with proving Propositions 5.3, 5.4.

##### Proof of Proposition 5.3

By the Wick rule, the vertex function in the left-hand side of (5.33) can be written in terms of the two-point function as:

5.52 $$\begin{aligned} \begin{aligned} {\hat{S}}_{3; \mu ,\sigma ,\zeta }(\boldsymbol{p}, \boldsymbol{k}, \boldsymbol{h}; x_{2}, y_{2})&= \sum _{\boldsymbol{r}} \sum _{\rho ,\varrho } \sum _{z_{2},w_{2}}\big ({\hat{J}}_{\mu ;\rho ,\varrho }(r_{1},p_{1};z_{2},w_{2}) \\&\qquad \cdot {\hat{S}}_{2;\sigma ,\rho } (\boldsymbol{k},\boldsymbol{r} - \boldsymbol{p}, x_{2},z_{2}) {\hat{S}}_{2;\varrho ,\zeta } ( \boldsymbol{r}, \boldsymbol{h}, w_{2}, y_{2} )\big ). \end{aligned} \end{aligned}$$

Recall the decomposition (3.3) of the two-point function. In Fourier space, it reads:

5.53 $$\begin{aligned} {\hat{S}}_{2;\sigma ,\zeta } (\boldsymbol{k}, {\boldsymbol{h}}; x_{2}, y_{2}) = {\hat{S}}^{\textrm{s}}_{2;\sigma ,\zeta } (\boldsymbol{k}, {\boldsymbol{h}}; x_{2}, y_{2}) + {\hat{R}}_{\sigma ,\zeta }({\boldsymbol{k}}, {\boldsymbol{h}}; x_{2}, y_{2}); \end{aligned}$$

both sides can be non-zero only if ${\boldsymbol{h}} = {\boldsymbol{k}} + n{\boldsymbol{\alpha }}$ for some integer *n*, and we set ${\hat{S}}^{\textrm{s}}_{2;\sigma ,\zeta } (\boldsymbol{k}, {\boldsymbol{k}} + n{\boldsymbol{\alpha }}; x_{2}, y_{2}) \equiv {\hat{S}}^{\textrm{s}}_{2;n,\sigma ,\zeta } (\boldsymbol{k}; x_{2}, y_{2})$. The main term in (5.53) can be written as, see Eq. (4.186):

5.54 $$\begin{aligned} {\hat{S}}^{\textrm{s}}_{2;n,\sigma ,\zeta } \big (\boldsymbol{k}; x_{2}, y_{2}\big ) = \sum _{m,\omega } Z_{m,\omega ,\sigma }(x_{2}) g_{\omega ;\textrm{s}}({\boldsymbol{q}} + m{\boldsymbol{\alpha }}) \overline{Z_{-n+m,\omega ,\zeta }(y_{2})}, \end{aligned}$$

where ${\boldsymbol{q}} = {\boldsymbol{k}} - {\boldsymbol{k}}_{F}^{\omega }(\lambda )$. Concerning the term ${\hat{R}}$ in (5.53), it is not explicit; the only information we will actually need is the following estimate in momentum space, proved in Appendix D:

5.55 $$\begin{aligned} \begin{aligned} \big | \text {d}_{k_{0}}^{n_{0}} \text {d}_{k_{1}}^{n_{1}} {\hat{R}}_{\sigma ,\zeta }({\boldsymbol{k}}, {\boldsymbol{k}} + n{\boldsymbol{\alpha }}; x_{2}, y_{2}) \big |&\le \frac{C_{n_{0},n_{1}} |\lambda |^{\delta _{n\ne 0}} e^{-\frac{c}{16}|n|} e^{-{\tilde{c}}|x_{2} - y_{2}|}}{\Vert {\boldsymbol{k}} - {\boldsymbol{k}}_{F}^{+} \Vert ^{n_{0} + n_{1} + 1-\xi }}\\  &\quad + \sum _{\begin{array}{c} \omega _{1},\omega _{2} \\ (\omega _{1},\omega _{2}) \ne (+,+) \end{array}} \!\!\! C_{n_{0},n_{1}}\frac{e^{-\frac{c}{16}|n|} e^{-c(|x_{2}|_{\omega _{1}} + |y_{2}|_{\omega _{2}})}}{|k_{0}|^{n_{0} + n_{1} + 1-\xi }}, \end{aligned} \nonumber \\ \end{aligned}$$

for values of the momentum ${\boldsymbol{k}}$ safisfying the assumptions of the proposition, Eq. (5.39). We rewrite (5.52) as, using that the only nonzero terms in the ${\boldsymbol{r}}$ sum are those such that ${\boldsymbol{r}} - {\boldsymbol{p}}= {\boldsymbol{k}} + n{\boldsymbol{\alpha }}$, and choosing ${\boldsymbol{h}} = {\boldsymbol{k}} + {\boldsymbol{p}}$:

5.56 $$\begin{aligned} \begin{aligned} {\hat{S}}_{3; \mu ,\sigma ,\zeta }(\boldsymbol{p}, \boldsymbol{k}, \boldsymbol{k} + {\boldsymbol{p}}; x_{2}, y_{2})&= \sum _{n} \sum _{\rho ,\varrho } \sum _{z_{2},w_{2}}\big ({\hat{J}}_{\mu ;\rho ,\varrho }(k_{1} + p_{1}+ n\alpha , p_{1};z_{2},w_{2}) \\&\quad \cdot {\hat{S}}_{2;n,\sigma ,\rho } (\boldsymbol{k}, x_{2}, z_{2}) {\hat{S}}_{2;-n,\varrho ,\zeta } ( \boldsymbol{k} + {\boldsymbol{p}} + n{\boldsymbol{\alpha }}, w_{2}, y_{2} )\big ). \end{aligned} \end{aligned}$$

Next, we write:

5.57 $$\begin{aligned}  &   {\hat{S}}_{3; \mu ,\sigma ,\zeta }(\boldsymbol{p}, \boldsymbol{k}, \boldsymbol{k} + {\boldsymbol{p}}; x_{2}, y_{2}) \nonumber \\  &   \quad = {\hat{S}}^{\textrm{s}}_{3; \mu ,\sigma ,\zeta }(\boldsymbol{p}, \boldsymbol{k}, \boldsymbol{k} + {\boldsymbol{p}}; x_{2}, y_{2}) + {\hat{R}}_{3; \mu ,\sigma ,\zeta }\big (\boldsymbol{p}, \boldsymbol{k}, \boldsymbol{k} + {\boldsymbol{p}}; x_{2}, y_{2}\big ), \end{aligned}$$

where ${\hat{S}}^{\textrm{s}}_{3}$ is obtained from ${\hat{S}}_{3}$ replacing all ${\hat{S}}_{2}$ with ${\hat{S}}^{\textrm{s}}_{2}$, while ${\hat{R}}_{3}$ collects all the error terms, where there is at least one ${\hat{R}}$. These last terms are subleading with respect to ${\hat{S}}^{\textrm{s}}_{3}$, due to the bound (5.55). In particular, the term ${\hat{R}}_{3; \mu ,\sigma ,\zeta }$ satisfies the estimate (5.43).

Using (5.54), we have:

5.58 $$\begin{aligned} \begin{aligned}&\sum _{n}{\hat{S}}^{\text {s}}_{2;n,\sigma ,\rho } \big (\boldsymbol{k}, x_{2}, z_{2}\big ) {\hat{S}}^{\text {s}}_{2;-n,\varrho ,\zeta } \big ( \boldsymbol{k} + {\boldsymbol{p}} + n{\boldsymbol{\alpha }}, w_{2}, y_{2} \big ) \\&\quad = \sum _{n} \sum _{m_{1}, m_{2}} \sum _{\omega _{1}, \omega _{2}}Z_{m_{1},\omega _{1},\sigma }(x_{2}) g_{\omega _{1};\textrm{s}}({\boldsymbol{q}} + m_{1}{\boldsymbol{\alpha }} + s_{+\omega _{1}}) \overline{Z_{-n+m_{1},\omega _{1},\rho }(z_{2})} \\&\qquad \cdot Z_{m_{2},\omega _{2},\varrho }(w_{2}) g_{\omega _{2};\textrm{s}}\big ({\boldsymbol{q}} + {\boldsymbol{p}} + (m_{2} + n){\boldsymbol{\alpha }} + s_{+\omega _{2}}\big ) \overline{Z_{n+m_{2},\omega _{2},\zeta }(y_{2})}. \end{aligned} \nonumber \\ \end{aligned}$$

We rewrite:

5.59 $$\begin{aligned} (5.58) = \text {I} + \text {II} + \text {III}, \end{aligned}$$

where: the terms $\text {I}$, $\text {II}$ collect the contributions to (5.58) with $\omega _{1} = \omega _{2} = +$, and $\text {III}$ all the others. Specifically, the term $\text {I}$ collects the contribution of the indices $n,m_{1},m_{2}$ such that $m_{1} = 0$ and $n+m_{2} = 0$:

5.60 $$\begin{aligned} \text {I} = \sum _{n} Z_{0,+,\sigma }(x_{2}) g_{+;\textrm{s}}({\boldsymbol{q}}) \overline{Z_{-n,+,\rho }(z_{2})} Z_{-n,+,\varrho }(w_{2}) g_{+;\textrm{s}}({\boldsymbol{q}} + {\boldsymbol{p}}) \overline{Z_{0,+,\zeta }(y_{2})}; \nonumber \\ \end{aligned}$$

instead, the term $\text {II}$ collects the combinations $m_{1}\ne 0$ and/or $n+m_{2} \ne 0$. Before discussing them, let us consider $\text {III}$. From the explicit form of the relativistic propagator and the decay of the *Z*’s in the second direction, we get:

5.61 $$\begin{aligned} \big |\text {III}\big |\le \sum _{\begin{array}{c} \omega _{1}, \omega _{2} \\ (\omega _{1},\omega _{2}) \ne (+,+) \end{array}}\frac{e^{-c(|x_{2}|_{\omega _{1}} + |z_{2}|_{\omega _{1}} + |w_{2}|_{\omega _{2}} +|y_{2}|_{\omega _{2}})}}{|k_{0}| |k_{0} + p_{0}|}. \end{aligned}$$

In particular, the term $\text {III}$ satisfies the estimate (5.43).

**Analysis of** $\text {II}$. Let us consider the contribution coming from $m_{2} + n\ne 0$:

5.62 $$\begin{aligned} \begin{aligned}&\text {II}^{\text {A}}:= \sum _{\begin{array}{c} n,m_{1},m_{2} \\ n+m_{2} \ne 0 \end{array}} Z_{m_{1},+,\sigma }(x_{2}) g_{+;\textrm{s}}({\boldsymbol{q}} + m_{1}{\boldsymbol{\alpha }}) \overline{Z_{-n+m_{1},+,\rho }(z_{2})} \\&\quad \qquad \cdot Z_{m_{2},+,\varrho }(w_{2}) g_{+;\textrm{s}}\big ({\boldsymbol{q}} + {\boldsymbol{p}} + (m_{2} + n){\boldsymbol{\alpha }}\big ) \overline{Z_{n+m_{2},+,\zeta }(y_{2})}, \end{aligned} \nonumber \\ \end{aligned}$$

we rewrite the sum over $m_{1}, m_{2}, n$ as:

5.63 $$\begin{aligned} \sum _{m_{1}, m_{2}, n:\, n+m_{2} \ne 0} (\cdots ) = \sum _{\begin{array}{c} m_{1}, m_{2}, n:\, n+m_{2} \ne 0 \\ m_{1} = 0 \end{array}} (\cdots ) + \sum _{\begin{array}{c} m_{1}, m_{2}, n:\, n+m_{2} \ne 0 \\ m_{1} \ne 0 \end{array}} (\cdots ). \end{aligned}$$

Consider the first sum. We further rewrite it as:

5.64 $$\begin{aligned} \sum _{\begin{array}{c} m_{1}, m_{2}, n:\, n+m_{2} \ne 0 \\ m_{1} = 0 \end{array}} (\cdots )= &   \sum _{\begin{array}{c} m_{1}, m_{2}, n:\, n+m_{2} \ne 0 \\ m_{1} = 0,\; \Vert (n + m_{2}) {\boldsymbol{\alpha }} \Vert \le \sqrt{\Vert {\boldsymbol{q}} + {\boldsymbol{p}}\Vert } \end{array}} (\cdots )\; + \sum _{\begin{array}{c} m_{1}, m_{2}, n:\, n+m_{2} \ne 0 \\ m_{1} = 0,\; \Vert (n + m_{2}) {\boldsymbol{\alpha }} \Vert > \sqrt{\Vert {\boldsymbol{q}} + {\boldsymbol{p}}\Vert } \end{array}} (\cdots ); \nonumber \\ \end{aligned}$$

Let us denote by $\text {II}^{\text {A}}_{1}$ and $\text {II}^{\text {A}}_{2}$ the two corresponding contributions to $\text {II}^{\text {A}}$. Consider $\text {II}^{\text {A}}_{1}$:

5.65 $$\begin{aligned} \begin{aligned} \text {II}^{\text {A}}_{1}&= \sum _{\begin{array}{c} m_{2}, n:\, n+m_{2} \ne 0 \\ \Vert (n + m_{2}) {\boldsymbol{\alpha }} \Vert \le \sqrt{\Vert {\boldsymbol{q}} + {\boldsymbol{p}}\Vert } \end{array}} Z_{0,+,\sigma }(x_{2}) g_{+;\textrm{s}}({\boldsymbol{q}}) \overline{Z_{-n,+,\rho }(z_{2})} \\&\quad \cdot Z_{m_{2},+,\varrho }(w_{2}) g_{+;\textrm{s}}\big ({\boldsymbol{q}} + {\boldsymbol{p}} + (m_{2} + n) {\boldsymbol{\alpha }}\big ) \overline{Z_{n + m_{2},+,\zeta }(y_{2})}. \end{aligned} \end{aligned}$$

By the Diophantine condition (2.9), since $n+m_{2} \ne 0$ we have:

5.66 $$\begin{aligned} \Vert (n + m_{2}) {\boldsymbol{\alpha }} \Vert \le \sqrt{\Vert {\boldsymbol{q}} + {\boldsymbol{p}}\Vert } \Rightarrow | n + m_{2} | \ge \frac{C}{\Vert {\boldsymbol{q}} + {\boldsymbol{p}} \Vert ^{\tau /2}}, \end{aligned}$$

therefore, by the estimate (3.7) for $Z_{n+m_{2}}$:

5.67 $$\begin{aligned} | Z_{n+m_{2},+,\zeta }(y_{2}) | \le C |\lambda |^{1-\delta _{n+m_{2},0}} e^{-\frac{c}{2} |n+m_{2}|} e^{-c y_{2}} \exp \Big (-\frac{c}{2\Vert {\boldsymbol{q}} + {\boldsymbol{p}}\Vert ^{\tau /2}}\Big ). \end{aligned}$$

Strictly speaking, the bound (5.66) only holds for $|n + m_{2}| \le L_{1}/2$, by the approximate Diophantine condition. In the case $|n + m_{2}| > L_{1}$, we use the exponential decay in $|n+m_{2}|$ of $Z_{n+m_{2}}$ to make sure that the bound (5.67) holds, possibly with a different c>0. This follows from $||{\boldsymbol{q}} + {\boldsymbol{p}}|| > 1/\beta$, taking $L_1\ge C\beta$ with *C* suitably large.

This bound allows us to show that $\text {II}^{\text {A}}_{1}$ is small. In fact:

5.68 $$\begin{aligned} \begin{aligned}&|g_{+;\textrm{s}}({\boldsymbol{q}})| \big | g_{+;\textrm{s}}({\boldsymbol{q}} + {\boldsymbol{p}} + (m_{2} + n) {\boldsymbol{\alpha }})\big | \Big | \overline{Z_{n + m_{2},+,\zeta }(y_{2})} \Big | \\&\qquad \le K |\lambda |^{1-\delta _{n + m_{2},0}} e^{-\frac{c}{2} |n + m_{2}|} e^{-c y_{2}} \frac{1}{|k_{0}| |k_{0} - p_{0}|} \exp \Big (-\frac{c}{2\Vert {\boldsymbol{q}} + {\boldsymbol{p}}\Vert ^{\tau /2}}\Big ). \end{aligned} \end{aligned}$$

Recalling the conditions (5.39), we get:

5.69 $$\begin{aligned} \begin{aligned}&|g_{+;\textrm{s}}({\boldsymbol{q}})| \big | g_{+;\textrm{s}}\big ({\boldsymbol{q}} + {\boldsymbol{p}} + (m_{2} + n) {\boldsymbol{\alpha }}\big )\big | \Big | \overline{Z_{n + m_{2},+,\zeta }(y_{2})} \Big | \\&\qquad \le K_{M} |\lambda |^{1-\delta _{n+m_{2},0}} e^{-\frac{c}{2} |n + m_{2}|} e^{-c y_{2}} \frac{1}{\Vert {\boldsymbol{q}}\Vert ^{2M}} \exp \Big (-\frac{c}{2\Vert {\boldsymbol{q}} \Vert ^{\tau /2}}\Big ) \\&\qquad \le C_{r} |\lambda |^{1-\delta _{n+m_{2},0}} e^{-\frac{c}{2} |n + m_{2}|} e^{-c y_{2}} \Vert {\boldsymbol{q}}\Vert ^{r},\qquad \text {for any } r\in \mathbb {N}. \end{aligned} \end{aligned}$$

In particular, the term $\text {II}^{\text {A}}_{1}$ is bounded as:

5.70 $$\begin{aligned} \big | \text {II}^{\text {A}}_{1} \big | \le C_{r} e^{-c (x_{2} + y_{2} + w_{2} + z_{2})} \Vert {\boldsymbol{q}}\Vert ^{r}. \end{aligned}$$

Consider now the term $\text {II}^{\text {A}}_{2}$,

5.71 $$\begin{aligned} \begin{aligned} \text {II}^{\text {A}}_{2}&= \sum _{\begin{array}{c} m_{2}, n:\, n+m_{2} \ne 0 \\ \Vert (n + m_{2}) {\boldsymbol{\alpha }} \Vert > \sqrt{\Vert {\boldsymbol{q}} + {\boldsymbol{p}}\Vert } \end{array}} \Big ( Z_{0,+,\sigma }(x_{2}) g_{+;\textrm{s}}({\boldsymbol{q}}) \overline{Z_{-n,+,\rho }(z_{2})} \\&\qquad \cdot Z_{m_{2},+,\varrho }(w_{2}) g_{+;\textrm{s}}\big ({\boldsymbol{q}} + {\boldsymbol{p}} + (m_{2} + n) {\boldsymbol{\alpha }}\big ) \overline{Z_{n + m_{2},+,\zeta }(y_{2})}\Big ). \end{aligned} \end{aligned}$$

Here we use that the second propagator is partially regularized by $(n+m_{2}){\boldsymbol{\alpha }}$. In fact, we have:

5.72 $$\begin{aligned} \big | g_{+;\textrm{s}}\big ({\boldsymbol{q}} + {\boldsymbol{p}} + (m_{2} + n){\boldsymbol{\alpha }}\big ) \big | \le \frac{C}{\sqrt{\Vert {\boldsymbol{q}} + {\boldsymbol{p}} \Vert }}. \end{aligned}$$

Therefore, again by the estimate (3.7):

5.73 $$\begin{aligned} \big | \text {II}^{\text {A}}_{2} \big | \le e^{-c (x_{2} + y_{2} + w_{2} + z_{2})}\frac{1}{\Vert {\boldsymbol{q}} \Vert } \frac{1}{\sqrt{\Vert {\boldsymbol{q}} + {\boldsymbol{p}}\Vert }}. \end{aligned}$$

This concludes the analysis of the $m_{1} = 0$ contribution to $\text {II}^{\text {A}}$. Let us now discuss the contribution of the modes $m_{1} \ne 0$ to $\text {II}^{\text {A}}$; we denote by $\text {II}^{\text {A}}_{3}$ this contribution. Here we separate both $n+m_{2}$ and $m_{1}$ in $\Vert (n + m_{2}) {\boldsymbol{\alpha }} \Vert \le \sqrt{\Vert {\boldsymbol{q}} + {\boldsymbol{p}}\Vert }$, $\Vert (n + m_{2}) {\boldsymbol{\alpha }} \Vert > \sqrt{\Vert {\boldsymbol{q}} + {\boldsymbol{p}}\Vert }$ and $\Vert m_{1}{\boldsymbol{\alpha }} \Vert \le \sqrt{\Vert {\boldsymbol{q}} \Vert }$, $\Vert m_{1}{\boldsymbol{\alpha }} \Vert > \sqrt{\Vert {\boldsymbol{q}} \Vert }$. As we did before for $n + m_{2}$, for the modes with small norm we use twice the Diophantine condition, while for the modes with larger norm, we use that the propagator is effectively regularized; we omit the details. The resulting estimate is:

5.74 $$\begin{aligned} |\text {II}^{\text {A}}_{3}| \le e^{-c (x_{2} + y_{2} + w_{2} + z_{2})} \frac{1}{\sqrt{\Vert {\boldsymbol{q}} \Vert }} \frac{1}{\sqrt{\Vert {\boldsymbol{q}} + {\boldsymbol{p}}\Vert }}. \end{aligned}$$

All in all, the term $\text {II}^{\text {A}}$ in (5.62) is estimated as:

5.75 $$\begin{aligned} |\text {II}^{\text {A}}| \le e^{-c (x_{2} + y_{2} + w_{2} + z_{2})} \frac{1}{\Vert {\boldsymbol{q}} \Vert } \frac{1}{\sqrt{\Vert {\boldsymbol{q}} + {\boldsymbol{p}}\Vert }}. \end{aligned}$$

In order to conclude the discussion of $\text {II}$, we are left with discussing the contributions associated with $m_{1}\ne 0$ and $n + m_{2} = 0$. These are estimated exactly as we did for $n + m_{2} \ne 0$, and we omit the details. All in all, we obtained:

5.76 $$\begin{aligned} |\text {II}| \le e^{-c (x_{2} + y_{2} + w_{2} + z_{2})} \Big ( \frac{1}{\sqrt{\Vert {\boldsymbol{q}} \Vert }} \frac{1}{\Vert {\boldsymbol{q}} + {\boldsymbol{p}}\Vert } + \frac{1}{\Vert {\boldsymbol{q}} \Vert } \frac{1}{\sqrt{\Vert {\boldsymbol{q}} + {\boldsymbol{p}}\Vert }}\Big ). \end{aligned}$$

In particular, the term $\text {II}$ satisfies the bound (5.43).

**Conclusion of the proof of Proposition**5.3. We are now ready to determine the low momentum behavior of the vertex function, starting from (5.57) and from the above estimates. We have, recall Eq. (5.56):

5.77 $$\begin{aligned} \begin{aligned}&{\hat{S}}^{\textrm{s}}_{3; \mu ,\sigma ,\zeta }(\boldsymbol{p}, \boldsymbol{k}, \boldsymbol{k} + {\boldsymbol{p}}; x_{2}, y_{2}) \\&= \sum _{n} \sum _{\rho ,\varrho } \sum _{z_{2},w_{2}}\Big ({\hat{J}}_{\mu ;\rho ,\varrho }(k_{1} + n\alpha + p_{1},p_{1};z_{2},w_{2}) Z_{0,+,\sigma }(x_{2}) g_{+;\textrm{s}}\big ({\boldsymbol{q}}(\boldsymbol{k})\big ) \overline{Z_{-n,+,\rho }(z_{2})} \\&\qquad \cdot Z_{-n,+,\varrho }(w_{2}) g_{+;\textrm{s}}\big ({\boldsymbol{q}}(\boldsymbol{k}) + {\boldsymbol{p}}\big ) \overline{Z_{0,+,\zeta }(y_{2})}\Big ) + {\widetilde{R}}_{3; \mu ,\sigma ,\zeta }(\boldsymbol{p}, \boldsymbol{k}, \boldsymbol{k} + {\boldsymbol{p}}; x_{2}, y_{2}), \end{aligned} \nonumber \\ \end{aligned}$$

where the error term ${\widetilde{R}}_{3}$ takes into account the contribution obtained plugging $\text {II}$ (bounded in (5.76)) and $\text {III}$ (bounded in (5.61)) into (5.56), while the main term is obtained plugging $\text {I}$ in (5.56). Consider the main term. Choosing ${\boldsymbol{k}}$ and ${\boldsymbol{k}} + {\boldsymbol{p}}$ close enough to ${\boldsymbol{k}}_{F}^{+}(\lambda )$, the main contribution is:

5.78 $$\begin{aligned} \begin{aligned}&{\hat{S}}^{\textrm{s}; \text {main}}_{3; \mu ,\sigma ,\zeta }(\boldsymbol{p}, \boldsymbol{k}, \boldsymbol{k} + {\boldsymbol{p}}; x_{2}, y_{2}) \\&= Z_{0,+,\sigma }(x_{2}) \overline{Z_{0,+,\zeta }(y_{2})} g_{+;\textrm{s}}({\boldsymbol{q}}) g_{+;\textrm{s}}({\boldsymbol{q}} + {\boldsymbol{p}}) \sum _{n} \langle Z_{-n,+}, J_{\mu }(k_{F}^{+}(\lambda ) + n\alpha ,0) Z_{-n,+} \rangle , \end{aligned} \nonumber \\ \end{aligned}$$

where we used the notation $\langle f,g \rangle := \sum _{x_{2}, \rho } \overline{f_{\rho }(x_{2})} g_{\rho }(x_{2})$. All in all, putting together (5.56), (5.57), (5.77), (5.78):

5.79 $$\begin{aligned} \begin{aligned} {\hat{S}}_{3; \mu ,\sigma ,\zeta }(\boldsymbol{p}, \boldsymbol{k}, \boldsymbol{k} + {\boldsymbol{p}}; x_{2}, y_{2})&= Z_{0,+,\sigma }(x_{2}) \overline{Z_{0,+,\zeta }(y_{2})} g_{+;\textrm{s}}({\boldsymbol{q}}) g_{+;\textrm{s}}({\boldsymbol{q}} + {\boldsymbol{p}}) \zeta _{\mu ,+} \\&\quad + R^{\text {tot}}_{3; \mu ,\sigma ,\zeta }(\boldsymbol{p}, \boldsymbol{k}, \boldsymbol{k} + {\boldsymbol{p}}; x_{2}, y_{2}), \end{aligned} \end{aligned}$$

where

5.80 $$\begin{aligned} \zeta _{\mu ,+} = \sum _{n} \langle Z_{-n,+}, J_{\mu }(k_{F}^{+}(\lambda ) + n\alpha ,0) Z_{-n,+} \rangle , \end{aligned}$$

and where, for $\Vert {\boldsymbol{k}} - {\boldsymbol{k}}_{F}^{\omega }(\lambda ) \Vert \ll 1$, $\Vert {\boldsymbol{p}} \Vert \ll 1$ and $L_{2}$ large enough:

5.81 $$\begin{aligned}  &   \sum _{x_{2}, y_{2}} \big | Z_{0,+,\sigma }(x_{2})\big | \big | Z_{0,+,\zeta }(y_{2})\big | \Big |g^{-1}_{+;\textrm{s}}({\boldsymbol{q}})\Big | \Big |g^{-1}_{+;\textrm{s}}({\boldsymbol{q}} + {\boldsymbol{p}})\Big | \Big |R^{\text {tot}}_{3; \mu ,\sigma ,\zeta }(\boldsymbol{p}, \boldsymbol{k}, \boldsymbol{k} + {\boldsymbol{p}}; x_{2}, y_{2})\Big |\nonumber \\  &   \quad = \textrm{o}(1). \end{aligned}$$

This concludes the proof of Proposition 5.3□

##### Proof of Proposition 5.4

As in the proof of Proposition 5.3, we start by writing:

5.82 $$\begin{aligned} {\hat{S}}_{2;\sigma ,\zeta }(\boldsymbol{k},{\boldsymbol{k}}; x_{2}, y_{2}) = \sum _{m,\omega } Z_{m,\omega ,\sigma }(x_{2}) g_{\omega ;\textrm{s}}({\boldsymbol{q}} + m{\boldsymbol{\alpha }}) \overline{Z_{-n+m,\omega ,\zeta }(y_{2})} + {\hat{R}}_{\sigma ,\zeta }({\boldsymbol{k}}, {\boldsymbol{k}}; x_{2}, y_{2}), \nonumber \\ \end{aligned}$$

where ${\hat{R}}_{\sigma ,\zeta }({\boldsymbol{k}}, {\boldsymbol{k}}; x_{2}, y_{2})$ is bounded as, recall (5.55):

5.83 $$\begin{aligned} \Big | \text {d}_{k_{0}}^{n_{0}} \text {d}_{k_{1}}^{n_{1}} {\hat{R}}_{\sigma ,\zeta }({\boldsymbol{k}}, {\boldsymbol{k}}; x_{2}, y_{2})\Big | \le \frac{C_{n_{0},n_{1}} e^{-{\tilde{c}}|x_{2} - y_{2}|}}{\Vert {\boldsymbol{k}} - {\boldsymbol{k}}_{F}^{+} \Vert ^{n_{0} + n_{1} + 1-\xi }} + \sum _{\begin{array}{c} \omega _{1},\omega _{2} \\ (\omega _{1},\omega _{2}) \ne (+,+) \end{array}} \!\!\! C_{n_{0},n_{1}}\frac{ e^{-c(|x_{2}|_{\omega _{1}} + |y_{2}|_{\omega _{2}})}}{|k_{0}|^{n_{0} + n_{1} + 1-\xi }}. \nonumber \\ \end{aligned}$$

Consider now the main term in (5.82). The contribution associated with ω=- satisfies the bound (5.45), using the exponential decay of the edge modes. Next, consider the main contribution to (5.82) with ω=+. Similarly to what we did in the analysis of the vertex function, we separate the m=0 term in the sum from the others. Proceeding as after (5.62), the terms with m≠0 give a contribution that is bounded by $e^{-c(x_{2} + y_{2})} / \Vert {\boldsymbol{q}} \Vert ^{(1/2)(n_{0} + n_{1} + 1)}$, which in particular satisfies (5.45). This concludes the proof of (5.44), (5.45).

### Proof of Theorem 3.3

In this section we shall prove Theorem 3.3, which allows us to compute the edge transport coefficients. The analysis will rely on the asymptotics of the two-point function, Eq. (3.3), on the relations (3.8), and on the discussion of Sect. 5.2.1 about the structure of the current-current correlations functions, recall Eqs. (5.20), (5.26).

#### Susceptibility

Let us start from the susceptibility, $\chi ^{\beta , L}_{0;\ell }(\eta , \theta )$. To avoid carrying out the error term $\varepsilon ^{\beta ,L}_{0;\ell }(\eta , \theta )$ in (5.1), we will suppose for the moment that $\eta = \eta _{\beta } \in (2\pi / \beta ) \mathbb {N}$; considering a general η>0 will simply introduce an additive error term bounded as in (5.2), which we will take into account at the very end. From (5.1), using Wick’s rule:

5.84 $$\begin{aligned}  &   \chi ^{\beta , L}_{0;\ell }(\eta , \theta )\nonumber \\  &   =-\theta \sum _{{\vec x},{\vec y}} \mu (\theta \vec x) \phi _{\ell }(\theta y_{1}, y_{2})\int _{-\frac{\beta }{2}}^{\frac{\beta }{2}} d s\,e^{-i\eta s} {{\,\textrm{Tr}\,}}\big (S^{\beta ,L}_{2}(s,{\vec x}; 0,{\vec y}) S^{\beta ,L}_{2}(0,{\vec y}; s,{\vec x})\big ),\nonumber \\ \end{aligned}$$

where the trace is over the internal degrees of freedom. We shall use that $\chi ^{\beta , L}_{0;\ell }(\eta , \theta ) = K_{00}^{\beta ,L}(\eta , \theta )$, recall Eq. (5.12), and we shall now define the decomposition (5.14). To this end, the following technical lemma will be useful.

##### Lemma 5.5

Let $$F(\vec {\boldsymbol{x}}; \vec {\boldsymbol{y}})$$ be a β-periodic function in the imaginary times and satisfying the cylindric boundary conditions in $\vec{x}$, $\vec{y}$, such that, for ξ>0:

5.85 $$\begin{aligned} F(\vec {\boldsymbol{x}}; \vec {\boldsymbol{y}})  &   = \sum _{m \in \mathbb {Z}} F_{m}({\boldsymbol{x}} - {\boldsymbol{y}}; x_{2}, y_{2}) e^{-i m\alpha y_{1}},\quad \nonumber \\ \big |F_{m}({\boldsymbol{x}} - {\boldsymbol{y}}; x_{2}, y_{2})\big |  &   \le \frac{C e^{-c|m|} e^{-c|x_{2} - y_{2}|}}{1 + \Vert {\boldsymbol{x}} - {\boldsymbol{y}} \Vert ^{2+\xi }} \end{aligned}$$

Consider the function:

5.86 $$\begin{aligned} g(\eta , \theta ) = \theta \sum _{{\vec x},{\vec y}} \mu (\theta \vec x)\phi _{\ell }(\theta y_{1}, y_{2}) \int _{-\frac{\beta }{2}}^{\frac{\beta }{2}} d s\,e^{-i\eta s} F((s,\vec x); (0, \vec y)). \end{aligned}$$

Then, for α>0:

5.87 $$\begin{aligned} \big |g(\eta , \theta ) - g(\eta ,\theta ')\big |\le C (|\theta |^{\alpha } + |\theta '|^{\alpha }). \end{aligned}$$

We postpone the proof of this lemma to Appendix E. Let us define $K^{\text {sing}}_{00}(\eta , \theta )$ in (5.14) as

5.88 $$\begin{aligned} K^{\text {sing}}_{00}(\eta , \theta ):= - \theta \sum _{{\vec x},{\vec y}} \mu (\theta \vec x)\phi _{\ell }(\theta y_{1}, y_{2})\int _{-\frac{\beta }{2}}^{\frac{\beta }{2}} d s\,e^{-i\eta s} {{\,\textrm{Tr}\,}}\big (S^{\textrm{s}}_{2}(s,{\vec x}; 0,{\vec y}) S^{\textrm{s}}_{2}(0,{\vec y}; s,{\vec x})\big ), \nonumber \\ \end{aligned}$$

with $S^{\textrm{s}}_{2}$ given by (4.188). Using the result about the structure of the subleading contributions to the two point function, Eq. (3.9), together with Lemma 5.5, we see that $K^{\text {reg}}_{00}(\eta , \theta )$ satisfies the estimate (5.15). Furthermore, from the information about the two-point function provided by Theorem 3.1, and from the compact support of the test functions, we have:

5.89 $$\begin{aligned} |K_{\mu \nu }^{\beta ,L}(\eta ,\theta )|\le C_{\ell } |\log \theta |, \end{aligned}$$

uniformly in $\beta , \eta , L_{1}, L_{2}$. Thus, we can apply the strategy outlined in Sect. 5.2.1, to compute the susceptibility only relying on $K^{\text {sing}}_{00}$.

We rewrite (5.88) using the explicit expression of $S^{\textrm{s}}_{2}$, Eq. (4.188). We have:

5.90 $$\begin{aligned} \begin{aligned} K^{\text {sing}}_{00}(\eta , \theta )&= - \theta \sum _{{\vec x},{\vec y} } \mu (\theta \vec x) \phi _{\ell }(\theta y_{1}, y_{2}) | Z_{+}(\vec x) |^{2} | Z_{+}(\vec y) |^{2} \\&\qquad \cdot \int _{-\beta /2}^{\beta /2} d s\, e^{-i\eta s} \check{g}_{+;\textrm{s}}\big ((x_{1}, s); (y_{1},0)\big ) \check{g}_{+;\textrm{s}}\big ((y_{1},0); (x_{1}, s)\big )+ \textrm{O}_{\eta ,\theta }(e^{-c L_{2}}), \end{aligned} \nonumber \\ \end{aligned}$$

where $| Z_{\omega }(\vec x) |^{2} = \sum _{\sigma } |Z_{\omega ,\sigma }(\vec x)|^{2}$. The exponentially small error term takes into account the contribution due to the case in which at least one chirality is ω=-. This corresponds to an edge state localized at $x_{2} = L_{2}$; the contribution is exponentially small because the test functions are localized at $x_{2} = 0$. The bound for this error term might be non-uniform in $\eta , \theta$ (via a logarithmic divergence); this is not an issue, since the limit $L_{2} \rightarrow \infty$ is taken before $(\eta , \theta ) \rightarrow (0,0)$.

##### Remark 5.6

In what follows, we will need bounds for the Fourier transform of the test function $\mu (\cdot )$, which satisfies the assumptions spelled out after (2.31). We have, for $\vec x \in \Lambda _{L}$:

5.91 $$\begin{aligned} \mu (\theta \vec x) = \frac{1}{L_{1}} \sum _{k \in \mathscr {D}_{L}}\mathrm e^{i kx_{1}} {\hat{\mu }}_{\theta }(k, x_{2}),\qquad \hat{\mu }_{\theta }(k,x_{2}) = \frac{1}{\theta } \sum _{n\in \mathbb {Z}} \hat{\mu }_{\infty }\big ((k + 2\pi n) / \theta , \theta x_{2}\big ). \nonumber \\ \end{aligned}$$

Let $|\cdot |_{\mathbb {T}}=\min _{n\in \mathbb {Z}} | \cdot + 2\pi n |$ be the distance on the torus of length 2π. By (2.33), the following bound holds:

5.92 $$\begin{aligned} |{\hat{\mu }}_{\theta }(k,x_{2})| \le \frac{C_{r}}{\theta } \frac{1}{1 + (| k |_{\mathbb {T}} / \theta )^{r}} \frac{1}{1 + \theta ^{r} |x_{2}|^{r}}. \end{aligned}$$

**Analysis of the singular part.** Let us consider the integral in (5.90). We have:

5.93 $$\begin{aligned} \begin{aligned}&\int _{-\beta /2}^{\beta /2} d s\, e^{-i\eta s} \check{g}_{+;\textrm{s}}\big ((x_{1}, s); (y_{1},0)\big ) \check{g}_{+;\textrm{s}}\big ((y_{1},0); (x_{1}, s)\big ) \\&\qquad = \frac{1}{L_{1}} \sum _{p \in \mathscr {D}_{L}} e^{ip(x_{1} - y_{1})} \frac{1}{\beta L_{1}} \sum _{\boldsymbol{q} \in \mathscr {D}_{\beta ,L}} g_{+;\textrm{s}}(q_{0} + \eta , q_1 + p) g_{+;\textrm{s}}(q_{0}, q_{1}) \\&\qquad \equiv \frac{1}{L_{1}} \sum _{p \in \mathscr {D}_{L}} e^{ip(x_{1} - y_{1})} \mathcal {B}_{+}^{\beta , L_{1}}(\eta , p), \end{aligned} \end{aligned}$$

where $\mathcal {B}_{\omega }^{\beta , L_{1}}(\eta ,p)$ is the regularized relativistic bubble diagram at external momentum (η,p), with chiralities ω; we refer to Appendix C for its computation. Plugging (5.93) into (5.90), we get:

5.94 $$\begin{aligned} \begin{aligned} \chi ^{\text {sing}}_{0;\ell }(\eta , \theta ) =&-\frac{\theta }{L_{1}}\sum _{p} \sum _{x_{2},y_{2}}\mathcal {F}\big (\mu (\theta \cdot , \theta x_{2}) |Z_{+} (\cdot ,x_{2})|^{2}\big )(-p) \\&\quad \cdot \mathcal {F}\big (\phi _{\ell }(\theta \cdot ,y_{2}) | Z_{+}(\cdot ,y_{2})|^{2} \big )(p) \,\mathcal {B}_{+}^{\beta , L_{1}}(\eta , p) + \textrm{O}_{\eta ,\theta }(e^{-c L_{2}}), \end{aligned} \end{aligned}$$

where $\mathcal {F}:f(\cdot )\mapsto {\hat{f}}(\cdot )= \sum _x e^{-i(\cdot ) x}f(x)$ denotes the Fourier transform and:

5.95 $$\begin{aligned} \begin{aligned} \mathcal {F}\big (\mu (\theta \cdot , \theta x_{2}) | Z_{\omega }(\cdot ,x_{2})|^{2}\big )(-p)&= \frac{1}{L_{1}} \sum _{q} {\hat{\mu }}_{\theta }(-p-q,x_2) \mathcal {F}\big (|Z_{\omega }(\cdot , x_{2}) |^2\big ) (q) \\ \mathcal {F}\big ( \phi _{\ell }(\theta \cdot , y_{2}) | Z_{\omega }(\cdot ,y_{2}) |^{2} \big )(p)&= \frac{1}{L_{1}}\sum _{q} {\hat{\phi }}_{\theta ,\ell }(p - q,y_2) \mathcal {F}\big (|Z_{\omega }(\cdot , y_{2})|^2\big )(q). \end{aligned} \end{aligned}$$

Now, using (4.187):

5.96 $$\begin{aligned} |Z_{\omega }(\vec x)|^2= &   \sum _{n \in \mathbb {Z}} \sum _{\sigma } \Big (\sum _{m\in {\mathbb {Z}}}Z_{m,\omega ,\sigma }(x_2) \overline{Z_{m-n,\omega ,\sigma }(x_2)} \Big ) e^{-in\alpha x_{1}}\nonumber \\\equiv &   \sum _{n \in \mathbb {Z}} (Z_{\omega }\star \overline{Z_{\omega }})(n;x_{2}) e^{-in\alpha x_{1}}, \end{aligned}$$

we can rewrite (5.95) as:

5.97 $$\begin{aligned} \begin{aligned} \mathcal {F}\big (\mu (\theta \cdot , \theta x_{2}) | Z_{\omega }(\cdot ,x_{2})|^{2}\big )(-p)&= \sum _{n\in \mathbb {Z}} {\hat{\mu }}_{\theta }(-p-n\alpha ,x_2) (Z_{\omega }\star \overline{Z_{\omega }})(n;x_{2}) \\ \mathcal {F}\big ( \phi _{\ell }(\theta \cdot , y_{2}) | Z_{\omega }(\cdot ,y_{2}) |^{2} \big )(p)&= \sum _{n\in \mathbb {Z}} {\hat{\phi }}_{\theta ,\ell }( p-n\alpha ,y_{2}) (Z_{\omega }\star \overline{Z_{\omega }})(n;y_{2}). \end{aligned} \end{aligned}$$

Observe that, from (3.7), the following estimate holds:

5.98 $$\begin{aligned} |(Z_{\omega }\star \overline{Z_{\omega }})(n;x_{2})| \le Ce^{-\frac{c}{2}|n|} e^{-c|x_{2}|_{\omega }}. \end{aligned}$$

Plugging these expressions into (5.94), we get:

5.99 $$\begin{aligned} \begin{aligned} \chi ^{\text {sing}}_{0;\ell }(\eta , \theta )&= - \frac{\theta }{L_{1}} \sum _{p} \sum _{n,m} \sum _{x_{2},y_{2}} (Z_{+}\star \overline{Z_{+}})(m;y_{2}) (Z_{+}\star \overline{Z_{+}})(n;x_{2})\\&\qquad \cdot {\hat{\mu }}_{\theta }(-p-n\alpha ,x_2) {\hat{\phi }}_{\theta ,\ell }( p-m\alpha ,y_{2}) \mathcal {B}_{+}^{\beta , L_{1}}(\eta , p) +\textrm{O}_{\eta ,\theta }(e^{-c L_{2}}) \\&= -\frac{\theta }{L_{1}} \sum _{p} \sum _{n,m} \sum _{x_{2},y_{2}} (Z_{+}\star \overline{Z_{+}})(m;y_{2}) (Z_{+}\star \overline{Z_{+}})(n;x_{2}) \\&\qquad \cdot {\hat{\mu }}_{\theta }(-p,x_2) {\hat{\phi }}_{\theta ,\ell }\big (p-(m+n)\alpha ,y_{2}\big ) \mathcal {B}_{+}^{\beta , L_{1}}(\eta , p-n\alpha ) + \textrm{O}_{\eta ,\theta }(e^{-c L_{2}}). \end{aligned} \end{aligned}$$

**Cutting off large frequencies.** Let:

5.100 $$\begin{aligned} \begin{aligned} \chi ^{\text {sing}}_{0; \ell , N}(\eta , \theta )&:= -\frac{\theta }{L_{1}} \sum _{p} \sum _{\begin{array}{c} n,m \\ |n| \le N,\, |m| \le N \end{array}} \sum _{x_{2},y_{2}} (Z_{+}\star \overline{Z_{+}})(m;y_{2}) (Z_{+}\star \overline{Z_{+}})(n;x_{2}) \\&\qquad \cdot {\hat{\mu }}_{\theta }(-p,x_2) {\hat{\phi }}_{\theta ,\ell }\big (p-(m+n)\alpha ,y_{2}\big ) \mathcal {B}_{+}^{\beta , L_{1}}(\eta , p-n\alpha ). \end{aligned} \end{aligned}$$

This function approximates $\chi ^{\text {sing}}_{0;\ell }(\eta , \theta )$, up to small errors, uniformly in all parameters. In fact, from the estimate (5.98) for $Z_{n,\omega ,\sigma }(x_{2})$, from the boundedness of ${\hat{\phi }}$, and from the boundedness of $\mathcal {B}_{\omega }^{\beta , L_{1}}$, we have:

5.101 $$\begin{aligned} \big | \chi ^{\text {sing}}_{0;\ell }(\eta , \theta ) - \chi ^{\text {sing}}_{0; \ell , N}(\eta , \theta ) \big |\le &   \frac{C}{L_{1}} \sum _{p} | {\hat{\mu }}_{\theta }(-p, x_2) | e^{-cN} + C_{\eta ,\theta }e^{-cL_{2}} \nonumber \\\le &   Ke^{-cN} + C_{\eta ,\theta }e^{-cL_{2}}, \end{aligned}$$

where the last term takes into account the exponentially small error in (5.90).

**Estimating the contribution of** m+n≠0. Let us now focus on $\chi ^{\text {sing}}_{0; \ell , N}(\eta , \theta )$. We claim that the contribution with n+m≠0 gives a subleading contribution, for θ small. In fact:

5.102 $$\begin{aligned} \begin{aligned}&\Big |\frac{\theta }{L_{1}} \sum _{p} \sum _{\begin{array}{c} n\ne -m \\ |n| \le N,\, |m| \le N \end{array}} \sum _{x_{2},y_{2}}(Z_{+}\star \overline{Z_{+}})(m;y_{2}) (Z_{+}\star \overline{Z_{+}})(n;x_{2}) \\&\qquad \cdot {\hat{\mu }}_{\theta }(-p,x_2) {\hat{\phi }}_{\theta ,\ell }\big (p-(m+n)\alpha ,y_{2}\big ) \mathcal {B}_{+}^{\beta , L_{1}}(\eta , p-n\alpha )\Big | \\&\le \frac{C}{\theta L_{1}} \sum _{p} \sum _{n\ne -m} \sum _{x_{2},y_{2}} e^{-\frac{c}{2}|m|} e^{-cy_{2}} e^{-\frac{c}{2}|n|} e^{-cx_{2}} \\&\qquad \cdot \frac{C_{r}}{1 + (|p|_{\mathbb {T}}/\theta )^{r}} \frac{C_{r}}{1 + \big (\big |\big (p - (m+n)\alpha \big )\big |_{\mathbb {T}}/\theta \big )^{r}},\qquad \text {for all } r\in \mathbb {N} \end{aligned} \end{aligned}$$

where the last two factors bound the functions ${\hat{\mu }}_{\theta }$ and ${\hat{\phi }}_{\theta ,\ell }$, recall (5.92). Thus, using that:

5.103 $$\begin{aligned} \begin{aligned}&\frac{1}{1 + (|p|_{\mathbb {T}}/\theta )^{r}} \frac{1}{1 + \big (\big |\big (p - (m+n)\alpha \big )\big |_{\mathbb {T}}/\theta \big )^{r}} \\&\le \frac{C_{r}}{1 + (|p|_{\mathbb {T}}/\theta )^{r/2}} \frac{1}{1 + (|p|_{\mathbb {T}}/\theta )^{r/2}}\frac{1}{1 + \big (\big |\big (p - (m+n)\alpha \big )\big |_{\mathbb {T}}/\theta \big )^{r/2}} \\&\le \frac{K_{r}}{1 + (|p|_{\mathbb {T}}/\theta )^{r/2}} \frac{\big (\big |p \big |_{\mathbb {T}}/\theta \big )^{r/2} + \big (\big |- p + (m+n)\alpha \big |_{\mathbb {T}}/\theta \big )^{r/2}}{1 + (|p|_{\mathbb {T}}/\theta )^{r/2}}\\  &\quad \cdot \frac{1}{1 + \big (\big |\big (p - (m+n)\alpha \big )\big |_{\mathbb {T}}/\theta \big )^{r/2}} \frac{1}{\big (\big |(m+n)\alpha \big |_{\mathbb {T}}/\theta \big )^{r/2}} \\&\le \frac{2K_{r}}{1 + (|p|_{\mathbb {T}}/\theta )^{r/2}} \frac{1}{\big (\big |(m+n)\alpha \big |_{\mathbb {T}}/\theta \big )^{r/2}}, \end{aligned} \end{aligned}$$

and that $|(n+m)\alpha |_{\mathbb {T}} \ge c / |n+m|^{\tau } \ge C N^{-\tau }$ for 0<|n+m|≤2N, we get

5.104 $$\begin{aligned} (5.102) \le C_{N} \theta ^{r/2}, \end{aligned}$$

uniformly in all the other parameters.

**Estimating the contribution of** m+n=0, n≠0. We are now left with the contribution to $\chi ^{\text {sing}}_{0; \ell , N}(\eta , \theta )$ coming from the modes *m*, *n* such that m+n=0. It is:

5.105 $$\begin{aligned} \begin{aligned} \chi ^{\text {main}}_{0; \ell , N}(\eta , \theta )&= -\frac{\theta }{L_{1}} \sum _{p} \sum _{n: |n| \le N} \sum _{x_{2},y_{2}} (Z_{+}\star \overline{Z_{+}})(-n;y_{2}) (Z_{+}\star \overline{Z_{+}})(n;x_{2}) \\&\qquad \cdot {\hat{\mu }}_{\theta }(-p,x_2) {\hat{\phi }}_{\theta ,\ell }(p,y_{2}) \mathcal {B}_{+}^{\beta , L_{1}}(\eta , p-n\alpha ) \end{aligned} \end{aligned}$$

which we further rewrite as:

5.106 $$\begin{aligned} \begin{aligned} \chi ^{\text {main}}_{0; \ell , N}(\eta , \theta )&= -\frac{\theta }{L_{1}} \sum _{p} \sum _{x_{2},y_{2}}(Z_{+}\star \overline{Z_{+}})(0;y_{2}) (Z_{+}\star \overline{Z_{+}})(0;x_{2}) \\&\qquad \cdot {\hat{\mu }}_{\theta }(-p,x_2) {\hat{\phi }}_{\theta ,\ell }(p,y_{2}) \mathcal {B}_{+}^{\beta , L_{1}}(\eta , p) + R_{0;\ell , N}(\eta ,\theta ) \end{aligned} \end{aligned}$$

where $R_{0;\ell , N}(\eta ,\theta )$ takes into account the contribution of the modes n≠0, |n|≤N. We rewrite this last term as, after the change of variables $p/\theta \rightarrow p$:

5.107 $$\begin{aligned} \begin{aligned} R_{0;\ell ,N}(\eta ,\theta )&= -\frac{1}{\theta L_{1}} \sum _{p \in \frac{2\pi }{\theta L_{1}} (\mathbb {Z} / L_{1}\mathbb {Z})} \sum _{n: 0<|n| \le N} \sum _{x_{2},y_{2}}(Z_{+}\star \overline{Z_{+}})(-n;y_{2}) (Z_{+}\star \overline{Z_{+}})(n;x_{2}) \\&\quad \cdot \theta {\hat{\mu }}_{\theta }(-\theta p,x_2) \,\theta {\hat{\phi }}_{\theta ,\ell }(\theta p,y_{2}) \mathcal {B}_{+}^{\beta , L_{1}}(\eta , \theta p-n\alpha ). \end{aligned} \end{aligned}$$

In order to bound this term, let us recall the estimates, valid for $p\in (2\pi / L_{1}\theta ) (\mathbb {Z} / L_{1}\mathbb {Z})$:

5.108 $$\begin{aligned} \theta \big |{\hat{\mu }}_{\theta }(\theta p,x_2)\big |\le &   \frac{C_{r}}{1+(|p|_{\mathbb {T}_{\theta ^{-1}}})^{r}}\frac{1}{1+|\theta x_{2}|^{r}},\quad \nonumber \\ \theta \big |{\hat{\phi }}_{\theta ,\ell }(-\theta p,y_{2})\big |\le &   \frac{C_{r}}{1+(|p|_{\mathbb {T}_{\theta ^{-1}}})^{r}} \frac{1}{1+|y_{2} / \ell |^{r}}, \end{aligned}$$

with $\mathbb {T}_{\theta ^{-1}}$ the torus of side $2\pi /\theta$. Observe that $|n\alpha |_{\mathbb {T}} \ge c / N^{\tau } = c_{N}$ for 0<|n|≤N; thus, we can further separate the sum over *p* as:

5.109 $$\begin{aligned} \sum _{p \in \frac{2\pi }{\theta L_{1}} (\mathbb {Z} / L_{1}\mathbb {Z})} (\cdots ) = \sum _{\begin{array}{c} p \in \frac{2\pi }{\theta L_{1}} (\mathbb {Z} / L_{1}\mathbb {Z}) \\ 2\theta |p|_{\mathbb {T}_{\theta ^{-1}}} \le c_{N} \end{array}} (\cdots ) + \sum _{\begin{array}{c} p \in \frac{2\pi }{\theta L_{1}} (\mathbb {Z} / L_{1}\mathbb {Z}) \\ 2\theta |p|_{\mathbb {T}_{\theta ^{-1}}} > c_{N} \end{array}} (\cdots ); \end{aligned}$$

correspondingly, we write:

5.110 $$\begin{aligned} R_{0;\ell ,N}(\eta ,\theta ) = R^{\le }_{0;\ell , N}(\eta ,\theta ) + R^{>}_{0;\ell , N}(\eta ,\theta ). \end{aligned}$$

Thanks to the estimates (5.108) for the decay of the test functions in Fourier space, $R^{>}_{0;\ell ,N}(\eta ,\theta )$ satisfies:

5.111 $$\begin{aligned} | R^{>}_{0;\ell ,N}(\eta ,\theta ) | \le C_{N,r} \theta ^{r},\qquad \text {for all } r\in \mathbb {N}. \end{aligned}$$

Next, consider $R^{\le }_{0;\ell ,N}$. Here, since $\theta | p |_{\mathbb {T}_{\theta ^{-1}}} = | \theta p |_{\mathbb {T}} < c_{N}/2$ and $|n \alpha |_{\mathbb {T}}>c_{N}$, we will use that the function $(\eta , \theta )\mapsto \mathcal {B}_{+}^{\beta , L_{1}}(\eta , \theta p-n\alpha )$ is continuous in θp at θp=0, which can be checked from the explicit expression obtained in Appendix C, and reported below, see (5.120). We claim that:

5.112 $$\begin{aligned} \Big | R^{\le }_{0;\ell ,N}(\eta ,\theta ) - R^{\le }_{0;\ell ,N}(\eta ,\theta ') \Big | \le C_{N} (|\theta |^{\alpha } + |\theta '|^{\alpha }) \end{aligned}$$

for some α>0. To see this, we approximate the sum over the momenta as an integral:

5.113 $$\begin{aligned}  &   R^{\le }_{0;\ell ,N}(\eta ,\theta )\nonumber \\  &   = -\int _{\mathbb {T}_{\theta ^{-1}}:\, 2\theta |p|_{\mathbb {T}_{\theta ^{-1}}} \le c_{N}} \frac{dp}{(2\pi )}\sum _{n: 0<|n| \le N} \sum _{x_{2},y_{2}}(Z_{+}\star \overline{Z_{+}})(-n;y_{2}) (Z_{+}\star \overline{Z_{+}})(n;x_{2}) \nonumber \\  &   \quad \cdot \theta {\hat{\mu }}_{\theta }(-\theta p,x_2) \,\theta {\hat{\phi }}_{\theta ,\ell }(\theta p,y_{2}) \mathcal {B}_{+}^{\infty }(\eta , \theta p-n\alpha ) + \textrm{o}(1), \end{aligned}$$

where the error terms vanish as $L_{1} \rightarrow \infty$ (to control the errors coming from the integral approximation). Then, using the exponential decay of $Z_{m,\omega ,\sigma }(x_2)$, we can localize the test functions at $x_{2} = y_{2} = 0$, up to errors that vanish as a positive power of θ as $\theta \rightarrow 0$ and $\ell \rightarrow \infty$, which are included in the new $\textrm{o}(1)$ terms:

5.114 $$\begin{aligned} \begin{aligned}&R^{\le }_{0;\ell ,N}(\eta ,\theta )\\&\quad = -\int _{\mathbb {T}_{\theta ^{-1}}:\, 2\theta |p|_{\mathbb {T}_{\theta ^{-1}}} \le c_{N}} \frac{dp}{(2\pi )} \sum _{n: 0<|n| \le N} \sum _{x_{2},y_{2}}(Z_{+}\star \overline{Z_{+}})(-n;y_{2}) \\&\qquad \cdot (Z_{+}\star \overline{Z_{+}})(n;x_{2})\theta {\hat{\mu }}_{\theta }(-\theta p, 0) \,\theta {\hat{\phi }}_{\theta ,\ell }(\theta p, 0) \mathcal {B}_{+}^{\infty }(\eta , \theta p-n\alpha ) + \textrm{o}(1). \end{aligned} \end{aligned}$$

Next, we rewrite:

5.115 $$\begin{aligned} \begin{aligned}&R^{\le }_{0;\ell ,N}(\eta ,\theta ) \\&\quad = -\int _{\mathbb {T}_{\theta ^{-1}}:\, 2\theta |p|_{\mathbb {T}_{\theta ^{-1}}} \le c_{N}} \frac{dp}{(2\pi )} \sum _{n: 0<|n| \le N} \sum _{x_{2},y_{2}} (Z_{+}\star \overline{Z_{+}})(-n;y_{2}) (Z_{+}\star \overline{Z_{+}})(n;x_{2}) \\&\qquad \cdot \theta {\hat{\mu }}_{\theta }(-\theta p,0) \,\theta {\hat{\phi }}_{\theta ,\ell }(\theta p,0) \mathcal {B}_{+}^{\infty }(\eta ,-n\alpha ) \\&\qquad + \int _{\mathbb {T}_{\theta ^{-1}}:\, 2\theta |p|_{\mathbb {T}_{\theta ^{-1}}} \le c_{N}}\frac{dp}{(2\pi )} \sum _{n: 0<|n| \le N} \sum _{x_{2},y_{2}}(Z_{+}\star \overline{Z_{+}})(-n;y_{2}) (Z_{+}\star \overline{Z_{+}})(n;x_{2}) \\&\qquad \cdot \theta {\hat{\mu }}_{\theta }(-\theta p,0) \,\theta {\hat{\phi }}_{\theta ,\ell }(\theta p,0) \Big ( \mathcal {B}_{+}^{\infty }(\eta , \theta p-n\alpha ) - \mathcal {B}_{+}^{\infty }(\eta ,-n\alpha )\Big ) + \textrm{o}(1). \end{aligned} \nonumber \\ \end{aligned}$$

Consider the first term in the right-hand side of (5.115). We have, using the decay properties of the test functions, and recalling the definition of (5.91) of periodized, rescaled test function:

5.116 $$\begin{aligned} \begin{aligned}&\int _{\mathbb {T}_{\theta ^{-1}}:\, 2\theta |p|_{\mathbb {T}_{\theta ^{-1}}} \le c_{N}} \frac{dp}{(2\pi )} \sum _{n: 0<|n| \le N} \sum _{x_{2},y_{2}}(Z_{+}\star \overline{Z_{+}})(-n;y_{2}) (Z_{+}\star \overline{Z_{+}})(n;x_{2}) \\&\qquad \cdot \theta {\hat{\mu }}_{\theta }(-\theta p,0) \,\theta {\hat{\phi }}_{\theta ,\ell }(\theta p,0) \mathcal {B}_{+}^{\infty }(\eta ,-n\alpha ) \\&= \int \frac{dp}{(2\pi )} \sum _{n: 0<|n| \le N} \sum _{x_{2},y_{2}}(Z_{+}\star \overline{Z_{+}})(-n;y_{2}) (Z_{+}\star \overline{Z_{+}})(n;x_{2}) \\&\qquad \cdot {\hat{\mu }}_{\infty }(-p,0) {\hat{\phi }}_{\infty ,}(p,0) \mathcal {B}_{+}^{\infty }(\eta ,-n\alpha ) + \textrm{o}_{N}(1), \end{aligned} \end{aligned}$$

where the main term in the right-hand side is now independent of θ and the new error $\textrm{o}_{N}(1)$ can be bounded proportionally to a positive power of θ for θ small enough. Consider now the second term in the right-hand side of (5.115). Using that $|n\alpha |_{\mathbb {T}} > c_{N}$, we have, for $2\theta |p| \le c_{N}$:

5.117 $$\begin{aligned} \Big | \mathcal {B}_{+}^{\infty }(\eta , \theta p-n\alpha ) - \mathcal {B}_{+}^{\infty }(\eta ,-n\alpha ) \Big | \le C_{N} \theta |p|_{\mathbb {T}_{\theta }^{-1}}. \end{aligned}$$

Plugging this bound in the second term in the right-hand side of (5.115), and using the decay properties of the test functions (5.108), we get:

5.118 $$\begin{aligned} \begin{aligned}&\Big |\int _{\mathbb {T}_{\theta ^{-1}}:\, 2\theta |p|_{\mathbb {T}_{\theta ^{-1}}} \le c_{N}} \frac{dp}{(2\pi )} \sum _{n: 0<|n| \le N} \sum _{x_{2},y_{2}} (Z_{+}\star \overline{Z_{+}})(-n;y_{2}) (Z_{+}\star \overline{Z_{+}})(n;x_{2}) \\&\qquad \cdot \theta {\hat{\mu }}_{\theta }(-\theta p,0) \,\theta {\hat{\phi }}_{\theta ,\ell }(\theta p,0) \Big ( \mathcal {B}_{+}^{\infty }(\eta , \theta p-n\alpha ) - \mathcal {B}_{+}^{\infty }(\eta ,-n\alpha )\Big ) \Big | \le C \theta . \end{aligned} \end{aligned}$$

Thus, putting together (5.113)–(5.118), and using that the main term in (5.116) is independent of θ, the bound (5.112) follows.

Combining (5.111) and (5.112), the function $R_{0;\ell ,N}(\eta ,\theta )$ satisfies the bound (5.15), with a constant $C_{N}$; hence, $R_{0;\ell ,N}(\eta ,\theta )$ can be considered together with $K^{\text {reg}}_{00}(\eta ,\theta )$.

**Conclusion.** Let $\widetilde{\chi }^{\text {main}}_{0;\ell }(\eta ,\theta )$ be the first term in the right-hand side of (5.106). As $\beta , L_{1}\rightarrow \infty$:

5.119 $$\begin{aligned} \begin{aligned} \widetilde{\chi }^{\text {main}}_{0;\ell }(\eta ,\theta )&= - \int \frac{d p}{2\pi } \sum _{x_{2},y_{2}} (Z_{+}\star \overline{Z_{+}})(0;y_{2}) (Z_{+}\star \overline{Z_{+}})(0;x_{2}) \\&\qquad \cdot {\hat{\mu }}_{\infty }( -p,\theta x_2) {\hat{\phi }}_{\infty }( p,y_{2}/\ell ) \mathcal {B}_{+}^{\infty }(\eta , \theta p) + \textrm{o}_{\beta ,L}(1), \end{aligned} \end{aligned}$$

where $\mathcal {B}_{+}^{\infty }(\eta , \theta p) = \lim _{\beta , L_{1}\rightarrow \infty } \mathcal {B}_{+}^{\beta ,L_{1}}( \eta , \theta p)$, and where $\textrm{o}_{\beta ,L}(1)$ takes into account errors vanishing as $\beta , L_{1}\rightarrow \infty$, arising from the approximation of the sum as an integral. As discussed in Appendix C:

5.120 $$\begin{aligned} \mathcal {B}_{+}^{\infty }(\eta , \theta p) = \frac{1}{4\pi \big |v_{1,+}(\lambda ) v_{0,+}(\lambda ) \big |}\frac{iv_{0,+}(\lambda )\eta -v_{1,+}(\lambda )\theta p}{i v_{0,+}(\lambda )\eta +v_{1,+}(\lambda )\theta p} + r_{+}(\eta ,\theta p), \end{aligned}$$

where $r_{+}(\eta ,\theta p)$ vanishes continuously as $(\eta , \theta ) \rightarrow (0,0)$. Now, by (5.20) we have:

5.121 $$\begin{aligned} \begin{aligned} \chi ^{\beta ,L}_{0;\ell }(\eta , \theta )&= - \int \frac{d p}{2\pi } \sum _{x_{2},y_{2}} (Z_{+}\star \overline{Z_{+}})(0;y_{2}) (Z_{+}\star \overline{Z_{+}})(0;x_{2}) \\&\cdot {\hat{\mu }}_{\infty }(- p,\theta x_2){\hat{\phi }}_{\infty }( p,y_{2} / \ell ) \big ( \mathcal {B}_{+}^{\infty }(\eta , \theta p) - \mathcal {B}_{+}^{\infty }(\eta , \eta ^{2} p)\big ) + \mathfrak {e}^{\beta ,L}_{0;\ell }(\eta ,\theta ,N), \end{aligned}\nonumber \\ \end{aligned}$$

where $\mathfrak {e}^{\beta ,L}_{0;\ell }(\eta ,\theta ,N)$ collects the error term $E_{00}^{\beta ,L}(\eta ,\theta )$ (updated to take into account also $R^{\le }_{0;N}$, Eqs. (5.107)–(5.110)) plus: the error term coming from the Wick rotation at finite temperature, Eq. (5.2), which arises if η>0 is not in $(2\pi /\beta )\mathbb {N}$; the error term (5.101); the error term (5.111); the error term coming from the integral approximation (5.119). All in all, the net error term goes to zero in the following order of limits:

5.122 $$\begin{aligned} \lim _{\ell \rightarrow \infty } \lim _{N\rightarrow \infty } \lim _{(\eta ,\theta ) \rightarrow 0} \lim _{\beta \rightarrow \infty } \lim _{L_{2}\rightarrow \infty } \lim _{L_{1}\rightarrow \infty }\mathfrak {e}^{\beta ,L}_{0;\ell }(\eta ,\theta ,N) = 0, \end{aligned}$$

where the relative order of η and θ is irrelevant, and where the $L_{1}\rightarrow \infty$ limit is taken over sequences specified in Remark 2.1 item (ii). The multiscale method used in this paper can also be used to prove the existence of the infinite volume limit of the Gibbs state, and hence of the transport coefficients; we refer the reader to e.g. [23, Section 4.4] for a proof of this statement for the quasi-periodic Ising model, which can be adapted to the present case. The existence of the $L_{2}\rightarrow \infty$ limit can also be proved, using the existence of the limit of the two-point function (which follows from Assumption 3).

In the following, we shall denote by $\mathfrak {e}^{\beta ,L}_{0,a;\ell }(\eta ,\theta )$, with a=1,2,3,4, further error terms that satisfy (5.122). Consider the main term in (5.121). Observe that, from (5.120):

5.123 $$\begin{aligned} \mathcal {B}_{+}^{\infty }(\eta , \eta ^{2} p) = \frac{1}{4\pi \big |v_{1,+}(\lambda ) v_{0,+}(\lambda ) \big |} + {\tilde{r}}(\eta , \eta p), \end{aligned}$$

where ${\tilde{r}}(\eta , \eta p)$ vanishes continuously as $(\eta , \theta ) \rightarrow (0,0)$ for all fixed *p*. Then:

5.124 $$\begin{aligned}  &   \mathcal {B}_{+}^{\infty }(\eta , \theta p) - \mathcal {B}_{+}^{\infty }(\eta , \eta ^{2} p) \nonumber \\  &   \quad = \frac{1}{2\pi \big |v_{1,+}(\lambda ) v_{0,+}(\lambda ) \big |}\frac{-v_{1,+}(\lambda )\theta p}{i v_{0,+}(\lambda )\eta +v_{1,+}(\lambda )\theta p} + r(\eta ,\theta p) - {\tilde{r}}(\eta , \eta p), \nonumber \\ \end{aligned}$$

we find:

5.125 $$\begin{aligned} \begin{aligned}&\chi ^{\beta ,L}_{0;\ell }(\eta , \theta )\\  &= \int \frac{d p}{2\pi } \sum _{x_{2},y_{2}} (Z_{+}\star \overline{Z_{+}})(0;y_{2}) (Z_{+}\star \overline{Z_{+}})(0;x_{2}) {\hat{\mu }}_{\infty }( -p,\theta x_2) {\hat{\phi }}_{\infty }( p,y_{2} / \ell )\\&\quad \cdot \frac{1}{2\pi \big |v_{1,+}(\lambda ) v_{0,+}(\lambda ) \big |} \frac{v_{1,+}(\lambda )\theta p}{i v_{0,+}(\lambda )\eta +v_{1,+}(\lambda )\theta p} + \mathfrak {e}^{\beta ,L}_{0,2;\ell }(\eta ,\theta ,N). \end{aligned} \end{aligned}$$

In order to further simplify the expression, we will rely on the relations implied by the vertex Ward identity, Eqs. (5.41). Using that:

5.126 $$\begin{aligned} \begin{aligned}&\sum _{x_{2},y_{2}} (Z_{+}\star \overline{Z_{+}})(0;y_{2}) (Z_{+}\star \overline{Z_{+}})(0;x_{2}) {\hat{\mu }}_{\infty }(- p,\theta x_2) {\hat{\phi }}_{\infty }( p,y_{2} / \ell ) \\&\qquad = v_{0,+}(\lambda )^{2} {\hat{\mu }}_{\infty }(-p, 0) {\hat{\phi }}_{\infty }(p, 0) + \textrm{o}_{\theta ,\ell }(1), \end{aligned} \end{aligned}$$

where $\textrm{o}_{\theta ,\ell }(1)$ vanishes as $\theta \rightarrow 0$ and $\ell \rightarrow \infty$, we get:

5.127 $$\begin{aligned} \chi ^{\beta ,L}_{0;\ell }(\eta , \theta ) {=} \int \frac{dp}{(2\pi )^2} {\hat{\mu }}_{\infty }(- p, 0) {\hat{\phi }}_{\infty }( p,0)\frac{\text {sgn}(v_{1,+})\,\theta p}{i \eta {+} \mathfrak {v}(\lambda ) \theta p}{+} \mathfrak {e}^{\beta ,L}_{0,3;\ell }(\eta ,\theta ,N), \end{aligned}$$

where $\mathfrak {v}(\lambda ):= \lim _{\beta \rightarrow \infty } \lim _{L_{2},L_{1}\rightarrow \infty } v_{1,+} / v_{0,+}$. In particular, for $\eta \ll \theta$:

5.128 $$\begin{aligned} \chi ^{\beta ,L}_{0;\ell }(\eta , \theta ) = \frac{1}{2\pi |\mathfrak {v}(\lambda )|} \langle \mu , \phi \rangle _{\text {edge}} + \mathfrak {e}^{\beta ,L}_{0,4;\ell }(\eta ,\theta ,N), \end{aligned}$$

which proves the first of (3.12).

#### Edge Conductance

To conclude the proof of Theorem 3.3, let us consider the edge conductance and prove the second of (3.12). Recall the expression of the edge conductance after Wick rotation, for $\eta \in (2\pi / \beta ) \mathbb {N}$:

5.129 $$\begin{aligned} \chi _{1;\ell }^{\beta ,L}(\eta , \theta ) = \theta \sum _{\vec x,\vec y } \mu (\theta \vec x) \phi _{\ell }(\theta y_{1}, y_{2}) \int _{-\beta /2}^{\beta /2} d s\, e^{-i\eta s} \langle \textbf{T}\gamma _{s}(n_{\vec x}); j_{1,\vec y} \rangle _{\beta ,\mu ,L}, \nonumber \\ \end{aligned}$$

where the current operator is:

5.130 $$\begin{aligned} j_{1,\vec y}:= j_{\vec y,\vec y+\vec e_1}+\frac{1}{2}\big ( j_{\vec y,\vec y+\vec e_1+\vec e_2}+ j_{\vec y,\vec y+\vec e_1-\vec e_2}+ j_{\vec y+\vec e_2,\vec y+\vec e_1}+ j_{\vec y-\vec e_2,\vec y+\vec e_1}\big ), \end{aligned}$$

and the bond current is:

5.131 $$\begin{aligned} j_{\vec y,\vec z} = i\sum _{\sigma ,\zeta } \big ( H_{\zeta ,\sigma }(\vec y,\vec z) a^*_{\vec y,\zeta } a_{\vec z,\sigma }-H_{\sigma ,\zeta }(\vec z,\vec y) a^*_{\vec z,\sigma } a_{\vec y,\zeta }\big ). \end{aligned}$$

We will compute the edge conductance by studying all the contributions associated with the bond currents, and then we will put everything together. To this end, let us introduce the notation, for $\vec a,\vec b\in \Lambda _{L}$:

5.132 $$\begin{aligned} j_{y}^{ab}:= i\sum _{\sigma ,\zeta } H^{ab}_{\zeta ,\sigma }(y_{2}) a^*_{\vec y + \vec a,\zeta } a_{\vec y + \vec b,\sigma },\qquad H^{ab}_{\zeta ,\sigma }(y_{2}):= H_{\zeta ,\sigma }(\vec y + \vec a, \vec y + \vec b); \nonumber \\ \end{aligned}$$

by translation-invariance in the $y_{1}$ direction, the right-hand side of the second of (5.132) only depends on $\vec a, \vec b, y_{2}$. It is clear that the edge current can be expressed as a combination of the operators $j_{y}^{ab}$. Thus, consider (dropping the $\beta , \mu , L$ labels in the state):

5.133 $$\begin{aligned} \begin{aligned} \chi _{ab;\ell }^{\beta ,L}(\eta , \theta )&= \theta \sum _{\vec x,\vec y } \mu (\theta \vec x) \phi _{\ell }(\theta y_{1}, y_{2}) \int _{-\frac{\beta }{2}}^{\frac{\beta }{2}} d s\, e^{-i\eta s} \langle \textbf{T}\gamma _{s}(n_{\vec x}); j^{ab}_{\vec y} \rangle \\&= i\theta \sum _{\vec x,\vec y }\sum _{\sigma ,\zeta }\mu (\theta \vec x) \phi _{\ell }(\theta y_{1}, y_{2}) H^{ab}_{\zeta ,\sigma }(y_{2})\\&\quad \cdot \int _{-\frac{\beta }{2}}^{\frac{\beta }{2}} d s\, e^{-i\eta s} \langle \textbf{T}\gamma _{s}(n_{\vec x}); a^*_{\vec y + \vec a,\zeta } a_{\vec y + \vec b,\sigma } \rangle . \end{aligned} \end{aligned}$$

We can evaluate the average by Wick’s rule. We get:

5.134 $$\begin{aligned} \begin{aligned} \chi _{ab;\ell }^{\beta ,L}(\eta , \theta )&= -i\theta \sum _{\vec x,\vec y }\sum _{\sigma ,\zeta ,\rho } \mu (\theta \vec x) \phi _{\ell }(\theta y_{1}, y_{2}) H^{ab}_{\zeta ,\sigma }(y_{2}) \\&\quad \cdot \int _{-\frac{\beta }{2}}^{\frac{\beta }{2}} d s\, e^{-i\eta s} S_{2;\rho ,\zeta }\big ((\vec x, s); (\vec y + \vec a, 0)\big ) S_{2;\sigma ,\rho }\big ((\vec y + \vec b, 0); (\vec x, s)\big ). \end{aligned} \end{aligned}$$

As for the susceptibility, we define the singular part of $\chi _{ab;\ell }^{\beta ,L}(\eta , \theta )$ as:

5.135 $$\begin{aligned} \begin{aligned} \chi _{ab;\ell }^{\text {sing}}(\eta , \theta )&= -i\theta \sum _{\vec x,\vec y } \sum _{\sigma ,\zeta ,\rho }\mu (\theta \vec x) \phi _{\ell }(\theta y_{1}, y_{2}) H^{ab}_{\zeta ,\sigma }(y_{2}) \\&\quad \cdot \int _{-\frac{\beta }{2}}^{\frac{\beta }{2}} d s\, e^{-i\eta s} S^{\textrm{s}}_{2;\rho ,\zeta }\big ((\vec x, s); (\vec y + \vec a, 0)\big ) S^{\textrm{s}}_{2;\sigma ,\rho }\big ((\vec y + \vec b, 0); (\vec x, s)\big ). \end{aligned} \end{aligned}$$

By Theorem 3.1:

5.136 $$\begin{aligned} \begin{aligned}&S^{\textrm{s}}_{2;\rho ,\zeta }\big ((\vec x, s); (\vec y + \vec a, 0)\big )\\&\quad = \sum _{\omega } e^{i k_{F}^{\omega }(\lambda )(x_{1} - y_{1} - a_{1})}Z_{\omega ,\rho }(\vec x) \check{g}_{\textrm{s};\omega }\big (( x_{1},s); (y_{1} +a_{1}, 0)\big ) \overline{Z_{\omega ,\zeta }(y_{1} + a_{1})} \\&S^{\textrm{s}}_{2;\sigma ,\rho }\big (( y_{1} + b_{1}, 0); ( x_{1}, s)\big ) \\&\quad = \sum _{\omega } e^{i k_{F}^{\omega }(\lambda )(y_{1} + b_{1} - x_{1})}Z_{\omega ,\sigma }(\vec y + \vec b) \check{g}_{\textrm{s};\omega }\big ( y_{1}+ b_{1},0); ( x_{1}, s)\big ) \overline{Z_{\omega ,\rho }(\vec x)}. \end{aligned} \end{aligned}$$

Now, for the purpose of determining the singular part of the response function, we can safely drop $a_{1}$ and $b_{1}$ in the relativistic propagator. In fact:

5.137 $$\begin{aligned} \big | \check{g}_{\textrm{s};\omega }\big (( y_{1} + b_{1},0); ( x_{1}, s)\big ) - \check{g}_{\textrm{s};\omega }\big (( y_{1},0); (x_{1}, s)\big )\big | \le \frac{C_{b}}{1 + \Vert ( x_{1}, s) - ( y_{1}, 0) \Vert ^{1+\alpha }}, \nonumber \\ \end{aligned}$$

with α>0. Since in our application $|\vec b| \le 2$, the above bound proves that the difference between the propagators has improved decay. We use this observation to rewrite:

5.138 $$\begin{aligned} \begin{aligned} \chi _{ab;\ell }^{\text {sing}}(\eta , \theta )&= -i\theta \sum _{\vec x,\vec y }\sum _{\sigma ,\zeta ,\rho } \mu (\theta \vec x) \phi _{\ell }(\theta y_{1}, y_{2}) e^{i k_{F}^{+}(\lambda )(b_{1} - a_{1})}H^{ab}_{\zeta ,\sigma }(y_{2}) \\&\qquad \cdot Z_{+,\rho }(\vec x) \overline{Z_{+,\zeta }(\vec y + \vec a)} Z_{+,\sigma }(\vec y + \vec b) \overline{Z_{+,\rho }(\vec x)} \\&\qquad \cdot \int _{-\frac{\beta }{2}}^{\frac{\beta }{2}} d s\, e^{-i\eta s} \check{g}_{\textrm{s};+}\big (( x_{1},s); ( y_{1}, 0)\big ) \check{g}_{\textrm{s};+}\big (( y_{1},0); ( x_{1}, s)\big )\\&\qquad + \mathfrak {e}_{1,1}(\eta , \theta ) + \mathfrak {e}_{1,2}(\eta , \theta ) \end{aligned} \end{aligned}$$

where $\mathfrak {e}_{1,1}(\eta , \theta )$ collects the contributions coming from the chiralities associated with the edge mode as $x_{2} = L_{2}$, and $\mathfrak {e}_{1,2}(\eta , \theta )$ the contribution coming from the difference of the propagators (5.137). Therefore,

5.139 $$\begin{aligned} | \mathfrak {e}_{1,1}(\eta , \theta ) | \le C_{\theta ,\ell } e^{-cL_{2}}, \end{aligned}$$

while $\mathfrak {e}_{1,2}(\eta , \theta )$ satisfies the bound (5.15), and hence it can be absorbed into the regular part. Now, let $Z_{+,\zeta }(\vec y + \vec a) \equiv Z^{a}_{+,\zeta }(\vec y)$, where:

5.140 $$\begin{aligned} Z^{a}_{+,\zeta }(\vec y) = \sum _{n} e^{-i n \alpha y_{1}} e^{-i n \alpha a_{1}} Z_{+,n,\zeta }(y_{2} + a_{2}) \equiv \sum _{n} e^{-i n \alpha y_{1}} Z^{a}_{+,n,\zeta }(y_{2}). \end{aligned}$$

Let us denote by $\widetilde{\chi }^{\text {sing}}_{ab}(\eta ,\theta )$ the main contribution to the right-hand side of (5.138). Using the above notation, we have:

5.141 $$\begin{aligned} \begin{aligned}&{\widetilde{\chi }}_{ab;\ell }^{\text {sing}}(\eta , \theta )= -i\theta \sum _{\vec x,\vec y } \sum _{\sigma ,\zeta ,\rho } \mu (\theta \vec x) \phi _{\ell }(\theta y_{1}, y_{2}) e^{i k_{F}^{+}(\lambda )(b_{1} - a_{1})}H^{ab}_{\zeta ,\sigma }(y_{2}) Z_{+,\rho }(\vec x)\overline{Z^{a}_{+,\zeta }(\vec y)} \\&\qquad \cdot Z^{b}_{+,\sigma }(\vec y) \overline{Z_{+,\rho }(\vec x)} \int _{-\frac{\beta }{2}}^{\frac{\beta }{2}} d s\, e^{-i\eta s} \check{g}_{\textrm{s};+}\big (( x_{1},s); (y_{1}, 0)\big ) \check{g}_{\textrm{s};+}\big (( y_{1},0); ( x_{1}, s)\big ), \end{aligned} \end{aligned}$$

which we rewrite as, using (5.93):

5.142 $$\begin{aligned} \begin{aligned} {\widetilde{\chi }}_{ab;\ell }^{\text {sing}}(\eta , \theta )&= -i\theta \sum _{\vec x,\vec y } \sum _{\sigma ,\zeta ,\rho } \mu (\theta \vec x) \phi _{\ell }(\theta y_{1}, y_{2}) e^{i k_{F}^{+}(\lambda )(b_{1} - a_{1})}H^{ab}_{\zeta ,\sigma }(y_{2}) \\&\qquad \cdot Z_{+,\rho }(\vec x) \overline{Z^{a}_{+,\zeta }(\vec y)} Z^{b}_{+,\sigma }(\vec y) \overline{Z_{+,\rho }(\vec x)} \frac{1}{L_{1}} \sum _{p} e^{ip (x_{1} - y_{1})} \mathcal {B}^{\beta ,L_{1}}_{+}(\eta ,p). \end{aligned} \end{aligned}$$

That is:

5.143 $$\begin{aligned} \begin{aligned} {\widetilde{\chi }}_{ab;\ell }^{\text {sing}}(\eta , \theta )&= -\frac{\theta }{L_{1}}\sum _{p} \sum _{x_{2},y_{2}}\sum _{\sigma ,\zeta ,\rho }\mathcal {F}\big (\mu (\theta \cdot , \theta x_{2}) |Z_{+,\rho } (\cdot ,x_{2})|^{2}\big )(-p) \\&\qquad \cdot \mathcal {F}\big (\phi ^{ab}_{\ell ;\zeta ,\sigma }(\theta \cdot ,y_{2}) Z^{b}_{+,\sigma }(\cdot ,y_{2}) \overline{Z^{a}_{+,\zeta }(\cdot , y_{2})}\big )(p) \mathcal {B}_{+}^{\beta , L_{1}}(\eta , p), \end{aligned} \end{aligned}$$

where we set:

5.144 $$\begin{aligned} \phi ^{ab}_{\ell ;\zeta ,\sigma }(\theta y_{1}, y_{2}):= i\phi _{\ell }(\theta y_{1}, y_{2}) e^{i k_{F}^{+}(\lambda )(b_{1} - a_{1})} H^{ab}_{\zeta ,\sigma }(y_{2}). \end{aligned}$$

The next step is to expand the oscillatory factors into their Fourier series, and proceed as after (5.96) to isolate the main singular term from other contributions that are either small or regular in $\eta , \theta$; the only difference in the present setting is the *a*, *b* decorations of the Fourier coefficients, and the redefinition (5.144) of the test function. Both are completely irrelevant for the purpose of the discussion following (5.94) till (5.119). Thus, we get:

5.145 $$\begin{aligned} \begin{aligned} {\widetilde{\chi }}_{ab;\ell }^{\text {sing}}(\eta , \theta )&= - \int \frac{d p}{2\pi } \sum _{x_{2},y_{2}} \sum _{\sigma , \zeta ,\rho } (Z^{b}_{+,\sigma }\star \overline{Z^{a}_{+,\zeta }})(0;y_{2}) (Z_{+,\rho }\star \overline{Z_{+,\rho }})(0;x_{2}) \\&\qquad \cdot {\hat{\mu }}_{\infty }( -p,\theta x_2) {\hat{\phi }}^{ab}_{\infty ; \zeta ,\sigma }( p,y_{2} / \ell ) \mathcal {B}_{+}^{\infty }(\eta , \theta p) + \textrm{o}(1), \end{aligned} \end{aligned}$$

where:

5.146 $$\begin{aligned} {\hat{\phi }}^{ab}_{\infty ;\zeta ,\sigma }( -p,y_{2} / \ell ) = i \hat{\phi }_{\infty }(-p,y_{2} / \ell ) e^{i k_{F}^{+}(\lambda )(b_{1} - a_{1})} H^{ab}_{\zeta ,\sigma }(y_{2}). \end{aligned}$$

Up to vanishing error terms as $\theta \rightarrow 0$, $\ell \rightarrow \infty$, we can localize the test functions on the boundary. We get:

5.147 $$\begin{aligned} \begin{aligned} {\widetilde{\chi }}_{ab;\ell }^{\text {sing}}(\eta , \theta ) =&- \Big (\int \frac{d p}{2\pi }\, {\hat{\mu }}_{\infty }( -p, 0){\hat{\phi }}_{\infty }(p,0) \mathcal {B}_{+}^{\infty }(\eta , \theta p)\Big )\\&\quad \cdot i v_{0,+}(\lambda ) \sum _{\sigma ,\zeta }\sum _{y_{2}} (Z^{b}_{+,\sigma }*\overline{Z^{a}_{+,\zeta }})(0;y_{2}) e^{i k_{F}^{+}(\lambda )(b_{1} - a_{1})} H^{ab}_{\zeta ,\sigma }(y_{2}) + \textrm{o}(1), \end{aligned} \nonumber \\ \end{aligned}$$

where we used the first of the relations (5.41), as for the susceptibility. Let us now consider the last factor. We rewrite it as:

5.148 $$\begin{aligned} \begin{aligned}&i\sum _{\sigma ,\zeta } \sum _{y_{2}} (Z^{b}_{+,\sigma }*\overline{Z^{a}_{+,\zeta }})(0;y_{2}) e^{i k_{F}^{+}(\lambda )(b_{1} - a_{1})} H^{ab}_{\zeta ,\sigma }(y_{2}) \\&\quad =i \sum _{n} e^{i (k_{F}^{+} - n\alpha )(b_{1} - a_{1})}\sum _{y_{2}} \big \langle Z_{+,n}(y_{2} + a_{2}), H_{\zeta ,\sigma }(\vec y + \vec a; \vec y + \vec b) Z_{+,n}(y_{2} + b_{2})\big \rangle , \end{aligned} \nonumber \\ \end{aligned}$$

recall (5.132) and (5.140), and where the scalar product is over the internal degrees of freedom as in. We now have to add up all the possible choices of $(\vec a, \vec b)$, and subtracting the case $\vec a \leftrightarrow \vec b$, because of the definition of bond current (5.131). From (5.130), the possible choices for $(\vec a, \vec b)$, associated with the bond currents, are:

5.149 $$\begin{aligned} (0, \vec e_{1}),\quad (0, \vec e_{1} + \vec e_{2}),\quad (0, \vec e_{1} - \vec e_{2}),\quad (\vec e_{2}, \vec e_{1}),\quad (-\vec e_{2}, \vec e_{1}), \end{aligned}$$

minus the cases $\vec a\leftrightarrow \vec b$. Except for the first case in (5.149), the other bond currents come with a factor 1/2. However, when summing over $y_{2}$, second case and the fourth case, and the third case and the fifth case, give the same outcome, using the Dirichlet boundary conditions; recall Eq. (5.34).

Therefore, summing over all bond currents we get:

5.150 $$\begin{aligned} \begin{aligned}&i\sum _{n} e^{i (k_{F}^{+}(\lambda ) - n\alpha )} \sum _{y_{2}} \big \langle Z_{+,n}(y_{2}), H(\vec y; \vec y + \vec e_{1}) Z_{+,n}(y_{2})\big \rangle \\&+ i\sum _{n} e^{i (k_{F}^{+}(\lambda ) - n\alpha )} \sum _{y_{2}} \big \langle Z_{+,n}(y_{2}), H(\vec y; \vec y + \vec e_{1} + \vec e_{2}) Z_{+,n}(y_{2} + 1)\big \rangle \\&+ i\sum _{n} e^{i (k_{F}^{+}(\lambda ) - n\alpha )} \sum _{y_{2}} \big \langle Z_{+,n}(y_{2}), H(\vec y; \vec y + \vec e_{1} - \vec e_{2}) Z_{+,n}(y_{2} - 1)\big \rangle + \text {c.c.;} \end{aligned} \end{aligned}$$

adding the complex conjugate is the same as subtracting the cases $\vec a\leftrightarrow \vec b$. Recalling (5.35) and (5.36), we rewrite the first term as:

5.151 $$\begin{aligned} \begin{aligned}&i\sum _{n} e^{i (k_{F}^{+}(\lambda ) - n\alpha )} \sum _{y_{2}} \big \langle Z_{+,n}(y_{2}), H(\vec y; \vec y + \vec e_{1}) Z_{+,n}(y_{2})\big \rangle + \text {c.c.} \\&= \sum _{n} \big \langle Z_{+,n}(y_{2}), \partial _{k_{1}} {\hat{H}}\big (k_{F}^{+}(\lambda ) - n\alpha ; y_{2}, y_{2}\big ) Z_{+,n}(y_{2}) \big \rangle , \end{aligned} \end{aligned}$$

Repeating the computation for the other two cases in (5.150), we see that:

5.152 $$\begin{aligned} (5.150) = \sum _{n} \big \langle Z_{+,n}, \partial _{k_{1}} {\hat{H}}\big (k_{F}^{+}(\lambda ) - n\alpha \big ) Z_{+,n} \big \rangle , \end{aligned}$$

where now the scalar product is over the internal degrees of freedom and over the second spatial variable. These quantity is precisely the coefficient $\zeta _{1,+}$, appearing in the analysis of the vertex function. By the second of (5.41), we know that $\zeta _{1,+} = v_{1,+}$. Coming back to the edge conductance, we obtained the following representation for its singular contribution:

5.153 $$\begin{aligned} {\widetilde{\chi }}^{\text {sing}}_{1;\ell }(\eta , \theta ) = v_{0,+}(\lambda ) v_{1,+}(\lambda ) \Big (\int \frac{d p}{2\pi }\, \hat{\mu }_{\infty }( -p, 0){\hat{\phi }}_{\infty }(p,0) \mathcal {B}_{+}^{\infty }(\eta , \theta p)\Big ) + \textrm{o}(1). \nonumber \\ \end{aligned}$$

By the consequence of the current-current Ward identity (5.26), we obtain the analogue of (5.121), (5.127) namely:

5.154 $$\begin{aligned} \chi ^{\beta ,L}_{1;\ell }(\eta , \theta ) = \int \frac{dp}{(2\pi )^2} {\hat{\mu }}_{\infty }( -p, 0) {\hat{\phi }}_{\infty }( p,0)\frac{|\mathfrak {v}(\lambda )|\theta p}{i \eta + \mathfrak {v}(\lambda ) \theta p} + \tilde{\mathfrak {e}}^{\beta ,L}_{1;\ell }(\eta ,\theta ,N). \nonumber \\ \end{aligned}$$

In particular, for $\eta \ll \theta$:

5.155 $$\begin{aligned} \chi ^{\beta ,L}_{1;\ell }(\eta , \theta ) = \frac{\text {sgn}(\mathfrak {v}(\lambda ))}{2\pi } \langle \mu , \varphi \rangle _{\text {edge}} + \mathfrak {e}_{1;\ell }^{\beta ,L}(\eta ,\theta ,N). \end{aligned}$$

This proves the second of (3.12), and concludes the proof of Theorem 3.3. □

## Data Availability

This manuscript has no associated data.

## Bound on the Propagator Away from the Fermi Level

In this appendix we shall prove the bound (4.12) for the propagator associated with energies away from the Fermi level μ. Given the definition of cutoff function $\chi (\cdot )$, Eq. (3.1), recall:

A.1 $$\begin{aligned} \begin{aligned} G^{(\text {b})}(\boldsymbol{k},x_2, y_2)&= \Big (\big (1-\chi (k_{0}) \chi \big ({\hat{H}}(k_{1}) - \mu \big )\big ) G(\boldsymbol{k})\Big )(x_{2},y_{2}) \\&= (1-\chi (k_{0})) G(\boldsymbol{k},x_2, y_2) + \chi (k_{0}) \Big (\big (1 - \chi \big ({\hat{H}}(k_{1}) - \mu \big )\big ) G(\boldsymbol{k})\Big )(x_{2},y_{2}) \\&\equiv G^{(\text {b}); 1}(\boldsymbol{k},x_2, y_2) + G^{(\text {b}); 2}(\boldsymbol{k},x_2, y_2). \end{aligned}\nonumber \\ \end{aligned}$$

For the first term in (A.1), the function $(1-\chi (k_{0}))$ implies that $|k_{0}| \ge C\delta$. For a finite-range Hamiltonian *H*, the Combes-Thomas bound states that, for $z \notin \sigma (H)$, see e.g. [1]:

A.2 $$\begin{aligned} \Big \Vert \frac{1}{H - z}(x_{2}, y_{2}) \Big \Vert \le \frac{C}{|\text {dist}(z,\sigma (H))|} e^{-c|x_{2} -y_{2}|}, \end{aligned}$$

where the constant *c* depends on the distance from *z* to the spectrum of *H*. Thus, in our case, for $|k_{0}|\ge C\delta$:

A.3 $$\begin{aligned} \Big \Vert G^{(\text {b}); 1}(\boldsymbol{k},x_2, y_2)\Big \Vert \le \frac{C_{\delta }}{1+|k_{0}|} e^{-c|x_{2}- y_{2}|}. \end{aligned}$$

Consider now the term $G^{(\text {b}); 2}_{\sigma ,\zeta }(\boldsymbol{k},x_2, y_2)$. By the approximate linearity of the edge modes close to $k_{F}^{\omega }$, if $|k_{1} - k_{F}^{\omega }| \ge \kappa \delta / v_{\omega }$ for all ω and for a suitable κ>0 then:

A.4 $$\begin{aligned} G^{(\text {b}); 2}(\boldsymbol{k},x_2, y_2) = \frac{\chi (k_{0})}{ik_{0} + {\hat{H}}(k_{1})- \mu }(x_{2}, y_{2}) \end{aligned}$$

and $\text {dist}(\mu , \sigma ({\hat{H}}(k_{1}))) \ge c\delta$. Therefore, we can apply the Combes-Thomas bound and we get:

A.5 $$\begin{aligned} \Big \Vert G^{(\text {b}); 2}(\boldsymbol{k},x_2, y_2)\Big \Vert \le \frac{C_{\delta }}{|k_{0}| + 1} e^{-c|x_{2} - y_{2}|}. \end{aligned}$$

Finally, suppose that $|k_{1} - k_{F}^{\omega }| < \delta / v_{\omega }$ for some ω. Let us introduce the short-hand notation:

A.6 $$\begin{aligned} \chi _{>\delta }({\hat{H}}(k_{1})):= 1 - \chi \big ({\hat{H}}(k_{1}) - \mu \big ). \end{aligned}$$

Observe that the function $\chi _{>\delta }(\lambda )$ is equal to 1 for $|\lambda - \mu | \ge 2\delta$, it vanishes for $|\lambda | \le \delta$ and it is between 0 and 1 for $\delta \le |\lambda - \mu | \le 2\delta$. We write:

A.7 $$\begin{aligned} \begin{aligned} G^{(\text {b}); 2}(\boldsymbol{k}) = \chi (k_{0}) \chi _{>\delta }({\hat{H}}(k_{1})) P G_{\sigma ,\zeta }(\boldsymbol{k}) + \chi (k_{0}) \chi _{>\delta }({\hat{H}}(k_{1})) P^{\perp } G_{\sigma ,\zeta }(\boldsymbol{k}) \end{aligned} \end{aligned}$$

where $P = 1\!\!1(|{\hat{H}}(k_{1}) - \mu | \le 2\delta )$. Observe that, by the assumptions on the Hamiltonian (Assumption 3), $P = \sum _{e: |\varepsilon _{e}(k_{1}) - \mu | \le 2\delta } P_{e}$, with $P_{e}$ the projector over the edge mode of ${\hat{H}}(k_{1})$ labelled by *e*. Also,

A.8 $$\begin{aligned} \chi _{>\delta }({\hat{H}}(k_{1})) P^{\perp } = P^{\perp },\qquad P^{\perp } \frac{1}{ik_{0} + {\hat{H}}(k_{1}) - \mu } = P^{\perp } \frac{1}{ik_{0} + P^{\perp }{\hat{H}}(k_{1}) - \mu }. \nonumber \\ \end{aligned}$$

Consider the first term in the right-hand side of (A.7). We have:

A.9 $$\begin{aligned} \chi (k_{0}) \chi _{>\delta }({\hat{H}}(k_{1})) P G_{\sigma ,\zeta }(\boldsymbol{k}) = \chi (k_{0})\sum _{e: |\varepsilon _{e}(k_{1}) - \mu | \le 2\delta } \frac{\chi _{>\delta }(\varepsilon _{e}(k_{1}))}{ik_{0} + \varepsilon _{e}(k_{1}) - \mu } P_{e}(x_{2};y_{2}).\nonumber \\ \end{aligned}$$

By the exponential decay of the edge modes, we easily get:

A.10 $$\begin{aligned} \Big \Vert \chi (k_{0}) \chi _{>\delta }({\hat{H}}(k_{1})) P G_{\sigma ,\zeta }(\boldsymbol{k}) \Big \Vert \le C_{\delta } \chi (k_{0}) e^{-|x_{2} - y_{2}|}. \end{aligned}$$

Consider now the second term in the right-hand side of (A.7). Here we have:

A.11 $$\begin{aligned} \chi (k_{0}) \chi _{>\delta }({\hat{H}}(k_{1})) P^{\perp } G_{\sigma ,\zeta }(\boldsymbol{k}) = \chi (k_{0}) P^{\perp } \frac{1}{ik_{0} + P^{\perp }{\hat{H}}(k_{1}) - \mu }, \end{aligned}$$

and

A.12 $$\begin{aligned} P^{\perp }{\hat{H}}(k_{1}) = {\hat{H}}(k_{1}) - \sum _{e: |\varepsilon _{e}(k_{1}) - \mu | \le 2\delta } \varepsilon _{e}(k_{1}) P_{e} =: \widetilde{H}(k_{1}). \end{aligned}$$

By Assumption 3, we observe that $\text {dist}(\mu , \sigma (\widetilde{H}(k_{1}))) \ge 2\delta$. The Hamiltonian $\widetilde{H}(k_{1})$ is not finite-range, due to the projectors of the edge modes. However, one can check that, for |α| sufficiently small:

A.13 $$\begin{aligned} \Vert e^{\alpha {\hat{x}}_{2}} \widetilde{H}(k_{1}) e^{-\alpha {\hat{x}}_{2}} - \widetilde{H}(k_{1}) \Vert \le C|\alpha |. \end{aligned}$$

This bound is all it is needed to prove the Combes-Thomas estimate for the resolvent of $\widetilde{H}(k_{1})$; it follows from the short-range of ${\hat{H}}(k_{1})$, and from the exponential decay of the edge modes. Thus, from the exponential decay of $P^{\perp }(x_{2}, y_{2}) = 1\!\!1(x_{2},y_{2}) - P(x_{2},y_{2})$, and from the Combes-Thomas bound for the resolvent of $\widetilde{H}(k_{1})$, we obtain:

A.14 $$\begin{aligned} \Big \Vert \chi (k_{0}) \Big ( P^{\perp } \frac{1}{ik_{0} + P^{\perp }{\hat{H}}(k_{1}) - \mu }\Big ) (x_{2},y_{2}) \Big \Vert \le C_{\delta } \chi (k_{0}) e^{-c|x_{2} - y_{2}|}. \end{aligned}$$

Finally, from (A.3), (A.5), (A.10), (A.14), we get:

A.15 $$\begin{aligned} \Big \Vert G^{(\text {b})}(\boldsymbol{k},x_2, y_2) \Big \Vert \le \frac{C_{\delta }}{|k_{0}| + 1} e^{-c|x_{2} - y_{2}|}. \end{aligned}$$

The bound for the derivatives of $G^{(\text {b})}$ can be proved in the same way, using the exponential decay of the derivatives of the edge modes, Assumption 3. We omit the details.

## Recursion Relation for the Two-point Function

In this appendix we shall prove the coupled recursion relations (4.133), (4.134).

**Proof of** (4.133). To begin, we write:

B.1 $$\begin{aligned} W_{\phi \phi ;n,\sigma ,\zeta }^{(h)}({\boldsymbol{k}}; x_{2}, y_{2}) = \beta L_{1} \frac{\partial ^{2}}{\partial \phi ^{-}_{{\boldsymbol{k}} + n{\boldsymbol{\alpha }}, y_{2}, \zeta }\partial \phi ^{+}_{{\boldsymbol{k}}, x_{2}, \sigma }} \log \mathbb{E}_{h}\Big ( e^{V^{(h)}(\psi ^{(\le h)}, \phi )} \Big )\Big |_{\phi =\psi ^{(<h)} = 0} \nonumber \\ \end{aligned}$$

where $\mathbb{E}_{h}$ is the Gaussian expectation associated with $\widetilde{P}_{h}(d\psi ^{(h)})$, recall (4.129), and $V^{(h)}(\psi ^{(\le h)}, \phi )$ is a short-hand notation for the argument of the exponential in (4.129). We compute:

B.2 $$\begin{aligned} \begin{aligned}&\frac{\partial ^{2}}{\partial \phi ^{-}_{{\boldsymbol{k}} + n{\boldsymbol{\alpha }}, y_{2}, \zeta }\partial \phi ^{+}_{{\boldsymbol{k}}, x_{2}, \sigma }} \log \mathbb{E}_{h}\Big ( e^{V^{(h)}(\psi ^{(\le h)}, \phi )} \Big )\Big |_{\psi ^{(<h)} = \phi = 0} \\&\quad = \frac{1}{(\beta L_{1})^{2}} \sum _{m,\omega } W^{(h)}_{\phi \psi ;m,\omega ,\sigma }({\boldsymbol{q}}, x_{2}) \sum _{m',\omega '} W^{(h)}_{\psi \phi ;m',\omega ',\zeta }({\boldsymbol{q}} + (n-m'){\boldsymbol{\alpha }} + s_{\omega \omega '}, y_{2}) \\&\qquad \cdot \frac{\mathbb{E}_{h}\Big ( \psi ^{(\le h)-}_{{\boldsymbol{q}} + m{\boldsymbol{\alpha }}, \omega } \psi ^{(\le h)+}_{{\boldsymbol{q}} + (n-m'){\boldsymbol{\alpha }} + s_{\omega \omega '}, \omega '} e^{V^{(h)}(\psi ^{(\le h)}, 0)} \Big ) }{\mathbb{E}_{h}\Big ( e^{V^{(h)}(\psi ^{(\le h)}, 0)} \Big )}\Big |_{\psi ^{(<h)} = 0}, \end{aligned}\nonumber \\ \end{aligned}$$

where we used that the average of an odd number of Grassmann variables is zero. Next, by Grassmann integration by parts:

B.3 $$\begin{aligned} \mathbb {E}_{h}(\psi ^{(h)-}_{{\boldsymbol{q}}, \omega } A)= &   \beta L_{1} g^{(h)}_{\omega }({\boldsymbol{q}}) \mathbb{E}_{h}\Big ( \frac{\partial }{\partial \psi ^{(h)+}_{{\boldsymbol{q}},\omega }}A \Big ),\nonumber \\ \mathbb {E}_{h}(\psi ^{(h)+}_{{\boldsymbol{q}}, \omega } A)&\,=&-\beta L_{1} g^{(h)}_{\omega }({\boldsymbol{q}}) \mathbb{E}_{h}\Big ( \frac{\partial }{\partial \psi ^{(h)-}_{{\boldsymbol{q}},\omega }}A \Big ). \end{aligned}$$

We use these identities to write:

B.4 $$\begin{aligned} \begin{aligned}&\mathbb{E}_{h}\Big ( \psi ^{(h)-}_{{\boldsymbol{q}} + m{\boldsymbol{\alpha }}, \omega } \psi ^{(h)+}_{{\boldsymbol{q}} + (n-m'){\boldsymbol{\alpha }} + s_{\omega \omega '}, \omega '} e^{V^{(h)}(\psi ^{(\le h)}, 0)} \Big ) \\&\qquad \qquad = \delta _{\omega ,\omega '} \delta _{n-m',m} \beta L_{1} g^{(h)}_{\omega }({\boldsymbol{q}} + m{\boldsymbol{\alpha }}) \mathbb{E}_{h}\Big ( e^{V^{(h)}(\psi ^{(\le h)}, 0)} \Big ) \\&\qquad \qquad \quad + (\beta L_{1})^{2} g^{(h)}_{\omega }({\boldsymbol{q}} + m{\boldsymbol{\alpha }}) g^{(h)}_{\omega '}({\boldsymbol{q}} + (n-m'){\boldsymbol{\alpha }} + s_{\omega \omega '}) \\  &\qquad \qquad \qquad \cdot \mathbb{E}_{h}\Big ( \frac{\partial ^{2}}{ \partial \psi ^{(h)-}_{{\boldsymbol{q}} + (n-m'){\boldsymbol{\alpha }} + s_{\omega \omega '}, \omega '} \partial \psi ^{(h)+}_{{\boldsymbol{q}} + m{\boldsymbol{\alpha }},\omega }} e^{V^{(h)}(\psi ^{(\le h)}, 0)} \Big ). \end{aligned} \end{aligned}$$

Thus, from this formula we get:

B.5 $$\begin{aligned} \begin{aligned}&\frac{\mathbb{E}_{h}\Big ( \psi ^{(\le h)-}_{{\boldsymbol{q}} + m{\boldsymbol{\alpha }}, \omega } \psi ^{(\le h)+}_{{\boldsymbol{q}} + (n-m'){\boldsymbol{\alpha }} + s_{\omega \omega '}, \omega '} e^{V^{(h)}(\psi ^{(\le h)}, 0)} \Big ) }{\mathbb{E}_{h}\Big ( e^{V^{(h)}(\psi ^{(\le h)}, 0)} \Big )}\Big |_{\psi ^{(<h)} = 0} \\&\quad = \delta _{\omega ,\omega '}\delta _{n-m',m}\beta L_{1} g^{(h)}_{\omega }({\boldsymbol{q}} + m{\boldsymbol{\alpha }}) \\&\qquad \quad + (\beta L_{1})^{2} g^{(h)}_{\omega }({\boldsymbol{q}} + m{\boldsymbol{\alpha }}) g^{(h)}_{\omega '}({\boldsymbol{q}} + (n-m'){\boldsymbol{\alpha }} + s_{\omega \omega '})\\  &\qquad \qquad \cdot \frac{\partial ^{2}}{ \partial \psi ^{(<h)-}_{{\boldsymbol{q}} + (n-m'){\boldsymbol{\alpha }} + s_{\omega \omega '}, \omega '} \partial \psi ^{(<h)+}_{{\boldsymbol{q}} + m{\boldsymbol{\alpha }},\omega }} \log \mathbb{E}_{h}\Big ( e^{V^{(h)}(\psi ^{(\le h)}, 0)} \Big )\Big |_{\psi ^{(<h)} = 0}; \end{aligned} \end{aligned}$$

to obtain the expression, we used that differentiating the integrand with respect to $\psi ^{(h)}$ or $\psi ^{(<h)}$ is equivalent, and the fact that the average of an odd number of Grassmann variables is zero. In terms of the definition of the effective potential:

B.6 $$\begin{aligned} \begin{aligned}&\frac{\mathbb{E}_{h}\Big ( \psi ^{(\le h)-}_{{\boldsymbol{q}} + m{\boldsymbol{\alpha }}, \omega } \psi ^{(\le h)+}_{{\boldsymbol{q}} + (n-m'){\boldsymbol{\alpha }} + s_{\omega \omega '}, \omega '} e^{V^{(h)}(\psi ^{(\le h)}, 0)} \Big ) }{\mathbb{E}_{h}\Big ( e^{V^{(h)}(\psi ^{(\le h)}, 0)} \Big )}\Big |_{\psi ^{(<h)} = 0} \\&\quad = \delta _{\omega ,\omega '}\delta _{n-m',m} \beta L_{1} g^{(h)}_{\omega }({\boldsymbol{q}} + m{\boldsymbol{\alpha }})\\&\qquad + \beta L_{1} g^{(h)}_{\omega }({\boldsymbol{q}} + m{\boldsymbol{\alpha }}) g^{(h)}_{\omega '}({\boldsymbol{q}} + (n-m'){\boldsymbol{\alpha }} + s_{\omega \omega '}) V^{(h-1)}_{\omega ,\omega ';n-m'-m}({\boldsymbol{q}} + m{\boldsymbol{\alpha }}). \end{aligned} \end{aligned}$$

Inserting this expression in (B.2), we find, recalling (B.1):

B.7 $$\begin{aligned} \begin{aligned}&W_{\phi \phi ;n,\sigma ,\zeta }^{(h)}({\boldsymbol{k}}; x_{2}, y_{2}) \\&\quad = \sum _{m,\omega } W^{(h)}_{\phi \psi ;m,\omega ,\sigma }({\boldsymbol{q}}, x_{2}) g^{(h)}_{\omega }({\boldsymbol{q}} + m{\boldsymbol{\alpha }}) W^{(h)}_{\psi \phi ;n-m,\omega ,\zeta }({\boldsymbol{q}} + m{\boldsymbol{\alpha }}, y_{2}) \\&\quad \quad + \sum _{\begin{array}{c} m,\omega \\ m',\omega ' \end{array}} W^{(h)}_{\phi \psi ;m,\omega ,\sigma }({\boldsymbol{q}}, x_{2}) g^{(h)}_{\omega }({\boldsymbol{q}} + m{\boldsymbol{\alpha }}) V^{(h-1)}_{\omega ,\omega '; n-m'-m}({\boldsymbol{q}} + m{\boldsymbol{\alpha }}) \\&\qquad \quad \cdot g^{(h)}_{\omega '}({\boldsymbol{q}} + (n-m') {\boldsymbol{\alpha }} + s_{\omega \omega '}) W^{(h)}_{\psi \phi ;m',\omega ',\zeta }({\boldsymbol{q}} + (n-m'){\boldsymbol{\alpha }} + s_{\omega \omega '}, y_{2}). \end{aligned} \end{aligned}$$

This proves (4.133), after the change of variables $n-m^{\prime }-m\to m^{\prime }$.

**Proof of** (4.134). The starting point is the formula, for ${\boldsymbol{q}} = {\boldsymbol{k}} - {\boldsymbol{k}}_{F}^{\omega }$:

B.8 $$\begin{aligned} W^{(h-1)}_{\phi \psi ;n,\omega ,\sigma }({\boldsymbol{q}}, x_{2}) = \beta L_{1} \frac{\partial ^{2}}{\partial \psi ^{(<h)-}_{{\boldsymbol{q}} + n{\boldsymbol{\alpha }}, \omega } \partial \phi ^{+}_{{\boldsymbol{k}}, x_{2}, \sigma } } \log \mathbb{E}_{h}\Big ( e^{V^{(h)}(\psi ^{(\le h)}, \phi )} \Big )\Big |_{\phi =\psi ^{(<h)} = 0}. \nonumber \\ \end{aligned}$$

A straightforward computation gives:

B.9 $$\begin{aligned} \begin{aligned}&\frac{\partial ^{2}}{\partial \psi ^{(<h)-}_{{\boldsymbol{q}} + n{\boldsymbol{\alpha }}, \omega } \partial \phi ^{+}_{{\boldsymbol{k}}, x_{2}, \sigma } } \log \mathbb{E}_{h}\Big ( e^{V^{(h)}(\psi ^{(\le h)}, \phi )} \Big ) = \frac{1}{\beta L_{1}} W^{(h)}_{\phi \psi ;n,\omega ,\sigma }({\boldsymbol{q}}, x_{2})\\&\qquad - \frac{1}{\beta L_{1}} \sum _{m,\omega '} W^{(h)}_{\phi \psi ;m,\omega ',\sigma }({\boldsymbol{q}} + s_{\omega \omega '}, x_{2}) \frac{\mathbb{E}_{h}\Big ( \psi ^{(\le h)-}_{{\boldsymbol{q}} + m{\boldsymbol{\alpha }} + s_{\omega \omega '}, \omega '} \frac{\partial }{\partial \psi ^{(<h)-}_{{\boldsymbol{q}} + n{\boldsymbol{\alpha }}, \omega }} e^{V^{(h)}(\psi ^{(\le h)}, \phi )}\Big )}{\mathbb{E}_{h}\Big ( e^{V^{(h)}(\psi ^{(\le h)}, \phi )} \Big )}, \end{aligned}\nonumber \\ \end{aligned}$$

and by (B.3) we have:

B.10 $$\begin{aligned} \begin{aligned}&\mathbb{E}_{h}\Big ( \psi ^{(\le h)-}_{{\boldsymbol{q}} + m{\boldsymbol{\alpha }} + s_{\omega \omega '}, \omega '} \frac{\partial }{\partial \psi ^{(<h)-}_{{\boldsymbol{q}} + n{\boldsymbol{\alpha }}, \omega }} e^{V^{(h)}(\psi ^{(\le h)}, \phi )}\Big )\Big |_{\psi ^{(<h)} = \phi = 0} \\&\quad = \beta L_{1} g^{(h)}_{\omega '}({\boldsymbol{q}} + m{\boldsymbol{\alpha }} + s_{\omega \omega '}) \frac{\partial ^{2}}{\partial \psi ^{(<h)+}_{{\boldsymbol{q}} + m{\boldsymbol{\alpha }} + s_{\omega \omega '}, \omega '} \partial \psi ^{(<h)-}_{{\boldsymbol{q}} + n{\boldsymbol{\alpha }}, \omega }} \mathbb{E}_{h}\Big ( e^{V^{(h)}(\psi ^{(\le h)}, \phi )}\Big )\Big |_{\psi ^{(<h)} = 0}. \end{aligned}\nonumber \\ \end{aligned}$$

Thus, plugging this identity in (B.9), we obtain (4.134):

B.11 $$\begin{aligned} \begin{aligned} W^{(h-1)}_{\phi \psi ;n,\omega ,\sigma }&({\boldsymbol{q}}, x_{2}) = W^{(h)}_{\phi \psi ;n,\omega ,\sigma }({\boldsymbol{q}}, x_{2}) + \sum _{m,\omega '} W^{(h)}_{\phi \psi ;m,\omega ',\sigma }({\boldsymbol{q}} + s_{\omega \omega '}, x_{2})\\&\quad \cdot g^{(h)}_{\omega '}({\boldsymbol{q}} + m{\boldsymbol{\alpha }} + s_{\omega \omega '}) V^{(h-1)}_{\omega ',\omega ;n-m}({\boldsymbol{q}} + m{\boldsymbol{\alpha }} + s_{\omega \omega '}). \end{aligned} \end{aligned}$$

## The Anomalous Bubble Diagram

In this appendix we shall compute the anomalous bubble diagram $\mathcal {B}_{+}^{\beta , L_{1}}(\boldsymbol{p})$, and recover the expression in Eq. (5.120). The computation is well-known, but we reproduce it here for completeness. Let $\boldsymbol{p}:=(\eta , p)$. For $\beta , L \gg 1$:

C.1 $$\begin{aligned} \begin{aligned} \mathcal {B}_{+}^{\beta , L_{1}}(\boldsymbol{p})&= \frac{1}{\beta L_{1}} \sum _{\boldsymbol{q} \in \mathscr {D}_{\beta ,L}} g_{+;\textrm{s}}(\boldsymbol{p}+\boldsymbol{q}) g_{+;\textrm{s}}(\boldsymbol{q})\\&=\underbrace{\int \frac{ d \boldsymbol{q}}{(2\pi )^2}\bigg (\frac{\chi ( \boldsymbol{p}+\boldsymbol{q})}{iv_{0,+}(\lambda )(\eta +q_0)+v_{1,+}(\lambda )(p+q_1)} \frac{\chi (\boldsymbol{q})}{iv_{0,+}(\lambda )q_0+v_{1,+}(\lambda )q_1} \bigg )}_{=:\mathcal {B}_{+}^{\infty }(\boldsymbol{p})}\\&\quad +\textrm{o}_{\beta , L}(1). \end{aligned} \nonumber \\ \end{aligned}$$

Then,

C.2 $$\begin{aligned} \begin{aligned} \mathcal {B}_{+}^{\infty }(\boldsymbol{p})&= \int \frac{ d \boldsymbol{q}}{(2\pi )^2} \,\frac{\chi ( \boldsymbol{q})\chi (\boldsymbol{p}+\boldsymbol{q} )}{iv_{0,+}(\lambda )\eta +v_{1,+}(\lambda )p} \bigg (\frac{1}{iv_{0,+}(\lambda )q_0+v_{1,+}(\lambda )q_1 }\\&\quad -\frac{1}{iv_{0,+}(\lambda )(\eta +q_0)+v_{1,+}(\lambda )(p+q_1)}\bigg )\\&= \int \frac{ d \boldsymbol{q}}{(2\pi )^2} \,\frac{\chi (\boldsymbol{q})\big (\chi (\boldsymbol{p}+\boldsymbol{q})-\chi (\boldsymbol{q}-\boldsymbol{p})\big )}{iv_{0,+}(\lambda )\eta +v_{1,+}(\lambda )p}\frac{1}{iv_{0,+}(\lambda )q_0+v_{1,+}(\lambda )q_1 }, \end{aligned} \end{aligned}$$

which we further rewrite as, by Taylor expanding in ${\boldsymbol{p}}$:

C.3 $$\begin{aligned} \begin{aligned} \mathcal {B}_{+}^{\infty }(\boldsymbol{p})&=\frac{2\eta }{iv_{0,+}(\lambda )\eta +v_{1,+}(\lambda )p} \underbrace{\int \frac{ d \boldsymbol{q}}{(2\pi )^2} \frac{\chi (\boldsymbol{q})\partial _0 \chi (\boldsymbol{q})}{iv_{0,+}(\lambda )q_0+v_{1,+}(\lambda )q_1 }}_{=:I_0} \\&\quad +\frac{2 p}{iv_{0,+}(\lambda )\eta +v_{1,+}(\lambda ) p} \underbrace{\int \frac{ d \boldsymbol{q}}{(2\pi )^2} \frac{ \chi (\boldsymbol{q})\partial _1\chi (\boldsymbol{q})}{iv_{0,+}(\lambda )q_0+v_{1,+}(\lambda ) q_1 }}_{=:I_1} +\mathrm O(\Vert \boldsymbol{p}\Vert ). \end{aligned} \end{aligned}$$

We recall that, with a slight abuse of notation, $\chi (\boldsymbol{q})=\chi \Big (\sqrt{\big (v_{0,+}(\lambda )q_{0}\big )^2+\big (v_{1,+}(\lambda )q_{1}\big )^2}\Big )$. Thus, after the change of variable $\kappa _{0} = v_{0,+}(\lambda ) q_{0}$ and $\kappa _{1} = v_{1,+}(\lambda ) q_{1}$, one easily gets:

C.4 $$\begin{aligned} I_0= &   \frac{1}{|{v}_{1,+}(\lambda ) |} \int \frac{ d \boldsymbol{\kappa }}{(2\pi )^2} \frac{\kappa _0 \, \chi (\Vert \boldsymbol{\kappa }\Vert )}{ \Vert \boldsymbol{\kappa }\Vert }\frac{\chi '(\Vert \boldsymbol{\kappa }\Vert )}{i \kappa _0+\kappa _1}\nonumber \\  &   = -\frac{i}{\mathfrak {v}(\lambda ) } I_1, \end{aligned}$$

that is $I_1=i\mathfrak {v}(\lambda )I_{0}$. The integral in $I_0$ can be computed explicitly:

C.5 $$\begin{aligned} \begin{aligned} \int \frac{ d \boldsymbol{\kappa }}{(2\pi )^2} \frac{\kappa _0 \chi (\Vert \boldsymbol{\kappa }\Vert )}{\Vert \boldsymbol{\kappa }\Vert }\frac{\chi '(\Vert \boldsymbol{\kappa }\Vert )}{i \kappa _0+\kappa _1}&= \int \frac{ d \theta \, d \rho }{(2\pi )^2}\,\rho \,\frac{\rho \cos \theta \,\chi (\rho )\chi '(\rho )}{\rho (i \rho \cos \theta +\rho \sin \theta )}\\&= { \left. \hspace{0.0pt}\frac{1}{2}\big (\chi \big )^2(\rho ) \phantom {\Big |} \right| _{0} }^{\hspace{-3.33328pt}\updelta } \int \frac{ d \theta }{(2\pi )^2} {\cos \theta \big (-i \cos \theta +\sin \theta \big )} = \frac{i}{8\pi }. \end{aligned} \nonumber \\ \end{aligned}$$

In conclusion:

C.6 $$\begin{aligned} \begin{aligned} \mathcal {B}_{+}^{\infty }(\boldsymbol{p})&=\frac{1}{4\pi \big | v_{0,+}(\lambda ) v_{1,+}(\lambda )\big |}\frac{iv_{0,+}(\lambda )\eta -v_{1,+}(\lambda )p}{i v_{0,+}(\lambda )\eta +v_{1,+}(\lambda )p} +\mathrm O(\Vert \boldsymbol{p}\Vert ). \end{aligned} \end{aligned}$$

## Proof of (5.55)

In this appendix we shall prove a momentum-space estimates for the remainder term in the expression of the two-point function, Eq. (5.55), which is used the analysis of the vertex Ward identity of Sect. 5.3.1. Suppose that ${\boldsymbol{k}}$ is close to ${\boldsymbol{k}}_{F}^{+}$, and that, for M≥1:

D.1 $$\begin{aligned} |k - k_{F}^{+}|^{M} \le |k_{0}| \le |k - k_{F}^{+}|. \end{aligned}$$

Then, recalling the notation ${\hat{R}}_{n,\sigma ,\zeta }({\boldsymbol{k}}; x_{2}, y_{2}) = {\hat{R}}_{\sigma ,\zeta }({\boldsymbol{k}}, {\boldsymbol{k}} + n{\boldsymbol{\alpha }}; x_{2}, y_{2})$, and setting ${\hat{R}}_{n,\sigma ,\zeta } = {\hat{R}}_{1;n,\sigma ,\zeta } + {\hat{R}}_{2;n,\sigma ,\zeta }$ where ${\hat{R}}_{i;n,\sigma ,\zeta }$ are defined in Sect. 4.3.2, we claim that, for $L_{2}$ large enough:

D.2 $$\begin{aligned} \begin{aligned} \big | \text {d}_{k_{0}}^{n_{0}} \text {d}_{k_{1}}^{n_{1}} {\hat{R}}_{n,\sigma ,\zeta }({\boldsymbol{k}}; x_{2}, y_{2}) \big |&\le \frac{C_{n_{0},n_{1}} |\lambda |^{\delta _{n\ne 0}} e^{-\frac{c}{16}|n|} e^{-{\tilde{c}}|x_{2} - y_{2}|}}{\Vert {\boldsymbol{k}} - {\boldsymbol{k}}_{F}^{+} \Vert ^{n_{0} + n_{1} + 1-\xi }}\\  &\quad + \sum _{\begin{array}{c} \omega _{1},\omega _{2} \\ (\omega _{1},\omega _{2}) \ne (+,+) \end{array}} \!\!\! C_{n_{0},n_{1}}\frac{e^{-\frac{c}{16}|n|} e^{-c(|x_{2}|_{\omega _{1}} + |y_{2}|_{\omega _{2}})}}{|k_{0}|^{n_{0} + n_{1} + 1-\xi }}. \end{aligned} \end{aligned}$$

The bound holds true for ${\hat{R}}_{2;n,\sigma ,\zeta }({\boldsymbol{k}}; x_{2}, y_{2})$, thanks to (4.156). Let us now prove it for ${\hat{R}}_{1;n,\sigma ,\zeta }({\boldsymbol{k}}; x_{2}, y_{2})$ defined as in (4.177). Let us denote by ${\hat{R}}_{1;+,n,\sigma ,\zeta }({\boldsymbol{k}}; x_{2}, y_{2})$ the contribution to ${\hat{R}}_{1;n,\sigma ,\zeta }({\boldsymbol{k}}; x_{2}, y_{2})$ coming from all chiralities equal to +. Then, by the exponential decay of the edge modes, and using that $\gamma ^{h+1} \ge |k_{0}|$, we easily get:

D.3 $$\begin{aligned}  &   \Big | \text {d}_{k_{0}}^{n_{0}} \text {d}_{k_{1}}^{n_{1}} \Big ({\hat{R}}_{1;n,\sigma ,\zeta }({\boldsymbol{k}}; x_{2}, y_{2}) - {\hat{R}}_{1;+,n,\sigma ,\zeta }({\boldsymbol{k}}; x_{2}, y_{2}) \Big )\Big | \nonumber \\  &   \quad \le \sum _{\begin{array}{c} \omega _{1},\omega _{2} \\ (\omega _{1},\omega _{2}) \ne (+,+) \end{array}} \!\!\! C_{n_{0},n_{1}}\frac{e^{-\frac{c}{16}|n|} e^{-c(|x_{2}|_{\omega _{1}} + |y_{2}|_{\omega _{2}})}}{|k_{0}|^{n_{0} + n_{1} + 1-\xi }}. \end{aligned}$$

Next, consider ${\hat{R}}_{1;+,n,\sigma ,\zeta }({\boldsymbol{k}}; x_{2}, y_{2})$. Observe that ${\hat{R}}_{1;+,n,\sigma ,\zeta }$ is given by a sum over scales *h* of contributions of the form (4.158) or of the form (4.159). We organize the sum over *m* and over the scales *h* as:

D.4 $$\begin{aligned}  &   \sum _{h, m} (\cdots ) = \sum _{h, m} 1\!\!1(m=0)(\cdots ) \nonumber \\  &   + \sum _{m} \sum _{h:\, \gamma ^{h} \ge |k - k_{F}^{+}|}1\!\!1(m\ne 0)(\cdots ) + \sum _{m} \sum _{h:\, \gamma ^{h} < |k - k_{F}^{+}|} 1\!\!1(m\ne 0)(\cdots ); \end{aligned}$$

correspondingly, we write:

D.5 $$\begin{aligned}  &   {\hat{R}}_{1;+,n,\sigma ,\zeta }({\boldsymbol{k}}; x_{2}, y_{2}) \nonumber \\  &   \quad = {\hat{R}}^{(a)}_{1;+,n,\sigma ,\zeta }({\boldsymbol{k}}; x_{2}, y_{2}) + {\hat{R}}^{(b)}_{1;+,n,\sigma ,\zeta }({\boldsymbol{k}}; x_{2}, y_{2}) + {\hat{R}}^{(c)}_{1;+,n,\sigma ,\zeta }({\boldsymbol{k}}; x_{2}, y_{2}). \end{aligned}$$

Consider the term ${\hat{R}}^{(a)}_{1;+,n,\sigma ,\zeta }({\boldsymbol{k}}; x_{2}, y_{2})$. Here, the scale *h* of the contributions (4.158), (4.159) has to be such that $\delta \gamma ^{h-1} \le \Vert {\boldsymbol{k}} - {\boldsymbol{k}}_{F}^{+}\Vert \le \delta \gamma ^{h+1}$. Therefore, from the bounds (4.166), (4.173) we get:

D.6 $$\begin{aligned} \sum _{h} \Big |\text {d}_{k_{0}}^{n_{0}} \text {d}_{k_{1}}^{n_{1}}{\hat{R}}^{(a);(h)}_{1;+,n,\sigma ,\zeta }({\boldsymbol{k}}; x_{2}, y_{2})\Big | \le C_{n_{0},n_{1}} \frac{|\lambda |^{\delta _{n\ne 0}} e^{-\frac{c}{16}|n|} e^{- \frac{{\tilde{c}}}{32}x_{2}} e^{- \frac{{\tilde{c}}}{32}y_{2}}}{\Vert {\boldsymbol{k}} - {\boldsymbol{k}}^{+}_{F}\Vert ^{n_{0} + n_{1}+1-\xi }}. \end{aligned}$$

Consider now the term ${\hat{R}}^{(b)}_{1;n,\sigma ,\zeta }({\boldsymbol{k}}; x_{2}, y_{2})$. Here, using that ${\boldsymbol{k}} - {\boldsymbol{k}}_{F}^{+} + m\alpha$ has to be in the support of $g_{+}^{(h)}$, together with the Diophantine condition, we get:

D.7 $$\begin{aligned} \Big (m\ne 0,\quad | m \alpha |_{\mathbb {T}} \lesssim \gamma ^{h+1}\Big )\Rightarrow |m| \lesssim \gamma ^{-h/\tau }. \end{aligned}$$

Thus, we can use a fraction of the exponential decay in *m* of the bound for the kernel $W^{(h)}_{\phi \psi ;m,+,\sigma }$ to prove that:

D.8 $$\begin{aligned} \sum _{h} \Big |\text {d}_{k_{0}}^{n_{0}} \text {d}_{k_{1}}^{n_{1}}{\hat{R}}^{(b);(h)}_{1;+,n,\sigma ,\zeta }({\boldsymbol{k}}; x_{2}, y_{2})\Big | \le C_{n_{0},n_{1}} |\lambda |^{\delta _{n\ne 0}} e^{-\frac{c}{16}|n|} e^{- \frac{{\tilde{c}}}{32}x_{2}} e^{- \frac{{\tilde{c}}}{32}y_{2}}. \end{aligned}$$

Finally, consider the last term in (D.4). Here, we use that the last scale is determined by $k_{0}$, since $\delta \gamma ^{h+1} \ge |k_{0}|$. Also, using that $|k - k_{F}^{+} + m\alpha |_{\mathbb {T}} \le \gamma ^{h+1}$, that $\gamma ^{h} < |k - k_{F}^{+}|$, and hence that $|m\alpha |_{\mathbb {T}} \le \gamma |k - k_{F}^{+}| + |k-k_{F}^{+}| \lesssim \gamma ^{h+1}$, the Diophantine condition in this regime implies:

D.9 $$\begin{aligned} \Big (m\ne 0,\quad | m \alpha |_{\mathbb {T}} \le 2\gamma |k - k_{F}^{+}|\Big )\Rightarrow |m| \ge C|k-k_{F}^{+}|^{-\frac{1}{\tau }}. \end{aligned}$$

Therefore, using again a fraction of the exponential decay in *m* of the bound for the kernel $W^{(h)}_{\phi \psi ;m,+,\sigma }$, we get:

D.10 $$\begin{aligned} \begin{aligned} \sum _{h} \Big | \text {d}_{k_{0}}^{n_{0}} \text {d}_{k_{1}}^{n_{1}} {\hat{R}}^{(c);(h)}_{1;+,n,\sigma ,\zeta }({\boldsymbol{k}}; x_{2}, y_{2})\Big |&\le C_{n_{0},n_{1}} \frac{|\lambda |^{\delta _{n\ne 0}} e^{-\frac{c}{16}|n|} e^{- \frac{{\tilde{c}}}{32}x_{2}} e^{- \frac{{\tilde{c}}}{32}y_{2}}}{|k_{0}|^{n_{0} + n_{1}+1-\xi }} e^{-\delta |k - k_{F}^{+}|^{-\frac{1}{\tau }}} \\&\le C_{n_{0},n_{1}} \frac{|\lambda |^{\delta _{n\ne 0}} e^{-\frac{c}{16}|n|} e^{- \frac{{\tilde{c}}}{32}x_{2}} e^{- \frac{{\tilde{c}}}{32}y_{2}}}{\Vert {\boldsymbol{k}} - {\boldsymbol{k}_{F}^{+}} \Vert ^{1-\xi }}, \end{aligned} \end{aligned}$$

where we used that, thanks to the assumption (D.1):

D.11 $$\begin{aligned} \begin{aligned} \frac{e^{-\delta |k - k_{F}^{+}|^{-\frac{1}{\tau }}}}{|k_{0}|^{n_{0} + n_{1} + 1-\xi }}&= \frac{e^{-\delta |k - k_{F}^{+}|^{-\frac{1}{\tau }}}}{\Vert {\boldsymbol{k}} - {\boldsymbol{k}_{F}^{+}} \Vert ^{n_{0} + n_{1} + 1-\xi }} \frac{\Vert {\boldsymbol{k}} - {\boldsymbol{k}_{F}^{+}} \Vert ^{n_{0} + n_{1} + 1-\xi }}{|k_{0}|^{n_{0} + n_{1} + 1-\xi }} \\&\le K\frac{e^{-\delta |k - k_{F}^{+}|^{-\frac{1}{\tau }}}}{\Vert {\boldsymbol{k}} - {\boldsymbol{k}_{F}^{+}} \Vert ^{n_{0} + n_{1} + 1-\xi }} \frac{| k - k_{F}^{+} |^{n_{0} + n_{1} + 1-\xi }}{|k - k_{F}^{+}|^{M(n_{0} + n_{1} + 1-\xi )}} \le \frac{K_{n_{0}, n_{1}}}{\Vert {\boldsymbol{k}} - {\boldsymbol{k}_{F}^{+}} \Vert ^{n_{0} + n_{1} + 1-\xi }}. \end{aligned} \end{aligned}$$

Thus, Eqs. (D.6), (D.8), (D.10) prove the momentum-space estimate (D.2).

## Proof of Lemma 5.5

Let us rewrite the function $g(\eta ,\theta )$ as:

E.1 $$\begin{aligned} g(\eta , \theta ) = \frac{\theta }{L_{1}}\sum _{p \in \mathscr {D}_{L}} \sum _{m\in \mathbb {Z}} \sum _{x_{2},y_{2}}{\hat{\mu }}_{\theta }(p, x_{2}) {\hat{\phi }}_{\theta ,\ell }(-p+m\alpha ,y_{2}) {\hat{F}}_{m}(\eta , p; x_{2}, y_{2}) \nonumber \\ \end{aligned}$$

where

E.2 $$\begin{aligned} {\hat{F}}_{m}(\eta , p; x_{2}, y_{2}) = \int _{-\beta /2}^{\beta /2} ds\, e^{-i\eta s} \sum _{x_{1} = 1}^{L_{1}} e^{-ip_{1}z_{1}} F_{m}((s, z_{1}); x_{2}, y_{2}). \end{aligned}$$

Let us change variable, $p \rightarrow \theta p$:

E.3 $$\begin{aligned} g(\eta , \theta ) = \frac{\theta }{L_{1}}\sum _{p \in \frac{1}{\theta }\mathscr {D}_{L}} \sum _{m\in \mathbb {Z}} \sum _{x_{2},y_{2}}{\hat{\mu }}_{\theta }(\theta p, x_{2}) \hat{\phi }_{\theta ,\ell }(-\theta p+m\alpha ,y_{2}) {\hat{F}}_{m}(\eta , \theta p; x_{2}, y_{2}), \nonumber \\ \end{aligned}$$

where, by (5.108), the functions $\hat{\mu }_{\theta }(\theta q, x_{2})$, ${\hat{\phi }}_{\theta ,\ell }(\theta q,y_{2})$ decay faster than any power in $|q|_{\mathbb {T}_{\theta ^{-1}}}$. Thus, in the sum over *m* we isolate the term with m=0 from the rest:

E.4 $$\begin{aligned} \begin{aligned} g(\eta , \theta )&= \frac{\theta }{L_{1}}\sum _{p \in \frac{1}{\theta }\mathscr {D}_{L}} \sum _{x_{2},y_{2}}{\hat{\mu }}_{\theta }(\theta p, x_{2}) {\hat{\phi }}_{\theta ,\ell }(-\theta p,y_{2}) {\hat{F}}_{0}(\eta , \theta p; x_{2}, y_{2})\\&\quad + \frac{\theta }{L_{1}}\sum _{p \in \frac{1}{\theta }\mathscr {D}_{L}} \sum _{m\ne 0} \sum _{x_{2},y_{2}}{\hat{\mu }}_{\theta }(\theta p, x_{2}) {\hat{\phi }}_{\theta ,\ell }(-\theta (p + m\alpha /\theta ),y_{2}) {\hat{F}}_{m}(\eta , \theta p; x_{2}, y_{2})\\&=: g_{0}(\eta , \theta ) + g_{>}(\eta , \theta ). \end{aligned} \nonumber \\ \end{aligned}$$

As a consequence of the assumptions on *F*, we have

E.5 $$\begin{aligned} |{\hat{F}}_{m}(\eta , \theta p; x_{2}, y_{2})|\le Ce^{-c|m|} e^{-c|x_{2} - y_{2}|} \end{aligned}$$

uniformly in all other parameters; hence, we can estimate:

E.6 $$\begin{aligned} \begin{aligned} |g_{>}(\eta , \theta )|&\le \frac{1}{\theta L_{1}}\sum _{p \in \frac{1}{\theta }\mathscr {D}_{L}} \sum _{m\ne 0} \sum _{x_{2},y_{2}} \frac{C_{r}}{1+(|p|_{\mathbb {T}_{\theta ^{-1}}})^{r}} \frac{1}{1+(|p + m\alpha /\theta |_{\mathbb {T}_{\theta ^{-1}}})^{r}} \\  &\quad \cdot \frac{1}{1 + | \theta x_{2}|^{r}} \frac{1}{1+|y_{2} / \ell |^{r}} e^{-c|m|} e^{-c|x_{2} - y_{2}|}. \end{aligned} \end{aligned}$$

We then separate the values of *m* such that $| m\alpha |_{\mathbb {T}} > \sqrt{\theta }$ and $| m\alpha |_{\mathbb {T}} \le \sqrt{\theta }$; both contributions are small as $\theta \rightarrow 0$, for different reasons. For the former, we proceed as follows:

E.7 $$\begin{aligned} \begin{aligned}&\frac{1}{1+(|p|_{\mathbb {T}_{\theta ^{-1}}})^{r}} \frac{1}{1+(|p + m\alpha /\theta |_{\mathbb {T}_{\theta ^{-1}}})^{r}} \\&\quad \le \frac{C_{r}}{1+(|p|_{\mathbb {T}_{\theta ^{-1}}})^{r/2}} \frac{1}{1+(|p|_{\mathbb {T}_{\theta ^{-1}}})^{r/2}} \frac{1}{1+(|p + m\alpha /\theta |_{\mathbb {T}_{\theta ^{-1}}})^{r/2}} \\&\quad \le \frac{K_{r}}{1+(|p|_{\mathbb {T}_{\theta ^{-1}}})^{r/2}} \frac{1}{1+(|p|_{\mathbb {T}_{\theta ^{-1}}})^{r/2}} \frac{|p + m\alpha /\theta |^{r/2}_{\mathbb {T}_{\theta ^{-1}}} + |p|^{r/2}_{\mathbb {T}_{\theta ^{-1}}}}{1+(|p + m\alpha /\theta |_{\mathbb {T}_{\theta ^{-1}}})^{r/2}} \frac{1}{1+(|m\alpha /\theta |_{\mathbb {T}_{\theta ^{-1}}})^{r/2}} \\&\quad \le \frac{2K_{r}}{1+(|p|_{\mathbb {T}_{\theta ^{-1}}})^{r/2}} \frac{1}{1+(|m\alpha /\theta |_{\mathbb {T}_{\theta ^{-1}}})^{r/2}}, \end{aligned} \nonumber \\ \end{aligned}$$

and we use the smallness of the function $e^{-c|m|} | m\alpha /\theta |_{\mathbb {T}_{\theta ^{-1}}}^{-r/2}$ as $\theta \ll 1$. For the regime $| m\alpha |_{\mathbb {T}} \le \sqrt{\theta }$, we use the Diophantine condition; we omit the details. We obtain:

E.8 $$\begin{aligned} |g_{>}(\eta , \theta )| \le C_{\ell }\theta . \end{aligned}$$

Next, consider the term $g_{0}(\eta , \theta )$. Using that, as a consequence of assumption (5.85),

E.9 $$\begin{aligned} \Big | {\hat{F}}_{m}(\eta , \theta p; x_{2}, y_{2}) - {\hat{F}}_{m}(\eta , 0; x_{2}, y_{2}) \Big | \le C |\theta p|^{\xi /2} e^{-c|m|} e^{-c|x_{2} - y_{2}|}, \end{aligned}$$

we have:

E.10 $$\begin{aligned} \begin{aligned} g_{0}(\eta , \theta )&= \frac{\theta }{L_{1}}\sum _{p \in \frac{1}{\theta }\mathscr {D}_{L}} \sum _{x_{2},y_{2}}{\hat{\mu }}_{\theta }(\theta p, x_{2}) {\hat{\phi }}_{\theta ,\ell }(-\theta p,y_{2}) {\hat{F}}_{0}(\eta , 0; x_{2}, y_{2}) + {\tilde{g}}_{0}(\eta , \theta ) \\ |{\tilde{g}}_{0}(\eta , \theta )|&\le C_{\ell }\theta ^{\xi /2}. \end{aligned} \end{aligned}$$

Concerning the main term in (E.10), for $\theta \ll 1$ and as $L_{1}\rightarrow \infty$, we can approximate it as an integral:

E.11 $$\begin{aligned} \begin{aligned}&\frac{\theta }{L_{1}}\sum _{p \in \frac{1}{\theta }\mathscr {D}_{L}} \sum _{x_{2},y_{2}}{\hat{\mu }}_{\theta }(\theta p, x_{2}) {\hat{\phi }}_{\theta ,\ell }(-\theta p,y_{2}) {\hat{F}}_{0}(\eta , 0; x_{2}, y_{2}) \\&\quad = \sum _{x_{2}, y_{2}} \int _{\mathbb {R}} \frac{dp}{(2\pi )}\, {\hat{\mu }}(p, 0) {\hat{\varphi }}(-p, y_{2}/\ell ) {\hat{F}}_{0}(\eta , 0; x_{2}, y_{2}) + O_{\ell }(\theta ^{\alpha }). \end{aligned} \end{aligned}$$

for some α>0. All in all, we rewrote $g(\eta , \theta )$ as a quantity that does not depend on θ, given by the integral in (E.11), plus error terms that vanish with θ uniformly in all parameters except ℓ. In particular,

E.12 $$\begin{aligned} | g(\eta , \theta ) - g(\eta , \theta ') | \le C_{\ell } (|\theta |^{\alpha } + |\theta '|^{\alpha }) \end{aligned}$$

for some α>0. This concludes the proof of Lemma 5.5. □

## Footnotes

1 Observe that, in this representation, the perturbation is obtained from the initial Fock space Hamiltonian after a shift of 1/2 of the density operator. This is the reason for the overall constant in the right-hand side of (4.1).

2 It is given by:

4.34 $$\begin{aligned} {\tilde{z}}_{\textrm{b}} = z_{\textrm{b}} \prod _{{\boldsymbol{k}},\omega } \frac{\tilde{g}_{\omega }^{(\le 1)}({\boldsymbol{k}}) }{g_{\omega }^{(\le 1)}({\boldsymbol{k}})} \end{aligned}$$

3 The s=0 ambiguity in (5.5) has no relevance, since all operators are bounded, and it involves a zero measure set of times.
